# Enhanced degradation of softwood versus hardwood by the white-rot fungus *Pycnoporus coccineus*

**DOI:** 10.1186/s13068-015-0407-8

**Published:** 2015-12-18

**Authors:** Marie Couturier, David Navarro, Didier Chevret, Bernard Henrissat, François Piumi, Francisco J. Ruiz-Dueñas, Angel T. Martinez, Igor V. Grigoriev, Robert Riley, Anna Lipzen, Jean-Guy Berrin, Emma R. Master, Marie-Noëlle Rosso

**Affiliations:** Aix Marseille Université, UMR1163 Biodiversité et Biotechnologie Fongiques, 163 avenue de Luminy, 13288 Marseille, France; INRA, UMR1163 Biodiversité et Biotechnologie Fongiques, 163 avenue de Luminy, 13288 Marseille, France; Polytech’Marseille, UMR1163 Biodiversité et Biotechnologie Fongiques, 163 avenue de Luminy, 13288 Marseille, France; Department of Chemical Engineering and Applied Chemistry, University of Toronto, Toronto, ON Canada; INRA, UMR1319 Micalis, Plateforme d’Analyse Protéomique de Paris Sud-Ouest, 78352 Jouy-En-Josas, France; Architecture et Fonction des Macromolécules Biologiques (AFMB), UMR 7257 CNRS, Université Aix-Marseille, 13288 Marseille, France; Department of Biological Sciences, King Abdulaziz University, Jeddah, Saudi Arabia; INRA, USC 1408 AFMB, 13288 Marseille, France; CIB, CSIC, Ramiro de Maeztu 9, 28040 Madrid, Spain; US Department of Energy Joint Genome Institute (JGI), Walnut Creek, CA USA

**Keywords:** Carbohydrate-active enzymes, Lignin-active enzymes, *Pycnoporus coccineus*, Transcriptomics, Proteomics, ToF-SIMS, White-rot

## Abstract

**Background:**

White-rot basidiomycete fungi are potent degraders of plant biomass, with the ability to mineralize all lignocellulose components. Recent comparative genomics studies showed that these fungi use a wide diversity of enzymes for wood degradation. Deeper functional analyses are however necessary to understand the enzymatic mechanisms leading to lignocellulose breakdown. The Polyporale fungus *Pycnoporus coccineus* BRFM310 grows well on both coniferous and deciduous wood. In the present study, we analyzed the early response of the fungus to softwood (pine) and hardwood (aspen) feedstocks and tested the effect of the secreted enzymes on lignocellulose deconstruction.

**Results:**

Transcriptomic and proteomic analyses revealed that *P. coccineus* grown separately on pine and aspen displayed similar sets of transcripts and enzymes implicated in lignin and polysaccharide degradation. In particular, the expression of lignin-targeting oxidoreductases, such as manganese peroxidases, increased upon cultivation on both woods. The sets of enzymes secreted during growth on both pine and aspen were more efficient in saccharide release from pine than from aspen, and characterization of the residual solids revealed polysaccharide conversion on both pine and aspen fiber surfaces.

**Conclusion:**

The combined analysis of soluble sugars and solid residues showed the suitability of *P. coccineus* secreted enzymes for softwood degradation. Analyses of solubilized products and residual surface chemistries of enzyme-treated wood samples pointed to differences in fiber penetration by different *P. coccineus* secretomes. Accordingly, beyond the variety of CAZymes identified in *P. coccineus* genome, transcriptome and secretome, we discuss several parameters such as the abundance of manganese peroxidases and the potential role of cytochrome P450s and pectin degradation on the efficacy of fungi for softwood conversion.

**Electronic supplementary material:**

The online version of this article (doi:10.1186/s13068-015-0407-8) contains supplementary material, which is available to authorized users.

## Background

Degradation of lignocellulosic biomass is efficiently achieved in nature by many organisms, among which filamentous fungi are considered key primary degraders. In particular, white-rot basidiomycete fungi are able to completely degrade all lignocellulose components, including cellulose, hemicellulose and lignin. Detailed characterization of *Phanerochaete chrysosporium* [1–3], and genomic, transcriptomic or secretomic analyses of other white-rot fungi including *Phanerochaete carnosa* [4–6], *Irpex lacteus* [7], *Ceriporiopsis subvermispora* [8, 9], *Phlebiopsis gigantea* [10], and *Dichomitus squalens* [11] have described the main lignocellulolytic enzymes involved in the biodegradation of wood. The order Polyporales is a group of fungi comprising many wood-rotters, and therefore particularly important to the discovery of new and more efficient biocatalysts for applications in plant biomass conversion. As of 2014, 11 Polyporales genomes were available out of the 31 genomes sequenced from the Agaricomycetes class [12], which in turn spurred several comparative analyses of genome sequences [13, 14] and of substrate-dependent secretomes [15, 16].

While many white-rot fungi characterized to date appear to grow better on hardwood than coniferous softwood, certain white-rot fungi, including *P. carnosa* [6], *P. gigantea* [10], and *D. squalens* [11], grow efficiently on softwood. Differences in fungi’s ability to utilize coniferous wood as a carbon source have been attributed to softwood lignin and hemicellulose compositions as compared to most hardwood species. For example, whereas acetylated glucuronoxylan is the main hemicellulose polymer in hardwood tissues, galactoglucomannan is the most abundant in coniferous wood [17]. Lignin contents also differ, where the fraction of guaiacyl lignin in coniferous wood is generally higher than that of the hardwood guaiacyl–syringyl lignin, while also being more extensively cross-linked [18]. Finally woody biomass also contains extractive compounds that vary considerably between tree species and plant tissues. For example, certain extractives including some fatty acids and phenolics (e.g. tannins) are generally found in both softwoods and hardwoods, whereas terpenes generally are dominant in softwoods [18].

The *Pycnoporus* genus belongs to the Polyporales order, and the four *Pycnoporus* species (*P. cinnabarinus, P. sanguineus, P. coccineus* and *P. puniceus*) are categorized as white-rot fungi. *P. cinnabarinus* is widely distributed in the Northern hemisphere, whereas *P. coccineus* is found in countries bordering the Indian and Pacific oceans. *P. sanguineus* is a pan-tropical species encountered in both hemispheres, and *P. puniceus* is a rare species found in Africa, India, Malaysia and New Caledonia. The genome of *P. cinnabarinus* was released in 2014 [19] and was found to encode a full complement of carbohydrate-active enzymes (CAZyme) required for polysaccharide degradation including classical hydrolases; cellobiohydrolases from Glycoside Hydrolase (GH) families GH6 and GH7, endoglucanases (GH5, GH12, GH45, GH74, GH131), β-glucosidases (GH1, GH3); and 15 lytic polysaccharide monooxygenases (LPMO) from the Auxiliary Activity (AA) family AA9. In addition, *P. cinnabarinus* possesses a complete and versatile enzymatic arsenal for lignin breakdown. For instance, several genes encoding members of the three ligninolytic peroxidase subfamilies, namely lignin peroxidases (LiP), manganese peroxidases (MnP) and versatile peroxidases (VP) were identified. *P. cinnabarinus* has been used for biotechnological applications due to its ability to produce high-value compounds such as aromas and antioxidants (for a review see [20]).

In addition to *P. cinnabarinus*, the genome of *P. coccineus* has been recently released by the JGI (http://genome.jgi.doe.org/Pycco1/Pycco1.home.html). At the same time, our preliminary studies demonstrated that one strain of *P. coccineus*, namely *P. coccineus* CIRM-BRFM 310, grew better on both deciduous and coniferous wood preparations than *P. cinnabarinus* CIRM-BRFM 137. Accordingly, *P. coccineus* CIRM-BRFM 310 was selected to investigate the profile of expressed genes and secreted proteins in response to growth on deciduous (*Populus grandidentata*) and coniferous (*Pinus halepensis*) woods. The effect of the secretomes on the surface composition of cryo-milled wood and the analysis of the released saccharides showed that *P. coccineus* displays enzyme cocktails particularly well adapted to softwood conversion.

## Results

**Fig. 1:**
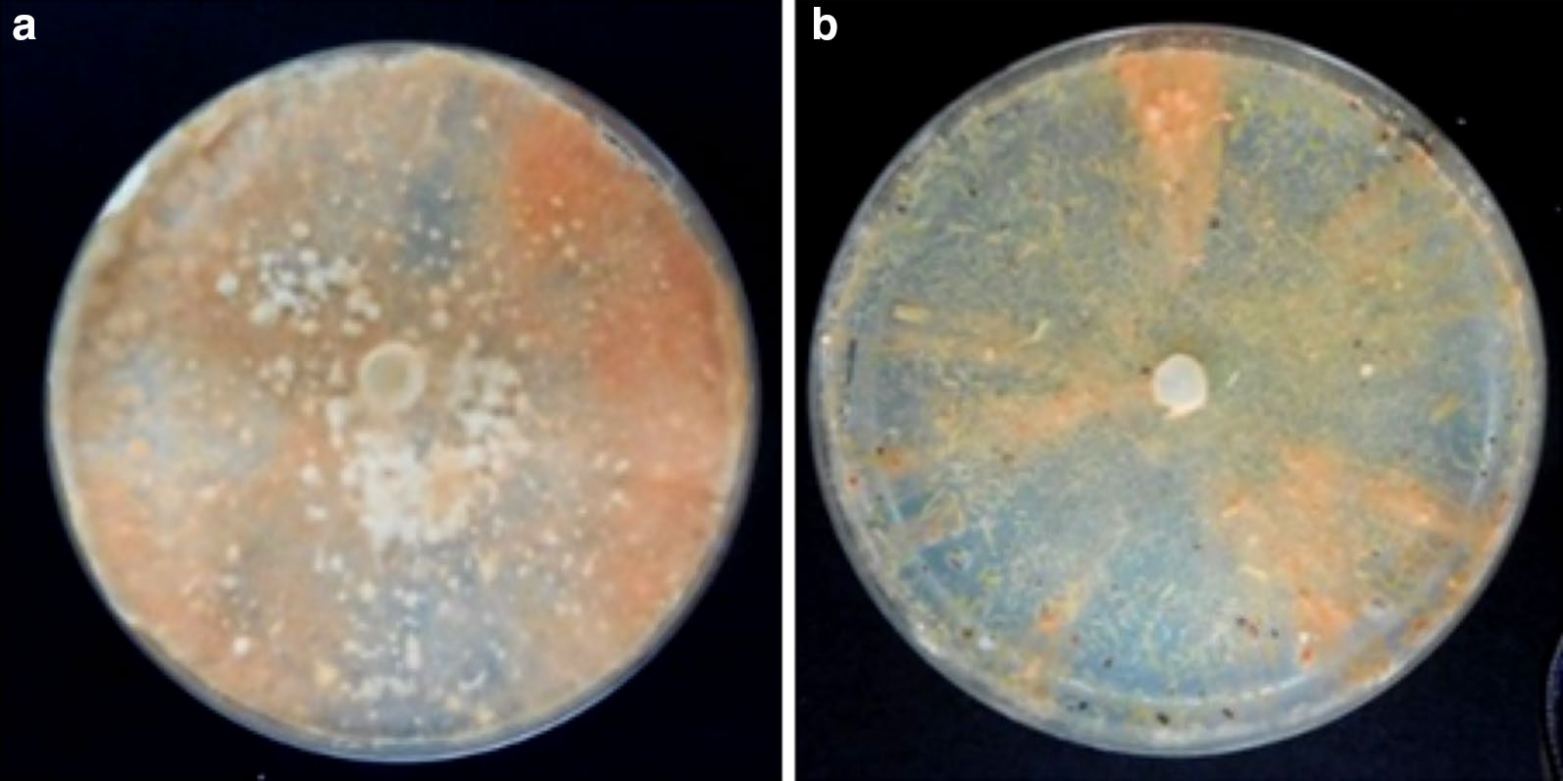
Growth of *P. coccineus* on wood. Cultivation of the fungus for 9 weeks at 30 °C on an agar plate containing **a** hardwood (aspen) or **b** softwood (pine) as the sole-carbon source

When cultivated on agar plates containing wood powder as the sole-carbon source, the *P. coccineus* monokaryotic strain CIRM-BRFM 310 was able to develop rapidly on hardwood (aspen). Softwood (pine) cultivation was not as fast, but the fungus was still able to grow (Fig. 1). The recent genome sequencing of *P. coccineus* CIRM-BRFM 310 revealed a 32.76-Mb genome with 12,690 predicted genes (to be discussed elsewhere). The genome was annotated using the JGI Annotation Pipeline and made available via the JGI fungal portal MycoCosm [21]. The repertoire of genes coding for predicted CAZymes and AA enzymes in the genome of *P. coccineus* CIRM-BRFM 310 was very similar to that of other Polyporales (Additional file 1, Additional file 2: Table S1). In an attempt to elucidate the mechanisms that permit *P. coccineus* growth on both hardwood and softwood fibers, the strain was selected for an in-depth study combining transcriptomics, proteomics and functional characterization.

### Transcripts related to lignocellulose degradation are enriched in wood grown cultures

**Fig. 2:**
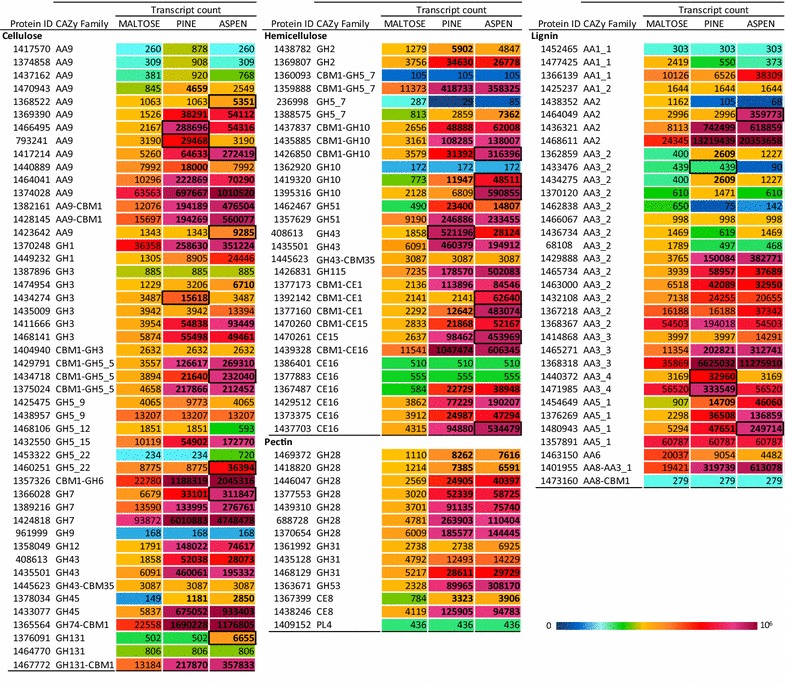
Heat maps and abundances of gene transcripts for selected CAZy families expressed during growth*. Bold numbers* highlight transcripts significantly more abundant in mycelia harvested from cultivations on wood than on maltose (at least fourfold difference). *Black frames* highlight transcripts significantly more enriched in one wood grown culture than the other (at least fourfold difference)

Transcriptomes were sequenced from triplicated independent three day-cultures under three different sets of conditions: (1) in the presence of maltose as the sole-carbon source, (2) in the presence of ground pine wood as the sole-carbon source, and (3) in the presence of ground aspen wood as the sole-carbon source. For each condition, RNAseq data quality was verified by performing qPCR on a sample set of eight genes (Additional file 3: Figure S1). Expression levels were in good agreement with transcriptomic data. In total, transcriptomic analyses revealed 267 transcript sequences predicted to encode CAZymes, which were classified into 71 families of the CAZy database (http://www.cazy.org, [22]). When applying a fourfold increase in relative transcript abundance as the significance threshold, 114 (43 %) and 105 (39 %) unique sequences were considered significantly more abundant in the samples obtained from pine and aspen, respectively, as compared to maltose (at least fourfold enrichment). Notably, 92 transcripts (34 % of total transcripts) were upregulated on both pine and aspen, and were largely assigned to CAZy families known to encode lignocellulolytic activities. These included predicted cellulases from families GH5_5, GH12, GH45, GH74, GH131; cellobiohydrolases from families GH6 and GH7; β-glucosidases from GH1 and GH3, and LPMOs from family AA9. Of note, 11 AA9 Lytic Polysaccharide Monooxygenase (LPMO) transcripts, out of the 15 AA9 encoded by *P. coccineus*, were between 4 and 120 times more abundant in at least one of the wood conditions (Fig. 2; Additional file 2: Table S2).

GH families that comprise hemicellulolytic activities were also represented in this set of differentially expressed transcripts, including putative GH5_7 endo-mannanases, GH2 β-mannosidase and GH27 α-galactosidase, as well as GH10 endo-xylanases, GH43 and GH51 α-arabinofuranosidases, and GH115 α-glucuronidases. Of particular note, transcripts corresponding to all seven of the polygalacturonases from family GH28 identified in *P. coccineus* genome were more abundant in both pine and aspen cultivations than maltose, as were transcript levels corresponding to other genes predicted to be involved in pectin degradation, i.e. the two carbohydrate esterases (CE) of family CE8 genes and the only gene predicted to encode a GH53 β-galactosidase.

Annotation of AA2 gene models identified five putative MnP genes in the *P*. *coccineus* genome, among which four were identified in transcriptomes after cultivation on wood, and three were significantly more abundant in at least one of these cultivations (genes encoding predicted proteins 1464049, 1436321 and 1468611). By contrast, no candidate LiP transcripts from any of the six genes were detected. Hydrogen peroxide production is central to lignin degradation as it is required for peroxidase activity. Accordingly, transcriptomes from cultivations on pine and aspen were respectively enriched in six and five unique transcripts predicted to encode family AA3_2 glucose methanol choline (GMC) oxidoreductases. Transcripts from three family AA5_1 glyoxal oxidases were also enriched in both transcriptomes (Fig. 2). In fact, the most abundant transcripts in both samples from cultivations on wood encoded a predicted AA2 MnP (1468611) and a predicted AA3_3 alcohol oxidase (1368318), consistent with there being a major role for oxidoreductase activities in lignocellulose conversion by *P. coccineus*.

Overall, *P. coccineus* transcriptomes obtained following growth on aspen or pine comprised similar levels of transcripts predicted to encode lignocellulose degradation activities. However, growth on aspen uniquely led to the enrichment of two AA9 LPMO transcripts (1423642 and 1417214), one AA2 MnP (1464049) and three GH10 xylanases (1395316, 1426850 and 1419320), the latter being consistent with differences in hemicellulose compositions in the two substrates (Fig. 2; Additional file 2: Table S2). Conversely, growth on pine was distinguished by the enrichment of two alternative AA9 LPMO transcripts (793241 and 1466495), one GH3 β-glucosidase (1434274) and two AA3_4 H_2_O_2_-producing pyranose oxidases (1471985 and 1440372). Unexpectedly, however, none of the 14 transcripts significantly more abundant in *P. coccineus* after growth on pine as compared to aspen was predicted to directly target galactoglucomannan.

### *Pycnoporus coccineus* displays similar sets of CAZymes upon growth on hardwood and softwood

**Fig. 3:**
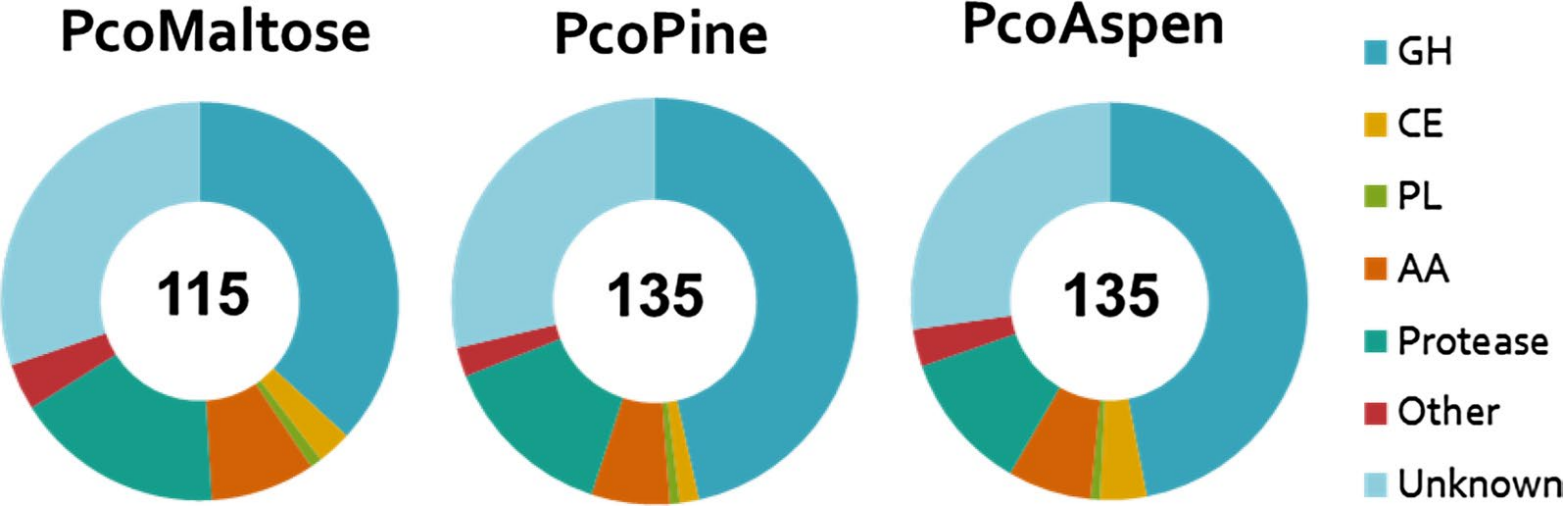
Distribution of *P. coccineus* proteins identified by LC–MS/MS in secretomes extracted from cultivations on maltose, pine and aspen. *GH* glycoside hydrolase, *PL* polysaccharide lyase, *CE* carbohydrate esterase, *AA* auxiliary activity

LC–MS/MS was subsequently used to identify secreted CAZymes contributing most to the activities of secretomes extracted after cultivation on pine (referred to herein as PcoPine) or on aspen (referred to herein as PcoAspen) (Additional file 3: Figure S2). The secretome extracted from control cultivation on maltose (referred to herein as PcoMaltose) was also analyzed for comparison. A total of 115, 135 and 135 proteins were identified in PcoMaltose, PcoPine and PcoAspen, respectively (Additional file 2: Table S3, Additional file 4). Most of the detected proteins (84 %) were common to the two wood cultivations. Signal peptides were predicted for 89 % of identified proteins. PcoPine and PcoAspen contained 73 and 78 CAZymes, respectively, versus 54 CAZymes in PcoMaltose (Fig. 3). These CAZymes were broadly categorized as GH (39 in PcoMaltose, 60 in PcoPine and 62 in PcoAspen), AA (9, 8, 9), CE (CE) (3, 2, 5) and polysaccharide lyases (PL) (1, 1, 1) in PcoMaltose, PcoPine and PcoAspen, respectively.

**Fig. 4:**
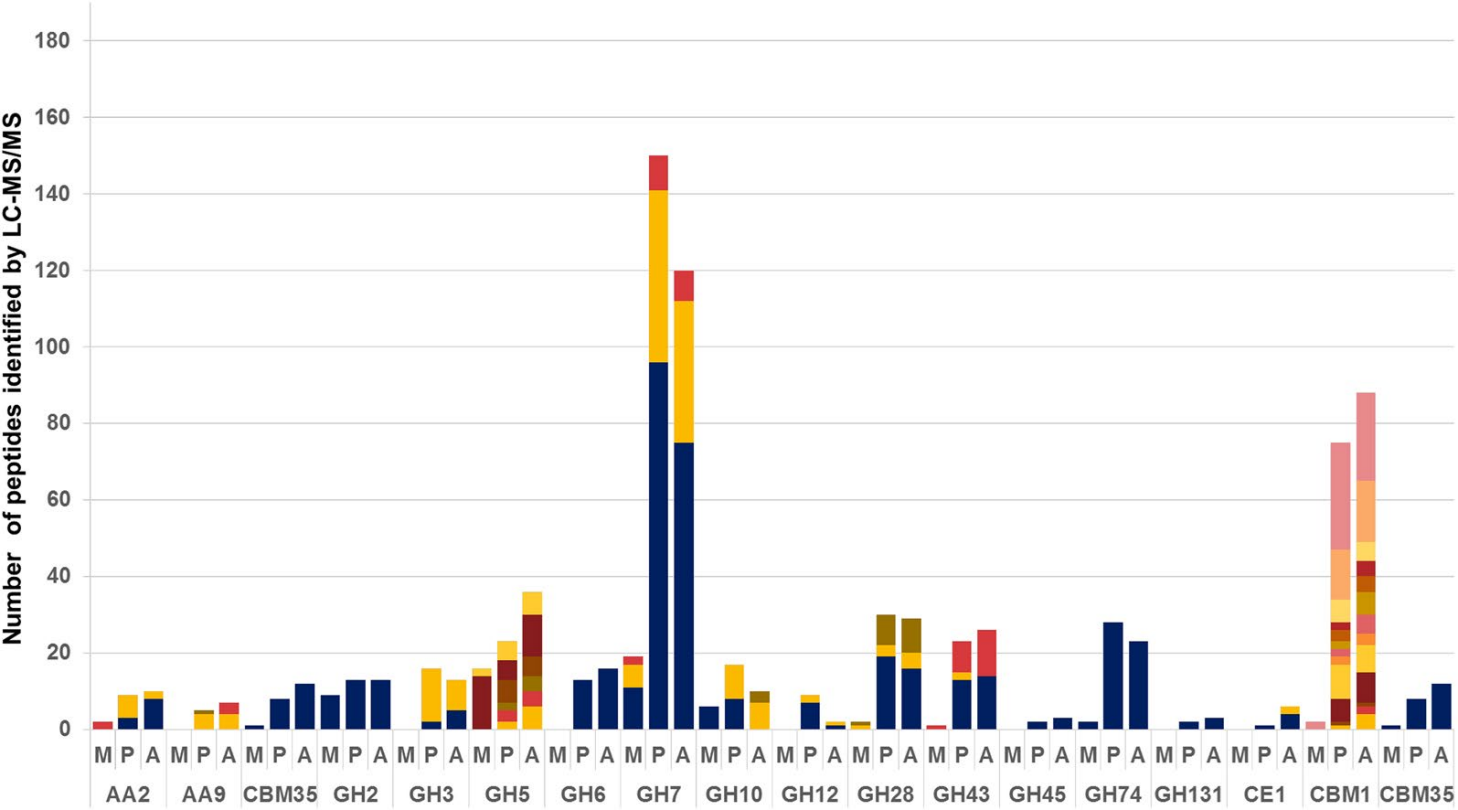
Number of representatives and peptide abundance for selected CAZy families identified in *P. coccineus* secretomes. *M* PcoMaltose, *P* PcoPine, *A* PcoAspen, *GH* glycoside hydrolase, *CBM* carbohydrate-binding module, *CE* carbohydrate esterase, *AA* auxiliary activity. *Colors* highlight different representatives of each family

Several CAZymes were identified in PcoPine and/or PcoAspen that were not detected in PcoMaltose (Table 1; Fig. 4). These included a total of 32 enzymes from CAZy families most often attributed to cellulose, hemicellulose, and pectin degradation, including GH families GH3, GH5_5, GH5_7, GH6, GH10, GH12, GH16, GH28, GH43 and GH45, as well as CE family CE1, and two AA9 LPMOs. Several AA families with activities attributed to lignin degradation, including enzymes from families AA2 and AA3, were also detected in the set of enzymes uniquely found in PcoPine and/or PcoAspen. Overall, the activities identified in the two wood conditions were globally similar, and the numbers of identified peptides suggested that most enzymes were produced in comparable amounts (Fig. 4). However, consistent with transcriptomic analyses, one AA2 MnP (1464049) was identified solely in PcoAspen, and one AA2 MnP (1436321) was only identified in PcoPine even though transcripts were identified in both cultivations on wood. Finally, whereas only one protein bearing a CBM1 module was detected in PcoMaltose, PcoPine and PcoAspen secretomes comprised 10 and 12 CBM1-containing CAZymes, respectively, which were largely attached to CAZy catalytic modules known to target cellulose: GH131 (1467772), GH3 (1404940), GH5_5 (1375024, 1429791 and 1434718), GH6 (1357326), or xylan: GH10 (1437837 and 1426850), and CE1 (1392142 and 1377173) (Table 1).

**Table 1:** CAZymes identified by LC–MS/MS in wood grown samples only

Protein ID	CAZY annotation	Number of detected peptides	
		PcoPine	PcoAspen
1452465	AA1_1	7	9
1436321	AA2	6	Nd
1464049	AA2	Nd	8
1401955	AA3_1-AA8	7	8
1374028	AA9	4	4
1423642	AA9	Nd	3
1377173	CBM1-CE1	Nd	4
1392142	CBM1-CE1	Nd	2
1411666	GH3	14	8
1404940	GH3-CBM1	2	5
1429791	CBM1-GH5_5	3	4
1375024	CBM1-GH5_5	2	6
1434718	CBM1-GH5_5	2	4
1359888	CBM1-GH5_7	6	5
1357326	CBM1-GH6_3	13	16
1366028	GH7	9	8
1437837	CBM1-GH10	9	7
1426850	CBM1-GH10	6	8
1395316	GH10	Nd	3
1358049	GH12	7	Nd
1447621	GH16_1	3	3
1372016	GH16	Nd	2
651762	GH16	2	Nd
1434602	GH16	3	Nd
1445051	GH18	17	7
1447824	GH18-CBM5	Nd	5
1463186	GH18-CBM5	Nd	5
1106774	GH18-CBM5	4	2
1463187	GH18-CBM5	Nd	3
1456924	GH18_14-CBM5 [×2]	Nd	6
688728	GH28_9	19	16
1439310	GH28	3	4
1370654	GH28	8	9
1358510	GH30	15	14
1435501	GH43	13	14
408613	GH43	2	Nd
1445623	GH43-CBM35	8	12
1433077	GH45	2	3
1377179	GH55	30	54
1361311	GH72-CBM43	3	3
1453868	GH92	Nd	2
1467772	GH131-CBM1	2	3

*Nd* not detected

### Other proteins potentially involved in biomass degradation

Cytochrome P450 monooxygenases can transform lignin fragments as well as aromatic extractives, and have been implicated in the detoxification of these compounds [23–25]. The *P. coccineus* genome comprises 205 genes predicted to encode P450 monooxygenases distributed among several P450 families. Among these, the transcripts of 86 P450 genes were identified in the *P. coccineus* transcriptomes collected herein, where 24 of them were more abundant (4–170 times) following cultivation on wood compared to maltose, and only 8 were differentially expressed between pine and aspen (Additional file 2: Table S4). Based on comparisons with curated fungal P450s (http://p450.riceblast.snu.ac.kr), these 24 P450 sequences largely grouped within clans 64 and 534 of P450 classification system [26, 27].

**Fig. 5:**
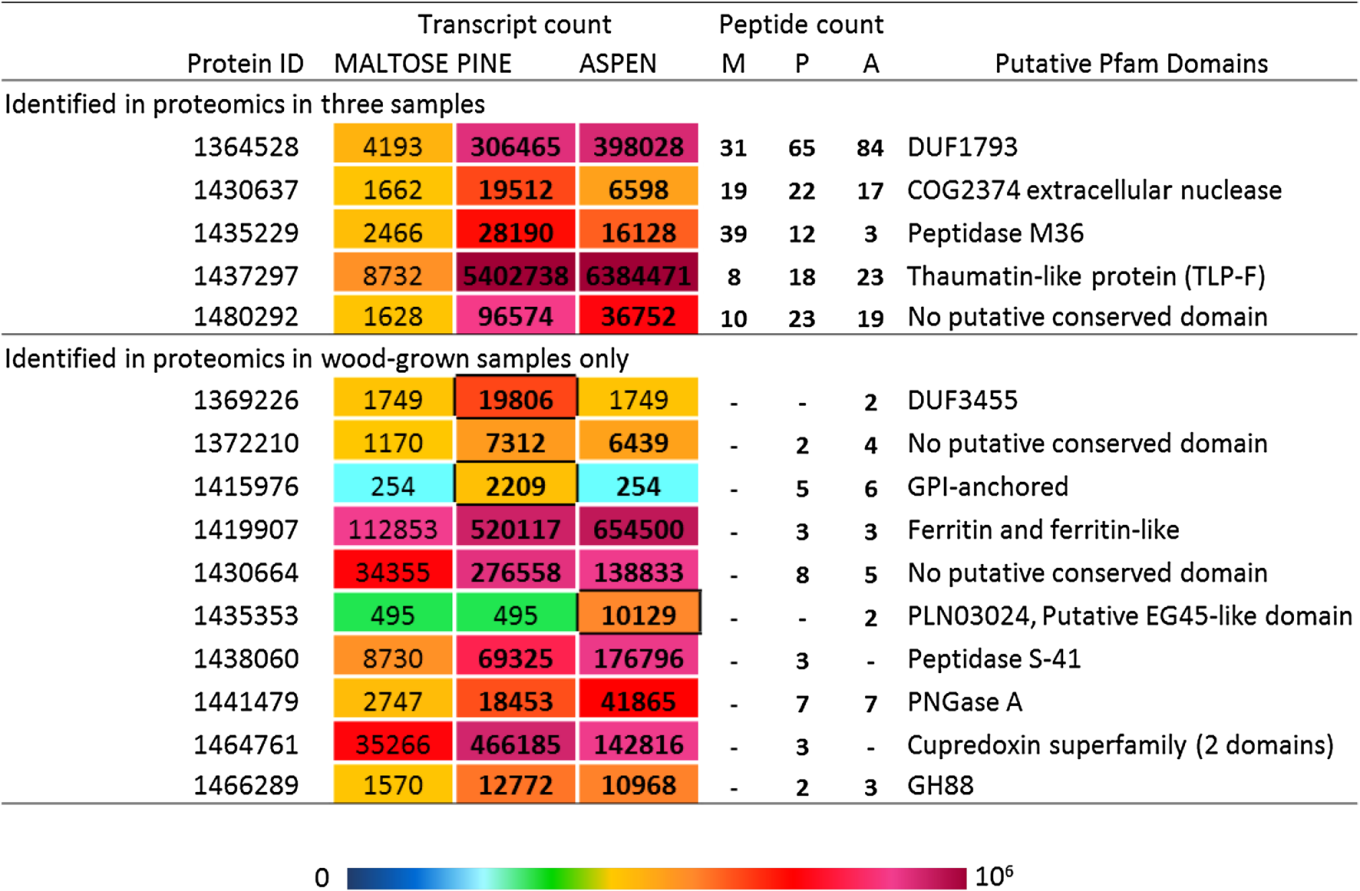
Heat maps and abundance of transcripts for proteins of unknown function produced by *P. coccineus* during growth. *M* PcoMaltose, *P* PcoPine, *A* PcoAspen

In addition, it is worth mentioning that both transcriptomic and proteomic analyses led to the identification of sequences of unknown or hypothetical functions. Transcriptomic data revealed an impressive number of gene models (2117) encoding proteins with unknown function (Additional file 2: Table S5). Among these, 455 and 414 transcript sequences were more than four times upregulated in pine and aspen cultivations, respectively. In particular, two sequences (1468135 and 1437297) were more than 500 times more abundant in both wood cultivations compared to the maltose reference. BlastP analysis revealed that sequence 1468135 is a putative hydrophobin (83 % identity with hydrophobin 2 from *Trametes versicolor*, EIW61059) and sequence 1437297 shared high homology with an osmotin thaumatin-like protein from *Trametes versicolor* (80 % identity, XP_008043489.1). Figure 5 summarizes transcripts encoding proteins with unknown function that encoded a signal peptide, differentially regulated in cultivations on wood, and for which proteins were identified in proteomic analyses. Notably, of the 48 proteins of unknown function identified in secretomes, 16 were uniquely identified in PcoPine and/or PcoAspen (Additional file 2: Table S6), and 15 corresponded to transcripts of unknown function that were enriched in at least one transcriptome from cultivation on wood (Additional file 2: Table S5).

### Characterization of wood hydrolysates produced by PcoPine and PcoAspen

**Fig. 6:**
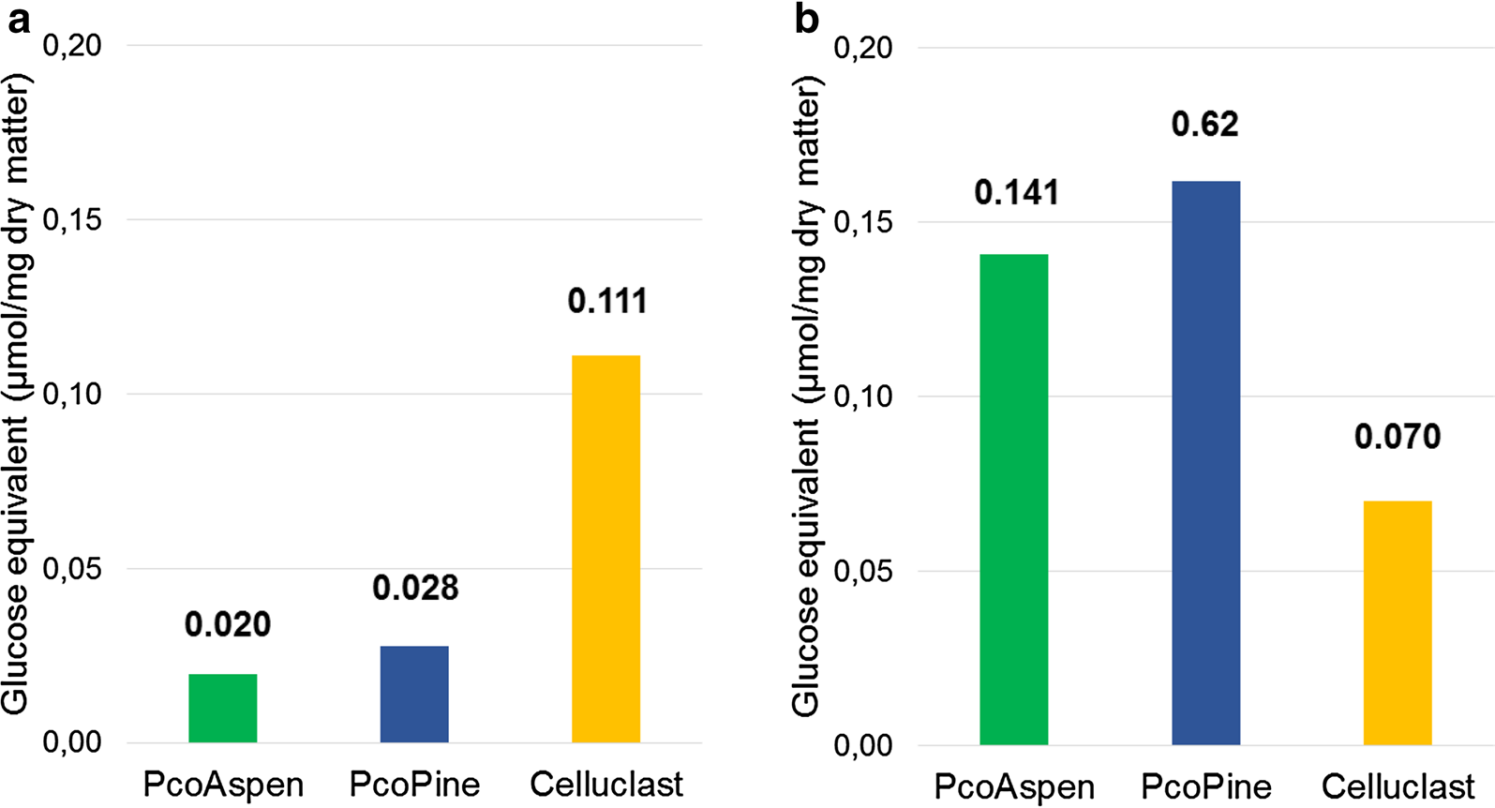
Saccharification of wood using PcoAspen and PcoPine secretomes. Soluble sugars as determined using the DNS assay following treatment of aspen (**a**) and pine (**b**). Celluclast-treated samples were included as a reference. Values were determined from three independent experiments

To compare the ability of PcoPine and PcoAspen to degrade aspen and pine wood, saccharification experiments were conducted. Wood fragments were incubated for 24 h with each secretome. The pine and aspen wood fragments used in this study contained 38.7 and 45.9 % glucose, 7. 6 and 16.9 % xylose, 9.0 and 1.8 % mannose, out of 65.1 and 69.9 % of total carbohydrates, respectively (Additional file 2: Table S7). DNS analyses revealed that PcoPine and PcoAspen were able to release comparable amounts of reducing sugars from pine and aspen, with PcoPine slightly but consistently more efficient over replicate experiments (Fig. 6). Surprisingly, both secretomes released significantly more reducing sugars from pine than from aspen. To check if this was due to the particular batches of wood that we utilized or to inherent properties of PcoPine and PcoAspen enzyme mixtures, we conducted a control experiment using Celluclast, a commercial enzyme mixture produced by *T. reesei*. As expected for a *T. reesei* cellulase cocktail, the Celluclast reference displayed the opposite pattern, with higher release of reducing sugars from aspen than pine (Fig. 6).

**Fig. 7:**
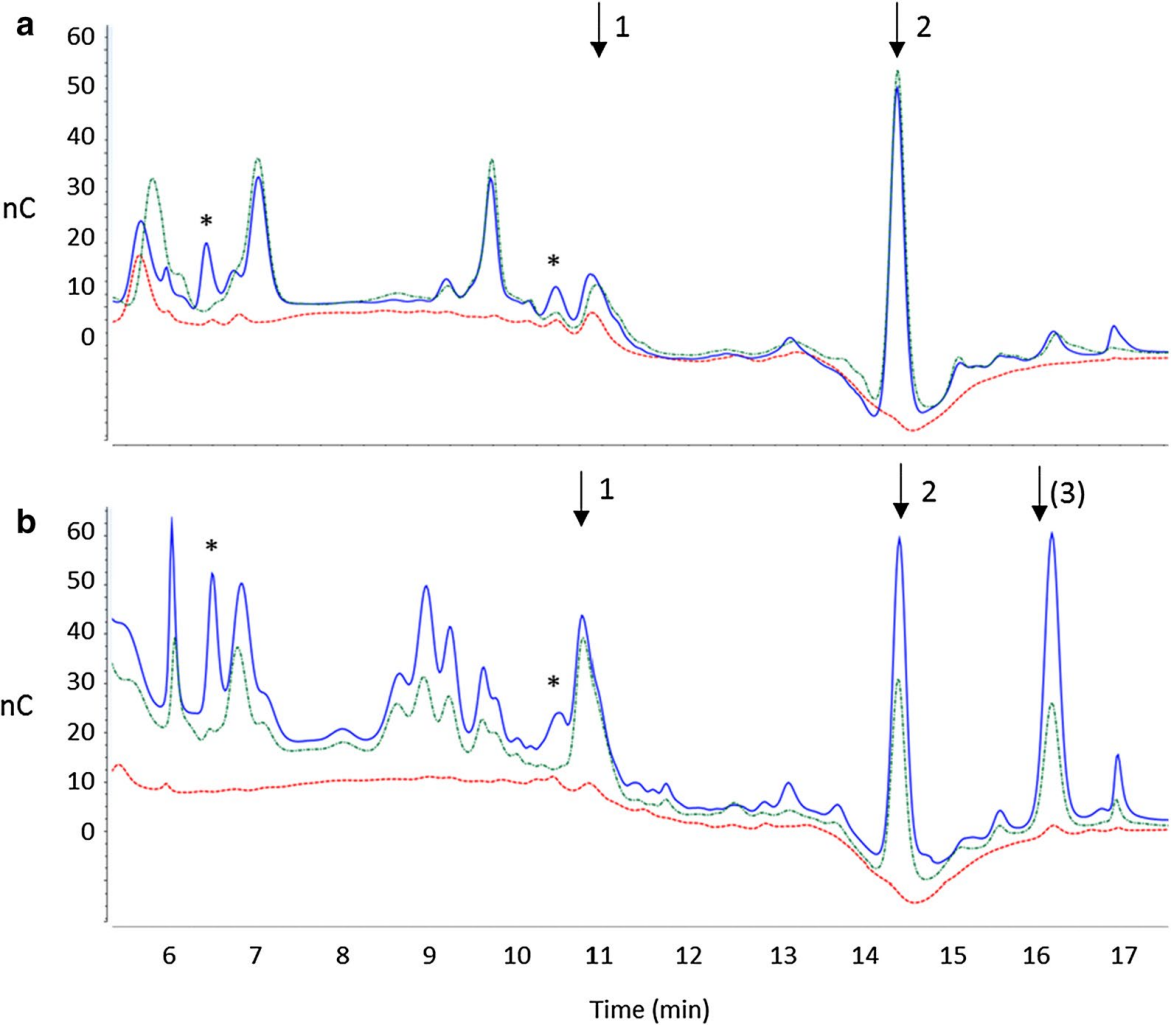
HPAEC profiles of soluble fractions after saccharification of aspen (**a**) and pine (**b**) using *P. coccineus* secretomes. The control sample (no enzymes) is shown in *red*, PcoPine-treated sample in *blue* and PcoAspen-treated sample in *green*. *Black arrows* indicate oxidized sugars: (*1*) gluconic acid was identified at 10.8 min, (*2*) cellobionic acid at 14.4 min and (*3*) possibly another oxidized sugar. *Black stars* indicate oligomers that were released upon incubation with PcoPine alone

Reaction supernatants were further analyzed using ionic chromatography to identify the released sugars (Fig. 7). Consistent with DNS analyses, the amounts of glucose released by PcoAspen and PcoPine from pine were significantly higher than that released from aspen (Additional file 3: Figure S3). In addition to the main peak corresponding to glucose (retention time 4.56 min), chromatograms of reaction products released from both aspen and pine also contained several smaller peaks with retention times between 5 and 17 min which likely corresponded to oligomers of cellulose or hemicellulose (Fig. 7). Of note, the soluble sugars released by PcoPine from both aspen (Fig. 7a) and pine (Fig. 7b) included at least two unique peaks (retention time 6.43 and 10.50 min, respectively), which may have contributed to the slightly higher DNS values measured using PcoPine.

In addition to neutral sugars, both PcoAspen and PcoPine also released significant quantities of oxidized sugars from both substrates. Oxidized sugar standards were used to confirm that at least gluconic acid and cellobionic acid (C1-oxidized sugars) were among the oxidized products. A third peak with a retention time of 16.20 min was detected only in reactions with pine, and could represent an additional oxidized sugar product. In a recent study, Isaksen et al. [28] have shown that an LPMO of family AA9 from *Neurospora crassa*, (NcLPMO9c), was able to cleave oligosaccharides as small as G4 and release oxidized G2, meaning that LPMOs can process oligomers released by cellulases. Given the detection of AA9 revealed by proteomic analysis, the presence of several oxidized sugars identified through HPAEC analysis might indicate a similar mechanism.

### Time-of-flight secondary ion mass spectrometry (ToF-SIMS) analysis of wood fiber residues

To gain further insight into the action of PcoPine and PcoAspen, residual pine and aspen wood were recovered following 24-h treatment with each secretome, and then analyzed by ToF-SIMS. ToF-SIMS is a mass spectrometry technique used to characterize the composition of sample surfaces, including relative occurrence of polysaccharides and lignin on wood fiber surfaces [29]. Previously, the sensitivity of ToF-SIMS (as low as ppb) was harnessed to investigate the action of both carbohydrate and lignin-active enzymes directly on wood fibers [30, 31], and so was used herein to detect changes in biomass compositions resulting from the action of PcoPine and PcoAspen. Notably, as a surface analysis technique, ToF-SIMS could provide new insight into the mode of action of each secretome, assuming that corresponding activities would first target fiber surfaces.

Wood samples treated with buffer alone were also used as negative controls. Peaks corresponding to lignin and polysaccharides were extracted from ToF-SIMS spectra, and intensities between wood treatments were compared using principal component analysis (PCA). Analysis of lignin specific peaks showed a composition of lignin in pine and aspen samples characteristic of softwood and hardwood samples, respectively. Spectra from pine mainly included peaks at 137 and 151 Da [characteristic of guaiacyl lignin (G-lignin)] and spectra from aspen included additional peaks at 167 and 181 Da [characteristic of syringyl lignin (S lignin)]. A large peak at 121 Da [characteristic of *p*-hydroxyphenyl lignin (H-lignin)] was also detected in aspen samples. However, this peak has also been ascribed to *p*-hydroxybenzoic acid, which is present in poplar species [32]. Accordingly, to minimize interferences from aromatic compounds not originating from lignin, the peak at 121 Da was discarded from subsequent PCA.

**Fig. 8:**
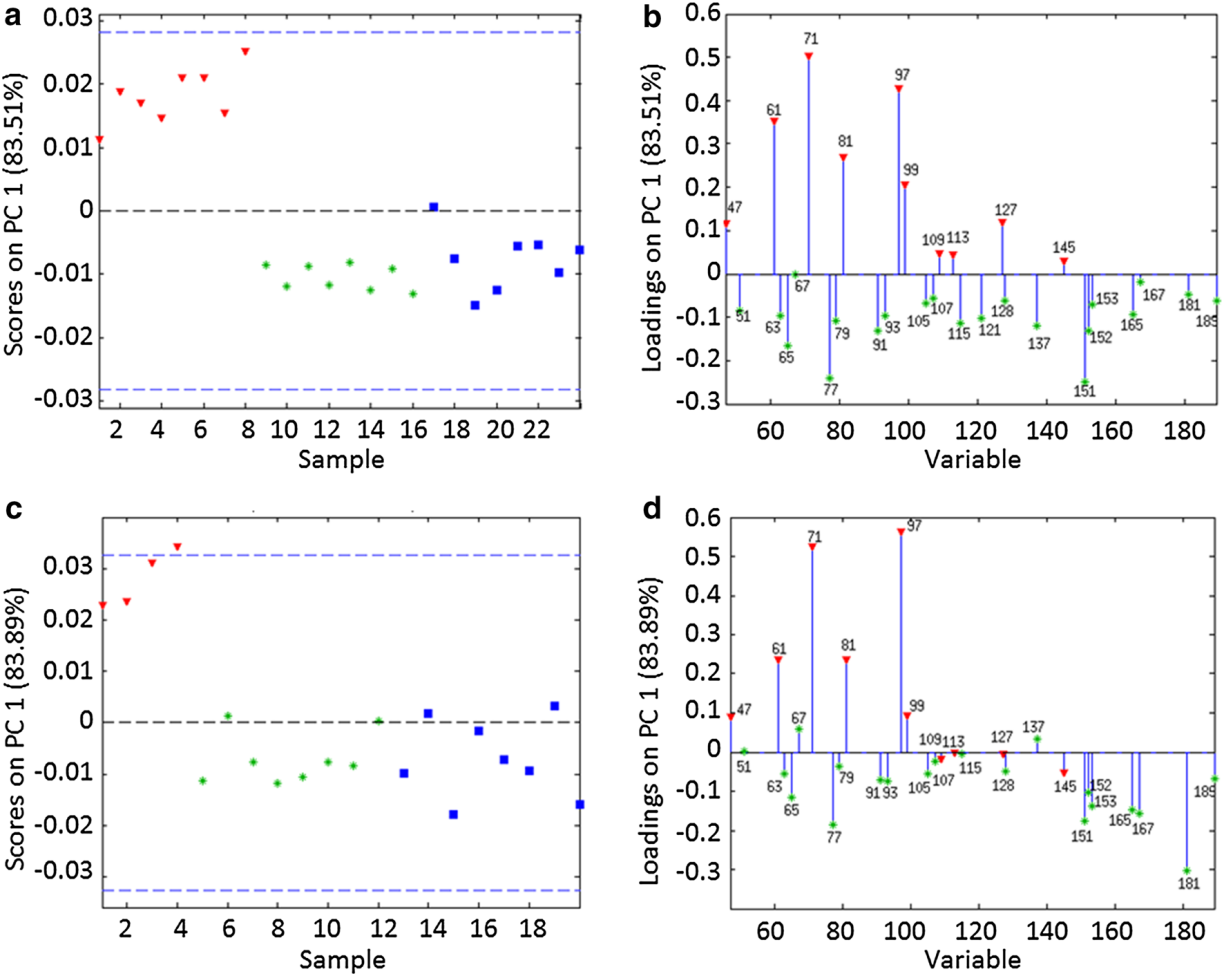
PCA analysis of ToF-SIMS spectra obtained from residual pine (**a**, **b**) and aspen (**c**, **d**) after enzymatic treatment. PCA scores (**a**, **c**) and loadings (**b**, **d**) show that the separation of treatment groups was explained by differences in lignin and polysaccharide ions. Score plots: *red triangles* control samples (no enzyme), *green stars* PcoAspen-treated samples, and *blue squares* PcoPine-treated samples. Loadings plots: *red triangles* polysaccharide peaks and *green* *stars* lignin peaks. Percent values denote the percent of total sample variance described by PC1. Four to eight spectra were acquired for each sample

PCA of spectra generated from wood treated with PcoAspen and PcoPine confirmed that the major difference relative to control samples was the depletion of polysaccharide peaks in the treated samples, indicating that the main modification of both pine and aspen by either secretome resulted from the enzymatic hydrolysis of plant polysaccharides (Fig. 8). Closer inspection of PCA loadings for aspen samples (Fig. 8d) revealed that the intensity of certain polysaccharide peaks did not differ between enzyme-treated and control samples, including peaks at 127 and 145 Da, previously attributed to cellulose [33]. By contrast, these peaks were depleted in enzyme-treated pine samples (Fig. 8b), consistent with more extensive degradation of cellulose as indicated by DNS and HPAEC analyses. This result would suggest that while both PcoPine and PcoAspen modified wood fiber surfaces, pine samples were more readily penetrated by both secretomes, leading to higher overall release of reducing sugars. PCA did not distinguish enzyme-treated and control samples based on lignin composition. This result suggests that lignin modifications by PcoPine and PcoAspen were minor in our experimental conditions, and would have had minimal impact on overall changes to polysaccharide composition.

## Discussion

The ability of the white-rot fungus *Pycnoporus coccineus* CIRM-BRFM310 to grow on both softwood and hardwood motivated the analysis of the enzyme combinations that could be used to improve processing of coniferous wood and hardwood feedstocks.

Our transcriptomic and secretomic analyses of *P.coccineus* in response to woody substrates identified the most highly expressed genes coding for secreted enzymes at early stages of fungal growth on wood. The distribution of CAZy families present in PcoAspen and PcoPine secretomes confirmed the typical white-rot behavior of *P. coccineus* [14]. Notably, many glycoside hydrolase families present in PcoAspen and PcoPine secretomes were also identified in the secretomes of other Agaricomycotina cultivated on the same aspen source [34]. In particular, enzymes belonging to families GH3 and GH5 were identified in the secretomes of all the Agaricomycotina compared in this earlier study. Similar to our findings, CBM1 was the most frequently represented carbohydrate-binding module, which was similarly fused to glycoside hydrolase families predicted to target cellulose and xylan (i.e. families GH5, GH6, GH10, GH11 and AA9) [34]. In our study, both PcoAspen and PcoPine comprised 12 CBM1-containing enzymes among which one GH6, three GH7s, and two AA2 peroxidases, representing typical activities required for both cellulose and lignin depolymerization. Family AA9 LPMOs are also produced by typical white-rot degraders [34] and have been shown to dramatically improve cellulase performance [35]. Characteristically then, transcriptomic analysis of *P. coccineus* following growth on aspen or pine confirmed the expression of 15 AA9 genes, in addition to the one AA9 and two AA9s for which corresponding proteins were detected in PcoPine and PcoAspen, respectively, confirming the potential role of LPMO in wood conversion.

Notably, the most upregulated transcript corresponded to a predicted H_2_O_2_-producing alcohol oxidase (1368318; CAZY family AA3_3). Alcohol oxidase enzymes are flavoproteins from the glucose–methanol–choline (GMC) oxidoreductases family [36]. Alcohol oxidases can use the methanol potentially produced during lignin attack (demethoxylation) to produce H_2_O_2_ and have so far been mainly associated to hydroxyl radical generation in Fenton reactions induced by brown-rot wood decay fungi [15, 37]. Genes coding for predicted alcohol oxidases have been found in the genome of white-rot fungi [34] and high levels of transcription for alcohol oxidases upon growth on wood have been highlighted in the white-rotter *P. chrysosporium* [15]. This suggests a major role for H_2_O_2_ producing enzymes which support both the action of peroxidases and the Fenton reaction, and will warrant further investigation.

*Pycnoporus coccineus* has previously been studied for its ability to secrete laccases (CAZy family AA1_1; 20). The monokaryotic strain CIRM-BRFM 310 used in this study was obtained in vitro from a basidiospore of the parental strain CIRM-BRFM 66 (IMB W006-2) which was first identified as a high producer of laccase [38]. After expert annotation of the AA1 CAZy family, we identified five gene models coding for predicted laccases (Additional file 2: Table S8). Our results show that three laccase genes are transcribed in CIRM-BRFM 310 during growth on maltose (1366139, 1452465 and 1477425). In our conditions, 1452465 shows constitutive transcription on pine and aspen and is found in the secretome of the fungus grown on these substrates. On the contrary, 1366139 and 1477425 are the most abundantly transcribed on maltose and are downregulated after 3-day growth on pine and aspen (Additional file 2: Table S8). These results show that, although two laccases are detected in the secretomes of the fungus grown on pine and aspen, low transcript levels are observed on these substrates, suggesting that laccases are rather downregulated during the early response of this fungus to woody biomass.

Although the overall profile of CAZymes obtained through transcriptome and secretome sequencing revealed similar sets of enzymes released after cultivating *P. coccineus* on aspen and pine, some notable differences were observed. In particular, transcripts encoding xylanases were more abundant following cultivation on aspen, and several distinct AA9 transcripts were enriched following growth on either aspen or pine. Nevertheless, these differences did not lead to detectable impact on saccharification results: release of total reducing sugars, profiles of neutral oligomers and oxidized sugars, and modifications of wood fiber surfaces. Perhaps more remarkable, both PcoPine and PcoAspen released more total sugars from pine preparations compared to aspen. This observation underscores the additional insight gained through characterizing both soluble and insoluble products from saccharification assays, and suggests that a core set of enzymes present in both PcoPine and PcoAspen penetrated pine particles more readily than aspen.

In general, wood fiber from hardwood species is more readily biologically transformed compared to coniferous softwood [39]. This has been attributed in part to higher lignin content, larger amounts of extractive components, and smaller pore size in softwood [40, 41]. Nevertheless, some white-rot fungi such as *D. squalens* [11], *P. carnosa* [6] and *P. gigantea* [10] have developed the ability to grow efficiently on softwood. Related studies have highlighted specific patterns in terms of gene content, gene expression and proteins secreted to tackle softwood substrates. For instance, pectin degradation has been previously correlated to efficient softwood decay; particularly through the solubilization of pectin-rich pit membranes which thereby promotes access to other lignocellulose components and penetration through adjacent tracheids [42]. In this context, it is interesting to note that the genomes of the conifer-adapted fungi *P. gigantea* and *Heterobasidion annosum* encode higher numbers of GH28 and CE8 pectinolytic enzymes compared to other Polyporales sequenced to date [10]. Higher expression of GH28 genes upon growth on softwood (especially pine) versus hardwood has been observed for *P. carnosa* [43]. Similar observations were made for the brown-rot fungus *P. placenta* [15], in which one GH28 polygalacturonase and one GH28 rhamnogalacturonase were enriched following growth on pine as opposed to aspen. In the current study, all seven genes predicted to encode GH28 polygalacturonases and the two genes predicted to encode CE8 pectin methylesterases were significantly enriched in *P. coccineus* CIRM-BRFM310 transcriptomes following growth on both aspen and pine (4–55 times more abundant than in the maltose condition). Involvement of cytochrome P450 monooxygenases in wood degradation and specifically in the transformation of softwood lignin and extractives has been previously discussed [23, 25]. For instance, 15 P450 monooxygenase genes were enriched upon growth on pine as compared to growth on aspen in the softwood-adapted brown-rot fungus *P. placenta* [15]. Similarly, the comparison of *P. chrysosporium* and *P. carnosa* genomes has revealed a particularly large number of P450 sequences in the softwood degrader *P. carnosa*. In this study, transcript levels of P450 sequences belonging to clans 64 and 534 were enriched upon growth on both aspen and pine. It is interesting to note that these clans were also among the most expanded clans in *P. carnosa* P450ome as compared to *P. chrysosporium* [6]. Preferential expression of MnPs over LiPs has also been correlated to growth on coniferous wood. Examples include *D. squalens,* whose genome encodes nine MnP and no LiP [44], and *P. carnosa* [6] in which seven MnP and only four LiP genes have been identified. Accordingly, of the four MnPs and four LiPs encoded by the *P. coccineus* genome (Additional file 2: Table S9), three MnP genes were significantly expressed upon growth on both aspen and pine, whereas transcripts corresponding to LiP genes were not identified. It is conceivable that Mn^3+^ chelates that mediate MnP activity can diffuse readily through the particularly dense G-lignin structures of plant cell walls, as suggested in Ref. [43]. However, this hypothesis will require further studies to compare the precise activity of MnP and LiPs on G and S lignin subunits.

In summary, the current study revealed that while *P. coccineus* growth on hardwood is common in nature, this fungus also contains the enzymatic arsenal appropriate for efficient conversion of softwood. Several parameters such as expression of pectinolytic enzymes, P450 monooxygenases and manganese peroxidases have all been noticed in earlier studies and might be critical to the ability of some fungi to degrade coniferous wood. Certainly, biochemical characterization of isolated enzymes and defined enzyme mixtures, along with additional post-genomic investigations over the Polyporales order, will help to clarify these correlations.

## Conclusion

This study reveals the surprising potential of the white-rot fungus *P. coccineus* for softwood degradation. The extensive analysis that was performed here combined genomic, transcriptomic and proteomic analyses with the assessment of enzyme activity on biomass, and revealed a full set of carbohydrate- and lignin-active enzymes produced upon cultivation on wood. The capability of *P. coccineus* for softwood deconstruction might be explained by several parameters such as peroxidase content and fiber penetration. Because of its ability, *P. coccineus* is a promising model to better understand the challenges of softwood biomass deconstruction and its use in biorefinery processes.

## Methods

### Fungal strain and enzymes

*Pycnoporus coccineus* CIRM-BRFM310 was obtained from the CIRM collection (Centre International de Ressources Microbiennes, https://www6.inra.fr/cirm_eng/Filamentous-Fungi) at the National Institute of Agricultural Research. Novozymes Celluclast 1.5L cocktail was purchased from Sigma-Aldrich (St-Louis, USA).

### *Pycnoporus coccineus* culture conditions and secretome preparation

*Pycnoporus coccineus* CIRM-BRFM 310 was grown on plates containing 15 g l^−1^ agar, YNB 1.7 g l^−1^, diammonium tartrate 1.84 g l^−1^ and either 20 g l^−1^ maltose or 15 g l^−1^ of non-pretreated and non-extracted wood powder. Plates were inoculated with a disk of a previous culture and incubated for 6 weeks at 30 °C. For RNA extractions from liquid cultures, inocula were prepared from 10-day-old non-agitated precultures as described in Ref. [45]. *P. coccineus* CIRM-BRFM 310 was grown for 3 days at 30 °C in a rotary shaker at 120 rpm in 250-ml Erlenmeyer flasks containing 100 ml of diammonium tartrate (1.84 g l^−1^); KH_2_PO_4_ (0.2 g l^−1^); CaCl_2_ (0.01 g l^−1^); MgSO_4_·7H_2_O (0.5 g l^−1^); Fe-SO4·7H_2_O (0.074 g l^−1^); ZnSO4·7H_2_O (0.077 g l^−1^); MnSO_4_·H_2_O (0.035 g l^−1^); CuSO_4_·5H_2_O (0.007 g l^−1^); yeast extract (0.5 g l^−1^); thiamin (0.002 g l^−1^); maltose (2.5 g l^−1^) in addition with ground and sifted pine wood fragments <2 mm (15 g l^−1^) or 1 mm Wiley-milled aspen (15 g l^−1^). Pine (*Pinus halepensis*) was obtained from Provence Forêt (Peyrolles en Provence, France) and consisted of air dried pine particles that contained all parts of the trunk including bark. Wiley-milled aspen (*Populusgrandidentata*) was kindly provided by Dan Cullen (Forest Product Laboratory, USDA, Madison, WI, USA). Both pine and aspen powders were raw, unextracted samples, and no pretreatment other than grinding was applied. For controls, the fungus was grown in the same medium with maltose (20 g l^−1^) as a carbon source. Each culture was performed in triplicate. Following the 3-day cultivation, culture supernatants containing secreted protein were harvested, filtered using a 0.2-µm pore-size polyethersulfone membrane (Vivaspin; Sartorius, Germany), diafiltered with 50 mM sodium acetate (pH 5.0) and concentrated using a Vivaspin polyethersulfone membrane with a 10-kDa cutoff (Sartorius). Concentrated samples were then stored at −20 °C until use. Mycelium and remaining substrate were immediately frozen in liquid nitrogen and stored at −80 °C before RNA extraction.

### RNA-seq and subsequent data analysis

Total RNA was extracted from 100 mg tissue ground with FastPrep Lysis Matrix A (MP Biomedicals) in 1 ml TRIZOL (Ambion). Nucleic acids were precipitated with isopropanol, resuspended in water and treated with RNase-free DNase I (QIAGEN). Total RNA was precipitated with LiCl and resuspended in DEPC-treated water. RNA quantity and quality were determined using the Experion RNA StdSens kit (QIAGEN). Double-stranded cDNAs were synthesized from Poly A RNA and fragmented (200–300 bp) before construction of the sequencing libraries (Kapa Library Amplification Kit; Kapa Biosystems). Sequencing was done on the Illumina HighSeq-2500 JGI platform generating paired end reads of 150 bp each. Raw fastq file reads were filtered and trimmed using the JGI QC pipeline. The reads obtained from each replicate; read counts and normalized read counts are available on GEO [46] under accession number GSE74234. QCed reads from each library were aligned to the reference genome *Pycnoporus cocci*neus BRFM 310 v1.0 using TopHat [47] with only unique mapping allowed. Read counts were determined by HTSeq (Galaxy Tool Version 0.3.2). Correlation between biological replicates was evaluated using Pearson’s correlation (Additional file 2: Table S10, Table S11). Differential gene expression was analyzed on R using DESeq2 package (version 1.5) [48]. Gene models for which the mean raw read counts were inferior to five were considered as not transcribed and their read counts were changed to zero. Read counts were normalized using ddsNorm from the DESeq Bioconductor package. This normalization method is based on the hypothesis that most genes are not differentially expressed and should have similar read counts across samples [49]. DESeq2 was used to determine differential expression between pairs of conditions. The parameters used to call a gene differentially expressed between conditions were *p* value Bonferroni <0.05 and p value Benjamini–Hochberg <0.05.

### Quantitative PCR

Reverse transcription was carried out using the iScript Reverse Transcription Supermix following the instructions of the manufacturer (Bio-Rad Laboratories, Marnes la Coquette, France). The primers for qPCR were determined using Primer3Plus (http://www.bioinformatics.nl/cgi-bin/primer3plus/primer3plus.cgi) and synthesized by Eurofins (Nantes, France) (Additional file 2: Table S12). The cDNA was diluted 50 times, and 2 µl was used per PCR, with 4 µl of Sso advanced SYBR Green Supermix (Bio-Rad Laboratories) and 0.3 µl each of 10 µM forward and reverse primers. Volume was adjusted to 20 µl with water. Thermocycling was carried out with one cycle at 95 °C for 30 s, followed by 40 cycles of 95 °C for 5 s and 57 °C for 5 s.

### Characterization of secretomes using LC–MS/MS

LC–MS/MS analysis of *P. coccineus* secretomes, PcoAspen, PcoPine and PcoMaltose, was performed as described in Ref. [50]. Briefly, short SDS-PAGE runs (pre-casted Bis–Tris Mini Gels, Invitrogen, France) were performed, allowing 10 µg of proteins diafiltered from secretomes to migrate on 0.5 cm length. Each one-dimensional electrophoresis lane was cut into two slices of gel and protein identification was performed using PAPPSO “Plate-forme d’Analyse Protéomique de Paris Sud-Ouest” platform facilities. In-gel digestion was carried out according to a standard trypsinolysis protocol. Online analysis of peptides was performed with a Q-exactive mass spectrometer (Thermo Fisher Scientific, USA), using a nanoelectrospray ion source. Protein identification was performed by querying MS/MS data against the *Pycnoporus coccineus* genome (http://genome.jgi.doe.org/Pycco1/Pycco1.home.html), together with an in-house contaminant database, using the X!Tandem software (X!Tandem Cyclone, Jouy en Josas, France). All peptides matched with an *E* value lower than 0.05 were parsed with X!Tandem pipeline software. Proteins identified with at least two unique peptides and a log (*E* value) lower than −2.6 were validated.

### Preparation of wood powders for activity assays

For activity measurement, the two wood samples (ground and sifted pine wood fragments and Wiley-milled aspen) were further ground separately using a cryomill to particles of roughly 100 μm (milled powders passed through a US mesh size 100 sieve, 0.150 mm diameter). Extraction on wood powders was done following the ASTM standard D1105. Briefly, samples were Soxhlet extracted in a mixture of ethanol:toluene (1:0.427 v/v) for 4 h, followed by ethanol for 8 h. The extractive-free samples were then oven-dried at 50 °C for one night and stored at room temperature until use. No other pretreatment was applied.

### Saccharification assay and soluble sugar analysis

The extractive-free wood powders described above were suspended [4 % (w/v)] in 50 mM acetate buffer (pH 5.0), and 100 µl samples were transferred to each well in 96-well microplates. Reactions were initiated by adding 6 µg of a fungal secretome or 6 µg of Celluclast to reach a final protein concentration of 30 mg ml^−1^. The final reaction volume was adjusted to 200 µl using 50 mM acetate buffer (pH5) to reach 2 % final suspension. Reaction mixtures were incubated at 40 °C for 24 h with intermittent shaking (5 min off, 20 s on cycles) at 1000 rpm in a Thermomixer incubator (Eppendorf Canada, Mississauga, Ontario). Reaction mixtures were then processed using a filtration plate (Millipore MultiScreen Barex/TiO_2_ plates) as previously described [31]. The dinitrosalicylic (DNS) assay for reducing sugar analysis was carried out with reaction supernatants using a Tecan liquid handler (Freedom Evo100, Tecan, Männedorf, Switzerland) and the automatized method described previously [51]. Reducing sugars were expressed in μmol of sugars released per mg of dry matter.

### HPAEC analysis of reducing sugars

After saccharification, soluble sugar profiles were analyzed using high-performance anion-exchange chromatography (HPAEC) coupled with pulsed amperometric detection (PAD) (ICS 3000; Dionex, Sunnyvale, USA) equipped with a carboPac PA-1 analytical column (250 × 2 mm) and guard column. Samples and standards were injected into the HPAEC system and elution was carried out using a multi-step gradient following the protocol described in Ref. [2]. Briefly, the eluents were 0.1 M NaOH (eluent A) and 1 M NaOAc in 0.1 M NaOH (eluent B). Elution was performed at a constant flow rate of 0.25 ml/min at room temperature, using a linear gradient of 0–10 % eluent B over 10 min, 10–30 % eluent B over 25 min, and an exponential gradient of 30–100 % eluent B in 5 min. The initial condition (100 % eluent A) was then restored in 1 min and maintained for 9 min to recondition the column.

### Time-of-flight secondary ion mass spectrometry

Time-of-flight secondary ion mass spectrometry measurements were made on extractive-free wood powders prepared as described above with a ToF-SIMS IV instrument (Ion-Tof Gmbh, Münster, Germany) equipped with a bismuth liquid metal ion source and reflectron-type analyzer with multichannel detector. Eight to ten spectra were acquired to ensure adequate sampling, using 50 keV Bi_3_^2+^ primary ions (~0.3 pA pulsed current) incident at 45°, operated on 100 μs cycle time with high-current bunched conditions. Spectra were acquired for 60–120 s using 128 × 128 pixel random raster pattern covering a 500 × 500 μm^2^ area. Ion doses were kept below 1 × 10^12^ ions/cm^2^ to limit sample damage. The pressure during analysis was maintained between 1 × 10^−8^ and 1 × 10^−7^ mbar. Low-energy electron flooding (20 eV) was used to reduce sample charging. Positive ion spectra were calibrated to C^+^, CH^+^, CH2^+^, CH_3_^+^, C_2_H_3_^+^ and C_3_H_5_^+^ ions using SurfaceLab v.6.1 software. Mass resolution (M/ΔM) varied depending on sample roughness and all ToF-SIMS data were binned to 1 Da before calculating peak ratios or applying statistical analysis.

### Principal component analysis

Principal component analysis (PCA) was performed using Matlab software v.7.14.0.739 R2012a (The Mathworks, Inc.) with PLS Toolbox v.6.7.1 (Eigenvector Research Inc.). Individual ToF-SIMS spectra from replicate locations on the wood powder residues were preprocessed as follows: (i) the peak intensities were normalized to the total ion intensity of the peak list to eliminate the influence of overall intensity changes that might arise from topography or variations in instrumental setup; (ii) data at each mass were mean centered so that all principal components (PCs) described variations from the data set mean.

## Additional files


10.1186/s13068-015-0407-8 Supplementary Materials and Methods.

10.1186/s13068-015-0407-8 Comparison of the CAZy repertoires in the genome of *Pycnoporus coccineus* CIRM-BRFM310-v1 with genomes of *Trametes cinnabarina* CIRM-BRFM137, *Trametes versicolor* FP-101664_SS1 and *Phanerochaete chrysosporium* RP-78 v2.1. **Table S2.** CAZy transcripts identified in transcriptome analyses. **Table S3.** CAZymes identified in proteome analyses. **Table S4.** Cytochrome P450 monooxygenases transcripts identified in transcriptome analyses. **Table S5.** Transcripts encoding sequences of unknown function identified in transcriptome analyses. **Table S6.** Proteins of unknown function identified in proteome analyses. **Table S7.** Carbohydrate composition of pine and aspen substrates used in the study. **Table S8.** Expert annotation and transcription profile of AA1s from *Pycnoporus coccineus* CIRM-BRFM 310. **Table S9.** Expert annotation and transcription profile of AA2s from *Pycnoporus coccineus* CIRM-BRFM 310. **Table S10.** Characteristics of RNA libraries used in this study. **Table S11.** Heat map of the Pearson correlations between RNASeq read counts obtained from biological triplicates of the fungus grown on maltose (M), pine (Pin) and aspen (Asp). **Table S12.** Primers used for qPCR in this study.

10.1186/s13068-015-0407-8 Transcripts abundancies determination of a set of genes by qPCR and RNAseq. Full bars indicate qPCR results, striped bars indicate RNASeq results. Expression levels on pine are shown in blue, expression levels on aspen are shown in green. Results are expressed as log2 of enrichment as compared to maltose control. UNK: 1437297, 447: 1362447, 558:1362558, 918:1297918, AA9: 1466495, AA2: 1468611, GH10: 1395316, GH28: 688728. **Figure S2.** Electrophoresis profile of *P. coccineus* secretomes. MW: Molecular weight in kDa (lane 1), secreted proteins after 3 day cultivations on maltose (lane 2), pine (lane 3), and aspen (lane 4). **Figure S3.** HPAEC profiles of soluble fractions after saccharification of aspen (A) and pine (B) using *P. coccineus* secretomes highlighting glucose release (star). **Figure S4.** Homology models for the molecular structures of class I and II heme peroxidases from the *P. coccineus* CIRM-BRFM 310 genome. Ligninolytic peroxidases, including models for 1431101 (A), 1403742 (B), 779035 (C) and 859168 (D) - harboring an exposed tryptophan potentially involved in oxidation of high redox-potential substrates, MnP-short models 1468611(E), 1436321 (F), 1464049 (G) and 1369658 (H) - harboring a putative Mn^2+^ oxidation site (formed by two glutamates and one aspartate), two VP models - 1468768 (I) and 1469331 (J) - harboring the two catalytic sites described for LiPs and MnPs, one atypical VP - 1438352 (K) - containing an atypical Mn^2+^ oxidation site formed by two aspartates and one glutamate; and CCP 1449695 (L).

10.1186/s13068-015-0407-8 Proteomics complementary information. For each condition (Maltose, Pine and Aspen), data are given regarding identified proteins (e.g. number of identified spectra, number of unique peptides, number of specific peptides) and data regarding each identified peptide (e.g. sequence, e value, charge).

## Abbreviations

AA: auxiliary activity
CAZyme: carbohydrate-active enzyme
CBM: carbohydrate-binding module
CE: carbohydrate esterase
DNS: dinitrosalicylic acid
GH: glycoside hydrolase
G lignin: guaiacyl lignin
GMC: glucose methanol choline
GT: glycosyltransferase
H lignin: *p*-hydroxyphenyl lignin
HPAEC-PAD: high-pressure anion-exchange chromatography with pulsed amperometric detection
LiP: lignin peroxidase
LPMO: lytic polysaccharide monooxygenase
MnP: manganese peroxidase
PC: principal component
PCA: principal component analysis
PL: polysaccharide lyase
S lignin: syringyl lignin
ToF-SIMS: time-of-flight secondary ion mass spectrometry
