# Supplementary material for: Enhanced degradation of softwood versus hardwood by the white-rot fungus Pycnoporus coccineus
Source: Biotechnol Biofuels. 2015 Dec 18;8:216. doi: 10.1186/s13068-015-0407-8 (PMC4683735; doi:10.1186/s13068-015-0407-8)
Supplement: Supplementary file 1 — 10.1186/s13068-015-0407-8 Supplementary Materials and Methods. [file 13068_2015_407_MOESM1_ESM.docx]

**Additional File 1 : Supplementary Materials and Methods**

**Functional annotation of CAZymes**

Annotation of the set of carbohydrate-active enzymes was performed by comparing the predicted proteins to those in the CAZy database (Carbohydrate-Active enzymes Database; [www.cazy.org](http://www.cazy.org); [1]. As CAZy annotation is based on the recognition of individual catalytic modules or Carbohydrate Binding Modules (CBMs), it is possible to assess whether a gene model encoding a CAZyme covers the full length of the identified modules. Only the gene models with more than 80% overlap with a reference sequence were validated as belonging to one or more of the CAZy families, depending on their modular composition.

**Functional annotation of AA2s**

A preliminary screening of the automatically-annotated genome was performed using the Search option (“peroxidase” as search term) at the JGI web-site, and 145 gene models were initially identified in *P. coccineus* CIRM-BRFM 310. Then, a sequence-by-sequence exhaustive analysis revealed that only sixteen of the above gene models encode heme peroxidases. Class II ligninolytic peroxidases could be annotated as LiP, MnP and VP on the basis of the presence or absence of only a few amino acid residues at the substrate oxidation sites [2, 3] after homology modeling. In this respect: i) LiPs are characterized by harboring an exposed catalytic tryptophan homologous to Trp171 in *Phanerochaete chrysosporium* LiP-H8 and Trp164 of *Pleurotus eryngii* VPL; ii) MnPs are characterized by containing a Mn(II)-oxidation site near the internal propionate of heme formed by three acidic residues homologous to *P. chrysosporium* MnP1 Glu35, Glu39 and Asp179, and *P. eryngii* VPL Glu36, Glu40 and Asp175; and iii) VPs are characterized by presenting both the catalytic tryptophan and the Mn(II) oxidation site of LiPs and MnPs, respectively (atypical VPs contain an atypical Mn-oxidation site formed by one glutamate and two aspartate residues). Regarding MnPs, these peroxidases were annotated as members of the subfamily of short MnPs containing a short C-terminal tail like the exhaustively characterized short MnPs from *Pleurotus ostreatus* and *Ceriporiopsis subvermispora* [4, 5].

A representation of the homology models obtained for the enzymes identified, including key amino acid residues putatively involved in catalysis, is presented in Supplementary Figure 4, Additional File 3. LiP, MnP and VP models were obtained using the *Phanerochaete chrysosporium* LiPH8 (PDB entries 1B80 and 1B82), *Pleurotus ostreatus* MnP4 (PDB entry 4BM1) and VP1 (PDB entries 4BLK and 4BLN), and *Pleurotus eryngii* VPL2 (PDB entries 2BOQ, 4FCS and 3FMU) crystal structures as templates. Protein IDs and transcription profiles of the annotated LiP, MnP and VP are shown in Supplementary Table 12, Additional File 2.

**References**

1. Lombard V, Golaconda Ramulu H, Drula E, Coutinho PM, Henrissat B. The Carbohydrate-active enzymes database (CAZy) in 2013. Nucleic Acids Res. 2014; 42:D490-D495.
2. Ruiz-Dueñas FJ, Morales M, García E, Miki Y, Martínez MJ, Martínez AT. Substrate oxidation sites in versatile peroxidase and other basidiomycete peroxidases. J Exp Bot. 2009; 60:441-52.
3. Ruiz-Dueñas FJ, Lundell T, Floudas D, Nagy LG, Barrasa JM, Hibbett DS, Martínez AT. Lignin-degrading peroxidases in Polyporales: An evolutionary survey based on ten sequenced genomes. Mycologia. 2013; 105:1428-44.
4. Fernández-Fueyo E, Ruiz-Dueñas FJ, Martínez MJ, Romero A, Hammel KE, Medrano FJ, Martínez AT. Ligninolytic peroxidase genes in the oyster mushroom genome: Heterologous expression, molecular structure, catalytic and stability properties and lignin-degrading ability. Biotechnol Biofuels. 2014; 7:2.
5. Fernández-Fueyo E, Acebes S, Ruiz-Dueñas FJ, Martínez MJ, Romero A, Medrano FJ, Guallar V, Martínez AT. Structural implications of the C-terminal tail in the catalytic and stability properties of manganese peroxidases from ligninolytic fungi. Acta Crystallogr D Biol Crystallogr 2014; 70:3253-65.
