# Supplementary material for: Enhanced degradation of softwood versus hardwood by the white-rot fungus Pycnoporus coccineus
Source: Biotechnol Biofuels. 2015 Dec 18;8:216. doi: 10.1186/s13068-015-0407-8 (PMC4683735; doi:10.1186/s13068-015-0407-8)
Supplement: Supplementary file 2 — 10.1186/s13068-015-0407-8 Comparison of the CAZy repertoires in the genome of Pycnoporus coccineus CIRM-BRFM310-v1 with genomes of Trametes cinnabarina CIRM-BRFM137, Trametes versicolor FP-101664_SS1 and Phanerochaete chrysosporium RP-78 v2.1. Table S2. CAZy transcripts identified in transcriptome analyses. Table S3. CAZymes identified in proteome analyses. Table S4. Cytochrome P450 monooxygenases transcripts identified in transcriptome analyses. Table S5. Transcripts encoding sequences of unknown function identified in transcriptome analyses. Table S6. Proteins of unknown function identified in proteome analyses. Table S7. Carbohydrate composition of pine and aspen substrates used in the study. Table S8. Expert annotation and transcription profile of AA1s from Pycnoporus coccineus CIRM-BRFM 310. Table S9. Expert annotation and transcription profile of AA2s from Pycnoporus coccineus CIRM-BRFM 310. Table S10. Characteristics of RNA libraries used in this study. Table S11. Heat map of the Pearson correlations between RNASeq read counts obtained from biological triplicates of the fungus grown on maltose (M), pine (Pin) and aspen (Asp). Table S12. Primers used for qPCR in this study. [file 13068_2015_407_MOESM2_ESM.docx]

**Additional File 2: Supplementary Tables 1 to 12**

**Supplementary Table S1**: Comparison of the CAZy repertoires in the genome of *Pycnoporus coccineus* CIRM-BRFM310-v1 with genomes of *Trametes cinnabarina* CIRM-BRFM137, *Trametes versicolor* FP-101664_SS1 and *Phanerochaete chrysosporium* RP-78 v2.1

| CAZy family |  | *Trametes cinnabarina* BRFM137 | *Pycnoporus coccineus* BRFM310_v1 | *Trametes versicolor* FP-101664_SS1 | *Phanerochaete chrysosporium* RP-78_v2.1 |
| --- | --- | --- | --- | --- | --- |
| GH1 | PCW ^(a)^ | 2 | 2 | 2 | 2 |
| GH2 | PCW | 3 | 2 | 5 | 2 |
| GH3 | PCW | 8 | 8 | 13 | 10 |
| GH5 | PCW | 20 | 20 | 22 | 19 |
| GH6 | PCW | 1 | 1 | 1 | 1 |
| GH7 | PCW | 3 | 3 | 4 | 8 |
| GH9 | PCW | 1 | 1 | 1 | 1 |
| GH10 | PCW | 6 | 6 | 6 | 6 |
| GH11 | PCW | 0 | 0 | 0 | 1 |
| GH12 | PCW | 3 | 3 | 5 | 2 |
| GH13 |  | 7 | 7 | 7 | 8 |
| GH15 |  | 2 | 2 | 4 | 2 |
| GH16 |  | 29 | 27 | 31 | 22 |
| GH17 |  | 2 | 2 | 3 | 2 |
| GH18 |  | 16 | 19 | 20 | 11 |
| GH20 |  | 5 | 5 | 6 | 4 |
| GH23 |  | 1 | 1 | 1 | 1 |
| GH25 |  | 1 | 1 | 1 | 1 |
| GH27 | PCW | 2 | 4 | 4 | 3 |
| GH28 | PCW | 7 | 7 | 11 | 5 |
| GH30 | PCW | 3 | 3 | 4 | 2 |
| GH31 | PCW | 5 | 6 | 5 | 6 |
| GH32 |  | 1 | 1 | 3 | 0 |
| GH35 | PCW | 2 | 2 | 2 | 3 |
| GH37 |  | 2 | 2 | 2 | 2 |
| GH38 |  | 1 | 1 | 1 | 1 |
| GH43 | PCW | 3 | 3 | 3 | 4 |
| GH45 | PCW | 2 | 3 | 2 | 2 |
| GH47 |  | 5 | 5 | 5 | 5 |
| GH51 | PCW | 2 | 2 | 2 | 2 |
| GH53 | PCW | 0 | 1 | 1 | 1 |
| GH55 |  | 2 | 2 | 2 | 2 |
| GH63 |  | 1 | 1 | 1 | 1 |
| GH71 |  | 5 | 4 | 5 | 3 |
| GH72 |  | 1 | 1 | 1 | 1 |
| GH74 | PCW | 1 | 1 | 1 | 4 |
| GH76 |  | 2 | 1 | 0 | 0 |
| GH78 | PCW | 2 | 2 | 3 | 1 |
| GH79 |  | 12 | 5 | 11 | 8 |
| GH85 |  | 1 | 1 | 1 | 1 |
| GH88 | PCW | 2 | 1 | 1 | 1 |
| GH89 |  | 1 | 1 | 1 | 2 |
| GH92 |  | 5 | 5 | 4 | 4 |
| GH95 |  | 1 | 1 | 1 | 1 |
| GH105 | PCW | 0 | 0 | 1 | 0 |
| GH115 | PCW | 2 | 2 | 2 | 1 |
| GH125 |  | 1 | 1 | 1 | 1 |
| GH128 |  | 4 | 4 | 4 | 5 |
| GH131 | PCW | 3 | 3 | 3 | 3 |
| GH133 |  | 1 | 1 | 1 | 1 |
| GT1 |  | 18 | 24 | 21 | 9 |
| GT2 |  | 13 | 13 | 14 | 13 |
| GT3 |  | 1 | 1 | 2 | 1 |
| GT4 |  | 4 | 4 | 4 | 4 |
| GT5 |  | 1 | 1 | 1 | 1 |
| GT8 |  | 5 | 6 | 5 | 9 |
| GT15 |  | 3 | 3 | 5 | 2 |
| GT17 |  | 1 | 1 | 1 | 1 |
| GT20 |  | 3 | 3 | 3 | 3 |
| GT21 |  | 1 | 1 | 1 | 1 |
| GT22 |  | 4 | 4 | 4 | 4 |
| GT24 |  | 1 | 1 | 1 | 1 |
| GT31 |  | 1 | 1 | 1 | 1 |
| GT32 |  | 1 | 1 | 1 | 1 |
| GT33 |  | 1 | 1 | 1 | 1 |
| GT35 |  | 1 | 1 | 1 | 1 |
| GT39 |  | 3 | 3 | 3 | 3 |
| GT41 |  | 1 | 0 | 1 | 1 |
| GT48 |  | 2 | 2 | 2 | 2 |
| GT49 |  | 1 | 1 | 1 | 1 |
| GT50 |  | 1 | 1 | 1 | 1 |
| GT57 |  | 2 | 2 | 2 | 2 |
| GT58 |  | 1 | 1 | 1 | 1 |
| GT59 |  | 1 | 1 | 1 | 1 |
| GT66 |  | 1 | 1 | 1 | 1 |
| GT69 |  | 3 | 2 | 2 | 2 |
| GT76 |  | 1 | 1 | 1 | 1 |
| GT90 |  | 1 | 1 | 1 | 1 |
| PL4 | PCW | 1 | 1 | 1 | 0 |
| PL8 | PCW | 2 | 2 | 2 | 1 |
| PL9 | PCW | 0 | 0 | 0 | 0 |
| PL14 | PCW | 5 | 5 | 6 | 4 |
| CE1 | PCW | 3 | 3 | 3 | 4 |
| CE4 | PCW | 3 | 2 | 3 | 4 |
| CE8 | PCW | 2 | 2 | 2 | 2 |
| CE9 |  | 2 | 1 | 1 | 1 |
| CE15 | PCW | 2 | 2 | 2 | 2 |
| CE16 | PCW | 6 | 9 | 8 | 7 |
| CBM1 | PCW | 20 | 22 | 23 | 36 |
| CBM5 |  | 5 | 6 | 6 | 4 |
| CBM12 |  | 1 | 1 | 1 | 1 |
| CBM13 | PCW | 5 | 6 | 8 | 8 |
| CBM18 |  | 1 | 1 | 1 | 1 |
| CBM20 |  | 1 | 2 | 4 | 2 |
| CBM21 | PCW | 2 | 2 | 2 | 2 |
| CBM35 | PCW | 1 | 1 | 1 | 1 |
| CBM43 |  | 1 | 1 | 1 | 1 |
| CBM48 |  | 2 | 3 | 3 | 3 |
| CBM50 |  | 7 | 5 | 1 | 12 |
| AA1 |  | 7 | 7 | 10 | 5 |
| AA2 |  | 12 | 12 | 26 | 16 |
| AA3 |  | 25 | 26 | 23 | 39 |
| AA5 |  | 7 | 7 | 9 | 7 |
| AA6 |  | 1 | 1 | 1 | 4 |
| AA8 |  | 2 | 2 | 2 | 2 |
| AA9 | PCW | 17 | 16 | 18 | 16 |
| AA12 | PCW | 0 | 0 | 0 | 5 |

AA: Auxiliary Activity, CBM: Carbohydrate Binding Module, CE: Carbohydrate Esterase, GH: Glycoside Hydrolase, GT: Glycoside Transferase, PL: Polysaccharide Lyase. (a) : The families containing genes potentially active on plant cell walls are indicated (PCW). The gene families targeting cellulose are highlighted in pink.

**Supplementary Table S2**: CAZy transcripts identified in transcriptome analyses

| ProteinID | Corresponding CAZy Family | Base Mean_ Maltose | log2Fold Change_  PIN | padj_PIN | padj_Bonf_  PIN | log2Fold Change_ ASP | padj_ASP | padj_Bonf_ ASP | P/A |
| --- | --- | --- | --- | --- | --- | --- | --- | --- | --- |
| 1366139 | AA1_1 | 38309 | -1.92 | 5,75E-07 | 1,51E-03 | -2.55 | 2,19E-11 | 2,64E-08 | 0.63 |
| 1452465 | AA1_1 | 303 | 0.00 | NA | NA | 0.00 | NA | NA | 0.00 |
| 1477425 | AA1_1 | 2419 | -2.14 | 4,45E-17 | 3,88E-14 | -2.70 | 1,59E-26 | 6,43E-24 | 0.56 |
| 1425237 | AA1_2 | 1644 | 0.00 | NA | NA | 0.00 | NA | NA | 0.00 |
| 1436321 | AA2 | 8113 | 6.52 | 1,38E-31 | 3,94E-29 | 6.25 | 3,50E-29 | 1,22E-26 | 0.26 |
| 1438352 | AA2 | 1162 | -3.46 | 2,12E-26 | 9,13E-24 | -4.10 | 1,98E-36 | 4,64E-34 | 0.64 |
| 1464049 | AA2 | 2996 | 0.00 | NA | NA | 6.91 | 7,16E-74 | 2,65E-72 | -6.91 |
| 1468611 | AA2 | 24345 | 9.08 | 3,82E-106 | 1,53E-105 | 9.71 | 1,67E-121 | 8,33E-121 | -0.62 |
| 68108 | AA3_2 | 1789 | -1.85 | 2,95E-20 | 1,95E-17 | -1.93 | 3,88E-22 | 2,03E-19 | 0.09 |
| 1362859 | AA3_2 | 400 | 2.71 | 3,70E-18 | 2,95E-15 | 1.62 | 4,85E-07 | 9,75E-04 | 1.09 |
| 1367218 | AA3_2 | 16188 | 0.00 | NA | NA | 1.21 | 1,77E-09 | 2,59E-06 | -1.21 |
| 1368367 | AA3_2 | 54503 | 1.83 | 1,65E-07 | 3,99E-04 | 0.00 | NA | NA | 1.83 |
| 1370120 | AA3_2 | 610 | 1.27 | 2,94E-08 | 6,45E-05 | 0.00 | NA | NA | 1.27 |
| 1429888 | AA3_2 | 3765 | 5.32 | 1,58E-62 | 7,91E-61 | 6.67 | 3,67E-98 | 4,40E-97 | -1.35 |
| 1432108 | AA3_2 | 7138 | 1.76 | 1,90E-07 | 4,63E-04 | 1.53 | 8,62E-06 | 2,12E-02 | 0.23 |
| 1433476 | AA3_2 | 439 | 0.00 | NA | NA | -2.28 | 4,96E-21 | 2,86E-18 | 2.28 |
| 1434275 | AA3_2 | 520 | 0.00 | NA | NA | 1.80 | 9,81E-09 | 1,56E-05 | -1.80 |
| 1436734 | AA3_2 | 1469 | -1.25 | 4,03E-08 | 9,02E-05 | 0.00 | NA | NA | -1.25 |
| 1462838 | AA3_2 | 650 | -3.11 | 6,29E-31 | 1,93E-28 | -2.20 | 2,49E-16 | 2,00E-13 | -0.91 |
| 1463000 | AA3_2 | 6518 | 2.69 | 3,76E-20 | 2,50E-17 | 2.34 | 2,12E-15 | 1,81E-12 | 0.35 |
| 1465734 | AA3_2 | 3939 | 3.90 | 8,27E-36 | 1,79E-33 | 3.26 | 3,19E-25 | 1,36E-22 | 0.65 |
| 1466067 | AA3_2 | 998 | 0.00 | NA | NA | 0.00 | NA | NA | 0.00 |
| 1368318 | AA3_3 | 35869 | 7.53 | 6,89E-68 | 2,69E-66 | 8.30 | 1,98E-82 | 5,75E-81 | -0.77 |
| 1414868 | AA3_3 | 3997 | 0.00 | NA | NA | 1.84 | 2,45E-09 | 3,64E-06 | -1.84 |
| 1465271 | AA3_3 | 11354 | 4.16 | 3,30E-32 | 9,21E-30 | 4.78 | 2,18E-42 | 3,86E-40 | -0.62 |
| 1440372 | AA3_4 | 3169 | 3.38 | 1,83E-12 | 2,52E-09 | 0.00 | NA | NA | 3.38 |
| 1471985 | AA3_4 | 56520 | 2.56 | 2,68E-12 | 3,77E-09 | 0.00 | NA | NA | 2.56 |
| 1357891 | AA5_1 | 60787 | 0.00 | NA | NA | 0.00 | NA | NA | 0.00 |
| 1376269 | AA5_1 | 2298 | 3.99 | 1,33E-17 | 1,10E-14 | 5.90 | 2,32E-37 | 5,18E-35 | -1.91 |
| 1454649 | AA5_1 | 907 | 4.02 | 4,06E-16 | 3,86E-13 | 5.67 | 4,10E-31 | 1,28E-28 | -1.65 |
| 1480943 | AA5_1 | 5294 | 3.17 | 3,54E-08 | 7,89E-05 | 5.56 | 6,89E-23 | 3,48E-20 | -2.39 |
| 1463150 | AA6 | 20037 | -1.15 | 1,18E-07 | 2,82E-04 | -2.16 | 2,13E-24 | 9,68E-22 | 1.01 |
| 1401955 | AA8-AA3_1 | 19421 | 4.04 | 2,61E-11 | 4,06E-08 | 4.98 | 1,51E-16 | 1,19E-13 | -0.94 |
| 1473160 | AA8-CBM1 | 279 | 0.00 | NA | NA | 0.00 | NA | NA | 0.00 |
| 793241 | AA9 | 3190 | 3.21 | 7,57E-06 | 2,34E-02 | 0.00 | NA | NA | 3.21 |
| 1368522 | AA9 | 1063 | 0.00 | NA | NA | 2.33 | 1,11E-06 | 2,38E-03 | -2.33 |
| 1369390 | AA9 | 1526 | 4.65 | 4,77E-26 | 2,12E-23 | 5.15 | 9,70E-32 | 2,95E-29 | -0.50 |
| 1374028 | AA9 | 63563 | 3.46 | 2,27E-06 | 6,51E-03 | 3.99 | 5,08E-08 | 8,87E-05 | -0.53 |
| 1374858 | AA9 | 309 | 1.56 | 1,35E-06 | 3,74E-03 | 0.00 | NA | NA | 1.56 |
| 1417214 | AA9 | 5260 | 3.62 | 1,14E-08 | 2,39E-05 | 5.69 | 7,69E-20 | 4,80E-17 | -2.08 |
| 1417570 | AA9 | 260 | 1.75 | 3,08E-07 | 7,76E-04 | 0.00 | NA | NA | 1.75 |
| 1423642 | AA9 | 1343 | 0.00 | NA | NA | 2.79 | 1,39E-08 | 2,25E-05 | -2.79 |
| 1437162 | AA9 | 381 | 1.27 | 1,15E-08 | 2,40E-05 | 1.01 | 8,91E-06 | 2,20E-02 | 0.26 |
| 1440889 | AA9 | 7992 | 1.17 | 1,53E-05 | 4,99E-02 | 0.00 | NA | NA | 1.17 |
| 1464041 | AA9 | 10296 | 4.44 | 1,79E-65 | 7,71E-64 | 2.77 | 4,21E-26 | 1,73E-23 | 1.66 |
| 1466495 | AA9 | 2167 | 7.06 | 1,67E-25 | 7,72E-23 | 4.65 | 1,52E-11 | 1,80E-08 | 2.41 |
| 1470943 | AA9 | 845 | 2.46 | 2,82E-17 | 2,44E-14 | 1.59 | 1,01E-07 | 1,82E-04 | 0.87 |
| 1382161 | AA9-CBM1 | 12076 | 4.01 | 1,16E-18 | 8,75E-16 | 5.30 | 6,16E-32 | 1,82E-29 | -1.30 |
| 1428145 | AA9-CBM1 | 15697 | 3.63 | 3,21E-16 | 3,02E-13 | 5.16 | 1,01E-31 | 3,07E-29 | -1.53 |
| 1431096 | CBM1 | 917 | 2.22 | 1,15E-05 | 3,66E-02 | 3.71 | 7,17E-14 | 6,83E-11 | -1.49 |
| 1472584 | CBM1 | 4173 | 3.18 | 1,53E-12 | 2,10E-09 | 5.20 | 7,46E-32 | 2,23E-29 | -2.02 |
| 1390248 | CBM12 | 28743 | 1.42 | 1,21E-12 | 1,63E-09 | 1.75 | 1,51E-18 | 1,04E-15 | -0.33 |
| 1439081 | CBM13 | 456 | 6.10 | 2,44E-37 | 4,85E-35 | 4.58 | 2,13E-21 | 1,18E-18 | 1.52 |
| 1440737 | CBM13 | 4135 | 0.00 | NA | NA | 0.00 | NA | NA | 0.00 |
| 1464925 | CBM13 | 225101 | 0.00 | NA | NA | 0.00 | NA | NA | 0.00 |
| 1447621 | CBM18-GH16 | 5521 | 1.79 | 4,66E-12 | 6,71E-09 | 0.00 | NA | NA | 1.79 |
| 1377160 | CBM1-CE1 | 2292 | 2.46 | 2,52E-11 | 3,90E-08 | 7.72 | 1,74E-104 | 1,74E-103 | -5.26 |
| 1377173 | CBM1-CE1 | 2136 | 5.74 | 1,01E-19 | 7,03E-17 | 5.31 | 5,72E-17 | 4,36E-14 | 0.43 |
| 1392142 | CBM1-CE1 | 2141 | 0.00 | NA | NA | 4.87 | 5,94E-38 | 1,26E-35 | -4.87 |
| 1470260 | CBM1-CE15 | 2833 | 2.95 | 1,63E-06 | 4,58E-03 | 4.20 | 5,05E-12 | 5,71E-09 | -1.25 |
| 1439328 | CBM1-CE16 | 11541 | 6.50 | 4,87E-27 | 2,01E-24 | 5.72 | 4,60E-21 | 2,62E-18 | 0.79 |
| 1426850 | CBM1-GH10 | 3579 | 3.13 | 2,45E-23 | 1,31E-20 | 6.47 | 5,92E-97 | 7,69E-96 | -3.33 |
| 1435885 | CBM1-GH10 | 3161 | 5.10 | 1,18E-14 | 1,30E-11 | 5.45 | 1,62E-16 | 1,28E-13 | -0.35 |
| 1437837 | CBM1-GH10 | 2656 | 4.20 | 1,90E-10 | 3,24E-07 | 4.55 | 6,12E-12 | 6,97E-09 | -0.34 |
| 1404940 | CBM1-GH3 | 2632 | 0.00 | NA | NA | 0.00 | NA | NA | 0.00 |
| 1375024 | CBM1-GH5_5 | 4658 | 5.55 | 1,68E-19 | 1,18E-16 | 5.51 | 3,20E-19 | 2,09E-16 | 0.04 |
| 1429791 | CBM1-GH5_5 | 3557 | 5.15 | 2,19E-17 | 1,87E-14 | 6.24 | 5,32E-25 | 2,32E-22 | -1.09 |
| 1434718 | CBM1-GH5_5 | 3894 | 2.47 | 1,92E-07 | 4,68E-04 | 5.90 | 1,66E-37 | 3,66E-35 | -3.42 |
| 1359888 | CBM1-GH5_7 | 11373 | 5.20 | 2,02E-19 | 1,43E-16 | 4.98 | 8,13E-18 | 5,82E-15 | 0.22 |
| 1360093 | CBM1-GH5_7 | 105 | 0.00 | NA | NA | 0.00 | NA | NA | 0.00 |
| 1357326 | CBM1-GH6 | 22780 | 5.70 | 1,39E-19 | 9,71E-17 | 6.49 | 5,27E-25 | 2,29E-22 | -0.78 |
| 1370357 | CBM21 | 5296 | 0.00 | NA | NA | 0.00 | NA | NA | 0.00 |
| 1069682 | CBM50 | 7916 | 1.47 | 1,50E-06 | 4,18E-03 | 0.00 | NA | NA | 1.47 |
| 1427559 | CBM50 | 11132 | -2.64 | 1,39E-18 | 1,06E-15 | -3.77 | 1,01E-36 | 2,32E-34 | 1.13 |
| 1436697 | CBM50 | 4348 | 0.00 | NA | NA | 0.00 | NA | NA | 0.00 |
| 1470261 | CE15 | 2637 | 5.22 | 1,88E-38 | 3,51E-36 | 7.43 | 2,97E-77 | 1,01E-75 | -2.20 |
| 1367487 | CE16 | 584 | 5.28 | 1,55E-28 | 5,81E-26 | 6.06 | 2,33E-37 | 5,23E-35 | -0.78 |
| 1373375 | CE16 | 3912 | 2.68 | 2,44E-12 | 3,41E-09 | 3.60 | 2,07E-21 | 1,14E-18 | -0.92 |
| 1377883 | CE16 | 555 | 0.00 | NA | NA | 0.00 | NA | NA | 0.00 |
| 1386401 | CE16 | 510 | 0.00 | NA | NA | 0.00 | NA | NA | 0.00 |
| 1429512 | CE16 | 3862 | 4.32 | 1,32E-25 | 6,03E-23 | 5.62 | 9,88E-43 | 1,72E-40 | -1.30 |
| 1437703 | CE16 | 4315 | 4.46 | 1,36E-25 | 6,26E-23 | 6.95 | 5,06E-61 | 3,29E-59 | -2.49 |
| 1361091 | CE4 | 35470 | 1.46 | 6,23E-08 | 1,42E-04 | 0.00 | NA | NA | 1.46 |
| 1433098 | CE4 | 5412 | 4.20 | 2,22E-54 | 1,73E-52 | 1.69 | 1,38E-09 | 1,98E-06 | 2.51 |
| 1367399 | CE8 | 784 | 2.08 | 3,10E-11 | 4,87E-08 | 2.32 | 1,42E-13 | 1,39E-10 | -0.23 |
| 1438246 | CE8 | 4119 | 4.93 | 1,78E-53 | 1,44E-51 | 4.52 | 3,58E-45 | 5,40E-43 | 0.41 |
| 1396409 | CE9 | 6477 | 0.00 | NA | NA | 0.00 | NA | NA | 0.00 |
| 148891 | EXP | 40 | -2.77 | 1,58E-13 | 1,93E-10 | -1.69 | 2,90E-07 | 5,61E-04 | -1.07 |
| 1422224 | EXPN | 1037 | 0.00 | NA | NA | -2.11 | 1,65E-11 | 1,96E-08 | 2.11 |
| 1373750 | EXPN | 1217 | -4.09 | 3,78E-29 | 1,37E-26 | -5.26 | 6,32E-47 | 8,41E-45 | 1.17 |
| 1386069 | EXPN | 2365 | -2.13 | 2,50E-06 | 7,21E-03 | -4.31 | 1,78E-22 | 9,19E-20 | 2.18 |
| 1477520 | EXPN | 3554 | 0.00 | NA | NA | -2.47 | 1,74E-12 | 1,88E-09 | 2.47 |
| 316489 | EXPN | 5546 | 2.51 | 3,88E-22 | 2,24E-19 | 0.00 | NA | NA | 2.51 |
| 1467568 | EXPN | 16280 | 1.89 | 5,72E-12 | 8,32E-09 | 1.25 | 9,32E-06 | 2,31E-02 | 0.64 |
| 1439344 | EXPN | 16289 | 0.00 | NA | NA | -2.04 | 8,16E-08 | 1,46E-04 | 2.04 |
| 1451679 | EXPN | 22898 | 1.82 | 5,04E-13 | 6,47E-10 | 0.00 | NA | NA | 1.82 |
| 1440495 | EXPN | 77285 | 0.00 | NA | NA | 0.00 | NA | NA | 0.00 |
| 1370248 | GH1 | 36358 | 2.83 | 1,76E-17 | 1,49E-14 | 3.27 | 5,57E-23 | 2,78E-20 | -0.44 |
| 1449232 | GH1 | 1305 | 2.77 | 1,71E-15 | 1,72E-12 | 4.23 | 5,73E-35 | 1,48E-32 | -1.46 |
| 1362920 | GH10 | 172 | 0.00 | NA | NA | 0.00 | NA | NA | 0.00 |
| 1395316 | GH10 | 2128 | 1.68 | 1,19E-06 | 3,28E-03 | 8.12 | 1,40E-140 | 4,21E-140 | -6.44 |
| 1419320 | GH10 | 773 | 3.95 | 1,36E-20 | 8,75E-18 | 5.97 | 4,31E-46 | 6,16E-44 | -2.02 |
| 1426831 | GH115 | 7235 | 4.63 | 7,24E-28 | 2,84E-25 | 6.12 | 4,53E-48 | 5,62E-46 | -1.49 |
| 1358049 | GH12 | 1791 | 6.37 | 3,98E-42 | 5,84E-40 | 5.38 | 2,37E-30 | 7,71E-28 | 0.99 |
| 1373549 | GH125 | 2970 | 0.00 | NA | NA | 0.00 | NA | NA | 0.00 |
| 1427524 | GH128 | 2709 | 0.00 | NA | NA | -1.46 | 8,32E-07 | 1,74E-03 | 1.46 |
| 1438566 | GH128 | 42490 | 1.42 | 2,52E-09 | 4,88E-06 | 0.00 | NA | NA | 1.42 |
| 1439107 | GH128 | 1471 | 0.00 | NA | NA | 1.03 | 7,70E-06 | 1,88E-02 | -1.03 |
| 1435071 | GH13_1 | 13987 | 2.25 | 3,27E-15 | 3,39E-12 | 1.82 | 3,06E-10 | 4,10E-07 | 0.43 |
| 1523626 | GH13_1 | 8742 | 0.00 | NA | NA | 0.00 | NA | NA | 0.00 |
| 1376091 | GH131 | 502 | 0.00 | NA | NA | 3.73 | 2,00E-09 | 2,95E-06 | -3.73 |
| 1464770 | GH131 | 806 | 0.00 | NA | NA | 0.00 | NA | NA | 0.00 |
| 1467772 | GH131-CBM1 | 13184 | 4.05 | 6,94E-10 | 1,26E-06 | 4.76 | 3,56E-13 | 3,56E-10 | -0.72 |
| 1439526 | GH15 | 6378 | -2.89 | 5,04E-48 | 5,49E-46 | -3.35 | 4,85E-64 | 2,81E-62 | 0.45 |
| 1507511 | GH15-CBM20 | 8454 | 3.80 | 1,12E-29 | 3,90E-27 | 0.00 | NA | NA | 3.80 |
| 65788 | GH16 | 26729 | 2.00 | 9,03E-10 | 1,65E-06 | 0.00 | NA | NA | 2.00 |
| 651762 | GH16 | 3633 | 0.00 | NA | NA | 1.58 | 9,43E-11 | 1,21E-07 | -1.58 |
| 1358415 | GH16 | 19709 | 0.00 | NA | NA | 0.00 | NA | NA | 0.00 |
| 1361613 | GH16 | 920 | 1.50 | 1,85E-12 | 2,55E-09 | 1.54 | 5,71E-13 | 5,84E-10 | -0.04 |
| 1363264 | GH16 | 299 | 0.00 | NA | NA | 0.00 | NA | NA | 0.00 |
| 1367729 | GH16 | 3511 | -1.87 | 2,67E-10 | 4,61E-07 | -4.11 | 2,67E-45 | 3,98E-43 | 2.24 |
| 1372016 | GH16 | 3588 | 0.00 | NA | NA | 1.27 | 5,63E-07 | 1,15E-03 | -1.27 |
| 1390014 | GH16 | 12768 | 1.39 | 9,43E-09 | 1,96E-05 | 0.00 | NA | NA | 1.39 |
| 1414778 | GH16 | 10 | -2.98 | 4,75E-06 | 1,43E-02 | 0.00 | NA | NA | -2.98 |
| 1422852 | GH16 | 13287 | 0.00 | NA | NA | 0.00 | NA | NA | 0.00 |
| 1430328 | GH16 | 216 | -1.74 | 4,85E-09 | 9,69E-06 | -2.70 | 1,84E-19 | 1,19E-16 | 0.96 |
| 1434602 | GH16 | 12319 | 2.47 | 2,65E-28 | 1,01E-25 | 1.35 | 3,80E-09 | 5,77E-06 | 1.11 |
| 1434603 | GH16 | 7040 | 0.00 | NA | NA | 0.00 | NA | NA | 0.00 |
| 1442230 | GH16 | 8703 | 0.00 | NA | NA | 0.00 | NA | NA | 0.00 |
| 1445175 | GH16 | 2263 | 0.00 | NA | NA | 1.90 | 2,32E-07 | 4,42E-04 | -1.90 |
| 1445188 | GH16 | 8003 | 3.78 | 3,36E-25 | 1,59E-22 | 2.85 | 1,20E-14 | 1,09E-11 | 0.93 |
| 1463396 | GH16 | 10301 | 1.78 | 1,00E-09 | 1,85E-06 | 0.00 | NA | NA | 1.78 |
| 1465104 | GH16 | 1864 | 0.00 | NA | NA | 0.00 | NA | NA | 0.00 |
| 1465130 | GH16 | 5047 | 1.98 | 1,99E-33 | 5,08E-31 | 1.33 | 1,54E-15 | 1,30E-12 | 0.65 |
| 1473510 | GH16 | 9049 | 2.19 | 1,16E-13 | 1,40E-10 | 0.00 | NA | NA | 2.19 |
| 1114620 | GH17 | 11699 | 0.00 | NA | NA | 0.00 | NA | NA | 0.00 |
| 1433845 | GH17 | 13044 | 0.00 | NA | NA | 0.00 | NA | NA | 0.00 |
| 1364582 | GH18 | 139 | 4.00 | 2,59E-17 | 2,23E-14 | 2.32 | 2,24E-06 | 4,99E-03 | 1.67 |
| 1424871 | GH18 | 1524 | 0.00 | NA | NA | 0.00 | NA | NA | 0.00 |
| 1433997 | GH18 | 34958 | 2.14 | 1,64E-15 | 1,65E-12 | 1.45 | 1,55E-07 | 2,85E-04 | 0.70 |
| 1444708 | GH18 | 359 | -1.71 | 1,38E-05 | 4,45E-02 | -3.22 | 1,06E-16 | 8,27E-14 | 1.51 |
| 1445051 | GH18 | 8246 | 2.64 | 7,32E-13 | 9,61E-10 | 1.80 | 1,91E-06 | 4,22E-03 | 0.84 |
| 1464569 | GH18 | 1003 | 0.00 | NA | NA | -2.40 | 2,49E-08 | 4,16E-05 | 2.40 |
| 1467254 | GH18 | 363 | 0.00 | NA | NA | -3.32 | 6,36E-15 | 5,59E-12 | 3.32 |
| 1471466 | GH18 | 5398 | 1.88 | 2,27E-10 | 3,88E-07 | 2.97 | 3,14E-24 | 1,45E-21 | -1.09 |
| 1106774 | GH18-CBM5 | 392 | 0.00 | NA | NA | -1.45 | 1,47E-05 | 3,74E-02 | 1.45 |
| 1447824 | GH18-CBM5 | 12407 | 0.00 | NA | NA | 1.58 | 4,37E-08 | 7,55E-05 | -1.58 |
| 1456924 | GH18-CBM5 | 4093 | 2.35 | 5,90E-13 | 7,64E-10 | 3.19 | 6,26E-23 | 3,14E-20 | -0.84 |
| 1463186 | GH18-CBM5 | 5877 | 0.00 | NA | NA | 1.63 | 3,63E-09 | 5,50E-06 | -1.63 |
| 1463187 | GH18-CBM5 | 478 | 0.00 | NA | NA | 0.00 | NA | NA | 0.00 |
| 1369807 | GH2 | 3756 | 3.20 | 5,90E-32 | 1,66E-29 | 2.83 | 3,52E-25 | 1,51E-22 | 0.37 |
| 1438782 | GH2 | 1279 | 2.21 | 1,27E-12 | 1,72E-09 | 1.92 | 9,95E-10 | 1,41E-06 | 0.28 |
| 650518 | GH20 | 711 | 2.67 | 1,50E-33 | 3,83E-31 | 2.76 | 5,49E-36 | 1,31E-33 | -0.09 |
| 1418425 | GH20 | 2884 | 0.00 | NA | NA | 0.00 | NA | NA | 0.00 |
| 1434713 | GH20 | 1062 | 1.40 | 2,50E-06 | 7,22E-03 | 0.00 | NA | NA | 1.40 |
| 1439660 | GH20 | 587 | 1.89 | 1,42E-05 | 4,58E-02 | 2.73 | 2,15E-10 | 2,85E-07 | -0.84 |
| 1464008 | GH23 | 5696 | 1.84 | 1,68E-20 | 1,09E-17 | 1.06 | 2,07E-07 | 3,90E-04 | 0.78 |
| 1471070 | GH25 | 12771 | 4.66 | 8,52E-45 | 1,11E-42 | 5.17 | 4,68E-55 | 3,93E-53 | -0.51 |
| 1070165 | GH27 | 82 | 0.00 | NA | NA | 2.33 | 1,05E-05 | 2,61E-02 | -2.33 |
| 1431843 | GH27 | 12772 | 1.91 | 7,91E-14 | 9,34E-11 | 1.35 | 2,27E-07 | 4,31E-04 | 0.56 |
| 1440257 | GH27 | 10278 | 3.21 | 1,09E-17 | 8,95E-15 | 2.94 | 6,92E-15 | 6,10E-12 | 0.28 |
| 1464704 | GH27 | 8 | 0.00 | NA | NA | 0.00 | NA | NA | 0.00 |
| 688728 | GH28 | 4781 | 5.79 | 1,39E-37 | 2,74E-35 | 4.53 | 2,21E-23 | 1,08E-20 | 1.26 |
| 1370654 | GH28 | 6009 | 4.95 | 4,04E-16 | 3,83E-13 | 4.59 | 6,46E-14 | 6,14E-11 | 0.36 |
| 1377553 | GH28 | 3020 | 4.12 | 1,78E-25 | 8,28E-23 | 4.28 | 1,80E-27 | 6,77E-25 | -0.17 |
| 1418820 | GH28 | 1214 | 2.61 | 2,26E-15 | 2,30E-12 | 2.44 | 1,52E-13 | 1,49E-10 | 0.16 |
| 1439310 | GH28 | 3701 | 4.62 | 6,30E-26 | 2,82E-23 | 4.36 | 4,31E-23 | 2,14E-20 | 0.27 |
| 1446047 | GH28 | 2569 | 3.28 | 7,88E-37 | 1,63E-34 | 3.97 | 5,89E-54 | 5,24E-52 | -0.70 |
| 1469372 | GH28 | 1110 | 2.90 | 1,15E-24 | 5,67E-22 | 2.78 | 9,09E-23 | 4,63E-20 | 0.12 |
| 1387896 | GH3 | 885 | 0.00 | NA | NA | 0.00 | NA | NA | 0.00 |
| 1411666 | GH3 | 3954 | 3.79 | 1,69E-34 | 4,12E-32 | 4.56 | 1,33E-49 | 1,51E-47 | -0.77 |
| 1434274 | GH3 | 3487 | 2.16 | 9,80E-08 | 2,31E-04 | 0.00 | NA | NA | 2.16 |
| 1435009 | GH3 | 3942 | 0.00 | NA | NA | 1.76 | 8,38E-08 | 1,50E-04 | -1.76 |
| 1468141 | GH3 | 5874 | 3.24 | 2,04E-14 | 2,27E-11 | 3.07 | 5,46E-13 | 5,57E-10 | 0.17 |
| 1474954 | GH3 | 1229 | 1.38 | 2,73E-07 | 6,82E-04 | 2.45 | 1,25E-20 | 7,37E-18 | -1.07 |
| 1357645 | GH30 | 4652 | 4.23 | 1,69E-19 | 1,19E-16 | 3.57 | 4,64E-14 | 4,35E-11 | 0.67 |
| 1358510 | GH30_3 | 516 | -2.10 | 4,58E-13 | 5,85E-10 | -1.41 | 1,83E-06 | 4,04E-03 | -0.69 |
| 1430584 | GH30_3 | 10565 | 0.00 | NA | NA | 1.56 | 5,45E-07 | 1,11E-03 | -1.56 |
| 1361992 | GH31 | 2738 | 0.00 | NA | NA | 1.34 | 1,05E-10 | 1,36E-07 | -1.34 |
| 1435128 | GH31 | 4792 | 1.38 | 1,56E-09 | 2,94E-06 | 1.57 | 6,77E-12 | 7,75E-09 | -0.19 |
| 1468129 | GH31 | 5217 | 2.46 | 1,10E-18 | 8,27E-16 | 2.51 | 1,85E-19 | 1,20E-16 | -0.06 |
| 718112 | GH32 | 3393 | 6.44 | 2,36E-76 | 6,13E-75 | 5.46 | 4,30E-55 | 3,57E-53 | 0.98 |
| 1439507 | GH35 | 806 | 3.97 | 1,12E-17 | 9,26E-15 | 2.63 | 3,00E-08 | 5,08E-05 | 1.34 |
| 1439531 | GH35 | 10729 | 4.75 | 6,68E-30 | 2,28E-27 | 4.18 | 2,49E-23 | 1,22E-20 | 0.57 |
| 1480666 | GH38 | 8180 | 1.10 | 3,75E-07 | 9,58E-04 | 1.07 | 9,96E-07 | 2,11E-03 | 0.03 |
| 408613 | GH43 | 1858 | 4.81 | 1,62E-29 | 5,72E-27 | 3.92 | 7,04E-20 | 4,37E-17 | 0.89 |
| 1435501 | GH43 | 6091 | 6.24 | 2,22E-16 | 2,04E-13 | 5.00 | 8,04E-11 | 1,02E-07 | 1.24 |
| 1445623 | GH43-CBM35 | 3087 | 0.00 | NA | NA | 0.00 | NA | NA | 0.00 |
| 1378034 | GH45 | 149 | 2.99 | 6,22E-12 | 9,10E-09 | 4.26 | 9,81E-24 | 4,62E-21 | -1.27 |
| 1433077 | GH45 | 5837 | 6.85 | 3,96E-74 | 1,23E-72 | 7.32 | 1,24E-84 | 3,22E-83 | -0.47 |
| 1369367 | GH47 | 16417 | 0.00 | NA | NA | 2.86 | 5,86E-13 | 6,00E-10 | -2.86 |
| 1423703 | GH47 | 3105 | 2.58 | 1,03E-18 | 7,69E-16 | 0.00 | NA | NA | 2.58 |
| 1436992 | GH47 | 4446 | 0.00 | NA | NA | 1.34 | 6,21E-08 | 1,09E-04 | -1.34 |
| 1468106 | GH5_12 | 1851 | 0.00 | NA | NA | -1.64 | 3,01E-12 | 3,32E-09 | 1.64 |
| 1432550 | GH5_15 | 10119 | 2.44 | 4,41E-13 | 5,63E-10 | 4.09 | 4,88E-35 | 1,26E-32 | -1.65 |
| 1453322 | GH5_22 | 234 | 0.00 | NA | NA | 1.62 | 3,32E-07 | 6,48E-04 | -1.62 |
| 1460251 | GH5_22 | 8775 | 0.00 | NA | NA | 2.05 | 5,45E-15 | 4,75E-12 | -2.05 |
| 236998 | GH5_7 | 287 | -3.30 | 1,74E-29 | 6,15E-27 | -1.76 | 3,15E-10 | 4,24E-07 | -1.53 |
| 1388575 | GH5_7 | 813 | 1.81 | 6,19E-06 | 1,89E-02 | 3.18 | 4,97E-16 | 4,07E-13 | -1.36 |
| 1425475 | GH5_9 | 4065 | 1.27 | 3,86E-07 | 9,90E-04 | 0.00 | NA | NA | 1.27 |
| 1438957 | GH5_9 | 13207 | 0.00 | NA | NA | 0.00 | NA | NA | 0.00 |
| 1357629 | GH51 | 9190 | 4.75 | 2,55E-25 | 1,20E-22 | 4.67 | 1,85E-24 | 8,36E-22 | 0.08 |
| 1462467 | GH51 | 490 | 5.58 | 4,12E-49 | 4,25E-47 | 4.92 | 2,51E-38 | 5,22E-36 | 0.66 |
| 1363671 | GH53 | 2328 | 5.27 | 4,13E-33 | 1,10E-30 | 7.05 | 1,35E-58 | 9,82E-57 | -1.78 |
| 1377179 | GH55 | 7092 | 3.99 | 1,54E-12 | 2,11E-09 | 4.04 | 9,78E-13 | 1,02E-09 | -0.05 |
| 1366028 | GH7 | 6679 | 2.31 | 6,82E-06 | 2,09E-02 | 5.55 | 6,07E-29 | 2,14E-26 | -3.24 |
| 1389216 | GH7 | 13590 | 3.30 | 6,91E-06 | 2,12E-02 | 4.35 | 2,63E-09 | 3,93E-06 | -1.05 |
| 1424818 | GH7 | 93872 | 6.00 | 3,61E-18 | 2,87E-15 | 5.66 | 3,20E-16 | 2,59E-13 | 0.34 |
| 1463537 | GH71 | 956 | 2.29 | 1,54E-13 | 1,88E-10 | 2.06 | 4,57E-11 | 5,65E-08 | 0.23 |
| 1463547 | GH71 | 297 | 0.00 | NA | NA | 0.00 | NA | NA | 0.00 |
| 1361311 | GH72-CBM43 | 12943 | 1.14 | 1,08E-05 | 3,41E-02 | 0.00 | NA | NA | 1.14 |
| 1365564 | GH74-CBM1 | 22558 | 6.23 | 1,02E-50 | 9,54E-49 | 5.71 | 8,80E-43 | 1,51E-40 | 0.52 |
| 320973 | GH76 | 5968 | 5.06 | 9,32E-39 | 1,73E-36 | 4.45 | 3,98E-30 | 1,30E-27 | 0.62 |
| 1067038 | GH78 | 1207 | 4.21 | 1,16E-18 | 8,73E-16 | 2.31 | 3,09E-06 | 7,04E-03 | 1.91 |
| 1434191 | GH78 | 2315 | 4.69 | 1,23E-31 | 3,51E-29 | 3.57 | 1,03E-18 | 7,02E-16 | 1.12 |
| 1374205 | GH79 | 13364 | 1.62 | 2,26E-23 | 1,20E-20 | 1.28 | 5,88E-15 | 5,15E-12 | 0.34 |
| 1413725 | GH79 | 121 | 3.36 | 8,95E-14 | 1,06E-10 | 0.00 | NA | NA | 3.36 |
| 1435894 | GH79 | 33130 | 4.78 | 1,08E-55 | 7,81E-54 | 4.63 | 2,48E-52 | 2,48E-50 | 0.15 |
| 1447349 | GH79 | 11634 | 2.23 | 2,48E-16 | 2,29E-13 | 0.00 | NA | NA | 2.23 |
| 1468712 | GH79 | 8821 | 3.92 | 1,16E-17 | 9,55E-15 | 3.11 | 2,03E-11 | 2,43E-08 | 0.81 |
| 1452485 | GH85 | 1082 | 0.00 | NA | NA | 0.93 | 1,28E-07 | 2,35E-04 | -0.93 |
| 1378812 | GH88 | 5087 | 3.55 | 4,70E-33 | 1,26E-30 | 4.05 | 9,10E-43 | 1,57E-40 | -0.50 |
| 961999 | GH9 | 168 | 0.00 | NA | NA | 0.00 | NA | NA | 0.00 |
| 1373467 | GH92 | 16529 | 0.00 | NA | NA | 1.85 | 7,66E-07 | 1,59E-03 | -1.85 |
| 1390886 | GH92 | 1971 | 3.13 | 2,27E-14 | 2,54E-11 | 3.27 | 1,52E-15 | 1,28E-12 | -0.14 |
| 1435879 | GH92 | 2987 | 0.96 | 1,45E-06 | 4,03E-03 | 0.00 | NA | NA | 0.96 |
| 1453868 | GH92 | 13253 | 4.71 | 5,59E-56 | 3,86E-54 | 5.41 | 1,15E-73 | 4,37E-72 | -0.70 |
| 1514584 | GH92 | 3695 | 0.00 | NA | NA | 1.91 | 6,97E-09 | 1,09E-05 | -1.91 |
| 1443101 | GH95 | 7196 | 1.66 | 2,28E-06 | 6,55E-03 | 1.61 | 6,30E-06 | 1,51E-02 | 0.05 |
| 289705 | GT1 | 1489 | -4.49 | 7,45E-30 | 2,55E-27 | -4.49 | 6,74E-30 | 2,24E-27 | 0.00 |
| 1403531 | GT1 | 775 | 0.00 | NA | NA | 0.00 | NA | NA | 0.00 |
| 1412761 | GT1 | 69 | 0.00 | NA | NA | 0.00 | NA | NA | 0.00 |
| 1422291 | GT1 | 70 | 0.00 | NA | NA | 0.00 | NA | NA | 0.00 |
| 1424219 | GT1 | 645 | -4.05 | 3,46E-57 | 2,18E-55 | -3.62 | 1,63E-47 | 2,11E-45 | -0.43 |
| 1466368 | GT1 | 882 | 0.00 | NA | NA | 1.52 | 2,57E-12 | 2,82E-09 | -1.52 |
| 1470544 | GT1 | 1779 | 1.64 | 2,32E-27 | 9,38E-25 | 1.53 | 1,15E-23 | 5,47E-21 | 0.12 |
| 1472250 | GT1 | 140 | 1.53 | 3,52E-10 | 6,18E-07 | 0.00 | NA | NA | 1.53 |
| 1476435 | GT1 | 544 | -4.37 | 4,44E-30 | 1,48E-27 | -4.90 | 2,60E-37 | 5,86E-35 | 0.53 |
| 1446713 | GT15 | 2239 | 0.00 | NA | NA | 0.00 | NA | NA | 0.00 |
| 1427488 | GT2 | 15230 | 1.40 | 6,21E-10 | 1,12E-06 | 1.01 | 1,50E-05 | 3,84E-02 | 0.40 |
| 1436848 | GT2 | 14981 | 1.62 | 1,37E-06 | 3,79E-03 | 1.49 | 1,18E-05 | 2,96E-02 | 0.13 |
| 1439511 | GT2 | 8103 | 1.10 | 3,65E-07 | 9,33E-04 | 1.55 | 5,90E-13 | 6,05E-10 | -0.44 |
| 1447996 | GT2 | 17557 | 0.00 | NA | NA | 0.00 | NA | NA | 0.00 |
| 1467191 | GT2 | 19534 | 2.40 | 6,61E-13 | 8,61E-10 | 0.00 | NA | NA | 2.40 |
| 1442619 | GT20 | 6537 | 1.31 | 1,01E-08 | 2,09E-05 | 0.00 | NA | NA | 1.31 |
| 1444629 | GT21 | 1979 | -0.86 | 1,45E-06 | 4,03E-03 | -1.04 | 4,45E-09 | 6,83E-06 | 0.18 |
| 1370181 | GT22 | 1128 | 0.00 | NA | NA | 0.00 | NA | NA | 0.00 |
| 1442917 | GT22 | 1923 | 0.00 | NA | NA | -1.19 | 2,17E-06 | 4,84E-03 | 1.19 |
| 1473155 | GT22 | 1374 | 1.83 | 1,12E-23 | 5,82E-21 | 1.34 | 4,07E-13 | 4,10E-10 | 0.49 |
| 1364471 | GT32 | 9553 | 2.27 | 8,80E-13 | 1,16E-09 | 0.00 | NA | NA | 2.27 |
| 1405373 | GT33 | 696 | 0.00 | NA | NA | 0.00 | NA | NA | 0.00 |
| 1434813 | GT39 | 7978 | 0.00 | NA | NA | 0.00 | NA | NA | 0.00 |
| 1376072 | GT4 | 2201 | 0.64 | 5,96E-06 | 1,81E-02 | 0.00 | NA | NA | 0.64 |
| 1433732 | GT57 | 3318 | 1.59 | 9,08E-18 | 7,43E-15 | 1.04 | 4,57E-08 | 7,91E-05 | 0.55 |
| 1435520 | GT69 | 3112 | 1.16 | 3,53E-07 | 8,98E-04 | 0.00 | NA | NA | 1.16 |
| 1379069 | GT8 | 763 | 0.00 | NA | NA | 0.00 | NA | NA | 0.00 |
| 1400833 | GT8 | 3388 | 0.00 | NA | NA | -1.83 | 3,21E-07 | 6,24E-04 | 1.83 |
| 1435027 | GT8 | 2156 | 1.58 | 4,51E-10 | 7,99E-07 | 0.00 | NA | NA | 1.58 |
| 1458648 | GT8 | 1852 | 0.00 | NA | NA | 0.00 | NA | NA | 0.00 |
| 1464752 | GT8 | 381 | 0.00 | NA | NA | 1.95 | 1,92E-12 | 2,08E-09 | -1.95 |
| 1478195 | GT8 | 30 | 0.00 | NA | NA | 0.00 | NA | NA | 0.00 |
| 1390652 | PL14 | 41 | 0.00 | NA | NA | -2.14 | 6,13E-06 | 1,46E-02 | 2.14 |
| 1425521 | PL14 | 441 | 4.68 | 7,62E-35 | 1,78E-32 | 3.63 | 3,22E-21 | 1,82E-18 | 1.05 |
| 1447227 | PL14 | 204 | 2.50 | 2,40E-21 | 1,46E-18 | 2.34 | 8,34E-19 | 5,61E-16 | 0.16 |
| 1452408 | PL14 | 27144 | 1.91 | 4,09E-13 | 5,20E-10 | 0.00 | NA | NA | 1.91 |
| 1409152 | PL4 | 436 | 0.00 | NA | NA | 0.00 | NA | NA | 0.00 |
| 1444442 | PL8 | 7755 | 4.55 | 1,12E-37 | 2,19E-35 | 3.85 | 3,67E-27 | 1,41E-24 | 0.71 |
| 1466845 | PL8 | 12405 | 0.00 | NA | NA | 0.00 | NA | NA | 0.00 |

The table shows the mean number of reads for each gene in the maltose control condition and the fold change of transcript levels expressed as log2 on each substrate as compared to maltose.

padj: p value adjusted with the Benjamini-Hochberg method

padj_Bonf: p value adjusted with the Bonferroni method

P/A: Ratio of log2Fold Change_PIN/ log2Fold Change_ASP

NA: Not Applicable

**Supplementary Table S3**: CAZymes identified in proteome analyses

|  |  |  | Number of identified peptides | | |
| --- | --- | --- | --- | --- | --- |
| Protein ID | Type | CAZY family | PcoMaltose | PcoPine | PcoAspen |
| 1366139 | REDOX | AA1_1 | 154 | 57 | 61 |
| 1434777 | REDOX | AA1_1 | 14 | Nd | Nd |
| 1452465 | REDOX | AA1_1 | Nd | 7 | 9 |
| 1477425 | REDOX | AA1_1 | 12 | Nd | Nd |
| 1436321 | REDOX | AA2 | Nd | 6 | Nd |
| 1438352 | REDOX | AA2 | 2 | Nd | Nd |
| 1464049 | REDOX | AA2 | Nd | Nd | 8 |
| 1401955 | REDOX | AA3_1-AA8 | Nd | 7 | 8 |
| 68108 | REDOX | AA3_2 | 20 | Nd | Nd |
| 1432108 | REDOX | AA3_2 | 9 | 3 | 4 |
| 1465734 | REDOX | AA3_2 | 11 | Nd | Nd |
| 1357891 | REDOX | AA5_1 | 13 | 17 | 8 |
| 1367686 | REDOX | AA5_1 | 51 | 12 | 6 |
| 1374028 | REDOX | AA9 | Nd | 4 | 4 |
| 1423642 | REDOX | AA9 | Nd | Nd | 3 |
| 1427559 | CAZY | CBM50 [x2] | 5 | 2 | Nd |
| 1377173 | CAZY | CBM1-CE1 | Nd | Nd | 4 |
| 1392142 | CAZY | CBM1-CE1 | Nd | Nd | 2 |
| 1361091 | CAZY | CE4 | 4 | Nd | 3 |
| 1438246 | CAZY | CE8 | 7 | 12 | 11 |
| 1373375 | CAZY | CE16 | 8 | 9 | 9 |
| 1451679 | CAZY | EXPN | 5 | 7 | 5 |
| 1369807 | CAZY | GH2 | 9 | 13 | 13 |
| 1411666 | CAZY | GH3 | Nd | 14 | 8 |
| 1404940 | CAZY | GH3-CBM1 | Nd | 2 | 5 |
| 1421509 | CAZY | GH5_9 | 2 | 5 | 6 |
| 1425475 | CAZY | GH5_9 | 14 | 5 | 11 |
| 1375024 | CAZY | CBM1-GH5_5 | Nd | 2 | 6 |
| 1429791 | CAZY | CBM1-GH5_5 | Nd | 3 | 4 |
| 1434718 | CAZY | CBM1-GH5_5 | Nd | 2 | 4 |
| 1359888 | CAZY | CBM1-GH5_7 | Nd | 6 | 5 |
| 1357326 | CAZY | CBM1-GH6_3 | Nd | 13 | 16 |
| 1366028 | CAZY | GH7 | Nd | 9 | 8 |
| 1389216 | CAZY | GH7 | 6 | 45 | 37 |
| 1424818 | CAZY | GH7 | 11 | 96 | 75 |
| 1395316 | CAZY | GH10 | Nd | Nd | 3 |
| 1426850 | CAZY | CBM1-GH10 | Nd | 6 | 8 |
| 1437837 | CAZY | CBM1-GH10 | Nd | 9 | 7 |
| 1358049 | CAZY | GH12 | Nd | 7 | Nd |
| 1497577 | CAZY | GH13_32-CBM20 | 11 | 7 | 7 |
| 1507511 | CAZY | GH15-CBM20 | 68 | 59 | 64 |
| 651762 | CAZY | GH16 | Nd | 2 | Nd |
| 1361613 | CAZY | GH16 | 2 | 2 | Nd |
| 1372016 | CAZY | GH16 | Nd | Nd | 2 |
| 1422852 | CAZY | GH16 | 4 | Nd | Nd |
| 1434602 | CAZY | GH16 | Nd | 3 | Nd |
| 1442230 | CAZY | GH16 | 3 | 5 | Nd |
| 1445175 | CAZY | GH16 | 15 | 14 | 29 |
| 1447621 | CAZY | GH16 | Nd | 3 | 3 |
| 1451086 | CAZY | GH16 | 4 | 5 | Nd |
| 1473510 | CAZY | GH16 | 4 | 11 | 5 |
| 1433845 | CAZY | GH17 | 3 | 2 | 2 |
| 1424871 | CAZY | GH18 | 9 | 7 | 8 |
| 1428287 | CAZY | GH18 | 4 | Nd | Nd |
| 1433997 | CAZY | GH18 | 6 | 4 | 6 |
| 1434821 | CAZY | GH18 | 10 | 6 | 12 |
| 1445051 | CAZY | GH18 | Nd | 17 | 7 |
| 1471466 | CAZY | GH18 | 5 | 3 | 13 |
| 1106774 | CAZY | GH18-CBM5 | Nd | 4 | 2 |
| 1447824 | CAZY | GH18-CBM5 | Nd | Nd | 5 |
| 1463186 | CAZY | GH18-CBM5 | Nd | Nd | 5 |
| 1463187 | CAZY | GH18-CBM5 | Nd | Nd | 3 |
| 1456924 | CAZY | GH18-CBM5 [x2] | Nd | Nd | 6 |
| 1471070 | CAZY | GH25 | 9 | 4 | 5 |
| 1431843 | CAZY | GH27 | 26 | 32 | 27 |
| 1440257 | CAZY | GH27 | 14 | 14 | 14 |
| 688728 | CAZY | GH28 | Nd | 19 | 16 |
| 1370654 | CAZY | GH28 | Nd | 8 | 9 |
| 1439310 | CAZY | GH28 | Nd | 3 | 4 |
| 1357645 | CAZY | GH30 | 5 | 13 | 15 |
| 1358510 | CAZY | GH30_3 | Nd | 15 | 14 |
| 1430584 | CAZY | GH30_3 | 3 | 9 | 12 |
| 1426153 | CAZY | GH31 | 2 | 4 | 4 |
| 1468129 | CAZY | GH31 | 9 | Nd | Nd |
| 718112 | CAZY | GH32 | 6 | 6 | 8 |
| 1439531 | CAZY | GH35 | 2 | 5 | 18 |
| 1480891 | CAZY | GH37 | 2 | Nd | 3 |
| 408613 | CAZY | GH43 | Nd | 2 | Nd |
| 1435501 | CAZY | GH43 | Nd | 13 | 14 |
| 1445623 | CAZY | GH43-CBM35 | Nd | 8 | 12 |
| 1433077 | CAZY | GH45 | Nd | 2 | 3 |
| 1369367 | CAZY | GH47 | 24 | 2 | 4 |
| 1357629 | CAZY | GH51 | 5 | 25 | 29 |
| 1377179 | CAZY | GH55 | Nd | 30 | 54 |
| 1401294 | CAZY | GH55 | 3 | 12 | 13 |
| 1361311 | CAZY | GH72-CBM43 | Nd | 3 | 3 |
| 1365564 | CAZY | GH74-CBM1 | 2 | 28 | 23 |
| 320973 | CAZY | GH76 | 42 | 30 | 45 |
| 1435894 | CAZY | GH79 | 29 | 28 | 33 |
| 1378812 | CAZY | GH88 | 5 | Nd | Nd |
| 1373467 | CAZY | GH92 | 92 | 147 | 176 |
| 1453868 | CAZY | GH92 | Nd | Nd | 2 |
| 1514584 | CAZY | GH92 | 2 | Nd | 4 |
| 1443101 | CAZY | GH95 | 9 | 18 | 23 |
| 1373549 | CAZY | GH125 | Nd | Nd | Nd |
| 1467772 | CAZY | GH131-CBM1 | Nd | 2 | 3 |
| 1466845 | CAZY | PL8_4 | 101 | 61 | 48 |

Nd: Not detected

**Supplementary Table S4**: Cytochrome P450 monooxygenase transcripts identified in transcriptome analyses

| Protein  ID | Base Mean Malt  ose | log2Fold Change PIN | padj  PIN | padjBonf PIN | log2Fold Change ASP | padj  ASP | padjBonf  ASP | KEGG  definition | KOG definition |
| --- | --- | --- | --- | --- | --- | --- | --- | --- | --- |
| 1461314 | 167 | -3097 | 3,12E-29 | 1,13E-26 | -3011 | 4,87E-21 | 2,80E-18 | Acting on paired donors, with incorporation or reduction of molecular oxygen | CYP4/CYP19/CYP26 |
| 1439865 | 679 | -2045 | 1,10E-13 | 1,32E-10 | -2040 | 4,21E-13 | 4,24E-10 |  | CYP3/CYP5/CYP6/CYP9 |
| 1361709 | 9807 | -1014 | 1,06E-05 | 3,36E-02 | -2013 | 2,53E-17 | 1,88E-14 |  | CYP4/CYP19/CYP26 |
| 1436408 | 403 | 0 | NA | NA | 1089 | 1,45E-13 | 1,42E-10 |  | CYP4/CYP19/CYP26 |
| 1370938 | 2065 | 1022 | 5,22E-14 | 6,04E-11 | 2018 | 2,83E-43 | 4,76E-41 |  | CYP4/CYP19/CYP26 |
| 1362757 | 2259 | 0 | NA | NA | 2021 | 5,04E-07 | 1,01E-03 |  | CYP4/CYP19/CYP26 |
| 1435960 | 3300 | 4026 | 6,63E-61 | 3,45E-59 | 3039 | 5,57E-39 | 1,14E-36 |  | CYP4/CYP19/CYP26 |
| 1362447 | 1654 | 6069 | 7,04E-56 | 4,93E-54 | 4006 | 3,70E-21 | 2,09E-18 |  | CYP4/CYP19/CYP26 |
| 1471371 | 6143 | 4003 | 6,40E-18 | 5,19E-15 | 4043 | 1,88E-21 | 1,03E-18 |  | CYP2 |
| 1439874 | 1631 | 2000 | 1,11E-07 | 2,62E-04 | 0 | NA | NA |  | CYP4/CYP19/CYP26 |
|  |  |  |  |  |  |  |  |  |  |
| 1436381 | 3119 | 1067 | 1,23E-14 | 1,35E-11 | 1033 | 1,58E-09 | 2,30E-06 | Alkane-1 mono  oxygenase | CYP3/CYP5/CYP6/CYP9 |
| 1411619 | 8667 | 1022 | 1,32E-05 | 4,25E-02 | 2026 | 1,32E-16 | 1,04E-13 |  | CYP4/CYP19/CYP26 |
|  |  |  |  |  |  |  |  |  |  |
|  |  |  |  |  |  |  |  | Flavonoid 3'-mono  oxygenase |  |
| 1449439 | 1046 | 3037 | 1,72E-20 | 1,11E-17 | 3093 | 1,87E-27 | 7,06E-25 |  | CYP2 |
|  |  |  |  |  |  |  |  |  |  |
| 1395204 | 521 | 0 | NA | NA | -2029 | 1,06E-14 | 9,49E-12 | Leukotriene-B4 20-mono  oxygenase | CYP4/CYP19/CYP26 |
| 1440969 | 3031 | -1091 | 2,39E-22 | 1,35E-19 | -2021 | 1,18E-29 | 4,00E-27 |  | CYP4/CYP19/CYP26 |
| 1445338 | 1320 | 0 | NA | NA | -88 | 3,16E-06 | 7,21E-03 |  | CYP4/CYP19/CYP26 |
| 1362558 | 15697 | 6055 | 2,88E-93 | 2,88E-92 | 4003 | 6,73E-36 | 1,63E-33 |  | CYP4/CYP19/CYP26 |
| 1297918 | 18928 | 7047 | 9,76E-76 | 2,63E-74 | 4064 | 8,80E-30 | 2,96E-27 |  | CYP4/CYP19/CYP26 |
| 1452417 | 230 | 1043 | 3,11E-06 | 9,09E-03 | 0 | NA | NA |  | CYP4/CYP19/CYP26 |
| 1451800 | 15153 | 1063 | 4,58E-07 | 1,19E-03 | 0 | NA | NA |  | CYP4/CYP19/CYP26 |
|  |  |  |  |  |  |  |  |  |  |
| 1414039 | 303 | -2019 | 2,83E-17 | 2,44E-14 | -2047 | 8,92E-22 | 4,81E-19 | Steroid 17-alpha-mono  oxygenase | CYP2 |
| 1370733 | 189 | -1041 | 1,27E-08 | 2,67E-05 | -1099 | 1,23E-15 | 1,03E-12 |  | CYP2 |
| 1467501 | 705 | 1043 | 3,19E-07 | 8,07E-04 | 2018 | 2,46E-15 | 2,11E-12 |  | CYP2 |
| 1416283 | 28135 | -1072 | 2,93E-11 | 4,60E-08 | 0 | NA | NA |  | CYP2 |
|  |  |  |  |  |  |  |  |  |  |
| 1461855 | 759 | -2062 | 1,77E-18 | 1,36E-15 | -3029 | 1,67E-28 | 6,05E-26 | Unspecific mono  oxygenase | CYP4/CYP19/CYP26 |
| 1464087 | 1182 | -1052 | 2,23E-06 | 6,40E-03 | -1086 | 6,53E-09 | 1,02E-05 |  | CYP2 |
| 1438803 | 1065 | -1048 | 4,87E-13 | 6,23E-10 | -1055 | 3,12E-14 | 2,90E-11 |  | CYP4/CYP19/CYP26 |
| 1368737 | 667 | -1075 | 2,09E-15 | 2,12E-12 | -1022 | 4,82E-08 | 8,37E-05 |  | CYP2 |
| 1468613 | 4079 | 0 | NA | NA | -1005 | 3,69E-06 | 8,51E-03 |  | CYP2 |
| 1357400 | 3279 | 0 | NA | NA | 1044 | 1,11E-06 | 2,38E-03 |  | CYP3/CYP5/CYP6/CYP9 |
| 1365256 | 789 | 1027 | 7,02E-06 | 2,16E-02 | 1047 | 2,15E-07 | 4,06E-04 |  | CYP2 |
| 1491467 | 818 | 0 | NA | NA | 1073 | 6,47E-10 | 8,98E-07 |  | CYP2 |
| 1469997 | 2080 | 1013 | 7,05E-06 | 2,17E-02 | 2009 | 1,23E-17 | 8,92E-15 |  | CYP2 |
| 1441038 | 1251 | 1065 | 4,15E-08 | 9,31E-05 | 2011 | 1,60E-12 | 1,72E-09 |  | CYP2 |
| 1382082 | 1636 | 2041 | 3,01E-21 | 1,84E-18 | 2059 | 2,31E-24 | 1,05E-21 |  | CYP2 |
| 1440415 | 2423 | 3017 | 2,38E-26 | 1,03E-23 | 2061 | 3,92E-18 | 2,73E-15 |  | CYP2 |
| 1434192 | 1444 | 2075 | 1,34E-25 | 6,15E-23 | 2067 | 3,29E-24 | 1,52E-21 |  | CYP2 |
| 1416824 | 11737 | 2085 | 2,90E-35 | 6,65E-33 | 2087 | 7,01E-36 | 1,70E-33 |  | CYP4/CYP19/CYP26 |
| 1369748 | 4284 | 2053 | 3,08E-16 | 2,88E-13 | 3014 | 1,75E-24 | 7,88E-22 |  | CYP4/CYP19/CYP26 |
| 1359011 | 1755 | 3074 | 5,54E-23 | 3,04E-20 | 3020 | 4,94E-17 | 3,75E-14 |  | CYP2 |
| 1465600 | 472 | 2092 | 3,63E-14 | 4,13E-11 | 3032 | 5,00E-18 | 3,53E-15 |  | CYP4/CYP19/CYP26 |
| 1469297 | 23817 | 2073 | 3,94E-20 | 2,63E-17 | 3046 | 1,36E-31 | 4,18E-29 |  | CYP2 |
| 1468151 | 21173 | 5000 | 2,27E-112 | 2,27E-112 | 4057 | 7,12E-95 | 1,14E-93 |  | CYP4/CYP19/CYP26 |
| 1463698 | 347 | 3084 | 1,66E-23 | 8,74E-21 | 4088 | 1,11E-37 | 2,42E-35 |  | CYP2 |
| 1381215 | 1091 | -2045 | 4,20E-16 | 4,00E-13 | 0 | NA | NA |  | CYP2 |
| 1458735 | 4278 | -1082 | 3,73E-11 | 5,93E-08 | 0 | NA | NA |  | CYP2 |
| 1410653 | 239 | -1065 | 3,36E-06 | 9,90E-03 | 0 | NA | NA |  | CYP2 |
| 1447291 | 15637 | -1031 | 4,12E-06 | 1,22E-02 | 0 | NA | NA |  | CYP2 |
| 1438806 | 1852 | -1027 | 9,12E-08 | 2,14E-04 | 0 | NA | NA |  | CYP4/CYP19/CYP26 |
| 1376156 | 2674 | 77 | 1,16E-07 | 2,75E-04 | 0 | NA | NA |  | CYP4/CYP19/CYP26 |
| 1500945 | 3109 | 1002 | 8,29E-08 | 1,93E-04 | 0 | NA | NA |  | CYP4/CYP19/CYP26 |
| 1362943 | 601 | 1060 | 7,23E-15 | 7,73E-12 | 0 | NA | NA |  | CYP2 |
| 1369315 | 1082 | 1071 | 5,42E-07 | 1,42E-03 | 0 | NA | NA |  | CYP2 |
| 1521754 | 3699 | 1074 | 9,96E-08 | 2,35E-04 | 0 | NA | NA |  | CYP2 |
| 1438328 | 8209 | 2076 | 6,58E-30 | 2,24E-27 | 0 | NA | NA |  | CYP4/CYP19/CYP26 |
|  |  |  |  |  |  |  |  |  |  |
|  |  |  |  |  |  |  |  | With  reduced flavin or  flavoprotein as one donor |  |
|  |  |  |  |  |  |  |  |  |  |
| 1366793 | 2779 | -1084 | 3,97E-25 | 1,89E-22 | -1075 | 6,54E-23 | 3,28E-20 |  | CYP2 |
|  |  |  |  |  |  |  |  |  |  |
|  |  |  |  |  |  |  |  |  |  |
| 1392735 | 97 | 2061 | 1,93E-10 | 3,29E-07 | 2059 | 2,72E-10 | 3,64E-07 | Other | CYP3/CYP5/CYP6/CYP9 |
| 1450696 | 423 | -1055 | 3,29E-08 | 7,28E-05 | -1052 | 6,26E-08 | 1,10E-04 |  | CYP4/CYP19/CYP26 |
| 1463074 | 3842 | 0 | NA | NA | -1006 | 3,52E-06 | 8,08E-03 |  | CYP3/CYP5/CYP6/CYP9 |
| 1468281 | 456 | 0 | NA | NA | -2007 | 1,23E-06 | 2,65E-03 |  | CYP11/CYP12/CYP24/CYP27 |
| 1468637 | 2042 | 0 | NA | NA | -1084 | 1,56E-09 | 2,26E-06 |  | . |

The table shows the mean number of reads for each gene in the maltose control condition and the fold change of transcript levels expressed as log2 on each substrate as compared to maltose.

padj: p value adjusted with the Benjamini-Hochberg method

padj_Bonf: p value adjusted with the Bonferroni method

NA: Not Applicable

**Supplementary Table** **5**: Transcripts encoding sequences of unknown function identified in transcriptome analyses

| Protein ID | Base  Mean Maltose | log2  Fold Change  PIN | padj_  PIN | padj_  Bonf_  PIN | log2  Fold Change  ASP | padj_  ASP | padj_  Bonf_  ASP | Peptide signal |
| --- | --- | --- | --- | --- | --- | --- | --- | --- |
| 1222227 | 29 | -6,32 | 2,18E-16 | 2,00E-13 | -5,11 | 7,32E-14 | 6,98E-11 | NO |
| 1441045 | 121 | -6,13 | 4,87E-31 | 1,47E-28 | -2,36 | 9,88E-08 | 1,78E-04 | NO |
| 1341371 | 595 | -5,57 | 8,05E-39 | 1,49E-36 | -6,8 | 8,18E-54 | 7,52E-52 | NO |
| 1414510 | 276 | -5,24 | 9,37E-47 | 1,12E-44 | -5,72 | 3,69E-54 | 3,24E-52 | NO |
| 1361970 | 2857 | -4,99 | 2,63E-80 | 5,25E-79 | -4,58 | 1,14E-68 | 5,60E-67 | NO |
| 1376903 | 864 | -4,99 | 5,60E-43 | 7,90E-41 | -5,28 | 4,41E-48 | 5,43E-46 | NO |
| 1463417 | 214 | -4,87 | 5,63E-28 | 2,19E-25 | -4,31 | 2,84E-23 | 1,40E-20 | NO |
| 1424503 | 2536 | -4,73 | 1,92E-22 | 1,08E-19 | -4,72 | 2,54E-22 | 1,32E-19 | NO |
| 1470278 | 60 | -4,67 | 1,47E-30 | 4,72E-28 | -3,24 | 2,72E-25 | 1,15E-22 | NO |
| 1447422 | 1019 | -4,48 | 2,94E-22 | 1,68E-19 | -2,52 | 8,76E-08 | 1,57E-04 | NO |
| 1432065 | 13814 | -4,41 | 1,01E-56 | 6,55E-55 | -4,15 | 1,66E-50 | 1,83E-48 | NO |
| 1480348 | 77 | -4,34 | 1,47E-20 | 9,53E-18 | -4,86 | 1,38E-24 | 6,17E-22 | NO |
| 1467522 | 1231 | -4,2 | 1,09E-20 | 6,99E-18 | -3,53 | 5,98E-15 | 5,25E-12 | NO |
| 1428636 | 196 | -4,17 | 9,08E-28 | 3,60E-25 | -5,29 | 1,48E-40 | 2,82E-38 | NO |
| 1434448 | 78 | -4,09 | 3,46E-08 | 7,68E-05 | 0 | NA | NA | NO |
| 1435277 | 146 | -4,06 | 2,51E-17 | 2,15E-14 | -4,17 | 2,56E-18 | 1,77E-15 | NO |
| 1430979 | 3938 | -4,04 | 5,27E-58 | 3,21E-56 | -3,62 | 3,46E-47 | 4,54E-45 | NO |
| 189492 | 996 | -3,96 | 1,77E-32 | 4,89E-30 | -3,46 | 2,58E-25 | 1,09E-22 | NO |
| 1417111 | 1164 | -3,89 | 3,95E-30 | 1,31E-27 | -2,03 | 3,95E-09 | 6,03E-06 | NO |
| 1401411 | 1292 | -3,86 | 2,10E-28 | 7,93E-26 | -4,03 | 6,26E-31 | 1,98E-28 | NO |
| 561077 | 59 | -3,82 | 9,37E-20 | 6,47E-17 | -3,58 | 7,24E-19 | 4,83E-16 | NO |
| 1433896 | 2664 | -3,82 | 1,42E-20 | 9,16E-18 | -3,47 | 3,33E-17 | 2,50E-14 | NO |
| 1435757 | 33 | -3,8 | 3,31E-17 | 2,87E-14 | 0 | NA | NA | NO |
| 1436991 | 5413 | -3,72 | 5,04E-48 | 5,49E-46 | -3,55 | 7,85E-44 | 1,28E-41 | NO |
| 1362788 | 563 | -3,66 | 6,25E-36 | 1,34E-33 | -3,33 | 9,93E-31 | 3,18E-28 | NO |
| 1437844 | 15 | -3,64 | 1,46E-07 | 3,50E-04 | -3,84 | 2,65E-08 | 4,44E-05 | NO |
| 211276 | 2548 | -3,6 | 4,67E-41 | 7,29E-39 | -2,64 | 8,43E-23 | 4,27E-20 | NO |
| 1429443 | 39 | -3,6 | 1,76E-16 | 1,61E-13 | -2,07 | 1,18E-08 | 1,90E-05 | NO |
| 1432525 | 264 | -3,59 | 1,75E-50 | 1,68E-48 | -2,9 | 6,75E-37 | 1,54E-34 | NO |
| 1222103 | 17 | -3,57 | 1,74E-07 | 4,20E-04 | 0 | NA | NA | NO |
| 1440097 | 5384 | -3,57 | 1,39E-32 | 3,84E-30 | -4,52 | 1,68E-51 | 1,79E-49 | NO |
| 1463966 | 950 | -3,56 | 2,54E-47 | 2,92E-45 | -3,08 | 1,23E-36 | 2,85E-34 | NO |
| 1423520 | 567 | -3,55 | 3,47E-28 | 1,33E-25 | -1,95 | 9,78E-10 | 1,38E-06 | NO |
| 1374716 | 410 | -3,54 | 2,47E-26 | 1,07E-23 | -3,3 | 2,25E-23 | 1,10E-20 | NO |
| 1475451 | 11154 | -3,51 | 8,06E-24 | 4,10E-21 | 0 | NA | NA | NO |
| 1523846 | 797 | -3,45 | 4,37E-26 | 1,93E-23 | -4,18 | 1,26E-37 | 2,74E-35 | NO |
| 566403 | 209 | -3,44 | 1,16E-12 | 1,55E-09 | -2,19 | 6,33E-06 | 1,52E-02 | NO |
| 1210310 | 60 | -3,43 | 2,54E-12 | 3,57E-09 | -4,42 | 2,73E-18 | 1,89E-15 | NO |
| 1374538 | 3373 | -3,43 | 7,37E-32 | 2,08E-29 | -2,83 | 4,09E-22 | 2,15E-19 | NO |
| 435231 | 373 | -3,4 | 2,74E-24 | 1,37E-21 | -3,54 | 2,45E-26 | 9,93E-24 | NO |
| 1479923 | 1838 | -3,3 | 1,25E-41 | 1,87E-39 | -2,44 | 1,57E-23 | 7,53E-21 | NO |
| 440257 | 412 | -3,27 | 5,20E-28 | 2,02E-25 | 0 | NA | NA | NO |
| 541681 | 7871 | -3,25 | 6,58E-26 | 2,95E-23 | -2,94 | 3,05E-21 | 1,72E-18 | NO |
| 1264708 | 9 | -3,23 | 1,47E-05 | 4,76E-02 | 0 | NA | NA | NO |
| 1432858 | 3720 | -3,21 | 3,17E-22 | 1,82E-19 | -3,27 | 4,17E-23 | 2,07E-20 | NO |
| 1441226 | 1083 | -3,2 | 1,32E-21 | 7,94E-19 | -3,1 | 1,87E-20 | 1,13E-17 | NO |
| 1468651 | 287 | -3,18 | 1,18E-30 | 3,75E-28 | -2,4 | 3,13E-19 | 2,04E-16 | NO |
| 1430154 | 340 | -3,14 | 9,12E-30 | 3,15E-27 | -2,18 | 7,99E-16 | 6,64E-13 | NO |
| 1436227 | 1846 | -3,14 | 3,80E-34 | 9,43E-32 | -1,65 | 3,29E-10 | 4,43E-07 | NO |
| 1180108 | 802 | -3,07 | 1,63E-17 | 1,37E-14 | 0 | NA | NA | NO |
| 1464316 | 29 | -3,01 | 2,62E-06 | 7,61E-03 | 0 | NA | NA | NO |
| 1365290 | 184 | -2,92 | 1,36E-11 | 2,06E-08 | 0 | NA | NA | NO |
| 1470760 | 101 | -2,91 | 7,01E-09 | 1,43E-05 | -2,52 | 5,71E-07 | 1,16E-03 | NO |
| 1390390 | 3268 | -2,9 | 3,38E-11 | 5,33E-08 | -3,98 | 4,71E-20 | 2,90E-17 | NO |
| 399366 | 1596 | -2,88 | 1,48E-22 | 8,26E-20 | -2,62 | 8,26E-19 | 5,54E-16 | NO |
| 1368050 | 266 | -2,88 | 6,98E-16 | 6,78E-13 | -1,78 | 7,69E-07 | 1,59E-03 | NO |
| 1468285 | 36 | -2,87 | 1,51E-10 | 2,54E-07 | -1,91 | 2,03E-06 | 4,51E-03 | NO |
| 318144 | 284 | -2,82 | 1,02E-09 | 1,89E-06 | -2,8 | 1,44E-09 | 2,09E-06 | NO |
| 1457492 | 262 | -2,76 | 8,56E-19 | 6,33E-16 | 0 | NA | NA | NO |
| 385776 | 3152 | -2,71 | 3,70E-23 | 2,00E-20 | -2,4 | 2,61E-18 | 1,81E-15 | NO |
| 1462054 | 415 | -2,71 | 1,23E-13 | 1,49E-10 | -1,95 | 1,45E-07 | 2,66E-04 | NO |
| 483919 | 21 | -2,7 | 6,15E-07 | 1,63E-03 | 0 | NA | NA | NO |
| 1463294 | 1461 | -2,69 | 4,28E-23 | 2,33E-20 | -1,9 | 3,95E-12 | 4,43E-09 | NO |
| 1481039 | 5666 | -2,64 | 6,77E-21 | 4,29E-18 | 0 | NA | NA | NO |
| 163179 | 57 | -2,62 | 1,47E-10 | 2,48E-07 | 0 | NA | NA | NO |
| 1430264 | 2353 | -2,61 | 8,08E-17 | 7,20E-14 | -1,71 | 9,65E-08 | 1,74E-04 | NO |
| 1405235 | 805 | -2,57 | 7,19E-25 | 3,49E-22 | -1,78 | 7,09E-13 | 7,33E-10 | NO |
| 1410289 | 1054 | -2,57 | 2,17E-35 | 4,87E-33 | -2,45 | 1,02E-32 | 2,92E-30 | NO |
| 1430053 | 3653 | -2,57 | 6,16E-13 | 7,98E-10 | -3,39 | 1,39E-21 | 7,57E-19 | NO |
| 1435318 | 586 | -2,57 | 2,14E-33 | 5,49E-31 | -1,83 | 4,16E-18 | 2,91E-15 | NO |
| 1431291 | 1025 | -2,56 | 5,02E-09 | 1,01E-05 | 0 | NA | NA | NO |
| 1432717 | 685 | -2,56 | 1,03E-12 | 1,38E-09 | 0 | NA | NA | NO |
| 1470346 | 401 | -2,56 | 2,63E-18 | 2,07E-15 | -2,3 | 3,42E-15 | 2,95E-12 | NO |
| 1337545 | 30 | -2,55 | 2,36E-06 | 6,78E-03 | -2,82 | 2,08E-07 | 3,91E-04 | NO |
| 1428395 | 531 | -2,55 | 1,41E-16 | 1,27E-13 | -2,02 | 8,34E-11 | 1,06E-07 | NO |
| 1462978 | 856 | -2,55 | 9,06E-22 | 5,37E-19 | -2,3 | 4,96E-18 | 3,50E-15 | NO |
| 1430975 | 359 | -2,53 | 1,42E-18 | 1,09E-15 | -2,69 | 7,23E-21 | 4,22E-18 | NO |
| 1375112 | 1143 | -2,51 | 9,66E-20 | 6,68E-17 | -2,08 | 5,07E-14 | 4,76E-11 | NO |
| 619779 | 195 | -2,49 | 3,42E-15 | 3,54E-12 | -2,13 | 1,20E-11 | 1,40E-08 | NO |
| 1382214 | 435 | -2,45 | 3,34E-21 | 2,06E-18 | -1,74 | 1,32E-11 | 1,56E-08 | NO |
| 1469662 | 164 | -2,42 | 1,01E-14 | 1,10E-11 | -2,34 | 3,55E-14 | 3,31E-11 | NO |
| 981922 | 867 | -2,41 | 4,15E-15 | 4,34E-12 | -1,71 | 3,88E-08 | 6,68E-05 | NO |
| 1370382 | 2253 | -2,41 | 4,98E-30 | 1,66E-27 | -1,58 | 1,29E-13 | 1,25E-10 | NO |
| 1378411 | 663 | -2,41 | 2,23E-12 | 3,10E-09 | -3,43 | 1,06E-23 | 5,02E-21 | NO |
| 1367138 | 172 | -2,4 | 1,32E-14 | 1,45E-11 | -2,13 | 5,99E-12 | 6,83E-09 | NO |
| 1374376 | 449 | -2,4 | 1,03E-17 | 8,47E-15 | -1,77 | 2,61E-10 | 3,48E-07 | NO |
| 1462675 | 802 | -2,4 | 1,18E-21 | 7,06E-19 | -1,39 | 4,18E-08 | 7,23E-05 | NO |
| 1359383 | 352 | -2,39 | 3,13E-10 | 5,46E-07 | -3,07 | 5,37E-16 | 4,40E-13 | NO |
| 1438529 | 631 | -2,38 | 3,38E-34 | 8,31E-32 | -0,98 | 3,46E-07 | 6,78E-04 | NO |
| 1472264 | 37 | -2,38 | 6,03E-07 | 1,59E-03 | -2,48 | 1,91E-07 | 3,56E-04 | NO |
| 162725 | 409 | -2,37 | 5,77E-09 | 1,17E-05 | 0 | NA | NA | NO |
| 1431718 | 724 | -2,37 | 6,13E-25 | 2,97E-22 | -1 | 1,48E-05 | 3,77E-02 | NO |
| 1455923 | 229 | -2,37 | 7,10E-16 | 6,91E-13 | 0 | NA | NA | NO |
| 567775 | 35 | -2,36 | 8,64E-06 | 2,69E-02 | 0 | NA | NA | NO |
| 1359188 | 1216 | -2,32 | 1,37E-09 | 2,57E-06 | -2,13 | 3,40E-08 | 5,82E-05 | NO |
| 1249038 | 242 | -2,31 | 1,10E-23 | 5,70E-21 | -2,74 | 1,52E-32 | 4,42E-30 | NO |
| 1436592 | 256 | -2,3 | 2,17E-07 | 5,35E-04 | -2,63 | 3,08E-09 | 4,64E-06 | NO |
| 1480739 | 335 | -2,3 | 6,55E-16 | 6,36E-13 | -1,51 | 1,24E-07 | 2,27E-04 | NO |
| 1452209 | 94 | -2,29 | 1,22E-15 | 1,21E-12 | -3,84 | 1,21E-33 | 3,29E-31 | NO |
| 1460517 | 19 | -2,28 | 8,50E-07 | 2,30E-03 | 0 | NA | NA | NO |
| 1469960 | 196 | -2,28 | 5,23E-13 | 6,73E-10 | -1,95 | 5,40E-10 | 7,46E-07 | NO |
| 321793 | 254 | -2,27 | 4,49E-21 | 2,80E-18 | -1,07 | 4,00E-06 | 9,26E-03 | NO |
| 1400991 | 1112 | -2,26 | 1,27E-12 | 1,71E-09 | -2,97 | 9,14E-21 | 5,36E-18 | NO |
| 1381703 | 35 | -2,25 | 9,56E-07 | 2,60E-03 | 0 | NA | NA | NO |
| 1358774 | 1506 | -2,24 | 3,79E-21 | 2,36E-18 | -1,95 | 2,38E-16 | 1,91E-13 | NO |
| 418968 | 826 | -2,23 | 1,14E-15 | 1,13E-12 | 0 | NA | NA | NO |
| 1411456 | 616 | -2,23 | 1,18E-20 | 7,53E-18 | -1,13 | 2,86E-06 | 6,49E-03 | NO |
| 513836 | 14513 | -2,22 | 1,36E-07 | 3,26E-04 | -3,52 | 2,75E-17 | 2,04E-14 | NO |
| 1470992 | 399 | -2,22 | 3,54E-18 | 2,81E-15 | -2,17 | 1,48E-17 | 1,08E-14 | NO |
| 981938 | 1970 | -2,21 | 6,77E-21 | 4,29E-18 | -1,55 | 7,13E-11 | 9,00E-08 | NO |
| 1435821 | 493 | -2,19 | 3,91E-14 | 4,48E-11 | -1,57 | 8,14E-08 | 1,45E-04 | NO |
| 1431344 | 585 | -2,18 | 6,93E-18 | 5,65E-15 | -1,28 | 5,54E-07 | 1,13E-03 | NO |
| 1433320 | 463 | -2,18 | 9,93E-10 | 1,83E-06 | 0 | NA | NA | NO |
| 1436748 | 2279 | -2,18 | 1,48E-18 | 1,13E-15 | -2,35 | 1,55E-21 | 8,49E-19 | NO |
| 1363918 | 436 | -2,17 | 2,52E-11 | 3,90E-08 | -2,31 | 1,09E-12 | 1,15E-09 | NO |
| 48078 | 782 | -2,16 | 3,50E-14 | 3,98E-11 | -3,4 | 7,67E-33 | 2,18E-30 | NO |
| 1442479 | 3449 | -2,16 | 5,16E-22 | 3,01E-19 | -1,28 | 2,58E-08 | 4,31E-05 | NO |
| 1439641 | 42 | -2,15 | 8,17E-07 | 2,21E-03 | -2,82 | 2,02E-10 | 2,67E-07 | NO |
| 807312 | 10640 | -2,14 | 1,28E-15 | 1,27E-12 | -1,78 | 5,60E-11 | 6,98E-08 | NO |
| 1468509 | 241 | -2,14 | 1,23E-09 | 2,28E-06 | 0 | NA | NA | NO |
| 647854 | 57 | -2,13 | 5,46E-11 | 8,76E-08 | -1,69 | 6,67E-08 | 1,18E-04 | NO |
| 1459275 | 627 | -2,13 | 4,12E-06 | 1,23E-02 | -4,59 | 5,67E-24 | 2,63E-21 | NO |
| 1478783 | 57 | -2,13 | 5,93E-07 | 1,57E-03 | 0 | NA | NA | NO |
| 1457407 | 37 | -2,12 | 1,03E-07 | 2,43E-04 | 0 | NA | NA | NO |
| 1431335 | 109 | -2,1 | 4,20E-12 | 6,02E-09 | -2,71 | 8,80E-19 | 5,93E-16 | NO |
| 549619 | 420 | -2,09 | 1,86E-15 | 1,89E-12 | 0 | NA | NA | NO |
| 1439409 | 1991 | -2,09 | 1,49E-07 | 3,58E-04 | -2,89 | 1,96E-13 | 1,94E-10 | NO |
| 1466308 | 140 | -2,09 | 1,91E-12 | 2,64E-09 | -2,06 | 2,47E-12 | 2,71E-09 | NO |
| 197409 | 154 | -2,07 | 6,33E-11 | 1,02E-07 | -2,13 | 1,58E-11 | 1,87E-08 | NO |
| 1443362 | 439 | -2,07 | 2,24E-14 | 2,51E-11 | 0 | NA | NA | NO |
| 1446955 | 281 | -2,07 | 1,01E-11 | 1,51E-08 | -2,56 | 3,35E-17 | 2,52E-14 | NO |
| 1375515 | 283 | -2,06 | 1,22E-08 | 2,56E-05 | -3,3 | 5,77E-20 | 3,57E-17 | NO |
| 1403347 | 972 | -2,06 | 6,13E-12 | 8,96E-09 | -1,72 | 1,53E-08 | 2,48E-05 | NO |
| 268846 | 80 | -2,05 | 1,05E-08 | 2,19E-05 | -2,37 | 4,25E-11 | 5,24E-08 | NO |
| 1335429 | 53 | -2,05 | 2,21E-08 | 4,78E-05 | -1,65 | 4,51E-06 | 1,05E-02 | NO |
| 640696 | 43 | -2,04 | 7,86E-08 | 1,82E-04 | -1,71 | 5,00E-06 | 1,18E-02 | NO |
| 765112 | 677 | -2,04 | 1,96E-07 | 4,80E-04 | 0 | NA | NA | NO |
| 1018863 | 72 | -2,03 | 5,27E-06 | 1,59E-02 | 0 | NA | NA | NO |
| 1440489 | 728 | -2,03 | 3,70E-14 | 4,23E-11 | -1,87 | 3,47E-12 | 3,85E-09 | NO |
| 1480083 | 107 | -2,02 | 2,24E-08 | 4,84E-05 | -2,08 | 8,32E-09 | 1,31E-05 | NO |
| 1434434 | 365 | -2,01 | 4,74E-17 | 4,15E-14 | -1,43 | 1,63E-09 | 2,37E-06 | NO |
| 1445997 | 39 | -2,01 | 1,35E-05 | 4,34E-02 | 0 | NA | NA | NO |
| 1475727 | 2087 | -2,01 | 8,45E-10 | 1,54E-06 | -2,09 | 1,90E-10 | 2,51E-07 | NO |
| 1380531 | 266 | -1,98 | 9,32E-11 | 1,53E-07 | -1,39 | 6,14E-06 | 1,47E-02 | NO |
| 1431285 | 215 | -1,98 | 1,94E-09 | 3,68E-06 | 0 | NA | NA | NO |
| 1463393 | 5005 | -1,97 | 1,03E-23 | 5,33E-21 | -1,28 | 1,70E-10 | 2,23E-07 | NO |
| 1434700 | 812 | -1,96 | 2,02E-18 | 1,57E-15 | -1,58 | 1,42E-12 | 1,52E-09 | NO |
| 1457490 | 215 | -1,96 | 2,74E-08 | 6,00E-05 | 0 | NA | NA | NO |
| 1451092 | 696 | -1,95 | 9,79E-20 | 6,79E-17 | -1,69 | 2,62E-15 | 2,25E-12 | NO |
| 1372907 | 164 | -1,94 | 9,55E-11 | 1,57E-07 | -1,32 | 1,11E-05 | 2,76E-02 | NO |
| 1437741 | 182 | -1,94 | 2,27E-09 | 4,35E-06 | 0 | NA | NA | NO |
| 1368558 | 468 | -1,93 | 4,85E-15 | 5,11E-12 | -1,65 | 2,17E-11 | 2,62E-08 | NO |
| 452973 | 2189 | -1,92 | 2,78E-06 | 8,08E-03 | 0 | NA | NA | NO |
| 485758 | 62 | -1,92 | 6,92E-06 | 2,13E-02 | 0 | NA | NA | NO |
| 1437551 | 113 | -1,92 | 7,24E-10 | 1,31E-06 | -3,02 | 2,72E-21 | 1,52E-18 | NO |
| 1358172 | 307 | -1,91 | 9,15E-23 | 5,08E-20 | -1,47 | 1,06E-14 | 9,47E-12 | NO |
| 1441057 | 213 | -1,91 | 3,54E-08 | 7,88E-05 | -1,53 | 1,23E-05 | 3,09E-02 | NO |
| 1478912 | 187 | -1,91 | 3,73E-08 | 8,34E-05 | -1,94 | 2,50E-08 | 4,18E-05 | NO |
| 1480766 | 185 | -1,91 | 2,56E-11 | 3,97E-08 | -1,97 | 5,33E-12 | 6,05E-09 | NO |
| 1360318 | 655 | -1,9 | 1,84E-12 | 2,53E-09 | 0 | NA | NA | NO |
| 876066 | 73 | -1,89 | 3,28E-06 | 9,63E-03 | 0 | NA | NA | NO |
| 1371805 | 244 | -1,89 | 2,80E-11 | 4,38E-08 | -1,3 | 4,85E-06 | 1,14E-02 | NO |
| 1431796 | 6217 | -1,89 | 5,11E-10 | 9,11E-07 | -2,5 | 1,21E-16 | 9,50E-14 | NO |
| 1446003 | 246 | -1,89 | 4,64E-15 | 4,87E-12 | 0 | NA | NA | NO |
| 149035 | 80 | -1,88 | 1,12E-08 | 2,34E-05 | 0 | NA | NA | NO |
| 240787 | 533 | -1,88 | 3,19E-16 | 2,99E-13 | -1 | 1,81E-05 | 4,68E-02 | NO |
| 1441729 | 161 | -1,87 | 7,98E-11 | 1,30E-07 | -1,81 | 3,07E-10 | 4,12E-07 | NO |
| 1443646 | 676 | -1,87 | 5,98E-06 | 1,82E-02 | 0 | NA | NA | NO |
| 1473314 | 182 | -1,87 | 1,28E-06 | 3,53E-03 | 0 | NA | NA | NO |
| 1436917 | 934 | -1,86 | 2,36E-12 | 3,29E-09 | -1,64 | 7,74E-10 | 1,08E-06 | NO |
| 1462788 | 1606 | -1,86 | 1,27E-19 | 8,84E-17 | -2,03 | 2,21E-23 | 1,08E-20 | NO |
| 1473577 | 2750 | -1,86 | 1,05E-10 | 1,75E-07 | 0 | NA | NA | NO |
| 1431454 | 7157 | -1,85 | 6,84E-13 | 8,95E-10 | -1,8 | 3,56E-12 | 3,97E-09 | NO |
| 1412935 | 156 | -1,84 | 7,12E-06 | 2,19E-02 | 0 | NA | NA | NO |
| 1434737 | 72 | -1,84 | 8,66E-09 | 1,79E-05 | -1,82 | 8,02E-09 | 1,26E-05 | NO |
| 1397057 | 99 | -1,83 | 8,42E-08 | 1,96E-04 | -1,53 | 5,32E-06 | 1,26E-02 | NO |
| 1477860 | 87 | -1,83 | 9,62E-06 | 3,02E-02 | 0 | NA | NA | NO |
| 1504512 | 110 | -1,83 | 1,05E-08 | 2,20E-05 | 0 | NA | NA | NO |
| 1188033 | 48 | -1,82 | 2,18E-06 | 6,22E-03 | 0 | NA | NA | NO |
| 1471905 | 210 | -1,82 | 2,18E-08 | 4,70E-05 | -1,87 | 8,42E-09 | 1,33E-05 | NO |
| 278121 | 77 | -1,8 | 2,84E-08 | 6,22E-05 | 0 | NA | NA | NO |
| 1364382 | 213 | -1,8 | 8,49E-09 | 1,75E-05 | 0 | NA | NA | NO |
| 231837 | 317 | -1,79 | 4,89E-11 | 7,84E-08 | -1,22 | 8,14E-06 | 1,99E-02 | NO |
| 1439226 | 210 | -1,79 | 1,70E-10 | 2,87E-07 | -1,3 | 3,50E-06 | 8,03E-03 | NO |
| 1440423 | 2842 | -1,79 | 9,82E-06 | 3,09E-02 | -3,73 | 3,05E-21 | 1,72E-18 | NO |
| 1441060 | 1589 | -1,79 | 5,18E-08 | 1,17E-04 | -2,17 | 3,15E-11 | 3,85E-08 | NO |
| 321738 | 626 | -1,78 | 1,37E-19 | 9,55E-17 | -0,93 | 2,75E-06 | 6,23E-03 | NO |
| 1398958 | 74 | -1,77 | 2,17E-08 | 4,67E-05 | -1,48 | 1,65E-06 | 3,63E-03 | NO |
| 1372719 | 119 | -1,76 | 2,47E-10 | 4,25E-07 | -1,34 | 1,18E-06 | 2,54E-03 | NO |
| 1430305 | 554 | -1,76 | 3,08E-13 | 3,89E-10 | -2,19 | 9,33E-20 | 5,86E-17 | NO |
| 1460785 | 1585 | -1,76 | 2,93E-15 | 3,02E-12 | -1,21 | 1,00E-07 | 1,81E-04 | NO |
| 1436134 | 119 | -1,74 | 4,16E-08 | 9,33E-05 | -1,9 | 1,52E-09 | 2,21E-06 | NO |
| 1481818 | 108 | -1,74 | 1,75E-07 | 4,23E-04 | 0 | NA | NA | NO |
| 1070559 | 562 | -1,73 | 4,14E-10 | 7,30E-07 | -1,76 | 1,99E-10 | 2,63E-07 | NO |
| 1463485 | 743 | -1,72 | 7,83E-11 | 1,28E-07 | 0 | NA | NA | NO |
| 1439083 | 5156 | -1,71 | 2,11E-21 | 1,28E-18 | -2,07 | 8,78E-31 | 2,79E-28 | NO |
| 1463065 | 81296 | -1,71 | 5,99E-10 | 1,08E-06 | -2,14 | 7,66E-15 | 6,77E-12 | NO |
| 1403042 | 620 | -1,7 | 5,76E-09 | 1,17E-05 | 0 | NA | NA | NO |
| 1041548 | 110 | -1,69 | 4,68E-08 | 1,05E-04 | -2,24 | 6,69E-13 | 6,90E-10 | NO |
| 1359167 | 5150 | -1,69 | 5,68E-14 | 6,59E-11 | -1,16 | 5,18E-07 | 1,04E-03 | NO |
| 1418592 | 245 | -1,69 | 1,01E-07 | 2,38E-04 | -1,6 | 5,26E-07 | 1,06E-03 | NO |
| 1476888 | 324 | -1,68 | 1,19E-10 | 1,99E-07 | 0 | NA | NA | NO |
| 1429859 | 283 | -1,67 | 7,75E-13 | 1,02E-09 | -1,08 | 3,43E-06 | 7,86E-03 | NO |
| 1430586 | 1267 | -1,67 | 4,94E-08 | 1,12E-04 | 0 | NA | NA | NO |
| 1439975 | 1117 | -1,67 | 9,47E-06 | 2,97E-02 | 0 | NA | NA | NO |
| 1433625 | 1138 | -1,66 | 2,90E-14 | 3,28E-11 | -1,59 | 4,93E-13 | 5,02E-10 | NO |
| 1456700 | 160 | -1,66 | 2,40E-09 | 4,63E-06 | -1,37 | 8,69E-07 | 1,82E-03 | NO |
| 1440730 | 3275 | -1,65 | 2,66E-18 | 2,09E-15 | -1,19 | 5,02E-10 | 6,92E-07 | NO |
| 1503536 | 1227 | -1,64 | 1,28E-16 | 1,16E-13 | 0 | NA | NA | NO |
| 1358328 | 139 | -1,63 | 1,03E-07 | 2,43E-04 | 0 | NA | NA | NO |
| 1370341 | 1341 | -1,63 | 1,77E-11 | 2,72E-08 | -1,61 | 4,53E-11 | 5,60E-08 | NO |
| 346217 | 12548 | -1,62 | 2,91E-08 | 6,39E-05 | -1,95 | 2,17E-11 | 2,61E-08 | NO |
| 1357282 | 60 | -1,62 | 4,02E-06 | 1,19E-02 | -1,61 | 3,62E-06 | 8,32E-03 | NO |
| 1477092 | 492 | -1,62 | 9,82E-16 | 9,66E-13 | 0 | NA | NA | NO |
| 1357992 | 2004 | -1,61 | 6,34E-13 | 8,24E-10 | -1,89 | 2,07E-17 | 1,52E-14 | NO |
| 1453506 | 1073 | -1,6 | 9,52E-11 | 1,56E-07 | 0 | NA | NA | NO |
| 1295418 | 943 | -1,59 | 5,94E-18 | 4,81E-15 | 0 | NA | NA | NO |
| 1439768 | 712 | -1,59 | 9,72E-09 | 2,02E-05 | 0 | NA | NA | NO |
| 1430743 | 290 | -1,58 | 5,53E-12 | 8,02E-09 | 0 | NA | NA | NO |
| 1429912 | 342 | -1,57 | 1,05E-11 | 1,57E-08 | -1,31 | 1,31E-08 | 2,11E-05 | NO |
| 433405 | 242 | -1,56 | 6,39E-08 | 1,46E-04 | -1,96 | 9,55E-12 | 1,11E-08 | NO |
| 1414665 | 198 | -1,56 | 1,20E-07 | 2,85E-04 | -1,5 | 3,59E-07 | 7,05E-04 | NO |
| 1412919 | 1393 | -1,55 | 2,37E-09 | 4,56E-06 | 0 | NA | NA | NO |
| 338436 | 154 | -1,54 | 5,29E-07 | 1,39E-03 | 0 | NA | NA | NO |
| 1434616 | 1322 | -1,54 | 1,55E-09 | 2,90E-06 | -1,96 | 1,28E-14 | 1,16E-11 | NO |
| 1481598 | 287 | -1,54 | 1,11E-06 | 3,04E-03 | 0 | NA | NA | NO |
| 1374323 | 173 | -1,53 | 1,74E-08 | 3,72E-05 | -1,66 | 8,80E-10 | 1,24E-06 | NO |
| 1383607 | 1118 | -1,53 | 1,13E-14 | 1,24E-11 | 0 | NA | NA | NO |
| 1468085 | 1442 | -1,53 | 2,49E-10 | 4,29E-07 | 0 | NA | NA | NO |
| 1436982 | 683 | -1,52 | 2,60E-09 | 5,05E-06 | 0 | NA | NA | NO |
| 1477492 | 1644 | -1,51 | 7,46E-10 | 1,36E-06 | 0 | NA | NA | NO |
| 1443107 | 398 | -1,5 | 6,04E-12 | 8,82E-09 | 0 | NA | NA | NO |
| 24361 | 271 | -1,49 | 7,87E-11 | 1,28E-07 | -1,29 | 1,74E-08 | 2,87E-05 | NO |
| 1457322 | 356 | -1,49 | 9,46E-09 | 1,96E-05 | 0 | NA | NA | NO |
| 442848 | 163 | -1,48 | 9,01E-07 | 2,45E-03 | 0 | NA | NA | NO |
| 1477933 | 2451 | -1,48 | 3,94E-07 | 1,01E-03 | 0 | NA | NA | NO |
| 1389139 | 925 | -1,47 | 1,15E-06 | 3,17E-03 | -1,96 | 6,20E-11 | 7,76E-08 | NO |
| 1031721 | 579 | -1,46 | 3,33E-08 | 7,37E-05 | 0 | NA | NA | NO |
| 1435174 | 280 | -1,46 | 4,40E-08 | 9,90E-05 | -1,42 | 1,01E-07 | 1,83E-04 | NO |
| 1465192 | 136 | -1,46 | 3,16E-07 | 7,97E-04 | 0 | NA | NA | NO |
| 1375398 | 488 | -1,45 | 1,02E-09 | 1,87E-06 | -1,34 | 1,56E-08 | 2,54E-05 | NO |
| 1466113 | 1142 | -1,45 | 5,99E-07 | 1,58E-03 | 0 | NA | NA | NO |
| 412716 | 434 | -1,44 | 3,64E-07 | 9,29E-04 | -1,45 | 3,46E-07 | 6,79E-04 | NO |
| 1480977 | 82 | -1,44 | 6,49E-06 | 1,99E-02 | 0 | NA | NA | NO |
| 1463594 | 2073 | -1,42 | 2,49E-13 | 3,10E-10 | -1,14 | 7,93E-09 | 1,24E-05 | NO |
| 1421463 | 193 | -1,41 | 5,64E-07 | 1,48E-03 | 0 | NA | NA | NO |
| 1437179 | 1126 | -1,41 | 2,96E-09 | 5,78E-06 | 0 | NA | NA | NO |
| 1441972 | 575 | -1,41 | 9,89E-11 | 1,63E-07 | -1,03 | 3,51E-06 | 8,07E-03 | NO |
| 1477809 | 156 | -1,41 | 2,50E-06 | 7,23E-03 | -1,51 | 4,45E-07 | 8,91E-04 | NO |
| 1469213 | 462 | -1,4 | 3,83E-10 | 6,73E-07 | -1,43 | 1,52E-10 | 1,98E-07 | NO |
| 488893 | 277 | -1,39 | 5,52E-06 | 1,67E-02 | 0 | NA | NA | NO |
| 1436799 | 273 | -1,39 | 7,08E-06 | 2,18E-02 | 0 | NA | NA | NO |
| 1403249 | 293 | -1,38 | 7,23E-10 | 1,31E-06 | 0 | NA | NA | NO |
| 818323 | 250 | -1,37 | 2,19E-06 | 6,25E-03 | 0 | NA | NA | NO |
| 1364819 | 1011 | -1,37 | 1,48E-06 | 4,12E-03 | 0 | NA | NA | NO |
| 1395600 | 1128 | -1,37 | 5,34E-08 | 1,21E-04 | 0 | NA | NA | NO |
| 777391 | 1467 | -1,36 | 8,23E-07 | 2,22E-03 | -1,23 | 1,14E-05 | 2,85E-02 | NO |
| 1431632 | 3224 | -1,36 | 5,14E-06 | 1,55E-02 | 0 | NA | NA | NO |
| 1442923 | 80 | -1,36 | 2,20E-07 | 5,42E-04 | -1,59 | 1,24E-09 | 1,78E-06 | NO |
| 1479097 | 88 | -1,36 | 3,42E-06 | 1,01E-02 | 0 | NA | NA | NO |
| 328980 | 351 | -1,35 | 3,48E-09 | 6,83E-06 | -1,37 | 1,98E-09 | 2,90E-06 | NO |
| 1370428 | 123 | -1,35 | 5,73E-06 | 1,74E-02 | -1,86 | 4,47E-10 | 6,13E-07 | NO |
| 1391215 | 687 | -1,35 | 4,00E-09 | 7,89E-06 | 0 | NA | NA | NO |
| 1438154 | 630 | -1,35 | 1,16E-09 | 2,15E-06 | -1,25 | 2,12E-08 | 3,51E-05 | NO |
| 1464625 | 577 | -1,35 | 1,09E-10 | 1,80E-07 | -1,1 | 1,50E-07 | 2,75E-04 | NO |
| 1435834 | 687 | -1,33 | 5,47E-08 | 1,24E-04 | 0 | NA | NA | NO |
| 1470725 | 110 | -1,33 | 6,09E-06 | 1,86E-02 | 0 | NA | NA | NO |
| 1434940 | 4092 | -1,32 | 4,08E-09 | 8,05E-06 | 0 | NA | NA | NO |
| 1431635 | 2675 | -1,31 | 8,65E-06 | 2,70E-02 | 0 | NA | NA | NO |
| 1477197 | 239 | -1,31 | 9,94E-07 | 2,71E-03 | 0 | NA | NA | NO |
| 1481498 | 151 | -1,31 | 1,81E-06 | 5,10E-03 | 0 | NA | NA | NO |
| 1360821 | 256 | -1,3 | 4,13E-07 | 1,06E-03 | 0 | NA | NA | NO |
| 1441272 | 298 | -1,3 | 6,89E-08 | 1,58E-04 | 0 | NA | NA | NO |
| 1474173 | 126 | -1,3 | 8,45E-06 | 2,63E-02 | 0 | NA | NA | NO |
| 1281224 | 1155 | -1,29 | 3,70E-09 | 7,27E-06 | -1,97 | 1,19E-19 | 7,56E-17 | NO |
| 1376299 | 144 | -1,29 | 2,13E-07 | 5,25E-04 | -1,15 | 3,08E-06 | 7,02E-03 | NO |
| 1434665 | 897 | -1,29 | 1,27E-06 | 3,53E-03 | 0 | NA | NA | NO |
| 1435695 | 1265 | -1,29 | 1,46E-05 | 4,74E-02 | 0 | NA | NA | NO |
| 1439832 | 1501 | -1,28 | 1,16E-09 | 2,16E-06 | 0 | NA | NA | NO |
| 1462277 | 294 | -1,28 | 5,82E-07 | 1,53E-03 | 0 | NA | NA | NO |
| 1426209 | 2499 | -1,27 | 1,44E-10 | 2,41E-07 | 0 | NA | NA | NO |
| 1467179 | 550 | -1,27 | 5,92E-09 | 1,20E-05 | -0,99 | 7,06E-06 | 1,71E-02 | NO |
| 1438628 | 382 | -1,26 | 7,58E-08 | 1,75E-04 | 0 | NA | NA | NO |
| 1470452 | 1382 | -1,26 | 5,31E-08 | 1,20E-04 | 0 | NA | NA | NO |
| 1470744 | 480 | -1,26 | 4,55E-06 | 1,36E-02 | 0 | NA | NA | NO |
| 1476002 | 2030 | -1,26 | 8,76E-09 | 1,81E-05 | 0 | NA | NA | NO |
| 1432596 | 987 | -1,25 | 7,80E-07 | 2,10E-03 | 0 | NA | NA | NO |
| 1497769 | 2798 | -1,25 | 3,37E-07 | 8,55E-04 | -1,64 | 1,53E-11 | 1,81E-08 | NO |
| 1433187 | 330 | -1,24 | 1,51E-05 | 4,91E-02 | 0 | NA | NA | NO |
| 1465711 | 147 | -1,24 | 1,94E-06 | 5,52E-03 | -1,38 | 1,08E-07 | 1,96E-04 | NO |
| 1346982 | 1102 | -1,23 | 1,43E-05 | 4,63E-02 | -2,09 | 5,69E-14 | 5,38E-11 | NO |
| 1366050 | 4460 | -1,23 | 1,49E-06 | 4,15E-03 | 0 | NA | NA | NO |
| 1374292 | 410 | -1,23 | 8,96E-11 | 1,47E-07 | -1,59 | 2,48E-17 | 1,84E-14 | NO |
| 676226 | 944 | -1,22 | 1,61E-07 | 3,88E-04 | 0 | NA | NA | NO |
| 1415728 | 334 | -1,22 | 3,16E-06 | 9,24E-03 | 0 | NA | NA | NO |
| 1385164 | 887 | -1,21 | 6,79E-07 | 1,81E-03 | -1,18 | 1,34E-06 | 2,90E-03 | NO |
| 1472514 | 2411 | -1,21 | 6,88E-06 | 2,11E-02 | 0 | NA | NA | NO |
| 1474663 | 325 | -1,21 | 5,72E-06 | 1,74E-02 | -1,38 | 2,39E-07 | 4,57E-04 | NO |
| 949919 | 1838 | -1,2 | 1,41E-12 | 1,92E-09 | 0 | NA | NA | NO |
| 222003 | 2887 | -1,19 | 3,09E-07 | 7,78E-04 | 0 | NA | NA | NO |
| 1451538 | 634 | -1,19 | 1,91E-08 | 4,10E-05 | 0 | NA | NA | NO |
| 1464354 | 1236 | -1,19 | 3,60E-11 | 5,71E-08 | -1,18 | 5,37E-11 | 6,68E-08 | NO |
| 1451710 | 1046 | -1,18 | 4,36E-06 | 1,31E-02 | 0 | NA | NA | NO |
| 1430423 | 908 | -1,17 | 9,68E-07 | 2,64E-03 | 0 | NA | NA | NO |
| 1433422 | 3908 | -1,17 | 2,13E-07 | 5,23E-04 | -1,33 | 4,07E-09 | 6,22E-06 | NO |
| 1457489 | 340 | -1,17 | 5,09E-07 | 1,33E-03 | 0 | NA | NA | NO |
| 1479701 | 376 | -1,17 | 1,60E-06 | 4,50E-03 | 0 | NA | NA | NO |
| 1447286 | 600 | -1,16 | 1,02E-09 | 1,89E-06 | 0 | NA | NA | NO |
| 1459218 | 334 | -1,16 | 1,36E-05 | 4,38E-02 | 0 | NA | NA | NO |
| 1480944 | 1158 | -1,16 | 2,13E-06 | 6,09E-03 | 0 | NA | NA | NO |
| 1438832 | 1788 | -1,14 | 5,75E-09 | 1,16E-05 | 0 | NA | NA | NO |
| 1190763 | 345 | -1,13 | 4,21E-06 | 1,26E-02 | 0 | NA | NA | NO |
| 1389700 | 1020 | -1,11 | 1,63E-06 | 4,57E-03 | 0 | NA | NA | NO |
| 1423837 | 722 | -1,1 | 4,26E-07 | 1,10E-03 | 0 | NA | NA | NO |
| 1498418 | 1085 | -1,09 | 1,26E-09 | 2,35E-06 | 0 | NA | NA | NO |
| 1422348 | 324 | -1,08 | 4,18E-06 | 1,25E-02 | -1,5 | 1,48E-10 | 1,93E-07 | NO |
| 1436364 | 792 | -1,08 | 3,56E-06 | 1,05E-02 | 0 | NA | NA | NO |
| 1437253 | 2403 | -1,08 | 3,41E-07 | 8,65E-04 | 0 | NA | NA | NO |
| 1375274 | 897 | -1,07 | 8,66E-07 | 2,35E-03 | 0 | NA | NA | NO |
| 1438090 | 666 | -1,07 | 1,82E-07 | 4,41E-04 | 0 | NA | NA | NO |
| 1476230 | 188 | -1,07 | 1,47E-05 | 4,76E-02 | 0 | NA | NA | NO |
| 1370060 | 1086 | -1,06 | 4,96E-06 | 1,49E-02 | 0 | NA | NA | NO |
| 1478383 | 1331 | -1,06 | 8,71E-06 | 2,72E-02 | 0 | NA | NA | NO |
| 193879 | 392 | -1,05 | 2,34E-07 | 5,80E-04 | 0 | NA | NA | NO |
| 1372578 | 607 | -1,05 | 9,87E-06 | 3,10E-02 | 0 | NA | NA | NO |
| 1433231 | 277 | -1,05 | 2,26E-06 | 6,47E-03 | 0 | NA | NA | NO |
| 1432214 | 855 | -1,04 | 5,21E-13 | 6,70E-10 | -0,65 | 9,78E-06 | 2,43E-02 | NO |
| 1465743 | 840 | -1,02 | 4,91E-06 | 1,48E-02 | 0 | NA | NA | NO |
| 1447241 | 511 | -1,01 | 1,29E-05 | 4,15E-02 | 0 | NA | NA | NO |
| 1470405 | 480 | -1,01 | 1,29E-05 | 4,15E-02 | 0 | NA | NA | NO |
| 1406138 | 696 | -1 | 6,95E-06 | 2,14E-02 | 0 | NA | NA | NO |
| 1461394 | 2021 | -1 | 4,22E-10 | 7,45E-07 | -1,04 | 1,37E-10 | 1,78E-07 | NO |
| 512351 | 729 | -0,99 | 2,16E-06 | 6,18E-03 | 0 | NA | NA | NO |
| 1365884 | 961 | -0,99 | 7,16E-07 | 1,91E-03 | 0 | NA | NA | NO |
| 1467335 | 432 | -0,98 | 8,81E-07 | 2,39E-03 | 0 | NA | NA | NO |
| 1366782 | 809 | -0,97 | 1,45E-06 | 4,04E-03 | 0 | NA | NA | NO |
| 1432337 | 1055 | -0,97 | 2,66E-08 | 5,82E-05 | 0 | NA | NA | NO |
| 1436994 | 2915 | -0,97 | 1,37E-05 | 4,42E-02 | 0 | NA | NA | NO |
| 1380522 | 472 | -0,96 | 2,32E-06 | 6,68E-03 | 0 | NA | NA | NO |
| 1380454 | 570 | -0,95 | 7,55E-07 | 2,03E-03 | 0 | NA | NA | NO |
| 1436961 | 3456 | -0,93 | 5,75E-07 | 1,52E-03 | 0 | NA | NA | NO |
| 1474610 | 732 | -0,91 | 5,27E-09 | 1,06E-05 | 0 | NA | NA | NO |
| 1430643 | 3930 | -0,88 | 1,85E-06 | 5,25E-03 | -1,12 | 1,26E-09 | 1,80E-06 | NO |
| 1431029 | 668 | -0,88 | 1,41E-05 | 4,55E-02 | 0 | NA | NA | NO |
| 1375154 | 315 | -0,87 | 4,93E-06 | 1,49E-02 | 0 | NA | NA | NO |
| 1430690 | 1551 | -0,86 | 4,88E-07 | 1,27E-03 | 0 | NA | NA | NO |
| 1373365 | 1814 | -0,82 | 6,94E-07 | 1,85E-03 | 0 | NA | NA | NO |
| 1474523 | 539 | -0,73 | 1,52E-05 | 4,95E-02 | -1,48 | 7,45E-19 | 4,97E-16 | NO |
| 488 | 150 | 0 | NA | NA | 3,13 | 7,76E-28 | 2,87E-25 | NO |
| 1179 | 180 | 0 | NA | NA | 2,68 | 1,12E-11 | 1,31E-08 | NO |
| 5915 | 274 | 0 | NA | NA | 1,15 | 7,49E-07 | 1,55E-03 | NO |
| 6212 | 84 | 0 | NA | NA | 0 | NA | NA | NO |
| 9896 | 861 | 0 | NA | NA | 0 | NA | NA | NO |
| 10717 | 2747 | 0 | NA | NA | 0 | NA | NA | NO |
| 12060 | 416 | 0 | NA | NA | 0 | NA | NA | NO |
| 19974 | 65 | 0 | NA | NA | 0 | NA | NA | NO |
| 20328 | 382 | 0 | NA | NA | -1,64 | 1,78E-05 | 4,60E-02 | NO |
| 21294 | 158 | 0 | NA | NA | 0 | NA | NA | NO |
| 36469 | 2240 | 0 | NA | NA | 2,63 | 2,21E-33 | 6,10E-31 | NO |
| 47881 | 83 | 0 | NA | NA | 0 | NA | NA | NO |
| 49822 | 133 | 0 | NA | NA | 0 | NA | NA | NO |
| 54395 | 171 | 0 | NA | NA | 0 | NA | NA | NO |
| 72588 | 11128 | 0 | NA | NA | 0 | NA | NA | NO |
| 80106 | 4664 | 0 | NA | NA | 2,96 | 1,63E-06 | 3,57E-03 | NO |
| 81103 | 166 | 0 | NA | NA | 0 | NA | NA | NO |
| 81588 | 1092 | 0 | NA | NA | 0 | NA | NA | NO |
| 86408 | 930 | 0 | NA | NA | -1,6 | 1,52E-08 | 2,46E-05 | NO |
| 91585 | 864 | 0 | NA | NA | -1,7 | 1,70E-05 | 4,39E-02 | NO |
| 101466 | 88 | 0 | NA | NA | 0 | NA | NA | NO |
| 101548 | 451 | 0 | NA | NA | 0 | NA | NA | NO |
| 109533 | 3021 | 0 | NA | NA | 0 | NA | NA | NO |
| 120170 | 1110 | 0 | NA | NA | 1,06 | 6,41E-06 | 1,54E-02 | NO |
| 124693 | 811 | 0 | NA | NA | 1,15 | 1,60E-08 | 2,61E-05 | NO |
| 131752 | 349 | 0 | NA | NA | 0 | NA | NA | NO |
| 132439 | 390 | 0 | NA | NA | 0 | NA | NA | NO |
| 133726 | 401 | 0 | NA | NA | 0 | NA | NA | NO |
| 137321 | 22815 | 0 | NA | NA | 0 | NA | NA | NO |
| 155427 | 46 | 0 | NA | NA | 3,13 | 2,23E-15 | 1,91E-12 | NO |
| 158305 | 225 | 0 | NA | NA | -1,39 | 1,37E-05 | 3,47E-02 | NO |
| 167459 | 170 | 0 | NA | NA | 0 | NA | NA | NO |
| 170159 | 8 | 0 | NA | NA | 0 | NA | NA | NO |
| 188248 | 10 | 0 | NA | NA | 0 | NA | NA | NO |
| 191138 | 37 | 0 | NA | NA | 0 | NA | NA | NO |
| 193010 | 72 | 0 | NA | NA | 0 | NA | NA | NO |
| 196323 | 460 | 0 | NA | NA | 0 | NA | NA | NO |
| 196673 | 172 | 0 | NA | NA | 0 | NA | NA | NO |
| 198830 | 1117 | 0 | NA | NA | 0 | NA | NA | NO |
| 209243 | 83 | 0 | NA | NA | 0 | NA | NA | NO |
| 219015 | 909 | 0 | NA | NA | 0 | NA | NA | NO |
| 228056 | 12 | 0 | NA | NA | 0 | NA | NA | NO |
| 231190 | 2118 | 0 | NA | NA | 0 | NA | NA | NO |
| 232115 | 8941 | 0 | NA | NA | 0 | NA | NA | NO |
| 250473 | 145 | 0 | NA | NA | 0 | NA | NA | NO |
| 250594 | 1145 | 0 | NA | NA | 0 | NA | NA | NO |
| 255025 | 41 | 0 | NA | NA | -2,39 | 3,50E-06 | 8,02E-03 | NO |
| 258643 | 822 | 0 | NA | NA | 0 | NA | NA | NO |
| 262497 | 154 | 0 | NA | NA | 0 | NA | NA | NO |
| 279582 | 60 | 0 | NA | NA | 0 | NA | NA | NO |
| 284287 | 3319 | 0 | NA | NA | 0 | NA | NA | NO |
| 286874 | 139 | 0 | NA | NA | 0 | NA | NA | NO |
| 296345 | 57 | 0 | NA | NA | 0 | NA | NA | NO |
| 297791 | 1019 | 0 | NA | NA | -2,11 | 2,01E-07 | 3,76E-04 | NO |
| 301271 | 115 | 0 | NA | NA | 0 | NA | NA | NO |
| 322392 | 266 | 0 | NA | NA | 0 | NA | NA | NO |
| 329316 | 1348 | 0 | NA | NA | 1,78 | 6,49E-11 | 8,16E-08 | NO |
| 332252 | 59 | 0 | NA | NA | 0 | NA | NA | NO |
| 332344 | 16 | 0 | NA | NA | 2,48 | 1,36E-05 | 3,45E-02 | NO |
| 334678 | 174 | 0 | NA | NA | 1,69 | 3,49E-06 | 8,00E-03 | NO |
| 341714 | 39 | 0 | NA | NA | -2,14 | 1,08E-06 | 2,30E-03 | NO |
| 341717 | 302 | 0 | NA | NA | 0 | NA | NA | NO |
| 341824 | 531 | 0 | NA | NA | 0 | NA | NA | NO |
| 359480 | 2146 | 0 | NA | NA | 1,15 | 1,11E-10 | 1,44E-07 | NO |
| 361283 | 37 | 0 | NA | NA | 2,48 | 3,79E-07 | 7,49E-04 | NO |
| 362395 | 194 | 0 | NA | NA | 0 | NA | NA | NO |
| 364389 | 202 | 0 | NA | NA | 1,52 | 1,28E-05 | 3,23E-02 | NO |
| 376818 | 213 | 0 | NA | NA | 1,25 | 4,76E-06 | 1,11E-02 | NO |
| 379405 | 331 | 0 | NA | NA | 1,45 | 1,44E-07 | 2,65E-04 | NO |
| 403342 | 291 | 0 | NA | NA | 0 | NA | NA | NO |
| 405195 | 77 | 0 | NA | NA | 2,08 | 6,50E-06 | 1,57E-02 | NO |
| 416651 | 125 | 0 | NA | NA | 0 | NA | NA | NO |
| 422790 | 306 | 0 | NA | NA | 0 | NA | NA | NO |
| 427028 | 1028 | 0 | NA | NA | -2,1 | 4,98E-10 | 6,85E-07 | NO |
| 435626 | 676 | 0 | NA | NA | 0 | NA | NA | NO |
| 437154 | 70 | 0 | NA | NA | 0 | NA | NA | NO |
| 442841 | 170 | 0 | NA | NA | 0 | NA | NA | NO |
| 443604 | 332 | 0 | NA | NA | 0 | NA | NA | NO |
| 443607 | 322 | 0 | NA | NA | 0 | NA | NA | NO |
| 444299 | 54 | 0 | NA | NA | 0 | NA | NA | NO |
| 450210 | 71 | 0 | NA | NA | 0 | NA | NA | NO |
| 454485 | 4305 | 0 | NA | NA | 0 | NA | NA | NO |
| 457386 | 41 | 0 | NA | NA | 0 | NA | NA | NO |
| 460225 | 29 | 0 | NA | NA | 0 | NA | NA | NO |
| 466847 | 100 | 0 | NA | NA | 1,95 | 3,36E-08 | 5,74E-05 | NO |
| 470656 | 4348 | 0 | NA | NA | 0 | NA | NA | NO |
| 483491 | 632 | 0 | NA | NA | 1,64 | 3,21E-07 | 6,26E-04 | NO |
| 487197 | 278 | 0 | NA | NA | 0 | NA | NA | NO |
| 488562 | 30 | 0 | NA | NA | 2,07 | 6,44E-06 | 1,55E-02 | NO |
| 523253 | 503 | 0 | NA | NA | 0 | NA | NA | NO |
| 538365 | 288 | 0 | NA | NA | 0 | NA | NA | NO |
| 539082 | 777 | 0 | NA | NA | 0 | NA | NA | NO |
| 570784 | 814 | 0 | NA | NA | 0 | NA | NA | NO |
| 571327 | 268 | 0 | NA | NA | 0 | NA | NA | NO |
| 585117 | 21 | 0 | NA | NA | 0 | NA | NA | NO |
| 590243 | 23 | 0 | NA | NA | 0 | NA | NA | NO |
| 591415 | 773 | 0 | NA | NA | 0 | NA | NA | NO |
| 593782 | 510 | 0 | NA | NA | 0 | NA | NA | NO |
| 595659 | 24997 | 0 | NA | NA | 0 | NA | NA | NO |
| 606685 | 41 | 0 | NA | NA | 0 | NA | NA | NO |
| 607232 | 279 | 0 | NA | NA | 0 | NA | NA | NO |
| 608987 | 183 | 0 | NA | NA | -1,91 | 4,08E-09 | 6,25E-06 | NO |
| 622449 | 1597 | 0 | NA | NA | 0 | NA | NA | NO |
| 630071 | 30 | 0 | NA | NA | 2 | 1,69E-06 | 3,72E-03 | NO |
| 650177 | 38 | 0 | NA | NA | 0 | NA | NA | NO |
| 652639 | 12 | 0 | NA | NA | 0 | NA | NA | NO |
| 660580 | 38 | 0 | NA | NA | 0 | NA | NA | NO |
| 667066 | 769 | 0 | NA | NA | 0 | NA | NA | NO |
| 668056 | 57 | 0 | NA | NA | -3,15 | 2,74E-09 | 4,09E-06 | NO |
| 673575 | 254 | 0 | NA | NA | 0 | NA | NA | NO |
| 676381 | 117 | 0 | NA | NA | 0 | NA | NA | NO |
| 680985 | 24 | 0 | NA | NA | 2,04 | 1,67E-05 | 4,31E-02 | NO |
| 685156 | 12 | 0 | NA | NA | 4,27 | 9,33E-09 | 1,48E-05 | NO |
| 699482 | 99 | 0 | NA | NA | 0 | NA | NA | NO |
| 702515 | 576 | 0 | NA | NA | 1,58 | 1,51E-05 | 3,84E-02 | NO |
| 707523 | 56 | 0 | NA | NA | 2,56 | 1,92E-11 | 2,29E-08 | NO |
| 713083 | 1553 | 0 | NA | NA | -1,72 | 3,91E-08 | 6,73E-05 | NO |
| 713636 | 134 | 0 | NA | NA | 0 | NA | NA | NO |
| 720025 | 349 | 0 | NA | NA | 1,62 | 1,51E-06 | 3,29E-03 | NO |
| 722556 | 219 | 0 | NA | NA | 1,1 | 4,72E-06 | 1,11E-02 | NO |
| 727059 | 727 | 0 | NA | NA | -2,81 | 1,45E-14 | 1,32E-11 | NO |
| 739269 | 60225 | 0 | NA | NA | 2,88 | 2,85E-18 | 1,98E-15 | NO |
| 739950 | 120 | 0 | NA | NA | 2,04 | 4,01E-09 | 6,12E-06 | NO |
| 751083 | 919 | 0 | NA | NA | 0 | NA | NA | NO |
| 751872 | 22 | 0 | NA | NA | 2,59 | 1,45E-05 | 3,70E-02 | NO |
| 753688 | 95 | 0 | NA | NA | -1,94 | 2,46E-06 | 5,53E-03 | NO |
| 754402 | 783 | 0 | NA | NA | 0 | NA | NA | NO |
| 761052 | 13 | 0 | NA | NA | 3,53 | 8,15E-06 | 1,99E-02 | NO |
| 768232 | 442 | 0 | NA | NA | -1,59 | 2,36E-07 | 4,50E-04 | NO |
| 775303 | 8 | 0 | NA | NA | 0 | NA | NA | NO |
| 780957 | 298 | 0 | NA | NA | 0 | NA | NA | NO |
| 796996 | 14 | 0 | NA | NA | 0 | NA | NA | NO |
| 799268 | 195 | 0 | NA | NA | 0 | NA | NA | NO |
| 801178 | 78 | 0 | NA | NA | -3,18 | 3,09E-09 | 4,65E-06 | NO |
| 809452 | 2747 | 0 | NA | NA | 0 | NA | NA | NO |
| 814162 | 32 | 0 | NA | NA | 0 | NA | NA | NO |
| 822787 | 266 | 0 | NA | NA | -1,58 | 8,45E-09 | 1,33E-05 | NO |
| 825480 | 3068 | 0 | NA | NA | 0 | NA | NA | NO |
| 854268 | 111 | 0 | NA | NA | 0 | NA | NA | NO |
| 858455 | 16 | 0 | NA | NA | 0 | NA | NA | NO |
| 880318 | 1272 | 0 | NA | NA | 0 | NA | NA | NO |
| 902896 | 2817 | 0 | NA | NA | 0 | NA | NA | NO |
| 909511 | 52 | 0 | NA | NA | 0 | NA | NA | NO |
| 911753 | 79 | 0 | NA | NA | 0 | NA | NA | NO |
| 912758 | 420 | 0 | NA | NA | 0 | NA | NA | NO |
| 916620 | 224 | 0 | NA | NA | 0 | NA | NA | NO |
| 919404 | 336 | 0 | NA | NA | 0 | NA | NA | NO |
| 925081 | 7797 | 0 | NA | NA | -1,43 | 4,20E-06 | 9,76E-03 | NO |
| 930530 | 4479 | 0 | NA | NA | 0 | NA | NA | NO |
| 931207 | 1026 | 0 | NA | NA | 0 | NA | NA | NO |
| 932036 | 60 | 0 | NA | NA | 0 | NA | NA | NO |
| 932114 | 79 | 0 | NA | NA | 0 | NA | NA | NO |
| 933344 | 20 | 0 | NA | NA | 0 | NA | NA | NO |
| 941137 | 21 | 0 | NA | NA | 0 | NA | NA | NO |
| 942896 | 43 | 0 | NA | NA | 0 | NA | NA | NO |
| 946704 | 132 | 0 | NA | NA | 0 | NA | NA | NO |
| 965602 | 6958 | 0 | NA | NA | 0 | NA | NA | NO |
| 966636 | 150 | 0 | NA | NA | 0 | NA | NA | NO |
| 970171 | 145 | 0 | NA | NA | 0 | NA | NA | NO |
| 977562 | 534 | 0 | NA | NA | 0 | NA | NA | NO |
| 980407 | 171 | 0 | NA | NA | 0 | NA | NA | NO |
| 995372 | 963 | 0 | NA | NA | 0 | NA | NA | NO |
| 997507 | 52 | 0 | NA | NA | 1,76 | 2,11E-06 | 4,70E-03 | NO |
| 1000119 | 2469 | 0 | NA | NA | 0 | NA | NA | NO |
| 1011314 | 68 | 0 | NA | NA | 0 | NA | NA | NO |
| 1029870 | 104 | 0 | NA | NA | 0 | NA | NA | NO |
| 1030733 | 210 | 0 | NA | NA | 0 | NA | NA | NO |
| 1042690 | 143 | 0 | NA | NA | 0 | NA | NA | NO |
| 1043154 | 181 | 0 | NA | NA | 0 | NA | NA | NO |
| 1043881 | 491 | 0 | NA | NA | 0 | NA | NA | NO |
| 1045278 | 85 | 0 | NA | NA | 0 | NA | NA | NO |
| 1046284 | 314 | 0 | NA | NA | 2,16 | 9,31E-22 | 5,04E-19 | NO |
| 1047383 | 1448 | 0 | NA | NA | 0 | NA | NA | NO |
| 1048600 | 151 | 0 | NA | NA | 0 | NA | NA | NO |
| 1051351 | 2079 | 0 | NA | NA | 1,34 | 2,22E-06 | 4,95E-03 | NO |
| 1052569 | 1858 | 0 | NA | NA | 1,46 | 2,29E-08 | 3,81E-05 | NO |
| 1056594 | 51 | 0 | NA | NA | 0 | NA | NA | NO |
| 1068979 | 2314 | 0 | NA | NA | 0 | NA | NA | NO |
| 1073040 | 97 | 0 | NA | NA | 0 | NA | NA | NO |
| 1078736 | 357 | 0 | NA | NA | 0 | NA | NA | NO |
| 1099672 | 7 | 0 | NA | NA | 0 | NA | NA | NO |
| 1104163 | 68 | 0 | NA | NA | 0 | NA | NA | NO |
| 1106993 | 513 | 0 | NA | NA | 0 | NA | NA | NO |
| 1113951 | 451 | 0 | NA | NA | -1,52 | 2,78E-09 | 4,16E-06 | NO |
| 1114086 | 297 | 0 | NA | NA | 0 | NA | NA | NO |
| 1117331 | 28 | 0 | NA | NA | 0 | NA | NA | NO |
| 1132117 | 21 | 0 | NA | NA | 0 | NA | NA | NO |
| 1152470 | 131 | 0 | NA | NA | 0 | NA | NA | NO |
| 1152802 | 303 | 0 | NA | NA | 0 | NA | NA | NO |
| 1156018 | 119 | 0 | NA | NA | 0 | NA | NA | NO |
| 1158325 | 78 | 0 | NA | NA | 0 | NA | NA | NO |
| 1159362 | 4057 | 0 | NA | NA | 0 | NA | NA | NO |
| 1184334 | 242 | 0 | NA | NA | 0 | NA | NA | NO |
| 1184835 | 29 | 0 | NA | NA | 0 | NA | NA | NO |
| 1185280 | 177 | 0 | NA | NA | 0 | NA | NA | NO |
| 1186661 | 911 | 0 | NA | NA | -1,7 | 7,84E-07 | 1,63E-03 | NO |
| 1200763 | 344 | 0 | NA | NA | 4,96 | 5,59E-34 | 1,50E-31 | NO |
| 1200795 | 84 | 0 | NA | NA | 2,88 | 1,02E-09 | 1,44E-06 | NO |
| 1200854 | 12 | 0 | NA | NA | 4,46 | 5,71E-09 | 8,81E-06 | NO |
| 1200868 | 137 | 0 | NA | NA | 3,52 | 4,28E-15 | 3,71E-12 | NO |
| 1201707 | 173 | 0 | NA | NA | 0 | NA | NA | NO |
| 1202869 | 910 | 0 | NA | NA | -1,61 | 1,70E-06 | 3,73E-03 | NO |
| 1204215 | 12 | 0 | NA | NA | 2,75 | 1,89E-05 | 4,91E-02 | NO |
| 1205437 | 92 | 0 | NA | NA | 0 | NA | NA | NO |
| 1211999 | 3233 | 0 | NA | NA | 0 | NA | NA | NO |
| 1221981 | 3006 | 0 | NA | NA | 0 | NA | NA | NO |
| 1223147 | 77 | 0 | NA | NA | 0 | NA | NA | NO |
| 1226524 | 476 | 0 | NA | NA | 0 | NA | NA | NO |
| 1237604 | 817 | 0 | NA | NA | 0 | NA | NA | NO |
| 1239000 | 55 | 0 | NA | NA | 0 | NA | NA | NO |
| 1240988 | 50 | 0 | NA | NA | 0 | NA | NA | NO |
| 1245050 | 101 | 0 | NA | NA | 0 | NA | NA | NO |
| 1261290 | 270 | 0 | NA | NA | 1,77 | 6,00E-07 | 1,23E-03 | NO |
| 1262223 | 1029 | 0 | NA | NA | 0 | NA | NA | NO |
| 1266067 | 271 | 0 | NA | NA | 0 | NA | NA | NO |
| 1270507 | 21 | 0 | NA | NA | 0 | NA | NA | NO |
| 1272028 | 114 | 0 | NA | NA | 0 | NA | NA | NO |
| 1273227 | 87 | 0 | NA | NA | 0 | NA | NA | NO |
| 1274104 | 451 | 0 | NA | NA | 0 | NA | NA | NO |
| 1275885 | 183 | 0 | NA | NA | 1,79 | 8,48E-07 | 1,77E-03 | NO |
| 1282071 | 268 | 0 | NA | NA | 0 | NA | NA | NO |
| 1282084 | 223 | 0 | NA | NA | 2,63 | 6,71E-11 | 8,46E-08 | NO |
| 1295039 | 26 | 0 | NA | NA | 0 | NA | NA | NO |
| 1305999 | 126 | 0 | NA | NA | 0 | NA | NA | NO |
| 1308853 | 953 | 0 | NA | NA | 0 | NA | NA | NO |
| 1331641 | 49 | 0 | NA | NA | 0 | NA | NA | NO |
| 1342325 | 1243 | 0 | NA | NA | 0 | NA | NA | NO |
| 1345039 | 109 | 0 | NA | NA | 0 | NA | NA | NO |
| 1345877 | 10 | 0 | NA | NA | 0 | NA | NA | NO |
| 1348507 | 790 | 0 | NA | NA | 1,45 | 1,49E-10 | 1,93E-07 | NO |
| 1356454 | 64 | 0 | NA | NA | 0 | NA | NA | NO |
| 1356917 | 847 | 0 | NA | NA | 1,39 | 2,10E-11 | 2,52E-08 | NO |
| 1356973 | 378 | 0 | NA | NA | 0 | NA | NA | NO |
| 1357249 | 354 | 0 | NA | NA | 0 | NA | NA | NO |
| 1357264 | 39592 | 0 | NA | NA | 0 | NA | NA | NO |
| 1357319 | 217 | 0 | NA | NA | -1,6 | 3,36E-07 | 6,57E-04 | NO |
| 1357714 | 122 | 0 | NA | NA | 0 | NA | NA | NO |
| 1357785 | 75 | 0 | NA | NA | 0 | NA | NA | NO |
| 1358138 | 3667 | 0 | NA | NA | 0 | NA | NA | NO |
| 1358292 | 200 | 0 | NA | NA | 0 | NA | NA | NO |
| 1358542 | 1698 | 0 | NA | NA | 0 | NA | NA | NO |
| 1358764 | 78 | 0 | NA | NA | 1,99 | 8,22E-11 | 1,04E-07 | NO |
| 1358933 | 77521 | 0 | NA | NA | 0 | NA | NA | NO |
| 1359095 | 305 | 0 | NA | NA | 0 | NA | NA | NO |
| 1359120 | 1732 | 0 | NA | NA | 0 | NA | NA | NO |
| 1359140 | 788 | 0 | NA | NA | 0 | NA | NA | NO |
| 1359171 | 1366 | 0 | NA | NA | 0 | NA | NA | NO |
| 1359659 | 757 | 0 | NA | NA | 0 | NA | NA | NO |
| 1359830 | 1199 | 0 | NA | NA | -1,64 | 1,01E-10 | 1,30E-07 | NO |
| 1360223 | 4216 | 0 | NA | NA | 0 | NA | NA | NO |
| 1360687 | 127 | 0 | NA | NA | 1,37 | 1,20E-06 | 2,58E-03 | NO |
| 1361023 | 1300 | 0 | NA | NA | 0,99 | 4,20E-07 | 8,35E-04 | NO |
| 1361837 | 696 | 0 | NA | NA | 0 | NA | NA | NO |
| 1363009 | 292 | 0 | NA | NA | 1,5 | 1,16E-06 | 2,50E-03 | NO |
| 1363175 | 2032 | 0 | NA | NA | 1,33 | 2,74E-09 | 4,10E-06 | NO |
| 1363805 | 68 | 0 | NA | NA | 0 | NA | NA | NO |
| 1364179 | 41206 | 0 | NA | NA | 1,26 | 5,21E-06 | 1,23E-02 | NO |
| 1365279 | 4771 | 0 | NA | NA | 0 | NA | NA | NO |
| 1365956 | 236 | 0 | NA | NA | 0 | NA | NA | NO |
| 1366421 | 358 | 0 | NA | NA | -1,93 | 2,25E-11 | 2,72E-08 | NO |
| 1367075 | 123 | 0 | NA | NA | 0 | NA | NA | NO |
| 1367359 | 566 | 0 | NA | NA | 0,79 | 1,13E-06 | 2,43E-03 | NO |
| 1367390 | 133 | 0 | NA | NA | 1,85 | 1,62E-06 | 3,55E-03 | NO |
| 1367966 | 59 | 0 | NA | NA | 0 | NA | NA | NO |
| 1368301 | 80 | 0 | NA | NA | 0 | NA | NA | NO |
| 1368764 | 1768 | 0 | NA | NA | -1,72 | 5,98E-09 | 9,26E-06 | NO |
| 1369193 | 9311 | 0 | NA | NA | 1,15 | 1,90E-05 | 4,94E-02 | NO |
| 1369239 | 166 | 0 | NA | NA | 0 | NA | NA | NO |
| 1369246 | 207 | 0 | NA | NA | 0 | NA | NA | NO |
| 1369557 | 192 | 0 | NA | NA | -1,44 | 1,22E-06 | 2,63E-03 | NO |
| 1370077 | 258 | 0 | NA | NA | 1,67 | 1,20E-05 | 3,01E-02 | NO |
| 1370098 | 142 | 0 | NA | NA | 1,65 | 4,84E-08 | 8,41E-05 | NO |
| 1370099 | 208 | 0 | NA | NA | 0 | NA | NA | NO |
| 1370246 | 3995 | 0 | NA | NA | 0 | NA | NA | NO |
| 1370753 | 80 | 0 | NA | NA | 0 | NA | NA | NO |
| 1371322 | 29 | 0 | NA | NA | 2,64 | 3,93E-06 | 9,11E-03 | NO |
| 1371332 | 3438 | 0 | NA | NA | 0 | NA | NA | NO |
| 1371407 | 20 | 0 | NA | NA | 0 | NA | NA | NO |
| 1371414 | 30113 | 0 | NA | NA | 0 | NA | NA | NO |
| 1372024 | 415 | 0 | NA | NA | 0 | NA | NA | NO |
| 1372360 | 10876 | 0 | NA | NA | 0 | NA | NA | NO |
| 1372559 | 919 | 0 | NA | NA | 0,87 | 4,70E-07 | 9,44E-04 | NO |
| 1372634 | 172 | 0 | NA | NA | -1,31 | 1,33E-05 | 3,36E-02 | NO |
| 1372804 | 4004 | 0 | NA | NA | 0 | NA | NA | NO |
| 1372935 | 465 | 0 | NA | NA | 0 | NA | NA | NO |
| 1373022 | 2181 | 0 | NA | NA | -1,42 | 1,52E-07 | 2,80E-04 | NO |
| 1373263 | 3116 | 0 | NA | NA | 0 | NA | NA | NO |
| 1374804 | 1385 | 0 | NA | NA | 2,12 | 1,31E-15 | 1,10E-12 | NO |
| 1374908 | 13168 | 0 | NA | NA | 0 | NA | NA | NO |
| 1374922 | 930 | 0 | NA | NA | 0 | NA | NA | NO |
| 1374981 | 226 | 0 | NA | NA | 0 | NA | NA | NO |
| 1374999 | 70 | 0 | NA | NA | 0 | NA | NA | NO |
| 1375041 | 114 | 0 | NA | NA | 1,54 | 1,06E-06 | 2,26E-03 | NO |
| 1375186 | 1109 | 0 | NA | NA | -1,17 | 6,12E-06 | 1,46E-02 | NO |
| 1375933 | 1232 | 0 | NA | NA | 1,26 | 1,42E-06 | 3,09E-03 | NO |
| 1375936 | 256 | 0 | NA | NA | 0 | NA | NA | NO |
| 1375938 | 47 | 0 | NA | NA | 0 | NA | NA | NO |
| 1375939 | 62 | 0 | NA | NA | 0 | NA | NA | NO |
| 1376414 | 192 | 0 | NA | NA | 0 | NA | NA | NO |
| 1376527 | 2023 | 0 | NA | NA | 0 | NA | NA | NO |
| 1376848 | 236 | 0 | NA | NA | -1,25 | 8,77E-06 | 2,16E-02 | NO |
| 1377044 | 537 | 0 | NA | NA | 0 | NA | NA | NO |
| 1377155 | 1360 | 0 | NA | NA | 0 | NA | NA | NO |
| 1377204 | 774 | 0 | NA | NA | -1,61 | 2,71E-08 | 4,56E-05 | NO |
| 1377732 | 10 | 0 | NA | NA | 0 | NA | NA | NO |
| 1377780 | 557 | 0 | NA | NA | 1,28 | 1,13E-06 | 2,42E-03 | NO |
| 1378294 | 153 | 0 | NA | NA | 0 | NA | NA | NO |
| 1378711 | 410 | 0 | NA | NA | 0 | NA | NA | NO |
| 1378839 | 546 | 0 | NA | NA | 0 | NA | NA | NO |
| 1379168 | 10793 | 0 | NA | NA | 0 | NA | NA | NO |
| 1379428 | 34 | 0 | NA | NA | 0 | NA | NA | NO |
| 1379582 | 11 | 0 | NA | NA | 0 | NA | NA | NO |
| 1379845 | 615 | 0 | NA | NA | 0 | NA | NA | NO |
| 1379924 | 127 | 0 | NA | NA | 0 | NA | NA | NO |
| 1381171 | 104 | 0 | NA | NA | 0 | NA | NA | NO |
| 1383454 | 3925 | 0 | NA | NA | 0 | NA | NA | NO |
| 1383807 | 343 | 0 | NA | NA | 0 | NA | NA | NO |
| 1384679 | 428 | 0 | NA | NA | 0 | NA | NA | NO |
| 1385632 | 7009 | 0 | NA | NA | 0 | NA | NA | NO |
| 1386804 | 704 | 0 | NA | NA | 0 | NA | NA | NO |
| 1388180 | 264 | 0 | NA | NA | 0 | NA | NA | NO |
| 1388525 | 2593 | 0 | NA | NA | 0 | NA | NA | NO |
| 1389573 | 2299 | 0 | NA | NA | 0 | NA | NA | NO |
| 1390155 | 395 | 0 | NA | NA | -2,1 | 8,18E-14 | 7,85E-11 | NO |
| 1390470 | 1200 | 0 | NA | NA | 5,06 | 1,26E-37 | 2,76E-35 | NO |
| 1390693 | 592 | 0 | NA | NA | -1,42 | 4,01E-06 | 9,30E-03 | NO |
| 1390992 | 799 | 0 | NA | NA | 0 | NA | NA | NO |
| 1391245 | 1009 | 0 | NA | NA | 0 | NA | NA | NO |
| 1391468 | 30 | 0 | NA | NA | 0 | NA | NA | NO |
| 1391801 | 10545 | 0 | NA | NA | 0 | NA | NA | NO |
| 1392422 | 222 | 0 | NA | NA | 0 | NA | NA | NO |
| 1393781 | 210 | 0 | NA | NA | 0 | NA | NA | NO |
| 1393929 | 388 | 0 | NA | NA | 0 | NA | NA | NO |
| 1394283 | 741 | 0 | NA | NA | 1,01 | 1,90E-08 | 3,13E-05 | NO |
| 1394319 | 1684 | 0 | NA | NA | 0,97 | 3,15E-09 | 4,74E-06 | NO |
| 1394822 | 30 | 0 | NA | NA | 0 | NA | NA | NO |
| 1395007 | 4479 | 0 | NA | NA | 0 | NA | NA | NO |
| 1395633 | 5467 | 0 | NA | NA | 0 | NA | NA | NO |
| 1395651 | 3676 | 0 | NA | NA | 0 | NA | NA | NO |
| 1395830 | 2129 | 0 | NA | NA | 0 | NA | NA | NO |
| 1396225 | 805 | 0 | NA | NA | 0 | NA | NA | NO |
| 1396916 | 223 | 0 | NA | NA | 1,1 | 1,57E-05 | 4,03E-02 | NO |
| 1397681 | 95 | 0 | NA | NA | 1,5 | 1,10E-06 | 2,34E-03 | NO |
| 1398369 | 220 | 0 | NA | NA | 0 | NA | NA | NO |
| 1398622 | 808 | 0 | NA | NA | 0 | NA | NA | NO |
| 1398761 | 3863 | 0 | NA | NA | 0 | NA | NA | NO |
| 1398812 | 66 | 0 | NA | NA | 0 | NA | NA | NO |
| 1399071 | 95 | 0 | NA | NA | 0 | NA | NA | NO |
| 1399090 | 512 | 0 | NA | NA | 0 | NA | NA | NO |
| 1399312 | 716 | 0 | NA | NA | 0 | NA | NA | NO |
| 1399794 | 428 | 0 | NA | NA | 0 | NA | NA | NO |
| 1400065 | 357 | 0 | NA | NA | -2,31 | 4,02E-07 | 7,98E-04 | NO |
| 1400230 | 2110 | 0 | NA | NA | -1,87 | 6,98E-08 | 1,24E-04 | NO |
| 1400290 | 1014 | 0 | NA | NA | 0 | NA | NA | NO |
| 1400544 | 174 | 0 | NA | NA | -2,67 | 1,11E-08 | 1,77E-05 | NO |
| 1400633 | 515 | 0 | NA | NA | 0 | NA | NA | NO |
| 1401846 | 6672 | 0 | NA | NA | 0 | NA | NA | NO |
| 1403500 | 1156 | 0 | NA | NA | 1,56 | 1,36E-07 | 2,48E-04 | NO |
| 1405060 | 330 | 0 | NA | NA | 0 | NA | NA | NO |
| 1406362 | 994 | 0 | NA | NA | 0 | NA | NA | NO |
| 1406719 | 115 | 0 | NA | NA | 0 | NA | NA | NO |
| 1407031 | 249 | 0 | NA | NA | 0 | NA | NA | NO |
| 1407879 | 1610 | 0 | NA | NA | 0 | NA | NA | NO |
| 1407918 | 31 | 0 | NA | NA | -2,38 | 1,03E-06 | 2,19E-03 | NO |
| 1408364 | 35 | 0 | NA | NA | 0 | NA | NA | NO |
| 1408481 | 59 | 0 | NA | NA | 0 | NA | NA | NO |
| 1408651 | 1274 | 0 | NA | NA | -1,2 | 3,44E-07 | 6,73E-04 | NO |
| 1408751 | 197 | 0 | NA | NA | 1,3 | 6,25E-06 | 1,50E-02 | NO |
| 1408813 | 172 | 0 | NA | NA | 0 | NA | NA | NO |
| 1409861 | 1667 | 0 | NA | NA | 0 | NA | NA | NO |
| 1410633 | 373 | 0 | NA | NA | 1,11 | 1,95E-10 | 2,58E-07 | NO |
| 1411227 | 2950 | 0 | NA | NA | 0 | NA | NA | NO |
| 1411299 | 214 | 0 | NA | NA | 1,41 | 6,05E-06 | 1,44E-02 | NO |
| 1411407 | 947 | 0 | NA | NA | 0 | NA | NA | NO |
| 1411717 | 186 | 0 | NA | NA | 0 | NA | NA | NO |
| 1411793 | 935 | 0 | NA | NA | 1,06 | 2,31E-09 | 3,42E-06 | NO |
| 1412441 | 356 | 0 | NA | NA | 0 | NA | NA | NO |
| 1412519 | 488 | 0 | NA | NA | 0 | NA | NA | NO |
| 1412807 | 1345 | 0 | NA | NA | 0 | NA | NA | NO |
| 1413591 | 1695 | 0 | NA | NA | 0 | NA | NA | NO |
| 1413611 | 707 | 0 | NA | NA | 0 | NA | NA | NO |
| 1413967 | 6061 | 0 | NA | NA | 0 | NA | NA | NO |
| 1414436 | 11 | 0 | NA | NA | 0 | NA | NA | NO |
| 1414559 | 140 | 0 | NA | NA | 0 | NA | NA | NO |
| 1414661 | 377 | 0 | NA | NA | 0 | NA | NA | NO |
| 1414712 | 50 | 0 | NA | NA | 0 | NA | NA | NO |
| 1414884 | 315 | 0 | NA | NA | 1,3 | 1,86E-06 | 4,11E-03 | NO |
| 1415288 | 798 | 0 | NA | NA | -0,98 | 6,42E-06 | 1,54E-02 | NO |
| 1415512 | 707 | 0 | NA | NA | -1,53 | 3,36E-06 | 7,67E-03 | NO |
| 1415617 | 1397 | 0 | NA | NA | 0 | NA | NA | NO |
| 1416287 | 21353 | 0 | NA | NA | 0 | NA | NA | NO |
| 1416620 | 3928 | 0 | NA | NA | 0 | NA | NA | NO |
| 1417139 | 381 | 0 | NA | NA | 0,97 | 2,12E-09 | 3,13E-06 | NO |
| 1417209 | 7754 | 0 | NA | NA | 1,43 | 1,84E-05 | 4,76E-02 | NO |
| 1417511 | 510 | 0 | NA | NA | 0 | NA | NA | NO |
| 1417614 | 4654 | 0 | NA | NA | 0 | NA | NA | NO |
| 1417948 | 241 | 0 | NA | NA | 0 | NA | NA | NO |
| 1418355 | 842 | 0 | NA | NA | 0 | NA | NA | NO |
| 1418389 | 434 | 0 | NA | NA | 0 | NA | NA | NO |
| 1418603 | 275 | 0 | NA | NA | -2,05 | 2,75E-15 | 2,37E-12 | NO |
| 1418726 | 98 | 0 | NA | NA | 1,79 | 1,68E-07 | 3,10E-04 | NO |
| 1419298 | 113 | 0 | NA | NA | 0 | NA | NA | NO |
| 1420299 | 1842 | 0 | NA | NA | 0 | NA | NA | NO |
| 1420552 | 314 | 0 | NA | NA | -1,15 | 2,63E-06 | 5,94E-03 | NO |
| 1421247 | 1012 | 0 | NA | NA | 0 | NA | NA | NO |
| 1421701 | 7 | 0 | NA | NA | 0 | NA | NA | NO |
| 1421748 | 1371 | 0 | NA | NA | 0 | NA | NA | NO |
| 1422249 | 534 | 0 | NA | NA | 0 | NA | NA | NO |
| 1422691 | 208 | 0 | NA | NA | 0 | NA | NA | NO |
| 1423145 | 301 | 0 | NA | NA | 0 | NA | NA | NO |
| 1424881 | 939 | 0 | NA | NA | 1,25 | 1,84E-05 | 4,77E-02 | NO |
| 1425670 | 23 | 0 | NA | NA | -2,32 | 5,21E-06 | 1,23E-02 | NO |
| 1425724 | 115 | 0 | NA | NA | 1,49 | 1,11E-06 | 2,36E-03 | NO |
| 1426149 | 4081 | 0 | NA | NA | 0 | NA | NA | NO |
| 1426557 | 377 | 0 | NA | NA | 2,81 | 1,36E-19 | 8,69E-17 | NO |
| 1426955 | 354 | 0 | NA | NA | 1,15 | 4,18E-06 | 9,71E-03 | NO |
| 1427223 | 2482 | 0 | NA | NA | 0 | NA | NA | NO |
| 1427422 | 568 | 0 | NA | NA | -1,66 | 4,03E-07 | 8,00E-04 | NO |
| 1427431 | 53126 | 0 | NA | NA | 0 | NA | NA | NO |
| 1427566 | 23 | 0 | NA | NA | 0 | NA | NA | NO |
| 1427617 | 1349 | 0 | NA | NA | 0 | NA | NA | NO |
| 1427905 | 1982 | 0 | NA | NA | 0 | NA | NA | NO |
| 1427927 | 476 | 0 | NA | NA | 0 | NA | NA | NO |
| 1428562 | 194 | 0 | NA | NA | 0 | NA | NA | NO |
| 1428807 | 3207 | 0 | NA | NA | 0 | NA | NA | NO |
| 1429339 | 230 | 0 | NA | NA | 0 | NA | NA | NO |
| 1429341 | 356 | 0 | NA | NA | 1,5 | 1,95E-07 | 3,64E-04 | NO |
| 1429391 | 90 | 0 | NA | NA | 2,38 | 1,55E-05 | 3,97E-02 | NO |
| 1429403 | 51 | 0 | NA | NA | 2,9 | 6,25E-10 | 8,67E-07 | NO |
| 1429522 | 2525 | 0 | NA | NA | -1,03 | 5,54E-06 | 1,31E-02 | NO |
| 1429533 | 181 | 0 | NA | NA | 1,73 | 5,54E-06 | 1,31E-02 | NO |
| 1429600 | 2413 | 0 | NA | NA | 0 | NA | NA | NO |
| 1429644 | 2675 | 0 | NA | NA | 0 | NA | NA | NO |
| 1429704 | 2842 | 0 | NA | NA | 0 | NA | NA | NO |
| 1429950 | 1257 | 0 | NA | NA | 0 | NA | NA | NO |
| 1430130 | 601 | 0 | NA | NA | 0 | NA | NA | NO |
| 1430150 | 20189 | 0 | NA | NA | 0 | NA | NA | NO |
| 1430164 | 9213 | 0 | NA | NA | 0 | NA | NA | NO |
| 1430257 | 537 | 0 | NA | NA | 1,42 | 2,82E-06 | 6,40E-03 | NO |
| 1430360 | 125 | 0 | NA | NA | 0 | NA | NA | NO |
| 1430361 | 2345 | 0 | NA | NA | 0 | NA | NA | NO |
| 1430471 | 21 | 0 | NA | NA | 0 | NA | NA | NO |
| 1430579 | 1283 | 0 | NA | NA | 0 | NA | NA | NO |
| 1430626 | 650 | 0 | NA | NA | -1,22 | 4,68E-06 | 1,09E-02 | NO |
| 1430635 | 1496 | 0 | NA | NA | 0 | NA | NA | NO |
| 1430818 | 724 | 0 | NA | NA | 0 | NA | NA | NO |
| 1431003 | 1157 | 0 | NA | NA | 0 | NA | NA | NO |
| 1431011 | 486 | 0 | NA | NA | 0 | NA | NA | NO |
| 1431171 | 43 | 0 | NA | NA | 0 | NA | NA | NO |
| 1431172 | 546 | 0 | NA | NA | 1,6 | 1,07E-05 | 2,65E-02 | NO |
| 1431206 | 1913 | 0 | NA | NA | 0,92 | 1,77E-06 | 3,90E-03 | NO |
| 1431253 | 4709 | 0 | NA | NA | 0 | NA | NA | NO |
| 1431273 | 1308 | 0 | NA | NA | -1,58 | 5,64E-08 | 9,90E-05 | NO |
| 1431290 | 675 | 0 | NA | NA | 0 | NA | NA | NO |
| 1431327 | 1109 | 0 | NA | NA | 0 | NA | NA | NO |
| 1431362 | 4319 | 0 | NA | NA | 0 | NA | NA | NO |
| 1431369 | 1117 | 0 | NA | NA | 0 | NA | NA | NO |
| 1431376 | 150 | 0 | NA | NA | 0 | NA | NA | NO |
| 1431487 | 612 | 0 | NA | NA | 1,05 | 5,90E-06 | 1,41E-02 | NO |
| 1431649 | 5010 | 0 | NA | NA | 0 | NA | NA | NO |
| 1431730 | 1168 | 0 | NA | NA | -2,02 | 1,60E-11 | 1,91E-08 | NO |
| 1431766 | 2922 | 0 | NA | NA | 0 | NA | NA | NO |
| 1431779 | 472 | 0 | NA | NA | 1,39 | 1,26E-09 | 1,81E-06 | NO |
| 1431780 | 945 | 0 | NA | NA | 1,63 | 2,80E-14 | 2,58E-11 | NO |
| 1431825 | 751 | 0 | NA | NA | 0 | NA | NA | NO |
| 1431853 | 597 | 0 | NA | NA | -1,23 | 3,98E-07 | 7,89E-04 | NO |
| 1431921 | 207 | 0 | NA | NA | -2,96 | 1,76E-15 | 1,50E-12 | NO |
| 1432031 | 1820 | 0 | NA | NA | 0 | NA | NA | NO |
| 1432101 | 572 | 0 | NA | NA | 2,48 | 1,29E-13 | 1,25E-10 | NO |
| 1432155 | 279 | 0 | NA | NA | 0 | NA | NA | NO |
| 1432223 | 3782 | 0 | NA | NA | 0 | NA | NA | NO |
| 1432299 | 295 | 0 | NA | NA | 1,53 | 4,14E-09 | 6,34E-06 | NO |
| 1432307 | 41 | 0 | NA | NA | -2,02 | 3,89E-08 | 6,69E-05 | NO |
| 1432365 | 132 | 0 | NA | NA | 1,74 | 4,30E-07 | 8,58E-04 | NO |
| 1432591 | 18 | 0 | NA | NA | 2,95 | 1,35E-05 | 3,42E-02 | NO |
| 1432615 | 314 | 0 | NA | NA | 0 | NA | NA | NO |
| 1432650 | 1582 | 0 | NA | NA | 2,1 | 2,24E-07 | 4,27E-04 | NO |
| 1432787 | 2845 | 0 | NA | NA | 1,81 | 1,54E-13 | 1,51E-10 | NO |
| 1432803 | 581 | 0 | NA | NA | 1,12 | 1,56E-05 | 3,99E-02 | NO |
| 1432856 | 7683 | 0 | NA | NA | 1,5 | 3,26E-06 | 7,45E-03 | NO |
| 1432866 | 1963 | 0 | NA | NA | 0 | NA | NA | NO |
| 1432987 | 18911 | 0 | NA | NA | 0 | NA | NA | NO |
| 1433051 | 306 | 0 | NA | NA | -1,75 | 5,47E-08 | 9,59E-05 | NO |
| 1433137 | 2711 | 0 | NA | NA | 0 | NA | NA | NO |
| 1433155 | 191 | 0 | NA | NA | 0 | NA | NA | NO |
| 1433211 | 941 | 0 | NA | NA | 0 | NA | NA | NO |
| 1433366 | 113 | 0 | NA | NA | 0 | NA | NA | NO |
| 1433489 | 75 | 0 | NA | NA | 0 | NA | NA | NO |
| 1433530 | 1057 | 0 | NA | NA | 0 | NA | NA | NO |
| 1433607 | 1553 | 0 | NA | NA | 1,91 | 1,04E-11 | 1,21E-08 | NO |
| 1433673 | 253 | 0 | NA | NA | 0 | NA | NA | NO |
| 1433771 | 4411 | 0 | NA | NA | 0 | NA | NA | NO |
| 1433868 | 692 | 0 | NA | NA | 0 | NA | NA | NO |
| 1434041 | 878 | 0 | NA | NA | -1,18 | 5,40E-07 | 1,10E-03 | NO |
| 1434045 | 88 | 0 | NA | NA | 0 | NA | NA | NO |
| 1434103 | 62 | 0 | NA | NA | 0 | NA | NA | NO |
| 1434104 | 92 | 0 | NA | NA | 0 | NA | NA | NO |
| 1434133 | 551 | 0 | NA | NA | 1 | 4,99E-06 | 1,17E-02 | NO |
| 1434136 | 308 | 0 | NA | NA | 0 | NA | NA | NO |
| 1434146 | 1631 | 0 | NA | NA | 0 | NA | NA | NO |
| 1434177 | 918 | 0 | NA | NA | 0 | NA | NA | NO |
| 1434221 | 134 | 0 | NA | NA | 0 | NA | NA | NO |
| 1434273 | 11 | 0 | NA | NA | 3,79 | 1,18E-06 | 2,54E-03 | NO |
| 1434281 | 567 | 0 | NA | NA | -1,43 | 1,71E-12 | 1,84E-09 | NO |
| 1434290 | 4283 | 0 | NA | NA | 0 | NA | NA | NO |
| 1434540 | 3710 | 0 | NA | NA | 0 | NA | NA | NO |
| 1434621 | 1151 | 0 | NA | NA | 0 | NA | NA | NO |
| 1434657 | 702 | 0 | NA | NA | 1,68 | 1,69E-12 | 1,82E-09 | NO |
| 1434677 | 2401 | 0 | NA | NA | -1,34 | 1,99E-07 | 3,74E-04 | NO |
| 1434681 | 5559 | 0 | NA | NA | -1,19 | 4,18E-06 | 9,72E-03 | NO |
| 1434830 | 197 | 0 | NA | NA | 1,7 | 2,48E-06 | 5,59E-03 | NO |
| 1434906 | 2050 | 0 | NA | NA | 1,03 | 2,48E-06 | 5,58E-03 | NO |
| 1434989 | 867 | 0 | NA | NA | 0 | NA | NA | NO |
| 1435056 | 136 | 0 | NA | NA | 0 | NA | NA | NO |
| 1435320 | 16398 | 0 | NA | NA | 0 | NA | NA | NO |
| 1435473 | 2274 | 0 | NA | NA | 0 | NA | NA | NO |
| 1435586 | 816 | 0 | NA | NA | 0 | NA | NA | NO |
| 1435636 | 77 | 0 | NA | NA | -1,63 | 1,98E-09 | 2,90E-06 | NO |
| 1435649 | 659 | 0 | NA | NA | 0 | NA | NA | NO |
| 1435709 | 3959 | 0 | NA | NA | 0 | NA | NA | NO |
| 1435712 | 86 | 0 | NA | NA | 0 | NA | NA | NO |
| 1435715 | 7473 | 0 | NA | NA | -1,99 | 3,37E-08 | 5,75E-05 | NO |
| 1435730 | 1365 | 0 | NA | NA | -1,7 | 1,34E-05 | 3,40E-02 | NO |
| 1435779 | 21 | 0 | NA | NA | 2,57 | 9,47E-06 | 2,35E-02 | NO |
| 1436060 | 358 | 0 | NA | NA | 0 | NA | NA | NO |
| 1436231 | 934 | 0 | NA | NA | 0 | NA | NA | NO |
| 1436268 | 1468 | 0 | NA | NA | 1,2 | 8,23E-07 | 1,71E-03 | NO |
| 1436271 | 1623 | 0 | NA | NA | -1,26 | 5,86E-07 | 1,20E-03 | NO |
| 1436302 | 155 | 0 | NA | NA | 2,2 | 5,52E-10 | 7,64E-07 | NO |
| 1436333 | 1122 | 0 | NA | NA | 0 | NA | NA | NO |
| 1436412 | 6540 | 0 | NA | NA | 0 | NA | NA | NO |
| 1436443 | 659 | 0 | NA | NA | 0 | NA | NA | NO |
| 1436498 | 1469 | 0 | NA | NA | 0 | NA | NA | NO |
| 1436517 | 114 | 0 | NA | NA | 0 | NA | NA | NO |
| 1436582 | 2146 | 0 | NA | NA | 0 | NA | NA | NO |
| 1436597 | 24 | 0 | NA | NA | 0 | NA | NA | NO |
| 1436625 | 303 | 0 | NA | NA | 0 | NA | NA | NO |
| 1436638 | 2105 | 0 | NA | NA | 1,21 | 1,66E-05 | 4,26E-02 | NO |
| 1436690 | 227 | 0 | NA | NA | 0 | NA | NA | NO |
| 1436691 | 382 | 0 | NA | NA | 1,08 | 2,71E-06 | 6,13E-03 | NO |
| 1436782 | 49589 | 0 | NA | NA | 1,71 | 1,95E-09 | 2,86E-06 | NO |
| 1436793 | 1634 | 0 | NA | NA | 0 | NA | NA | NO |
| 1436812 | 457 | 0 | NA | NA | 0 | NA | NA | NO |
| 1436891 | 4970 | 0 | NA | NA | -0,74 | 1,03E-05 | 2,57E-02 | NO |
| 1436909 | 177 | 0 | NA | NA | 2,13 | 1,42E-14 | 1,29E-11 | NO |
| 1437095 | 1883 | 0 | NA | NA | 2,44 | 4,71E-13 | 4,77E-10 | NO |
| 1437132 | 74 | 0 | NA | NA | -1,87 | 1,49E-07 | 2,74E-04 | NO |
| 1437152 | 71 | 0 | NA | NA | 0 | NA | NA | NO |
| 1437192 | 757 | 0 | NA | NA | 0 | NA | NA | NO |
| 1437210 | 103 | 0 | NA | NA | 0 | NA | NA | NO |
| 1437211 | 930 | 0 | NA | NA | 0 | NA | NA | NO |
| 1437266 | 168 | 0 | NA | NA | 0 | NA | NA | NO |
| 1437319 | 1091 | 0 | NA | NA | 0 | NA | NA | NO |
| 1437349 | 702 | 0 | NA | NA | 0 | NA | NA | NO |
| 1437365 | 122 | 0 | NA | NA | 1,83 | 2,67E-08 | 4,48E-05 | NO |
| 1437408 | 2624 | 0 | NA | NA | 0 | NA | NA | NO |
| 1437520 | 195 | 0 | NA | NA | 1,7 | 6,64E-07 | 1,37E-03 | NO |
| 1437527 | 13 | 0 | NA | NA | 2,93 | 1,27E-05 | 3,20E-02 | NO |
| 1437554 | 7 | 0 | NA | NA | 0 | NA | NA | NO |
| 1437660 | 321 | 0 | NA | NA | 1,01 | 6,90E-06 | 1,67E-02 | NO |
| 1437671 | 186 | 0 | NA | NA | 0 | NA | NA | NO |
| 1437689 | 1180 | 0 | NA | NA | 2,23 | 2,30E-15 | 1,97E-12 | NO |
| 1437698 | 1087 | 0 | NA | NA | 0 | NA | NA | NO |
| 1437808 | 3459 | 0 | NA | NA | 0 | NA | NA | NO |
| 1437947 | 1026 | 0 | NA | NA | 0 | NA | NA | NO |
| 1437949 | 41 | 0 | NA | NA | 2,01 | 4,74E-06 | 1,11E-02 | NO |
| 1437976 | 1363 | 0 | NA | NA | -2,22 | 3,79E-09 | 5,76E-06 | NO |
| 1438046 | 97 | 0 | NA | NA | -1,89 | 8,90E-07 | 1,87E-03 | NO |
| 1438050 | 273 | 0 | NA | NA | 0 | NA | NA | NO |
| 1438052 | 389 | 0 | NA | NA | 0 | NA | NA | NO |
| 1438067 | 1351 | 0 | NA | NA | 0,99 | 8,34E-07 | 1,74E-03 | NO |
| 1438124 | 968 | 0 | NA | NA | 0 | NA | NA | NO |
| 1438138 | 106 | 0 | NA | NA | 2,56 | 5,00E-14 | 4,69E-11 | NO |
| 1438142 | 97 | 0 | NA | NA | 0 | NA | NA | NO |
| 1438145 | 18471 | 0 | NA | NA | 0 | NA | NA | NO |
| 1438155 | 7783 | 0 | NA | NA | 0 | NA | NA | NO |
| 1438165 | 1888 | 0 | NA | NA | 0 | NA | NA | NO |
| 1438187 | 111 | 0 | NA | NA | 0 | NA | NA | NO |
| 1438307 | 2042 | 0 | NA | NA | 0 | NA | NA | NO |
| 1438323 | 414 | 0 | NA | NA | 1,76 | 3,56E-08 | 6,10E-05 | NO |
| 1438434 | 57 | 0 | NA | NA | 0 | NA | NA | NO |
| 1438562 | 1333 | 0 | NA | NA | 0 | NA | NA | NO |
| 1438639 | 417 | 0 | NA | NA | 0 | NA | NA | NO |
| 1438743 | 2928 | 0 | NA | NA | 0 | NA | NA | NO |
| 1438771 | 2729 | 0 | NA | NA | 1,56 | 1,15E-05 | 2,88E-02 | NO |
| 1438784 | 55 | 0 | NA | NA | 0 | NA | NA | NO |
| 1438790 | 2166 | 0 | NA | NA | 0 | NA | NA | NO |
| 1438820 | 1010 | 0 | NA | NA | 0 | NA | NA | NO |
| 1438882 | 1732 | 0 | NA | NA | 0 | NA | NA | NO |
| 1438946 | 344 | 0 | NA | NA | 0 | NA | NA | NO |
| 1438958 | 289 | 0 | NA | NA | 0 | NA | NA | NO |
| 1438991 | 3855 | 0 | NA | NA | 1,97 | 7,41E-17 | 5,71E-14 | NO |
| 1439044 | 815 | 0 | NA | NA | 0 | NA | NA | NO |
| 1439075 | 94 | 0 | NA | NA | -3,14 | 8,65E-09 | 1,37E-05 | NO |
| 1439091 | 9759 | 0 | NA | NA | 1,38 | 7,88E-14 | 7,55E-11 | NO |
| 1439271 | 817 | 0 | NA | NA | 0,94 | 2,18E-07 | 4,13E-04 | NO |
| 1439291 | 1517 | 0 | NA | NA | 0 | NA | NA | NO |
| 1439316 | 112 | 0 | NA | NA | -4,87 | 1,12E-19 | 7,11E-17 | NO |
| 1439394 | 346 | 0 | NA | NA | 0 | NA | NA | NO |
| 1439438 | 1416 | 0 | NA | NA | 0 | NA | NA | NO |
| 1439543 | 430 | 0 | NA | NA | 2,06 | 7,11E-08 | 1,26E-04 | NO |
| 1439711 | 289 | 0 | NA | NA | 0 | NA | NA | NO |
| 1439754 | 1075 | 0 | NA | NA | -1,67 | 5,07E-06 | 1,19E-02 | NO |
| 1439892 | 709 | 0 | NA | NA | 0 | NA | NA | NO |
| 1440012 | 890 | 0 | NA | NA | 0 | NA | NA | NO |
| 1440037 | 1453 | 0 | NA | NA | 0 | NA | NA | NO |
| 1440418 | 620 | 0 | NA | NA | -3,89 | 1,70E-29 | 5,82E-27 | NO |
| 1440426 | 7816 | 0 | NA | NA | 0 | NA | NA | NO |
| 1440439 | 67 | 0 | NA | NA | 0 | NA | NA | NO |
| 1440520 | 25 | 0 | NA | NA | 0 | NA | NA | NO |
| 1440571 | 297 | 0 | NA | NA | 0 | NA | NA | NO |
| 1440586 | 625 | 0 | NA | NA | 0 | NA | NA | NO |
| 1440593 | 5720 | 0 | NA | NA | 1,35 | 9,86E-07 | 2,09E-03 | NO |
| 1440712 | 904 | 0 | NA | NA | 0 | NA | NA | NO |
| 1440742 | 429 | 0 | NA | NA | 0 | NA | NA | NO |
| 1440842 | 56 | 0 | NA | NA | -1,84 | 1,44E-06 | 3,14E-03 | NO |
| 1440928 | 3032 | 0 | NA | NA | 1,75 | 4,24E-17 | 3,21E-14 | NO |
| 1441006 | 228 | 0 | NA | NA | 0 | NA | NA | NO |
| 1441040 | 298 | 0 | NA | NA | 0 | NA | NA | NO |
| 1441176 | 9 | 0 | NA | NA | 0 | NA | NA | NO |
| 1441195 | 228 | 0 | NA | NA | 0 | NA | NA | NO |
| 1441656 | 1571 | 0 | NA | NA | 1,11 | 2,19E-07 | 4,15E-04 | NO |
| 1442304 | 352 | 0 | NA | NA | 0 | NA | NA | NO |
| 1442338 | 408 | 0 | NA | NA | 0 | NA | NA | NO |
| 1442511 | 970 | 0 | NA | NA | 1,12 | 1,67E-05 | 4,30E-02 | NO |
| 1442690 | 31 | 0 | NA | NA | 0 | NA | NA | NO |
| 1442759 | 1642 | 0 | NA | NA | 0 | NA | NA | NO |
| 1442844 | 13588 | 0 | NA | NA | 0 | NA | NA | NO |
| 1443788 | 997 | 0 | NA | NA | 0 | NA | NA | NO |
| 1443936 | 492 | 0 | NA | NA | 1,34 | 3,41E-09 | 5,16E-06 | NO |
| 1444108 | 105938 | 0 | NA | NA | 0 | NA | NA | NO |
| 1444180 | 395 | 0 | NA | NA | 0 | NA | NA | NO |
| 1444758 | 3616 | 0 | NA | NA | 0 | NA | NA | NO |
| 1445733 | 456 | 0 | NA | NA | 0 | NA | NA | NO |
| 1445747 | 42 | 0 | NA | NA | 0 | NA | NA | NO |
| 1445825 | 429 | 0 | NA | NA | 0 | NA | NA | NO |
| 1446046 | 1012 | 0 | NA | NA | 0 | NA | NA | NO |
| 1446370 | 137 | 0 | NA | NA | 1,59 | 5,18E-06 | 1,22E-02 | NO |
| 1447069 | 701 | 0 | NA | NA | 0 | NA | NA | NO |
| 1447108 | 455 | 0 | NA | NA | 0 | NA | NA | NO |
| 1447451 | 297 | 0 | NA | NA | 0 | NA | NA | NO |
| 1447606 | 477 | 0 | NA | NA | 0 | NA | NA | NO |
| 1447737 | 2015 | 0 | NA | NA | 0 | NA | NA | NO |
| 1447991 | 483 | 0 | NA | NA | 1,15 | 1,00E-05 | 2,49E-02 | NO |
| 1448047 | 51 | 0 | NA | NA | 2,74 | 6,44E-11 | 8,09E-08 | NO |
| 1448716 | 282 | 0 | NA | NA | 0 | NA | NA | NO |
| 1448866 | 1742 | 0 | NA | NA | -1,47 | 3,14E-12 | 3,47E-09 | NO |
| 1449013 | 695 | 0 | NA | NA | 0 | NA | NA | NO |
| 1449712 | 696 | 0 | NA | NA | 0 | NA | NA | NO |
| 1449892 | 7124 | 0 | NA | NA | -1,02 | 1,44E-05 | 3,67E-02 | NO |
| 1450609 | 213 | 0 | NA | NA | 0 | NA | NA | NO |
| 1450689 | 1546 | 0 | NA | NA | 0 | NA | NA | NO |
| 1450783 | 1722 | 0 | NA | NA | 0 | NA | NA | NO |
| 1451702 | 6006 | 0 | NA | NA | 0 | NA | NA | NO |
| 1452177 | 711 | 0 | NA | NA | 0 | NA | NA | NO |
| 1453073 | 3079 | 0 | NA | NA | 0 | NA | NA | NO |
| 1454567 | 527 | 0 | NA | NA | -1,65 | 7,62E-14 | 7,28E-11 | NO |
| 1455000 | 244 | 0 | NA | NA | 0 | NA | NA | NO |
| 1455092 | 126 | 0 | NA | NA | 0 | NA | NA | NO |
| 1455819 | 949 | 0 | NA | NA | 0 | NA | NA | NO |
| 1456176 | 163 | 0 | NA | NA | 1,24 | 1,65E-06 | 3,62E-03 | NO |
| 1456549 | 24 | 0 | NA | NA | 2,49 | 5,14E-07 | 1,04E-03 | NO |
| 1456652 | 1161 | 0 | NA | NA | 0 | NA | NA | NO |
| 1456706 | 55 | 0 | NA | NA | 0 | NA | NA | NO |
| 1457318 | 320 | 0 | NA | NA | 0 | NA | NA | NO |
| 1458186 | 3043 | 0 | NA | NA | 1,33 | 2,20E-12 | 2,38E-09 | NO |
| 1458585 | 760 | 0 | NA | NA | -1,34 | 1,58E-07 | 2,92E-04 | NO |
| 1460490 | 370 | 0 | NA | NA | 0 | NA | NA | NO |
| 1460558 | 921 | 0 | NA | NA | 1,95 | 9,00E-11 | 1,15E-07 | NO |
| 1461199 | 253 | 0 | NA | NA | 0 | NA | NA | NO |
| 1462501 | 610 | 0 | NA | NA | 0 | NA | NA | NO |
| 1462613 | 244 | 0 | NA | NA | -2,15 | 2,06E-08 | 3,41E-05 | NO |
| 1462629 | 449 | 0 | NA | NA | 1,19 | 2,75E-06 | 6,23E-03 | NO |
| 1462732 | 881 | 0 | NA | NA | 0 | NA | NA | NO |
| 1462870 | 232 | 0 | NA | NA | 0 | NA | NA | NO |
| 1462877 | 55 | 0 | NA | NA | 0 | NA | NA | NO |
| 1462908 | 1719 | 0 | NA | NA | 0 | NA | NA | NO |
| 1462922 | 113 | 0 | NA | NA | 0 | NA | NA | NO |
| 1463066 | 5508 | 0 | NA | NA | -2,24 | 1,65E-11 | 1,96E-08 | NO |
| 1463207 | 59 | 0 | NA | NA | 0 | NA | NA | NO |
| 1463209 | 807 | 0 | NA | NA | 0 | NA | NA | NO |
| 1463746 | 189 | 0 | NA | NA | 0 | NA | NA | NO |
| 1463755 | 913 | 0 | NA | NA | 0 | NA | NA | NO |
| 1463764 | 527 | 0 | NA | NA | 0 | NA | NA | NO |
| 1463785 | 195 | 0 | NA | NA | 0 | NA | NA | NO |
| 1464180 | 413 | 0 | NA | NA | 0 | NA | NA | NO |
| 1464612 | 175 | 0 | NA | NA | 0 | NA | NA | NO |
| 1464857 | 39 | 0 | NA | NA | 0 | NA | NA | NO |
| 1464918 | 946 | 0 | NA | NA | 0 | NA | NA | NO |
| 1464961 | 493 | 0 | NA | NA | 0 | NA | NA | NO |
| 1464963 | 307 | 0 | NA | NA | 0 | NA | NA | NO |
| 1464992 | 393 | 0 | NA | NA | 0 | NA | NA | NO |
| 1465031 | 1049 | 0 | NA | NA | 0 | NA | NA | NO |
| 1465172 | 73 | 0 | NA | NA | 0 | NA | NA | NO |
| 1465360 | 70 | 0 | NA | NA | 0 | NA | NA | NO |
| 1465385 | 576 | 0 | NA | NA | 0 | NA | NA | NO |
| 1465390 | 522 | 0 | NA | NA | 0,99 | 1,59E-05 | 4,08E-02 | NO |
| 1465420 | 158 | 0 | NA | NA | 0 | NA | NA | NO |
| 1465448 | 754 | 0 | NA | NA | 0,98 | 2,33E-06 | 5,22E-03 | NO |
| 1465456 | 58 | 0 | NA | NA | 0 | NA | NA | NO |
| 1465784 | 295 | 0 | NA | NA | 1,85 | 1,44E-06 | 3,13E-03 | NO |
| 1465908 | 73 | 0 | NA | NA | 1,38 | 9,09E-06 | 2,25E-02 | NO |
| 1465930 | 383 | 0 | NA | NA | 0 | NA | NA | NO |
| 1466038 | 4004 | 0 | NA | NA | 0 | NA | NA | NO |
| 1466137 | 6466 | 0 | NA | NA | 0 | NA | NA | NO |
| 1466307 | 2159 | 0 | NA | NA | 0 | NA | NA | NO |
| 1466534 | 15541 | 0 | NA | NA | 0 | NA | NA | NO |
| 1466657 | 68 | 0 | NA | NA | 1,81 | 9,32E-06 | 2,31E-02 | NO |
| 1466699 | 116 | 0 | NA | NA | 0 | NA | NA | NO |
| 1466700 | 19 | 0 | NA | NA | 0 | NA | NA | NO |
| 1466796 | 1012 | 0 | NA | NA | 2,22 | 1,53E-12 | 1,64E-09 | NO |
| 1466903 | 68 | 0 | NA | NA | 0 | NA | NA | NO |
| 1466955 | 137 | 0 | NA | NA | 0 | NA | NA | NO |
| 1466977 | 88 | 0 | NA | NA | 0 | NA | NA | NO |
| 1467043 | 43 | 0 | NA | NA | 2,28 | 4,45E-07 | 8,90E-04 | NO |
| 1467066 | 408 | 0 | NA | NA | 0 | NA | NA | NO |
| 1467250 | 1175 | 0 | NA | NA | 0 | NA | NA | NO |
| 1467399 | 2015 | 0 | NA | NA | 0 | NA | NA | NO |
| 1467401 | 665 | 0 | NA | NA | 0 | NA | NA | NO |
| 1467480 | 578 | 0 | NA | NA | -2,02 | 2,21E-07 | 4,19E-04 | NO |
| 1467706 | 27 | 0 | NA | NA | 0 | NA | NA | NO |
| 1467895 | 5927 | 0 | NA | NA | 0 | NA | NA | NO |
| 1467918 | 645 | 0 | NA | NA | 0 | NA | NA | NO |
| 1467924 | 165 | 0 | NA | NA | 0 | NA | NA | NO |
| 1467929 | 424 | 0 | NA | NA | 1,47 | 1,28E-05 | 3,23E-02 | NO |
| 1468002 | 706 | 0 | NA | NA | 0 | NA | NA | NO |
| 1468376 | 4054 | 0 | NA | NA | 0 | NA | NA | NO |
| 1468397 | 750 | 0 | NA | NA | 0 | NA | NA | NO |
| 1468413 | 3999 | 0 | NA | NA | 1,19 | 2,01E-06 | 4,46E-03 | NO |
| 1468513 | 415 | 0 | NA | NA | 0 | NA | NA | NO |
| 1468581 | 162 | 0 | NA | NA | 1,45 | 7,23E-06 | 1,75E-02 | NO |
| 1468588 | 1468 | 0 | NA | NA | 1,45 | 1,30E-09 | 1,86E-06 | NO |
| 1468672 | 18711 | 0 | NA | NA | -2,66 | 5,78E-06 | 1,37E-02 | NO |
| 1468775 | 10963 | 0 | NA | NA | 0 | NA | NA | NO |
| 1468829 | 259 | 0 | NA | NA | -1,33 | 8,08E-08 | 1,44E-04 | NO |
| 1468846 | 25 | 0 | NA | NA | 2,66 | 2,04E-06 | 4,53E-03 | NO |
| 1469171 | 6535 | 0 | NA | NA | 0 | NA | NA | NO |
| 1469218 | 315 | 0 | NA | NA | 0 | NA | NA | NO |
| 1469313 | 902 | 0 | NA | NA | 2,14 | 2,98E-08 | 5,04E-05 | NO |
| 1469417 | 16387 | 0 | NA | NA | 0 | NA | NA | NO |
| 1469552 | 269 | 0 | NA | NA | 0 | NA | NA | NO |
| 1469564 | 347 | 0 | NA | NA | 0 | NA | NA | NO |
| 1469735 | 5794 | 0 | NA | NA | 0 | NA | NA | NO |
| 1469739 | 15643 | 0 | NA | NA | 0 | NA | NA | NO |
| 1469938 | 71 | 0 | NA | NA | 0 | NA | NA | NO |
| 1470034 | 32 | 0 | NA | NA | 0 | NA | NA | NO |
| 1470072 | 11797 | 0 | NA | NA | 0 | NA | NA | NO |
| 1470104 | 317 | 0 | NA | NA | -1,18 | 9,62E-07 | 2,03E-03 | NO |
| 1470135 | 412 | 0 | NA | NA | 1,31 | 1,04E-06 | 2,20E-03 | NO |
| 1470450 | 1803 | 0 | NA | NA | 0 | NA | NA | NO |
| 1470463 | 14515 | 0 | NA | NA | 0 | NA | NA | NO |
| 1471068 | 1673 | 0 | NA | NA | -2 | 8,86E-09 | 1,40E-05 | NO |
| 1471107 | 75 | 0 | NA | NA | 0 | NA | NA | NO |
| 1471246 | 171 | 0 | NA | NA | 1,16 | 4,18E-06 | 9,70E-03 | NO |
| 1471276 | 241 | 0 | NA | NA | 3,63 | 6,69E-52 | 6,89E-50 | NO |
| 1471324 | 608 | 0 | NA | NA | 0 | NA | NA | NO |
| 1471329 | 754 | 0 | NA | NA | 0 | NA | NA | NO |
| 1471958 | 574 | 0 | NA | NA | 2,19 | 2,75E-19 | 1,79E-16 | NO |
| 1472091 | 2945 | 0 | NA | NA | 0 | NA | NA | NO |
| 1472143 | 837 | 0 | NA | NA | 0 | NA | NA | NO |
| 1472199 | 295 | 0 | NA | NA | 1,09 | 9,83E-07 | 2,08E-03 | NO |
| 1472495 | 302 | 0 | NA | NA | 0 | NA | NA | NO |
| 1472908 | 428 | 0 | NA | NA | 0 | NA | NA | NO |
| 1473406 | 367 | 0 | NA | NA | 0 | NA | NA | NO |
| 1473489 | 37 | 0 | NA | NA | 0 | NA | NA | NO |
| 1473951 | 7127 | 0 | NA | NA | 2,68 | 1,51E-18 | 1,04E-15 | NO |
| 1474151 | 15316 | 0 | NA | NA | 0 | NA | NA | NO |
| 1474169 | 30 | 0 | NA | NA | 3,45 | 2,99E-07 | 5,79E-04 | NO |
| 1474252 | 55 | 0 | NA | NA | 0 | NA | NA | NO |
| 1474296 | 223 | 0 | NA | NA | -1,36 | 4,69E-07 | 9,40E-04 | NO |
| 1474680 | 225 | 0 | NA | NA | 0 | NA | NA | NO |
| 1474864 | 83 | 0 | NA | NA | -1,86 | 2,87E-10 | 3,84E-07 | NO |
| 1474962 | 454 | 0 | NA | NA | 0 | NA | NA | NO |
| 1475471 | 188 | 0 | NA | NA | 1,95 | 1,42E-09 | 2,05E-06 | NO |
| 1475659 | 2232 | 0 | NA | NA | 0 | NA | NA | NO |
| 1475665 | 2851 | 0 | NA | NA | 0 | NA | NA | NO |
| 1475728 | 320 | 0 | NA | NA | 0 | NA | NA | NO |
| 1475775 | 154 | 0 | NA | NA | 2,53 | 6,80E-12 | 7,80E-09 | NO |
| 1475848 | 658 | 0 | NA | NA | 2,06 | 8,50E-14 | 8,17E-11 | NO |
| 1475883 | 50 | 0 | NA | NA | -1,93 | 3,01E-06 | 6,84E-03 | NO |
| 1476333 | 8989 | 0 | NA | NA | 0 | NA | NA | NO |
| 1476335 | 1690 | 0 | NA | NA | 0 | NA | NA | NO |
| 1477210 | 4226 | 0 | NA | NA | 0 | NA | NA | NO |
| 1477217 | 535 | 0 | NA | NA | 0 | NA | NA | NO |
| 1477549 | 41 | 0 | NA | NA | 0 | NA | NA | NO |
| 1477699 | 29 | 0 | NA | NA | 0 | NA | NA | NO |
| 1478000 | 527 | 0 | NA | NA | 0 | NA | NA | NO |
| 1478011 | 282 | 0 | NA | NA | 0 | NA | NA | NO |
| 1478097 | 52 | 0 | NA | NA | 1,62 | 3,75E-06 | 8,67E-03 | NO |
| 1478131 | 456 | 0 | NA | NA | 0 | NA | NA | NO |
| 1478819 | 295 | 0 | NA | NA | 0 | NA | NA | NO |
| 1479005 | 138 | 0 | NA | NA | 0 | NA | NA | NO |
| 1479044 | 184 | 0 | NA | NA | 0 | NA | NA | NO |
| 1479045 | 168 | 0 | NA | NA | 0 | NA | NA | NO |
| 1479061 | 74 | 0 | NA | NA | 0 | NA | NA | NO |
| 1479646 | 779 | 0 | NA | NA | 0 | NA | NA | NO |
| 1479872 | 63 | 0 | NA | NA | 0 | NA | NA | NO |
| 1480490 | 40099 | 0 | NA | NA | 1,69 | 1,96E-07 | 3,67E-04 | NO |
| 1480792 | 446 | 0 | NA | NA | 1,38 | 8,59E-07 | 1,80E-03 | NO |
| 1480850 | 515 | 0 | NA | NA | 0 | NA | NA | NO |
| 1481058 | 210 | 0 | NA | NA | 0 | NA | NA | NO |
| 1481060 | 83 | 0 | NA | NA | 0 | NA | NA | NO |
| 1481080 | 202 | 0 | NA | NA | -1,52 | 5,69E-06 | 1,35E-02 | NO |
| 1481190 | 597 | 0 | NA | NA | 0 | NA | NA | NO |
| 1481353 | 196 | 0 | NA | NA | 0 | NA | NA | NO |
| 1481551 | 1242 | 0 | NA | NA | 0 | NA | NA | NO |
| 1481611 | 510 | 0 | NA | NA | 0 | NA | NA | NO |
| 1481636 | 750 | 0 | NA | NA | 1,16 | 1,79E-05 | 4,62E-02 | NO |
| 1481665 | 524 | 0 | NA | NA | 0 | NA | NA | NO |
| 1491109 | 7044 | 0 | NA | NA | -2,15 | 1,97E-07 | 3,69E-04 | NO |
| 1495213 | 905 | 0 | NA | NA | 0 | NA | NA | NO |
| 1501688 | 2211 | 0 | NA | NA | -1,69 | 1,38E-06 | 2,99E-03 | NO |
| 1511553 | 16 | 0 | NA | NA | 0 | NA | NA | NO |
| 1513435 | 319 | 0 | NA | NA | 1 | 5,53E-06 | 1,31E-02 | NO |
| 1531380 | 3055 | 0 | NA | NA | 0 | NA | NA | NO |
| 1533092 | 246 | 0 | NA | NA | 0 | NA | NA | NO |
| 1436682 | 3297 | 0,8 | 3,54E-06 | 1,04E-02 | 0 | NA | NA | NO |
| 1440718 | 588 | 0,82 | 7,45E-06 | 2,30E-02 | 0 | NA | NA | NO |
| 1361443 | 13223 | 0,84 | 3,60E-06 | 1,07E-02 | 0 | NA | NA | NO |
| 1509747 | 406 | 0,86 | 5,42E-06 | 1,64E-02 | 0 | NA | NA | NO |
| 1436593 | 638 | 0,87 | 8,98E-06 | 2,81E-02 | 0,87 | 9,32E-06 | 2,30E-02 | NO |
| 1471672 | 471 | 0,9 | 1,45E-05 | 4,68E-02 | 0 | NA | NA | NO |
| 378331 | 928 | 0,91 | 6,86E-09 | 1,40E-05 | 0 | NA | NA | NO |
| 1438828 | 1423 | 0,91 | 4,64E-06 | 1,39E-02 | 0 | NA | NA | NO |
| 1403293 | 536 | 0,92 | 2,00E-06 | 5,69E-03 | 0 | NA | NA | NO |
| 1443464 | 5283 | 0,92 | 1,16E-05 | 3,71E-02 | 0 | NA | NA | NO |
| 1362181 | 544 | 0,93 | 7,66E-07 | 2,06E-03 | 0 | NA | NA | NO |
| 1436386 | 1240 | 0,93 | 1,38E-05 | 4,46E-02 | 1,78 | 7,32E-18 | 5,23E-15 | NO |
| 1428771 | 1159 | 0,94 | 6,94E-07 | 1,85E-03 | 0 | NA | NA | NO |
| 1433870 | 1160 | 0,94 | 4,47E-06 | 1,34E-02 | 0 | NA | NA | NO |
| 1416087 | 2207 | 0,95 | 8,43E-06 | 2,62E-02 | 0 | NA | NA | NO |
| 1450789 | 2282 | 0,95 | 6,47E-08 | 1,48E-04 | 1,58 | 2,84E-20 | 1,73E-17 | NO |
| 1457690 | 876 | 0,96 | 1,72E-06 | 4,84E-03 | 0 | NA | NA | NO |
| 1421865 | 1467 | 0,98 | 3,06E-06 | 8,95E-03 | 1,33 | 1,70E-10 | 2,23E-07 | NO |
| 1433169 | 1593 | 0,99 | 6,92E-06 | 2,13E-02 | 0 | NA | NA | NO |
| 1440228 | 296 | 0,99 | 1,15E-05 | 3,65E-02 | 0 | NA | NA | NO |
| 1439658 | 234 | 1 | 1,34E-06 | 3,71E-03 | 0 | NA | NA | NO |
| 1478586 | 968 | 1 | 2,09E-06 | 5,97E-03 | 1,08 | 3,89E-07 | 7,70E-04 | NO |
| 1356135 | 1641 | 1,03 | 1,24E-07 | 2,97E-04 | 0,94 | 2,27E-06 | 5,08E-03 | NO |
| 1429384 | 686 | 1,03 | 4,55E-07 | 1,18E-03 | 0,93 | 7,25E-06 | 1,76E-02 | NO |
| 1466959 | 8738 | 1,03 | 9,86E-07 | 2,69E-03 | 1,07 | 4,30E-07 | 8,58E-04 | NO |
| 1473806 | 595 | 1,03 | 1,71E-06 | 4,82E-03 | 1,35 | 2,35E-10 | 3,12E-07 | NO |
| 578743 | 348 | 1,05 | 2,57E-06 | 7,45E-03 | 0 | NA | NA | NO |
| 1437528 | 2776 | 1,05 | 4,20E-07 | 1,08E-03 | 0 | NA | NA | NO |
| 749779 | 1935 | 1,06 | 1,39E-05 | 4,49E-02 | 0 | NA | NA | NO |
| 1101144 | 2768 | 1,06 | 6,37E-09 | 1,30E-05 | 0,99 | 8,63E-08 | 1,55E-04 | NO |
| 1440072 | 769 | 1,06 | 8,02E-08 | 1,86E-04 | 0,97 | 9,90E-07 | 2,10E-03 | NO |
| 1430719 | 542 | 1,07 | 8,95E-06 | 2,80E-02 | 0 | NA | NA | NO |
| 1404822 | 2743 | 1,08 | 8,49E-06 | 2,64E-02 | 0 | NA | NA | NO |
| 1470710 | 2596 | 1,08 | 3,18E-06 | 9,33E-03 | 0 | NA | NA | NO |
| 1422409 | 7297 | 1,09 | 3,66E-06 | 1,08E-02 | 1,16 | 1,04E-06 | 2,21E-03 | NO |
| 1436330 | 5559 | 1,1 | 1,30E-12 | 1,76E-09 | 0,85 | 6,48E-08 | 1,14E-04 | NO |
| 1454458 | 13075 | 1,1 | 1,33E-05 | 4,26E-02 | 0 | NA | NA | NO |
| 1438175 | 1923 | 1,11 | 8,08E-08 | 1,88E-04 | 0 | NA | NA | NO |
| 1449007 | 892 | 1,11 | 4,87E-08 | 1,10E-04 | 0 | NA | NA | NO |
| 1472010 | 192 | 1,11 | 4,13E-06 | 1,23E-02 | 1,3 | 4,97E-08 | 8,65E-05 | NO |
| 1440630 | 1165 | 1,12 | 2,37E-07 | 5,88E-04 | 0 | NA | NA | NO |
| 1423479 | 289 | 1,13 | 7,89E-06 | 2,45E-02 | 0 | NA | NA | NO |
| 1466107 | 394 | 1,13 | 1,25E-06 | 3,46E-03 | 1,15 | 9,27E-07 | 1,96E-03 | NO |
| 1373153 | 16577 | 1,15 | 4,26E-06 | 1,27E-02 | 0 | NA | NA | NO |
| 1475341 | 203 | 1,15 | 8,31E-07 | 2,25E-03 | 0 | NA | NA | NO |
| 330237 | 188 | 1,16 | 7,69E-06 | 2,38E-02 | 0 | NA | NA | NO |
| 759211 | 806 | 1,16 | 3,86E-06 | 1,15E-02 | 1,32 | 1,77E-07 | 3,28E-04 | NO |
| 1418430 | 5476 | 1,16 | 4,21E-06 | 1,26E-02 | 0 | NA | NA | NO |
| 1472975 | 1255 | 1,16 | 7,83E-12 | 1,16E-08 | 1,24 | 1,64E-13 | 1,61E-10 | NO |
| 662167 | 7048 | 1,17 | 1,27E-05 | 4,07E-02 | 0 | NA | NA | NO |
| 1435311 | 798 | 1,17 | 1,96E-06 | 5,56E-03 | 1,24 | 5,24E-07 | 1,06E-03 | NO |
| 314467 | 10765 | 1,18 | 2,04E-07 | 5,01E-04 | 0 | NA | NA | NO |
| 1335644 | 1083 | 1,18 | 6,33E-06 | 1,94E-02 | 0 | NA | NA | NO |
| 1419774 | 620 | 1,19 | 2,71E-09 | 5,28E-06 | 1,22 | 1,08E-09 | 1,53E-06 | NO |
| 1445849 | 10600 | 1,19 | 7,24E-07 | 1,94E-03 | 0 | NA | NA | NO |
| 1470989 | 47901 | 1,19 | 2,88E-08 | 6,33E-05 | 0 | NA | NA | NO |
| 1490426 | 224 | 1,19 | 7,67E-06 | 2,37E-02 | 0 | NA | NA | NO |
| 1354384 | 5721 | 1,2 | 2,17E-08 | 4,67E-05 | 0 | NA | NA | NO |
| 1481057 | 619 | 1,2 | 3,63E-07 | 9,25E-04 | 0 | NA | NA | NO |
| 771767 | 850 | 1,21 | 2,41E-07 | 6,00E-04 | 1,34 | 1,01E-08 | 1,60E-05 | NO |
| 1436186 | 11894 | 1,21 | 7,75E-07 | 2,09E-03 | 0 | NA | NA | NO |
| 877497 | 1005 | 1,22 | 1,60E-06 | 4,50E-03 | 1,36 | 1,04E-07 | 1,88E-04 | NO |
| 1423568 | 3982 | 1,22 | 5,62E-08 | 1,28E-04 | 1,36 | 1,74E-09 | 2,53E-06 | NO |
| 1464686 | 691 | 1,22 | 2,32E-06 | 6,66E-03 | 1,78 | 2,43E-12 | 2,65E-09 | NO |
| 596708 | 4041 | 1,23 | 1,11E-05 | 3,52E-02 | 0 | NA | NA | NO |
| 1435043 | 1401 | 1,23 | 6,58E-07 | 1,75E-03 | 1,19 | 2,07E-06 | 4,60E-03 | NO |
| 1475690 | 915 | 1,23 | 8,47E-06 | 2,64E-02 | 0 | NA | NA | NO |
| 1438598 | 1815 | 1,24 | 2,08E-06 | 5,93E-03 | 0 | NA | NA | NO |
| 1374933 | 9085 | 1,25 | 2,09E-07 | 5,14E-04 | 1,34 | 2,75E-08 | 4,63E-05 | NO |
| 1440901 | 363 | 1,25 | 1,70E-07 | 4,12E-04 | 1,09 | 7,30E-06 | 1,77E-02 | NO |
| 223652 | 1584 | 1,26 | 9,30E-06 | 2,91E-02 | 0 | NA | NA | NO |
| 917530 | 583 | 1,26 | 2,73E-09 | 5,32E-06 | 0 | NA | NA | NO |
| 1368854 | 2967 | 1,26 | 1,95E-06 | 5,55E-03 | 1,62 | 7,30E-10 | 1,02E-06 | NO |
| 1426500 | 619 | 1,26 | 3,17E-08 | 7,00E-05 | 1,25 | 5,35E-08 | 9,35E-05 | NO |
| 1435474 | 3988 | 1,26 | 9,01E-07 | 2,45E-03 | 1,78 | 2,22E-12 | 2,41E-09 | NO |
| 1270521 | 1754 | 1,27 | 2,72E-07 | 6,80E-04 | 0 | NA | NA | NO |
| 1374328 | 1098 | 1,27 | 1,18E-07 | 2,80E-04 | 0 | NA | NA | NO |
| 1432761 | 10200 | 1,27 | 1,02E-09 | 1,88E-06 | 1,61 | 8,44E-15 | 7,50E-12 | NO |
| 1458522 | 10259 | 1,27 | 2,35E-07 | 5,82E-04 | 1,3 | 1,41E-07 | 2,58E-04 | NO |
| 2248 | 1023 | 1,28 | 6,10E-07 | 1,61E-03 | 1,67 | 6,00E-11 | 7,51E-08 | NO |
| 1375035 | 3063 | 1,28 | 1,47E-05 | 4,76E-02 | 1,47 | 7,04E-07 | 1,45E-03 | NO |
| 1431220 | 5203 | 1,29 | 1,42E-09 | 2,66E-06 | 0 | NA | NA | NO |
| 19685 | 7707 | 1,3 | 9,19E-10 | 1,69E-06 | 1,82 | 4,68E-18 | 3,29E-15 | NO |
| 1410495 | 1372 | 1,31 | 7,51E-08 | 1,74E-04 | 2,05 | 1,08E-17 | 7,79E-15 | NO |
| 1427277 | 140 | 1,31 | 3,11E-06 | 9,11E-03 | 1,87 | 9,31E-12 | 1,08E-08 | NO |
| 1438723 | 6909 | 1,31 | 6,92E-06 | 2,13E-02 | 0 | NA | NA | NO |
| 1448777 | 3466 | 1,31 | 1,99E-06 | 5,68E-03 | 0 | NA | NA | NO |
| 317321 | 473 | 1,32 | 8,22E-08 | 1,91E-04 | 1,28 | 2,21E-07 | 4,19E-04 | NO |
| 1425618 | 344 | 1,32 | 1,13E-08 | 2,37E-05 | 0 | NA | NA | NO |
| 1434014 | 8464 | 1,32 | 8,07E-08 | 1,87E-04 | 3,15 | 1,39E-39 | 2,79E-37 | NO |
| 1437992 | 9912 | 1,32 | 7,84E-08 | 1,82E-04 | 1,45 | 4,07E-09 | 6,21E-06 | NO |
| 9834 | 1882 | 1,33 | 7,76E-09 | 1,59E-05 | 0 | NA | NA | NO |
| 1476589 | 1187 | 1,33 | 2,36E-12 | 3,30E-09 | 0 | NA | NA | NO |
| 1038313 | 171 | 1,34 | 4,84E-07 | 1,26E-03 | 1,41 | 1,21E-07 | 2,22E-04 | NO |
| 1467586 | 1226 | 1,34 | 4,24E-09 | 8,39E-06 | 1,09 | 2,96E-06 | 6,72E-03 | NO |
| 1397132 | 145 | 1,35 | 9,65E-06 | 3,03E-02 | 0 | NA | NA | NO |
| 1466922 | 11519 | 1,35 | 4,07E-09 | 8,04E-06 | 1,54 | 1,94E-11 | 2,33E-08 | NO |
| 546151 | 1438 | 1,36 | 8,67E-08 | 2,03E-04 | 0 | NA | NA | NO |
| 1407562 | 1855 | 1,36 | 1,37E-15 | 1,36E-12 | 1 | 8,46E-09 | 1,34E-05 | NO |
| 1419737 | 809 | 1,36 | 3,70E-15 | 3,86E-12 | 1,24 | 9,09E-13 | 9,48E-10 | NO |
| 1435432 | 939 | 1,36 | 5,04E-09 | 1,01E-05 | 0 | NA | NA | NO |
| 256026 | 1088 | 1,37 | 2,66E-08 | 5,82E-05 | 0 | NA | NA | NO |
| 1442967 | 1579 | 1,37 | 1,07E-09 | 1,98E-06 | 0 | NA | NA | NO |
| 427404 | 171 | 1,38 | 3,05E-07 | 7,67E-04 | 2,15 | 1,77E-16 | 1,41E-13 | NO |
| 1434682 | 712 | 1,38 | 9,68E-11 | 1,59E-07 | 0,95 | 1,53E-05 | 3,90E-02 | NO |
| 1157188 | 167 | 1,39 | 1,64E-06 | 4,63E-03 | 0 | NA | NA | NO |
| 1358761 | 1077 | 1,39 | 7,68E-08 | 1,78E-04 | 0 | NA | NA | NO |
| 1430960 | 24657 | 1,39 | 5,15E-06 | 1,55E-02 | 0 | NA | NA | NO |
| 1440164 | 6499 | 1,39 | 1,41E-08 | 2,99E-05 | 2,9 | 7,86E-34 | 2,13E-31 | NO |
| 1441066 | 753 | 1,39 | 2,75E-12 | 3,87E-09 | 1,14 | 1,53E-08 | 2,48E-05 | NO |
| 1475001 | 1715 | 1,39 | 6,41E-09 | 1,31E-05 | 1,23 | 3,86E-07 | 7,65E-04 | NO |
| 591428 | 451 | 1,4 | 6,90E-10 | 1,25E-06 | 1,17 | 3,71E-07 | 7,33E-04 | NO |
| 1376354 | 227 | 1,4 | 1,25E-05 | 4,00E-02 | 2,61 | 1,25E-17 | 9,10E-15 | NO |
| 1396414 | 502 | 1,4 | 3,88E-12 | 5,54E-09 | 0 | NA | NA | NO |
| 1439740 | 446 | 1,4 | 1,85E-13 | 2,29E-10 | 1,8 | 4,38E-22 | 2,31E-19 | NO |
| 930492 | 487 | 1,41 | 1,89E-06 | 5,35E-03 | 0 | NA | NA | NO |
| 1358286 | 146 | 1,41 | 8,99E-10 | 1,65E-06 | 0 | NA | NA | NO |
| 1437324 | 6639 | 1,41 | 1,53E-05 | 5,00E-02 | 2,92 | 4,53E-20 | 2,78E-17 | NO |
| 1440062 | 8313 | 1,41 | 8,31E-07 | 2,25E-03 | 0 | NA | NA | NO |
| 1471185 | 169 | 1,41 | 1,57E-07 | 3,78E-04 | 0 | NA | NA | NO |
| 147854 | 926 | 1,42 | 1,04E-05 | 3,29E-02 | 1,85 | 7,81E-09 | 1,22E-05 | NO |
| 1270594 | 86 | 1,42 | 6,51E-08 | 1,49E-04 | 0 | NA | NA | NO |
| 1478080 | 309 | 1,42 | 3,92E-09 | 7,73E-06 | 1,92 | 5,83E-16 | 4,81E-13 | NO |
| 1486914 | 2513 | 1,42 | 6,74E-08 | 1,55E-04 | 1,47 | 3,20E-08 | 5,45E-05 | NO |
| 1409615 | 24394 | 1,43 | 4,15E-10 | 7,32E-07 | 0 | NA | NA | NO |
| 1473793 | 242 | 1,43 | 5,67E-09 | 1,14E-05 | 1,46 | 2,55E-09 | 3,79E-06 | NO |
| 1376040 | 5208 | 1,44 | 2,57E-17 | 2,21E-14 | 1,19 | 4,72E-12 | 5,33E-09 | NO |
| 1433337 | 3662 | 1,44 | 2,24E-09 | 4,30E-06 | 1,32 | 5,07E-08 | 8,84E-05 | NO |
| 1467429 | 637 | 1,44 | 2,91E-07 | 7,33E-04 | 0 | NA | NA | NO |
| 1357648 | 2377 | 1,45 | 1,49E-07 | 3,59E-04 | 0 | NA | NA | NO |
| 1465509 | 1301 | 1,45 | 2,80E-07 | 7,03E-04 | 1,62 | 1,12E-08 | 1,80E-05 | NO |
| 166211 | 602 | 1,46 | 2,57E-08 | 5,59E-05 | 0 | NA | NA | NO |
| 1430325 | 342 | 1,46 | 1,57E-06 | 4,40E-03 | 0 | NA | NA | NO |
| 1441253 | 1886 | 1,47 | 1,46E-09 | 2,74E-06 | 0 | NA | NA | NO |
| 1478540 | 778 | 1,47 | 2,09E-06 | 5,97E-03 | 0 | NA | NA | NO |
| 163375 | 3814 | 1,48 | 2,48E-09 | 4,79E-06 | 1,15 | 6,77E-06 | 1,64E-02 | NO |
| 574122 | 50 | 1,48 | 3,57E-06 | 1,05E-02 | 0 | NA | NA | NO |
| 1387955 | 130 | 1,48 | 1,98E-07 | 4,85E-04 | 0 | NA | NA | NO |
| 1413628 | 19814 | 1,48 | 5,10E-14 | 5,89E-11 | 0 | NA | NA | NO |
| 1434019 | 4018 | 1,48 | 5,18E-15 | 5,48E-12 | 2,08 | 6,60E-29 | 2,34E-26 | NO |
| 1434945 | 1025 | 1,48 | 1,05E-10 | 1,74E-07 | 0 | NA | NA | NO |
| 1445248 | 188 | 1,48 | 3,85E-09 | 7,57E-06 | 1,15 | 7,85E-06 | 1,92E-02 | NO |
| 831070 | 625 | 1,49 | 5,12E-07 | 1,34E-03 | 0 | NA | NA | NO |
| 888731 | 4006 | 1,49 | 8,29E-12 | 1,23E-08 | 0 | NA | NA | NO |
| 1393977 | 55 | 1,49 | 6,70E-06 | 2,05E-02 | 0 | NA | NA | NO |
| 1397131 | 1418 | 1,49 | 4,28E-08 | 9,62E-05 | 0 | NA | NA | NO |
| 1430686 | 9469 | 1,49 | 4,12E-07 | 1,06E-03 | 0 | NA | NA | NO |
| 169305 | 1383 | 1,5 | 2,20E-07 | 5,43E-04 | 0 | NA | NA | NO |
| 1373230 | 1181 | 1,5 | 2,96E-06 | 8,62E-03 | 2,04 | 1,33E-10 | 1,72E-07 | NO |
| 1413425 | 3564 | 1,5 | 1,87E-10 | 3,18E-07 | 0 | NA | NA | NO |
| 1413780 | 4345 | 1,51 | 1,90E-07 | 4,64E-04 | 1,51 | 2,47E-07 | 4,73E-04 | NO |
| 747779 | 449 | 1,52 | 3,83E-07 | 9,80E-04 | 0 | NA | NA | NO |
| 1392020 | 2728 | 1,52 | 3,49E-08 | 7,76E-05 | 0 | NA | NA | NO |
| 1037577 | 181 | 1,53 | 1,22E-07 | 2,90E-04 | 1,4 | 1,61E-06 | 3,53E-03 | NO |
| 1372417 | 747 | 1,53 | 3,07E-06 | 8,96E-03 | 2,7 | 3,04E-17 | 2,27E-14 | NO |
| 1450178 | 14560 | 1,53 | 5,60E-11 | 9,01E-08 | 0 | NA | NA | NO |
| 1478352 | 5173 | 1,53 | 2,57E-15 | 2,63E-12 | 1,39 | 1,14E-12 | 1,21E-09 | NO |
| 1479940 | 2422 | 1,53 | 1,65E-09 | 3,11E-06 | 1,13 | 1,51E-05 | 3,86E-02 | NO |
| 511875 | 140 | 1,54 | 1,50E-06 | 4,19E-03 | 2,62 | 1,68E-17 | 1,23E-14 | NO |
| 1149908 | 1038 | 1,54 | 1,10E-14 | 1,19E-11 | 1,05 | 2,41E-07 | 4,62E-04 | NO |
| 1178528 | 3407 | 1,54 | 1,33E-07 | 3,17E-04 | 1,66 | 1,24E-08 | 2,00E-05 | NO |
| 1359232 | 3981 | 1,54 | 1,16E-11 | 1,74E-08 | 1,38 | 1,63E-09 | 2,38E-06 | NO |
| 1378165 | 132 | 1,54 | 1,14E-06 | 3,13E-03 | 0 | NA | NA | NO |
| 1431773 | 3154 | 1,54 | 1,05E-15 | 1,04E-12 | 1,73 | 2,14E-19 | 1,39E-16 | NO |
| 784641 | 417 | 1,55 | 2,34E-06 | 6,74E-03 | 0 | NA | NA | NO |
| 1044651 | 865 | 1,55 | 4,40E-07 | 1,14E-03 | 2 | 5,48E-11 | 6,82E-08 | NO |
| 1369429 | 756 | 1,55 | 9,45E-08 | 2,22E-04 | 0 | NA | NA | NO |
| 1434904 | 71 | 1,55 | 1,37E-05 | 4,41E-02 | 2 | 7,86E-09 | 1,23E-05 | NO |
| 912878 | 1256 | 1,56 | 4,88E-08 | 1,10E-04 | 0 | NA | NA | NO |
| 1378223 | 503 | 1,56 | 2,27E-08 | 4,93E-05 | 1,78 | 1,68E-10 | 2,21E-07 | NO |
| 1446288 | 291 | 1,56 | 4,62E-06 | 1,39E-02 | 0 | NA | NA | NO |
| 359400 | 8308 | 1,57 | 2,50E-06 | 7,22E-03 | 2,58 | 3,97E-15 | 3,43E-12 | NO |
| 1399073 | 726 | 1,58 | 1,76E-16 | 1,62E-13 | 1,16 | 2,87E-09 | 4,30E-06 | NO |
| 1435561 | 222 | 1,58 | 1,40E-07 | 3,35E-04 | 1,52 | 5,93E-07 | 1,21E-03 | NO |
| 1466248 | 3243 | 1,58 | 9,46E-07 | 2,57E-03 | 0 | NA | NA | NO |
| 1477152 | 21819 | 1,58 | 1,74E-10 | 2,96E-07 | 0 | NA | NA | NO |
| 976354 | 302 | 1,59 | 1,10E-07 | 2,61E-04 | 3,31 | 6,26E-31 | 1,97E-28 | NO |
| 1432593 | 147 | 1,59 | 7,22E-08 | 1,67E-04 | 0 | NA | NA | NO |
| 759876 | 1970 | 1,6 | 1,63E-16 | 1,48E-13 | 1,02 | 2,94E-07 | 5,68E-04 | NO |
| 1138206 | 1418 | 1,6 | 5,00E-09 | 1,00E-05 | 0 | NA | NA | NO |
| 1383488 | 954 | 1,6 | 7,58E-16 | 7,39E-13 | 0 | NA | NA | NO |
| 1357121 | 4982 | 1,61 | 1,08E-12 | 1,45E-09 | 0 | NA | NA | NO |
| 1361559 | 261 | 1,61 | 7,91E-07 | 2,13E-03 | 0 | NA | NA | NO |
| 1431301 | 463 | 1,61 | 5,57E-10 | 9,97E-07 | 0 | NA | NA | NO |
| 1439097 | 5490 | 1,61 | 1,87E-12 | 2,58E-09 | 1,25 | 6,81E-08 | 1,20E-04 | NO |
| 1479530 | 545 | 1,61 | 8,27E-08 | 1,93E-04 | 0 | NA | NA | NO |
| 1464220 | 3951 | 1,62 | 8,49E-09 | 1,75E-05 | 2,75 | 2,97E-23 | 1,46E-20 | NO |
| 408431 | 989 | 1,63 | 5,60E-13 | 7,25E-10 | 1,42 | 4,75E-10 | 6,54E-07 | NO |
| 1429508 | 158 | 1,63 | 3,46E-09 | 6,79E-06 | 1,3 | 4,33E-06 | 1,01E-02 | NO |
| 1434927 | 1949 | 1,63 | 5,77E-15 | 6,12E-12 | 1,13 | 1,09E-07 | 1,98E-04 | NO |
| 1446270 | 5203 | 1,63 | 1,66E-10 | 2,81E-07 | 2,12 | 6,32E-17 | 4,84E-14 | NO |
| 1465389 | 77 | 1,63 | 2,03E-06 | 5,78E-03 | 2,09 | 4,36E-10 | 5,96E-07 | NO |
| 456019 | 711 | 1,64 | 1,91E-10 | 3,25E-07 | 2,13 | 6,18E-17 | 4,73E-14 | NO |
| 896460 | 553 | 1,64 | 1,74E-11 | 2,67E-08 | 1,23 | 7,52E-07 | 1,56E-03 | NO |
| 1241195 | 246 | 1,64 | 4,59E-09 | 9,15E-06 | 0 | NA | NA | NO |
| 1380269 | 1898 | 1,64 | 1,53E-10 | 2,59E-07 | 0 | NA | NA | NO |
| 1473592 | 6870 | 1,64 | 1,19E-13 | 1,43E-10 | 3,18 | 1,22E-48 | 1,48E-46 | NO |
| 1406112 | 187 | 1,65 | 2,76E-07 | 6,89E-04 | 0 | NA | NA | NO |
| 246973 | 1991 | 1,66 | 2,97E-15 | 3,06E-12 | 1,4 | 4,87E-11 | 6,03E-08 | NO |
| 1378401 | 510 | 1,66 | 1,42E-10 | 2,38E-07 | 1,55 | 3,50E-09 | 5,29E-06 | NO |
| 1472192 | 528 | 1,66 | 2,41E-06 | 6,95E-03 | 3,5 | 5,32E-25 | 2,32E-22 | NO |
| 552142 | 107 | 1,67 | 5,50E-07 | 1,44E-03 | 0 | NA | NA | NO |
| 1374087 | 63778 | 1,67 | 8,87E-13 | 1,17E-09 | 2,21 | 1,88E-21 | 1,04E-18 | NO |
| 13768 | 2921 | 1,68 | 3,07E-10 | 5,33E-07 | 0 | NA | NA | NO |
| 1365254 | 96 | 1,68 | 1,17E-06 | 3,23E-03 | 1,57 | 6,48E-06 | 1,56E-02 | NO |
| 1388999 | 1555 | 1,68 | 1,88E-13 | 2,32E-10 | 1,59 | 3,76E-12 | 4,20E-09 | NO |
| 1439873 | 5311 | 1,68 | 1,60E-10 | 2,70E-07 | 1,72 | 5,74E-11 | 7,16E-08 | NO |
| 1466958 | 153 | 1,68 | 1,75E-10 | 2,98E-07 | 0 | NA | NA | NO |
| 1471940 | 95 | 1,68 | 7,33E-06 | 2,26E-02 | 1,65 | 1,29E-05 | 3,25E-02 | NO |
| 1481052 | 3004 | 1,68 | 9,40E-13 | 1,25E-09 | 1,94 | 1,15E-16 | 8,97E-14 | NO |
| 1388183 | 822 | 1,69 | 1,67E-23 | 8,78E-21 | 2,24 | 3,25E-41 | 6,05E-39 | NO |
| 1463152 | 9238 | 1,69 | 1,90E-07 | 4,63E-04 | 2,66 | 8,42E-17 | 6,55E-14 | NO |
| 1470997 | 915 | 1,69 | 8,07E-10 | 1,47E-06 | 2,46 | 1,24E-19 | 7,92E-17 | NO |
| 1427000 | 233 | 1,7 | 1,83E-11 | 2,81E-08 | 1,53 | 2,00E-09 | 2,94E-06 | NO |
| 1429617 | 619 | 1,7 | 4,34E-07 | 1,12E-03 | 1,66 | 1,08E-06 | 2,30E-03 | NO |
| 1425172 | 109155 | 1,71 | 4,41E-07 | 1,14E-03 | 0 | NA | NA | NO |
| 1439697 | 115 | 1,71 | 1,96E-07 | 4,78E-04 | 0 | NA | NA | NO |
| 1450026 | 221 | 1,71 | 5,99E-10 | 1,08E-06 | 0 | NA | NA | NO |
| 832887 | 432 | 1,72 | 1,23E-08 | 2,59E-05 | 0 | NA | NA | NO |
| 1466366 | 789 | 1,72 | 1,84E-15 | 1,86E-12 | 1,68 | 7,94E-15 | 7,03E-12 | NO |
| 1383781 | 650 | 1,73 | 2,45E-16 | 2,26E-13 | 1,65 | 6,66E-15 | 5,86E-12 | NO |
| 1428593 | 11403 | 1,73 | 4,46E-07 | 1,16E-03 | 0 | NA | NA | NO |
| 1445390 | 955 | 1,73 | 3,81E-12 | 5,44E-09 | 1,51 | 2,12E-09 | 3,13E-06 | NO |
| 722587 | 579 | 1,74 | 9,09E-09 | 1,88E-05 | 1,71 | 1,85E-08 | 3,04E-05 | NO |
| 937238 | 93 | 1,74 | 9,21E-09 | 1,91E-05 | 2,12 | 6,92E-13 | 7,14E-10 | NO |
| 1430448 | 4014 | 1,74 | 5,50E-08 | 1,25E-04 | 0 | NA | NA | NO |
| 1434011 | 460 | 1,74 | 5,67E-09 | 1,14E-05 | 1,67 | 2,71E-08 | 4,56E-05 | NO |
| 32020 | 90 | 1,75 | 4,46E-07 | 1,16E-03 | 1,76 | 4,26E-07 | 8,48E-04 | NO |
| 304066 | 776 | 1,75 | 3,47E-12 | 4,92E-09 | 2,06 | 1,75E-16 | 1,39E-13 | NO |
| 410417 | 331 | 1,75 | 2,26E-06 | 6,48E-03 | 0 | NA | NA | NO |
| 1203984 | 1011 | 1,75 | 2,04E-12 | 2,83E-09 | 1,69 | 1,51E-11 | 1,79E-08 | NO |
| 1361397 | 49 | 1,75 | 7,10E-06 | 2,19E-02 | 0 | NA | NA | NO |
| 1432368 | 385 | 1,75 | 5,68E-11 | 9,14E-08 | 1,54 | 1,28E-08 | 2,07E-05 | NO |
| 1447680 | 59 | 1,75 | 1,72E-07 | 4,16E-04 | 0 | NA | NA | NO |
| 1204121 | 546 | 1,76 | 1,72E-10 | 2,92E-07 | 1,51 | 6,49E-08 | 1,15E-04 | NO |
| 1471780 | 432 | 1,76 | 2,51E-06 | 7,27E-03 | 0 | NA | NA | NO |
| 1302692 | 197 | 1,77 | 3,33E-10 | 5,83E-07 | 2,04 | 3,24E-13 | 3,24E-10 | NO |
| 83673 | 3120 | 1,78 | 9,45E-09 | 1,96E-05 | 0 | NA | NA | NO |
| 560466 | 1549 | 1,78 | 4,03E-16 | 3,81E-13 | 1,02 | 6,48E-06 | 1,56E-02 | NO |
| 1006568 | 108 | 1,78 | 7,84E-08 | 1,82E-04 | 2,11 | 1,04E-10 | 1,35E-07 | NO |
| 1080139 | 79 | 1,78 | 7,62E-07 | 2,05E-03 | 1,89 | 1,50E-07 | 2,75E-04 | NO |
| 1429787 | 5441 | 1,78 | 2,42E-09 | 4,67E-06 | 0 | NA | NA | NO |
| 1432736 | 780 | 1,78 | 3,70E-12 | 5,27E-09 | 1,98 | 8,03E-15 | 7,11E-12 | NO |
| 1442916 | 1688 | 1,78 | 6,55E-10 | 1,18E-06 | 1,91 | 4,11E-11 | 5,07E-08 | NO |
| 1469294 | 112 | 1,78 | 1,33E-07 | 3,18E-04 | 0 | NA | NA | NO |
| 649451 | 2173 | 1,79 | 1,88E-09 | 3,56E-06 | 0 | NA | NA | NO |
| 930009 | 1371 | 1,79 | 3,73E-11 | 5,92E-08 | 0 | NA | NA | NO |
| 1440380 | 154 | 1,79 | 1,57E-11 | 2,41E-08 | 1,74 | 6,91E-11 | 8,71E-08 | NO |
| 1467799 | 79 | 1,79 | 3,79E-07 | 9,70E-04 | 0 | NA | NA | NO |
| 1468931 | 527 | 1,79 | 1,02E-09 | 1,89E-06 | 1,38 | 4,11E-06 | 9,52E-03 | NO |
| 1376974 | 1544 | 1,8 | 9,00E-13 | 1,19E-09 | 1,76 | 3,51E-12 | 3,90E-09 | NO |
| 1429376 | 1302 | 1,8 | 1,31E-17 | 1,09E-14 | 1,65 | 5,57E-15 | 4,86E-12 | NO |
| 1438830 | 698 | 1,8 | 2,11E-15 | 2,15E-12 | 1,91 | 2,11E-17 | 1,55E-14 | NO |
| 1305915 | 67 | 1,81 | 1,12E-08 | 2,35E-05 | 0 | NA | NA | NO |
| 1381724 | 157 | 1,81 | 1,04E-05 | 3,29E-02 | 0 | NA | NA | NO |
| 1471562 | 2324 | 1,81 | 4,12E-08 | 9,24E-05 | 2,16 | 5,31E-11 | 6,58E-08 | NO |
| 1205993 | 208 | 1,82 | 2,10E-09 | 4,01E-06 | 0 | NA | NA | NO |
| 1372842 | 1197 | 1,82 | 4,22E-13 | 5,38E-10 | 1,81 | 5,74E-13 | 5,87E-10 | NO |
| 1382418 | 7847 | 1,82 | 4,69E-15 | 4,93E-12 | 1,21 | 3,66E-07 | 7,21E-04 | NO |
| 1415528 | 323 | 1,82 | 9,72E-11 | 1,60E-07 | 2,32 | 6,06E-17 | 4,63E-14 | NO |
| 1434974 | 1327 | 1,82 | 1,58E-10 | 2,68E-07 | 1,59 | 3,19E-08 | 5,42E-05 | NO |
| 1378128 | 445 | 1,83 | 3,59E-14 | 4,08E-11 | 0 | NA | NA | NO |
| 381063 | 790 | 1,84 | 1,55E-23 | 8,10E-21 | 0,97 | 3,31E-07 | 6,47E-04 | NO |
| 1437412 | 52425 | 1,84 | 6,77E-15 | 7,23E-12 | 1,55 | 9,06E-11 | 1,16E-07 | NO |
| 1431484 | 90 | 1,85 | 2,73E-07 | 6,81E-04 | 2,24 | 3,28E-10 | 4,42E-07 | NO |
| 1472708 | 50 | 1,85 | 1,09E-05 | 3,45E-02 | 1,85 | 1,14E-05 | 2,86E-02 | NO |
| 1481622 | 899 | 1,85 | 4,51E-15 | 4,73E-12 | 1,82 | 1,12E-14 | 1,01E-11 | NO |
| 1165383 | 261 | 1,86 | 1,79E-08 | 3,84E-05 | 0 | NA | NA | NO |
| 1474773 | 410 | 1,86 | 2,25E-14 | 2,52E-11 | 0 | NA | NA | NO |
| 1471011 | 1133 | 1,88 | 1,20E-10 | 2,01E-07 | 2,92 | 2,61E-24 | 1,20E-21 | NO |
| 1219506 | 1003 | 1,89 | 1,01E-12 | 1,35E-09 | 0 | NA | NA | NO |
| 747888 | 84 | 1,9 | 1,03E-08 | 2,14E-05 | 0 | NA | NA | NO |
| 1454428 | 2426 | 1,9 | 5,94E-31 | 1,81E-28 | 2,16 | 8,37E-40 | 1,64E-37 | NO |
| 1463849 | 5349 | 1,9 | 1,06E-13 | 1,26E-10 | 1,45 | 2,63E-08 | 4,40E-05 | NO |
| 1477236 | 484 | 1,91 | 4,71E-09 | 9,39E-06 | 0 | NA | NA | NO |
| 1436702 | 642 | 1,92 | 5,37E-20 | 3,63E-17 | 2,36 | 5,48E-30 | 1,80E-27 | NO |
| 746229 | 2620 | 1,93 | 1,77E-15 | 1,78E-12 | 0 | NA | NA | NO |
| 1456372 | 341 | 1,93 | 7,31E-12 | 1,08E-08 | 0 | NA | NA | NO |
| 1465949 | 705 | 1,93 | 1,02E-18 | 7,61E-16 | 1,95 | 5,38E-19 | 3,57E-16 | NO |
| 1379537 | 140 | 1,94 | 3,90E-09 | 7,68E-06 | 0 | NA | NA | NO |
| 1429357 | 709 | 1,94 | 1,03E-10 | 1,70E-07 | 1,67 | 4,70E-08 | 8,15E-05 | NO |
| 1398543 | 162 | 1,95 | 8,31E-14 | 9,82E-11 | 1,85 | 1,53E-12 | 1,64E-09 | NO |
| 322268 | 145 | 1,96 | 2,15E-09 | 4,12E-06 | 2,28 | 1,79E-12 | 1,94E-09 | NO |
| 1356993 | 937 | 1,96 | 2,88E-16 | 2,69E-13 | 1,75 | 3,97E-13 | 3,99E-10 | NO |
| 301897 | 1643 | 1,97 | 1,33E-15 | 1,32E-12 | 1,38 | 4,52E-08 | 7,82E-05 | NO |
| 1232370 | 1040 | 1,97 | 3,24E-11 | 5,10E-08 | 2,1 | 1,69E-12 | 1,81E-09 | NO |
| 1426486 | 2545 | 1,98 | 7,13E-09 | 1,46E-05 | 0 | NA | NA | NO |
| 1438178 | 4167 | 1,98 | 1,83E-17 | 1,55E-14 | 2,33 | 9,13E-24 | 4,29E-21 | NO |
| 828192 | 307 | 1,99 | 4,91E-15 | 5,18E-12 | 1,85 | 4,84E-13 | 4,92E-10 | NO |
| 1205761 | 55 | 1,99 | 4,91E-06 | 1,48E-02 | 0 | NA | NA | NO |
| 1372865 | 15239 | 1,99 | 1,73E-08 | 3,69E-05 | 0 | NA | NA | NO |
| 1435362 | 155 | 1,99 | 3,96E-11 | 6,30E-08 | 2,46 | 1,09E-16 | 8,55E-14 | NO |
| 1441180 | 178 | 1,99 | 2,59E-11 | 4,03E-08 | 1,39 | 5,89E-06 | 1,40E-02 | NO |
| 1277067 | 446 | 2,01 | 2,30E-07 | 5,67E-04 | 0 | NA | NA | NO |
| 1393643 | 4907 | 2,01 | 3,60E-15 | 3,75E-12 | 1,53 | 3,88E-09 | 5,91E-06 | NO |
| 1463413 | 161 | 2,01 | 7,01E-07 | 1,87E-03 | 0 | NA | NA | NO |
| 1472280 | 1105 | 2,02 | 1,03E-12 | 1,37E-09 | 0 | NA | NA | NO |
| 1425056 | 5239 | 2,03 | 2,01E-13 | 2,48E-10 | 0 | NA | NA | NO |
| 375970 | 278 | 2,04 | 3,69E-07 | 9,42E-04 | 1,78 | 1,33E-05 | 3,36E-02 | NO |
| 383804 | 5636 | 2,04 | 1,75E-10 | 2,97E-07 | 0 | NA | NA | NO |
| 1130331 | 172 | 2,04 | 1,26E-08 | 2,65E-05 | 0 | NA | NA | NO |
| 1438280 | 120 | 2,04 | 2,74E-07 | 6,85E-04 | 0 | NA | NA | NO |
| 1445885 | 5555 | 2,04 | 1,69E-17 | 1,43E-14 | 1,23 | 5,68E-07 | 1,16E-03 | NO |
| 11109 | 333 | 2,05 | 3,52E-17 | 3,05E-14 | 1,83 | 6,96E-14 | 6,62E-11 | NO |
| 1438179 | 2137 | 2,05 | 1,05E-13 | 1,25E-10 | 2,51 | 4,91E-20 | 3,02E-17 | NO |
| 1440221 | 19808 | 2,05 | 2,84E-17 | 2,46E-14 | 1,4 | 1,72E-08 | 2,83E-05 | NO |
| 1470062 | 2324 | 2,05 | 1,04E-35 | 2,27E-33 | 1,9 | 4,77E-31 | 1,49E-28 | NO |
| 1254137 | 1164 | 2,06 | 6,27E-19 | 4,60E-16 | 1,94 | 7,68E-17 | 5,94E-14 | NO |
| 1001730 | 43 | 2,07 | 2,90E-06 | 8,46E-03 | 2,94 | 4,51E-12 | 5,09E-09 | NO |
| 1410050 | 231 | 2,07 | 1,72E-11 | 2,63E-08 | 1,86 | 2,33E-09 | 3,45E-06 | NO |
| 1431721 | 11752 | 2,07 | 9,79E-15 | 1,06E-11 | 1,5 | 3,74E-08 | 6,42E-05 | NO |
| 276499 | 850 | 2,08 | 2,30E-07 | 5,68E-04 | 0 | NA | NA | NO |
| 734985 | 743 | 2,08 | 3,08E-13 | 3,87E-10 | 3,91 | 6,64E-45 | 1,02E-42 | NO |
| 1382897 | 1738 | 2,08 | 2,22E-18 | 1,73E-15 | 1,83 | 1,68E-14 | 1,53E-11 | NO |
| 1468023 | 129 | 2,08 | 6,97E-09 | 1,42E-05 | 2,53 | 8,10E-13 | 8,39E-10 | NO |
| 1359272 | 394 | 2,09 | 3,54E-08 | 7,86E-05 | 0 | NA | NA | NO |
| 1429990 | 51 | 2,09 | 1,33E-05 | 4,27E-02 | 0 | NA | NA | NO |
| 1446174 | 121 | 2,09 | 1,47E-09 | 2,76E-06 | 1,89 | 5,91E-08 | 1,04E-04 | NO |
| 1340728 | 169 | 2,1 | 1,17E-06 | 3,21E-03 | 0 | NA | NA | NO |
| 1441034 | 49 | 2,1 | 8,99E-08 | 2,11E-04 | 2,06 | 1,75E-07 | 3,25E-04 | NO |
| 1444915 | 226 | 2,1 | 1,61E-18 | 1,24E-15 | 2,03 | 1,94E-17 | 1,42E-14 | NO |
| 1435843 | 269 | 2,11 | 2,56E-15 | 2,62E-12 | 0 | NA | NA | NO |
| 1437675 | 2035 | 2,11 | 3,08E-23 | 1,65E-20 | 2,53 | 5,53E-33 | 1,55E-30 | NO |
| 1439079 | 8265 | 2,11 | 1,29E-10 | 2,17E-07 | 0 | NA | NA | NO |
| 165797 | 53 | 2,12 | 8,41E-06 | 2,61E-02 | 0 | NA | NA | NO |
| 279064 | 1644 | 2,12 | 8,08E-12 | 1,20E-08 | 1,69 | 7,61E-08 | 1,35E-04 | NO |
| 552184 | 4250 | 2,12 | 1,34E-20 | 8,64E-18 | 1,29 | 3,05E-08 | 5,17E-05 | NO |
| 1459264 | 442 | 2,12 | 9,75E-18 | 7,98E-15 | 2,32 | 3,78E-21 | 2,14E-18 | NO |
| 606841 | 1607 | 2,14 | 8,06E-12 | 1,19E-08 | 0 | NA | NA | NO |
| 1435338 | 73 | 2,14 | 2,69E-06 | 7,81E-03 | 0 | NA | NA | NO |
| 283678 | 92 | 2,15 | 1,34E-08 | 2,84E-05 | 2,25 | 2,91E-09 | 4,37E-06 | NO |
| 1439649 | 54 | 2,15 | 4,57E-07 | 1,19E-03 | 2,44 | 6,40E-09 | 9,93E-06 | NO |
| 1469729 | 420 | 2,15 | 4,92E-12 | 7,10E-09 | 2,08 | 3,28E-11 | 4,02E-08 | NO |
| 258510 | 5088 | 2,16 | 2,52E-12 | 3,54E-09 | 0 | NA | NA | NO |
| 1365351 | 411 | 2,16 | 2,22E-12 | 3,08E-09 | 0 | NA | NA | NO |
| 1437238 | 125 | 2,16 | 5,46E-10 | 9,77E-07 | 0 | NA | NA | NO |
| 475600 | 62 | 2,17 | 4,93E-09 | 9,86E-06 | 0 | NA | NA | NO |
| 857219 | 3917 | 2,17 | 4,51E-18 | 3,62E-15 | 1,74 | 8,06E-12 | 9,31E-09 | NO |
| 1145966 | 55 | 2,17 | 5,71E-07 | 1,50E-03 | 2,42 | 1,69E-08 | 2,76E-05 | NO |
| 1156268 | 1060 | 2,17 | 3,89E-31 | 1,16E-28 | 2,12 | 8,49E-30 | 2,85E-27 | NO |
| 1405721 | 159 | 2,17 | 1,07E-10 | 1,77E-07 | 3,31 | 3,87E-25 | 1,66E-22 | NO |
| 1465716 | 102 | 2,17 | 1,90E-09 | 3,62E-06 | 2,19 | 1,57E-09 | 2,28E-06 | NO |
| 1195034 | 1887 | 2,18 | 5,20E-16 | 5,01E-13 | 1,92 | 1,58E-12 | 1,70E-09 | NO |
| 1461440 | 146 | 2,18 | 5,85E-07 | 1,54E-03 | 0 | NA | NA | NO |
| 1465854 | 3624 | 2,18 | 4,61E-10 | 8,18E-07 | 2,39 | 9,95E-12 | 1,16E-08 | NO |
| 1428035 | 3543 | 2,19 | 2,32E-13 | 2,88E-10 | 0 | NA | NA | NO |
| 1459112 | 36 | 2,19 | 2,08E-06 | 5,93E-03 | 2,12 | 4,87E-06 | 1,14E-02 | NO |
| 1463566 | 3057 | 2,19 | 5,74E-06 | 1,74E-02 | 0 | NA | NA | NO |
| 1389340 | 4694 | 2,2 | 2,54E-09 | 4,92E-06 | 2,2 | 3,26E-09 | 4,92E-06 | NO |
| 1434740 | 488 | 2,2 | 1,72E-16 | 1,57E-13 | 3,04 | 6,26E-31 | 1,98E-28 | NO |
| 1438174 | 1375 | 2,2 | 1,16E-12 | 1,56E-09 | 0 | NA | NA | NO |
| 1471050 | 331 | 2,2 | 2,83E-11 | 4,44E-08 | 0 | NA | NA | NO |
| 1296164 | 249 | 2,21 | 1,52E-08 | 3,24E-05 | 0 | NA | NA | NO |
| 1389393 | 25833 | 2,21 | 4,59E-21 | 2,87E-18 | 1,84 | 7,45E-15 | 6,58E-12 | NO |
| 1439965 | 4403 | 2,21 | 6,69E-20 | 4,56E-17 | 1,96 | 9,12E-16 | 7,61E-13 | NO |
| 1465392 | 76 | 2,21 | 2,46E-10 | 4,22E-07 | 2,12 | 1,25E-09 | 1,80E-06 | NO |
| 11138 | 4222 | 2,24 | 1,38E-28 | 5,13E-26 | 1,96 | 3,84E-22 | 2,00E-19 | NO |
| 691220 | 566 | 2,25 | 4,76E-12 | 6,86E-09 | 1,77 | 1,04E-07 | 1,88E-04 | NO |
| 1429227 | 256 | 2,25 | 4,04E-14 | 4,63E-11 | 1,59 | 1,92E-07 | 3,58E-04 | NO |
| 1439325 | 7292 | 2,25 | 4,53E-09 | 9,01E-06 | 2,04 | 1,43E-07 | 2,63E-04 | NO |
| 1441091 | 675 | 2,25 | 3,12E-18 | 2,47E-15 | 1,93 | 1,23E-13 | 1,20E-10 | NO |
| 1436801 | 454 | 2,26 | 6,65E-11 | 1,08E-07 | 0 | NA | NA | NO |
| 1435538 | 7196 | 2,27 | 5,26E-13 | 6,77E-10 | 0 | NA | NA | NO |
| 1477385 | 8507 | 2,27 | 6,18E-30 | 2,08E-27 | 3,06 | 7,11E-54 | 6,47E-52 | NO |
| 1482029 | 83 | 2,27 | 2,36E-07 | 5,86E-04 | 2,18 | 8,99E-07 | 1,89E-03 | NO |
| 1367850 | 1159 | 2,28 | 2,88E-13 | 3,61E-10 | 2,46 | 3,39E-15 | 2,92E-12 | NO |
| 919508 | 117 | 2,29 | 7,02E-15 | 7,50E-12 | 0 | NA | NA | NO |
| 1385320 | 18 | 2,29 | 1,16E-05 | 3,69E-02 | 0 | NA | NA | NO |
| 1478585 | 481 | 2,3 | 2,33E-21 | 1,42E-18 | 2,34 | 3,85E-22 | 2,01E-19 | NO |
| 582723 | 106 | 2,31 | 1,75E-07 | 4,25E-04 | 0 | NA | NA | NO |
| 1435485 | 13412 | 2,32 | 9,97E-18 | 8,17E-15 | 2,38 | 1,10E-18 | 7,47E-16 | NO |
| 1444197 | 266 | 2,32 | 1,12E-15 | 1,10E-12 | 2,75 | 6,95E-22 | 3,73E-19 | NO |
| 699446 | 850 | 2,33 | 5,29E-16 | 5,10E-13 | 1,3 | 1,23E-05 | 3,10E-02 | NO |
| 1055086 | 1229 | 2,33 | 4,95E-21 | 3,12E-18 | 1,86 | 9,19E-14 | 8,85E-11 | NO |
| 173699 | 1076 | 2,34 | 6,48E-19 | 4,77E-16 | 1,87 | 2,59E-12 | 2,84E-09 | NO |
| 1419696 | 106 | 2,34 | 4,59E-09 | 9,13E-06 | 2,5 | 3,49E-10 | 4,73E-07 | NO |
| 1434773 | 12062 | 2,34 | 4,73E-18 | 3,80E-15 | 2,3 | 2,12E-17 | 1,56E-14 | NO |
| 1448556 | 13336 | 2,34 | 1,68E-12 | 2,30E-09 | 9,13 | 2,18E-179 | 2,18E-179 | NO |
| 1430831 | 3473 | 2,36 | 3,69E-16 | 3,47E-13 | 2,5 | 6,17E-18 | 4,37E-15 | NO |
| 1357952 | 118 | 2,38 | 2,98E-10 | 5,18E-07 | 0 | NA | NA | NO |
| 1447729 | 30 | 2,39 | 2,39E-07 | 5,94E-04 | 0 | NA | NA | NO |
| 1469154 | 33 | 2,39 | 9,77E-08 | 2,31E-04 | 2,39 | 9,78E-08 | 1,76E-04 | NO |
| 1469278 | 394 | 2,39 | 5,13E-20 | 3,46E-17 | 1,84 | 3,53E-12 | 3,93E-09 | NO |
| 1371133 | 263 | 2,4 | 7,02E-14 | 8,25E-11 | 4,22 | 2,39E-42 | 4,29E-40 | NO |
| 1454573 | 2223 | 2,41 | 1,41E-16 | 1,28E-13 | 1,45 | 1,22E-06 | 2,63E-03 | NO |
| 1427883 | 1477 | 2,42 | 1,34E-33 | 3,38E-31 | 2,4 | 3,41E-33 | 9,51E-31 | NO |
| 1481999 | 59 | 2,42 | 8,26E-12 | 1,23E-08 | 0 | NA | NA | NO |
| 1433904 | 416 | 2,43 | 2,92E-15 | 3,00E-12 | 0 | NA | NA | NO |
| 1464131 | 413 | 2,43 | 2,13E-24 | 1,06E-21 | 2,44 | 9,69E-25 | 4,30E-22 | NO |
| 1473318 | 2075 | 2,43 | 7,01E-17 | 6,21E-14 | 2,77 | 1,27E-21 | 6,88E-19 | NO |
| 362268 | 1290 | 2,45 | 1,07E-18 | 8,03E-16 | 1,86 | 3,83E-11 | 4,71E-08 | NO |
| 1245600 | 49 | 2,45 | 6,10E-07 | 1,62E-03 | 2,16 | 1,58E-05 | 4,04E-02 | NO |
| 1386616 | 12690 | 2,45 | 3,13E-40 | 5,07E-38 | 1,61 | 4,89E-18 | 3,45E-15 | NO |
| 1352092 | 1383 | 2,46 | 3,86E-37 | 7,80E-35 | 1,88 | 4,40E-22 | 2,32E-19 | NO |
| 1475124 | 111 | 2,48 | 2,93E-15 | 3,01E-12 | 2,1 | 4,01E-11 | 4,95E-08 | NO |
| 1435476 | 2621 | 2,49 | 2,35E-21 | 1,43E-18 | 2,06 | 8,14E-15 | 7,22E-12 | NO |
| 1467006 | 350 | 2,49 | 1,80E-13 | 2,21E-10 | 2,33 | 8,38E-12 | 9,70E-09 | NO |
| 1377468 | 608 | 2,5 | 8,16E-12 | 1,21E-08 | 2,15 | 6,60E-09 | 1,03E-05 | NO |
| 328939 | 195 | 2,51 | 1,47E-12 | 2,00E-09 | 2,12 | 3,44E-09 | 5,20E-06 | NO |
| 1435950 | 43 | 2,51 | 1,78E-08 | 3,81E-05 | 2,93 | 2,36E-11 | 2,86E-08 | NO |
| 1470904 | 8026 | 2,51 | 2,21E-26 | 9,53E-24 | 1,96 | 2,35E-16 | 1,89E-13 | NO |
| 1200522 | 233 | 2,52 | 2,48E-20 | 1,63E-17 | 2,11 | 2,19E-14 | 2,01E-11 | NO |
| 1442900 | 59 | 2,52 | 6,04E-10 | 1,08E-06 | 2,33 | 1,38E-08 | 2,23E-05 | NO |
| 1479775 | 1109 | 2,52 | 8,57E-27 | 3,59E-24 | 2,2 | 1,33E-20 | 7,90E-18 | NO |
| 918926 | 1525 | 2,53 | 4,84E-26 | 2,15E-23 | 1,77 | 3,82E-13 | 3,84E-10 | NO |
| 1457875 | 338 | 2,54 | 3,07E-07 | 7,74E-04 | 0 | NA | NA | NO |
| 1417840 | 124 | 2,55 | 1,82E-08 | 3,90E-05 | 0 | NA | NA | NO |
| 1437003 | 236 | 2,55 | 3,07E-13 | 3,87E-10 | 0 | NA | NA | NO |
| 1430008 | 538 | 2,56 | 3,42E-14 | 3,88E-11 | 4,5 | 2,11E-42 | 3,72E-40 | NO |
| 1468123 | 1286 | 2,56 | 6,97E-17 | 6,16E-14 | 1,52 | 1,40E-06 | 3,05E-03 | NO |
| 1474983 | 9572 | 2,56 | 8,62E-22 | 5,09E-19 | 1,72 | 2,39E-10 | 3,18E-07 | NO |
| 1440406 | 1324 | 2,58 | 8,45E-10 | 1,54E-06 | 0 | NA | NA | NO |
| 1254437 | 2220 | 2,59 | 3,62E-26 | 1,59E-23 | 2,4 | 1,27E-22 | 6,54E-20 | NO |
| 1362360 | 8395 | 2,59 | 7,15E-31 | 2,21E-28 | 2,37 | 4,80E-26 | 1,98E-23 | NO |
| 1436334 | 8269 | 2,59 | 4,79E-24 | 2,41E-21 | 2,17 | 3,56E-17 | 2,68E-14 | NO |
| 1459843 | 1819 | 2,59 | 2,74E-42 | 4,00E-40 | 2,63 | 1,13E-43 | 1,86E-41 | NO |
| 740571 | 878 | 2,6 | 8,07E-12 | 1,20E-08 | 2,16 | 2,33E-08 | 3,88E-05 | NO |
| 1436483 | 8666 | 2,6 | 3,09E-19 | 2,22E-16 | 1,91 | 9,00E-11 | 1,15E-07 | NO |
| 1482015 | 97 | 2,6 | 5,43E-13 | 7,01E-10 | 2,57 | 9,78E-13 | 1,02E-09 | NO |
| 878073 | 73 | 2,61 | 3,40E-09 | 6,64E-06 | 3,09 | 8,74E-13 | 9,10E-10 | NO |
| 665627 | 43 | 2,62 | 6,97E-07 | 1,86E-03 | 0 | NA | NA | NO |
| 959205 | 3877 | 2,62 | 2,17E-08 | 4,69E-05 | 0 | NA | NA | NO |
| 1435835 | 4811 | 2,62 | 1,69E-17 | 1,43E-14 | 3,01 | 9,43E-23 | 4,82E-20 | NO |
| 1440985 | 3197 | 2,62 | 1,51E-15 | 1,50E-12 | 4,26 | 1,29E-39 | 2,58E-37 | NO |
| 1467115 | 854 | 2,62 | 1,31E-25 | 5,96E-23 | 2,66 | 1,77E-26 | 7,19E-24 | NO |
| 9658 | 313 | 2,64 | 2,32E-12 | 3,23E-09 | 3 | 1,15E-15 | 9,67E-13 | NO |
| 844603 | 277 | 2,64 | 1,26E-17 | 1,05E-14 | 3,27 | 3,81E-27 | 1,47E-24 | NO |
| 1357854 | 1256 | 2,64 | 8,76E-10 | 1,60E-06 | 0 | NA | NA | NO |
| 1466810 | 12481 | 2,64 | 4,08E-33 | 1,08E-30 | 2,38 | 4,72E-27 | 1,83E-24 | NO |
| 928730 | 6141 | 2,65 | 4,75E-32 | 1,33E-29 | 1,84 | 5,64E-16 | 4,64E-13 | NO |
| 1084 | 223 | 2,66 | 3,13E-16 | 2,92E-13 | 3,97 | 6,10E-36 | 1,47E-33 | NO |
| 1403165 | 207 | 2,66 | 3,43E-09 | 6,71E-06 | 4,49 | 9,74E-25 | 4,33E-22 | NO |
| 1479288 | 1168 | 2,66 | 3,63E-08 | 8,10E-05 | 3,57 | 9,29E-14 | 8,96E-11 | NO |
| 1415569 | 870 | 2,67 | 2,48E-12 | 3,47E-09 | 2,28 | 3,50E-09 | 5,30E-06 | NO |
| 1259472 | 34 | 2,68 | 1,40E-06 | 3,88E-03 | 0 | NA | NA | NO |
| 1458413 | 348 | 2,68 | 3,58E-11 | 5,66E-08 | 3,65 | 7,50E-20 | 4,67E-17 | NO |
| 1347963 | 10199 | 2,69 | 5,85E-15 | 6,22E-12 | 2,1 | 1,87E-09 | 2,75E-06 | NO |
| 1437345 | 41 | 2,69 | 7,30E-08 | 1,68E-04 | 0 | NA | NA | NO |
| 1434108 | 159 | 2,7 | 2,96E-10 | 5,14E-07 | 2,2 | 5,25E-07 | 1,06E-03 | NO |
| 1473478 | 2131 | 2,7 | 9,08E-22 | 5,39E-19 | 3,61 | 4,05E-38 | 8,54E-36 | NO |
| 1285430 | 44 | 2,72 | 6,81E-13 | 8,90E-10 | 2,17 | 2,20E-08 | 3,66E-05 | NO |
| 1037199 | 365 | 2,73 | 2,87E-14 | 3,23E-11 | 2,11 | 8,69E-09 | 1,38E-05 | NO |
| 193198 | 138 | 2,74 | 1,09E-08 | 2,27E-05 | 0 | NA | NA | NO |
| 649962 | 5790 | 2,74 | 9,29E-14 | 1,10E-10 | 4,65 | 8,22E-38 | 1,76E-35 | NO |
| 757596 | 369 | 2,74 | 5,90E-18 | 4,77E-15 | 2,42 | 4,32E-14 | 4,03E-11 | NO |
| 28189 | 8024 | 2,75 | 1,91E-25 | 8,93E-23 | 2,2 | 1,20E-16 | 9,40E-14 | NO |
| 1288894 | 335 | 2,75 | 1,28E-30 | 4,07E-28 | 2,69 | 1,88E-29 | 6,47E-27 | NO |
| 1424317 | 1138 | 2,75 | 1,12E-23 | 5,81E-21 | 2,59 | 4,48E-21 | 2,55E-18 | NO |
| 1432371 | 307 | 2,75 | 1,10E-10 | 1,84E-07 | 3,55 | 4,36E-17 | 3,31E-14 | NO |
| 1435062 | 134 | 2,75 | 1,00E-14 | 1,09E-11 | 3,08 | 1,57E-18 | 1,08E-15 | NO |
| 1462983 | 4619 | 2,75 | 2,86E-14 | 3,22E-11 | 2,39 | 6,40E-11 | 8,02E-08 | NO |
| 1426277 | 5297 | 2,76 | 4,97E-19 | 3,62E-16 | 1,59 | 5,86E-07 | 1,20E-03 | NO |
| 1434367 | 15047 | 2,76 | 1,03E-25 | 4,65E-23 | 2,28 | 6,71E-18 | 4,76E-15 | NO |
| 118070 | 660 | 2,77 | 1,30E-13 | 1,57E-10 | 2,2 | 7,58E-09 | 1,19E-05 | NO |
| 1463008 | 2113 | 2,77 | 1,01E-25 | 4,55E-23 | 3,24 | 4,22E-35 | 1,08E-32 | NO |
| 1464950 | 8650 | 2,77 | 1,08E-51 | 9,37E-50 | 2,25 | 2,21E-34 | 5,84E-32 | NO |
| 1149518 | 351 | 2,78 | 1,82E-29 | 6,46E-27 | 3,04 | 1,85E-35 | 4,66E-33 | NO |
| 1157395 | 2145 | 2,78 | 2,38E-32 | 6,61E-30 | 2,28 | 4,47E-22 | 2,36E-19 | NO |
| 1478755 | 1687 | 2,79 | 1,14E-30 | 3,61E-28 | 2,13 | 3,78E-18 | 2,64E-15 | NO |
| 933199 | 177 | 2,8 | 3,37E-19 | 2,43E-16 | 3,27 | 2,65E-26 | 1,08E-23 | NO |
| 1414740 | 92 | 2,8 | 5,90E-12 | 8,60E-09 | 3 | 1,32E-13 | 1,29E-10 | NO |
| 1481807 | 352 | 2,8 | 1,12E-15 | 1,11E-12 | 2,36 | 2,54E-11 | 3,08E-08 | NO |
| 695259 | 680 | 2,81 | 6,87E-28 | 2,69E-25 | 2,64 | 1,04E-24 | 4,63E-22 | NO |
| 1104708 | 1519 | 2,83 | 1,03E-24 | 5,05E-22 | 2,39 | 8,82E-18 | 6,32E-15 | NO |
| 1435313 | 53 | 2,83 | 1,97E-10 | 3,36E-07 | 2,06 | 7,58E-06 | 1,85E-02 | NO |
| 1440946 | 3151 | 2,83 | 1,23E-17 | 1,02E-14 | 2,35 | 2,15E-12 | 2,33E-09 | NO |
| 1451417 | 11261 | 2,83 | 3,48E-19 | 2,51E-16 | 4,15 | 4,02E-40 | 7,76E-38 | NO |
| 605309 | 186 | 2,84 | 5,17E-25 | 2,49E-22 | 2,05 | 2,82E-13 | 2,81E-10 | NO |
| 1296963 | 26073 | 2,84 | 2,60E-21 | 1,59E-18 | 2,24 | 1,35E-13 | 1,32E-10 | NO |
| 1470339 | 687 | 2,84 | 7,90E-16 | 7,72E-13 | 3,27 | 1,09E-20 | 6,40E-18 | NO |
| 1442548 | 98 | 2,85 | 6,30E-14 | 7,34E-11 | 3,29 | 1,45E-18 | 9,91E-16 | NO |
| 734033 | 2800 | 2,86 | 4,39E-19 | 3,18E-16 | 1,93 | 3,40E-09 | 5,13E-06 | NO |
| 667616 | 95 | 2,87 | 1,82E-14 | 2,01E-11 | 2,71 | 5,53E-13 | 5,65E-10 | NO |
| 868398 | 3363 | 2,88 | 9,40E-31 | 2,94E-28 | 2,84 | 6,64E-30 | 2,19E-27 | NO |
| 1277099 | 348 | 2,88 | 6,95E-27 | 2,89E-24 | 3,28 | 8,89E-35 | 2,33E-32 | NO |
| 1417917 | 44 | 2,89 | 8,21E-15 | 8,83E-12 | 1,9 | 1,05E-06 | 2,23E-03 | NO |
| 336992 | 68 | 2,9 | 9,82E-08 | 2,32E-04 | 0 | NA | NA | NO |
| 1438874 | 4512 | 2,9 | 2,34E-20 | 1,53E-17 | 4,38 | 2,35E-45 | 3,49E-43 | NO |
| 1437769 | 1367 | 2,91 | 1,51E-26 | 6,45E-24 | 1,32 | 3,43E-06 | 7,84E-03 | NO |
| 1475335 | 111 | 2,91 | 1,97E-08 | 4,24E-05 | 0 | NA | NA | NO |
| 805226 | 207 | 2,93 | 2,09E-12 | 2,88E-09 | 0 | NA | NA | NO |
| 1012603 | 164 | 2,94 | 1,03E-10 | 1,69E-07 | 2,81 | 8,09E-10 | 1,13E-06 | NO |
| 1484590 | 3873 | 2,94 | 1,38E-17 | 1,15E-14 | 1,73 | 1,14E-06 | 2,44E-03 | NO |
| 608653 | 21 | 2,95 | 3,19E-06 | 9,34E-03 | 3 | 2,17E-06 | 4,84E-03 | NO |
| 1260776 | 1167 | 2,96 | 1,07E-27 | 4,27E-25 | 3,4 | 1,37E-36 | 3,19E-34 | NO |
| 1376810 | 1883 | 2,96 | 8,57E-25 | 4,16E-22 | 1,49 | 5,40E-07 | 1,09E-03 | NO |
| 786731 | 20 | 2,97 | 3,24E-06 | 9,51E-03 | 0 | NA | NA | NO |
| 996145 | 21 | 2,97 | 2,29E-06 | 6,58E-03 | 0 | NA | NA | NO |
| 488736 | 397 | 2,98 | 3,19E-16 | 2,99E-13 | 2,92 | 1,42E-15 | 1,20E-12 | NO |
| 1417700 | 452 | 2,98 | 4,77E-17 | 4,18E-14 | 1,73 | 2,46E-06 | 5,53E-03 | NO |
| 265269 | 392 | 2,99 | 2,13E-23 | 1,13E-20 | 1,38 | 1,06E-05 | 2,64E-02 | NO |
| 943815 | 413 | 2,99 | 2,34E-29 | 8,40E-27 | 3,6 | 5,76E-43 | 9,79E-41 | NO |
| 1434914 | 360 | 2,99 | 8,37E-20 | 5,75E-17 | 2,07 | 7,97E-10 | 1,12E-06 | NO |
| 1104621 | 3956 | 3 | 3,87E-37 | 7,85E-35 | 2,54 | 8,52E-27 | 3,37E-24 | NO |
| 1434837 | 38 | 3 | 1,23E-05 | 3,94E-02 | 0 | NA | NA | NO |
| 1448351 | 1372 | 3 | 1,12E-14 | 1,23E-11 | 3,9 | 3,52E-24 | 1,63E-21 | NO |
| 1370942 | 767 | 3,02 | 1,00E-24 | 4,91E-22 | 1,56 | 2,90E-07 | 5,60E-04 | NO |
| 1462962 | 14417 | 3,04 | 1,33E-28 | 4,91E-26 | 2,51 | 8,62E-20 | 5,41E-17 | NO |
| 699268 | 2932 | 3,05 | 8,74E-19 | 6,46E-16 | 2,52 | 4,44E-13 | 4,49E-10 | NO |
| 1476423 | 1424 | 3,05 | 5,13E-30 | 1,72E-27 | 4,18 | 7,97E-56 | 6,45E-54 | NO |
| 1439644 | 22 | 3,06 | 5,53E-07 | 1,45E-03 | 3,48 | 8,08E-09 | 1,27E-05 | NO |
| 1406122 | 113 | 3,07 | 4,12E-13 | 5,24E-10 | 0 | NA | NA | NO |
| 1431146 | 98 | 3,07 | 4,70E-12 | 6,77E-09 | 3,04 | 8,19E-12 | 9,47E-09 | NO |
| 1048895 | 947 | 3,08 | 3,78E-10 | 6,64E-07 | 4,63 | 1,13E-21 | 6,11E-19 | NO |
| 1373451 | 155 | 3,09 | 3,83E-11 | 6,08E-08 | 4,08 | 3,76E-19 | 2,47E-16 | NO |
| 1469456 | 55 | 3,09 | 9,27E-08 | 2,18E-04 | 0 | NA | NA | NO |
| 1470293 | 73 | 3,1 | 8,74E-15 | 9,46E-12 | 2,13 | 3,07E-07 | 5,96E-04 | NO |
| 237344 | 21 | 3,13 | 6,76E-07 | 1,80E-03 | 0 | NA | NA | NO |
| 1365110 | 540 | 3,13 | 8,09E-20 | 5,55E-17 | 4,19 | 4,77E-35 | 1,22E-32 | NO |
| 1393742 | 151 | 3,13 | 3,70E-12 | 5,27E-09 | 3,48 | 7,31E-15 | 6,45E-12 | NO |
| 1436729 | 87 | 3,13 | 9,44E-14 | 1,12E-10 | 0 | NA | NA | NO |
| 1436732 | 373 | 3,13 | 4,67E-20 | 3,13E-17 | 0 | NA | NA | NO |
| 1458699 | 301 | 3,13 | 1,86E-44 | 2,46E-42 | 3,19 | 2,48E-46 | 3,44E-44 | NO |
| 1473694 | 101 | 3,13 | 2,77E-13 | 3,46E-10 | 3,16 | 1,66E-13 | 1,63E-10 | NO |
| 685757 | 34 | 3,14 | 1,03E-05 | 3,24E-02 | 5,79 | 7,13E-18 | 5,09E-15 | NO |
| 1437225 | 554 | 3,14 | 2,12E-29 | 7,54E-27 | 2,65 | 3,60E-21 | 2,03E-18 | NO |
| 1466461 | 1175 | 3,14 | 6,13E-14 | 7,14E-11 | 3,78 | 9,86E-20 | 6,21E-17 | NO |
| 1447868 | 19 | 3,15 | 2,70E-06 | 7,84E-03 | 3,53 | 1,23E-07 | 2,24E-04 | NO |
| 358500 | 197 | 3,16 | 1,98E-23 | 1,04E-20 | 3,12 | 6,64E-23 | 3,34E-20 | NO |
| 1432456 | 186 | 3,16 | 1,29E-15 | 1,29E-12 | 1,91 | 3,63E-06 | 8,37E-03 | NO |
| 1470419 | 10677 | 3,16 | 4,22E-34 | 1,05E-31 | 4,37 | 1,59E-64 | 9,07E-63 | NO |
| 361980 | 1276 | 3,18 | 1,36E-17 | 1,14E-14 | 0 | NA | NA | NO |
| 1432880 | 1212 | 3,18 | 3,33E-37 | 6,69E-35 | 2,73 | 1,20E-27 | 4,47E-25 | NO |
| 1436952 | 4122 | 3,19 | 6,68E-21 | 4,22E-18 | 2,15 | 6,58E-10 | 9,14E-07 | NO |
| 1367513 | 398 | 3,21 | 1,37E-18 | 1,05E-15 | 3,38 | 1,76E-20 | 1,07E-17 | NO |
| 1436852 | 875 | 3,21 | 3,47E-22 | 1,99E-19 | 3,5 | 2,67E-26 | 1,09E-23 | NO |
| 1460751 | 3052 | 3,21 | 1,06E-26 | 4,44E-24 | 3,69 | 3,22E-35 | 8,14E-33 | NO |
| 607462 | 1009 | 3,22 | 9,09E-24 | 4,67E-21 | 2,31 | 1,29E-12 | 1,37E-09 | NO |
| 1441158 | 55 | 3,22 | 1,87E-11 | 2,88E-08 | 3,03 | 3,18E-10 | 4,29E-07 | NO |
| 60439 | 364 | 3,26 | 2,37E-11 | 3,67E-08 | 0 | NA | NA | NO |
| 1366581 | 450 | 3,26 | 4,72E-28 | 1,83E-25 | 2,88 | 5,54E-22 | 2,96E-19 | NO |
| 1438735 | 107603 | 3,28 | 4,45E-52 | 3,78E-50 | 3,64 | 6,09E-64 | 3,66E-62 | NO |
| 1459075 | 1070 | 3,29 | 1,77E-21 | 1,07E-18 | 1,96 | 3,43E-08 | 5,88E-05 | NO |
| 516113 | 827 | 3,3 | 1,10E-39 | 1,89E-37 | 2,36 | 1,42E-20 | 8,47E-18 | NO |
| 823548 | 114 | 3,3 | 1,91E-14 | 2,12E-11 | 2,67 | 1,01E-09 | 1,43E-06 | NO |
| 1479046 | 323 | 3,32 | 4,08E-33 | 1,09E-30 | 2,62 | 6,41E-21 | 3,73E-18 | NO |
| 824442 | 118 | 3,33 | 7,95E-12 | 1,18E-08 | 2,96 | 1,93E-09 | 2,84E-06 | NO |
| 908900 | 3169 | 3,34 | 2,08E-44 | 2,76E-42 | 3,23 | 1,41E-41 | 2,61E-39 | NO |
| 1261180 | 346 | 3,35 | 1,07E-20 | 6,81E-18 | 4,18 | 2,63E-32 | 7,70E-30 | NO |
| 1428382 | 158 | 3,35 | 4,43E-31 | 1,33E-28 | 3,6 | 2,37E-36 | 5,56E-34 | NO |
| 1456839 | 1166 | 3,36 | 1,82E-51 | 1,66E-49 | 4,14 | 1,64E-78 | 5,24E-77 | NO |
| 1370744 | 154 | 3,37 | 5,67E-12 | 8,25E-09 | 0 | NA | NA | NO |
| 2254 | 3228 | 3,39 | 1,96E-18 | 1,52E-15 | 3,53 | 6,16E-20 | 3,82E-17 | NO |
| 1414248 | 228 | 3,4 | 1,18E-12 | 1,59E-09 | 0 | NA | NA | NO |
| 1461735 | 50 | 3,41 | 1,19E-11 | 1,79E-08 | 3,58 | 8,10E-13 | 8,40E-10 | NO |
| 1467792 | 346 | 3,42 | 4,37E-26 | 1,94E-23 | 2,35 | 1,02E-12 | 1,07E-09 | NO |
| 1261580 | 320 | 3,43 | 9,86E-25 | 4,81E-22 | 3,56 | 1,22E-26 | 4,91E-24 | NO |
| 1437493 | 1131 | 3,43 | 9,45E-13 | 1,26E-09 | 2,32 | 2,56E-06 | 5,77E-03 | NO |
| 1463251 | 2369 | 3,44 | 1,04E-29 | 3,61E-27 | 4,24 | 8,30E-45 | 1,28E-42 | NO |
| 1372078 | 4073 | 3,45 | 2,72E-26 | 1,19E-23 | 5,06 | 1,20E-55 | 9,82E-54 | NO |
| 1435705 | 1451 | 3,45 | 1,05E-39 | 1,79E-37 | 3 | 3,81E-30 | 1,24E-27 | NO |
| 1060546 | 1941 | 3,46 | 1,58E-18 | 1,22E-15 | 3,38 | 1,11E-17 | 8,01E-15 | NO |
| 1441702 | 13112 | 3,46 | 7,90E-50 | 7,74E-48 | 3,23 | 1,35E-43 | 2,24E-41 | NO |
| 1456064 | 633 | 3,46 | 4,69E-21 | 2,94E-18 | 2,62 | 2,20E-12 | 2,39E-09 | NO |
| 1436879 | 292 | 3,49 | 9,77E-37 | 2,04E-34 | 3,2 | 3,96E-31 | 1,23E-28 | NO |
| 1473347 | 5604 | 3,53 | 1,69E-17 | 1,43E-14 | 2,21 | 2,12E-07 | 4,00E-04 | NO |
| 1480330 | 58 | 3,54 | 4,00E-13 | 5,08E-10 | 4,36 | 7,84E-20 | 4,90E-17 | NO |
| 1370986 | 1938 | 3,56 | 2,02E-17 | 1,72E-14 | 3,35 | 1,68E-15 | 1,43E-12 | NO |
| 598171 | 430 | 3,57 | 3,37E-27 | 1,37E-24 | 2,95 | 9,12E-19 | 6,17E-16 | NO |
| 918011 | 25 | 3,57 | 4,66E-08 | 1,05E-04 | 3,21 | 1,27E-06 | 2,74E-03 | NO |
| 1468577 | 296 | 3,57 | 2,90E-30 | 9,41E-28 | 3,09 | 7,70E-23 | 3,90E-20 | NO |
| 1471269 | 82 | 3,63 | 3,27E-14 | 3,71E-11 | 0 | NA | NA | NO |
| 1472566 | 1932 | 3,63 | 1,52E-41 | 2,30E-39 | 1,74 | 3,31E-10 | 4,47E-07 | NO |
| 710961 | 8 | 3,71 | 5,66E-06 | 1,72E-02 | 0 | NA | NA | NO |
| 1299577 | 26 | 3,72 | 1,23E-10 | 2,06E-07 | 3,21 | 5,05E-08 | 8,80E-05 | NO |
| 424576 | 2746 | 3,74 | 2,22E-33 | 5,74E-31 | 4,02 | 2,16E-38 | 4,48E-36 | NO |
| 959207 | 40 | 3,74 | 3,08E-13 | 3,88E-10 | 3,08 | 3,49E-09 | 5,28E-06 | NO |
| 47492 | 3147 | 3,75 | 9,32E-22 | 5,55E-19 | 3,06 | 9,75E-15 | 8,68E-12 | NO |
| 1224602 | 20 | 3,76 | 2,72E-09 | 5,30E-06 | 2,85 | 1,40E-05 | 3,55E-02 | NO |
| 1216997 | 15 | 3,8 | 2,44E-08 | 5,31E-05 | 3,58 | 1,98E-07 | 3,70E-04 | NO |
| 1374888 | 17425 | 3,82 | 8,82E-56 | 6,26E-54 | 3,84 | 3,33E-56 | 2,63E-54 | NO |
| 1474604 | 9122 | 3,82 | 9,03E-43 | 1,30E-40 | 2,13 | 6,33E-14 | 6,00E-11 | NO |
| 1096478 | 86 | 3,85 | 1,37E-21 | 8,21E-19 | 3,29 | 8,01E-16 | 6,66E-13 | NO |
| 762507 | 801 | 3,86 | 1,53E-15 | 1,53E-12 | 3,61 | 1,14E-13 | 1,10E-10 | NO |
| 1122554 | 295 | 3,86 | 9,78E-49 | 1,02E-46 | 2,87 | 4,14E-27 | 1,60E-24 | NO |
| 1465383 | 286 | 3,86 | 3,24E-36 | 6,87E-34 | 3,67 | 6,99E-33 | 1,97E-30 | NO |
| 1468122 | 3028 | 3,86 | 3,02E-39 | 5,25E-37 | 4,34 | 1,98E-49 | 2,28E-47 | NO |
| 1481386 | 357 | 3,88 | 8,11E-29 | 2,98E-26 | 2,17 | 1,44E-09 | 2,08E-06 | NO |
| 1368062 | 2163 | 3,91 | 6,61E-29 | 2,41E-26 | 4,43 | 5,75E-37 | 1,30E-34 | NO |
| 1435424 | 205 | 3,92 | 2,51E-27 | 1,02E-24 | 4,04 | 4,50E-29 | 1,57E-26 | NO |
| 4193 | 732 | 3,93 | 2,74E-25 | 1,29E-22 | 4,05 | 9,44E-27 | 3,74E-24 | NO |
| 1460750 | 366 | 3,93 | 5,82E-38 | 1,11E-35 | 4,45 | 4,51E-49 | 5,37E-47 | NO |
| 1466667 | 8543 | 3,94 | 3,25E-38 | 6,10E-36 | 4,48 | 2,39E-49 | 2,80E-47 | NO |
| 131196 | 11667 | 3,95 | 4,19E-63 | 2,05E-61 | 2,79 | 4,29E-32 | 1,27E-29 | NO |
| 1481976 | 280 | 3,95 | 4,87E-43 | 6,82E-41 | 2,76 | 4,90E-21 | 2,82E-18 | NO |
| 1459070 | 590 | 3,96 | 2,33E-45 | 2,96E-43 | 4 | 1,79E-46 | 2,47E-44 | NO |
| 1369886 | 838 | 3,97 | 1,81E-25 | 8,44E-23 | 3,92 | 8,48E-25 | 3,71E-22 | NO |
| 1465357 | 313 | 3,97 | 1,16E-26 | 4,91E-24 | 3,92 | 4,83E-26 | 2,00E-23 | NO |
| 1362569 | 244 | 3,98 | 2,28E-28 | 8,61E-26 | 2,82 | 1,54E-14 | 1,40E-11 | NO |
| 1458386 | 34 | 4,03 | 2,75E-12 | 3,88E-09 | 3,86 | 2,64E-11 | 3,20E-08 | NO |
| 1373202 | 1673 | 4,05 | 1,38E-33 | 3,50E-31 | 5,09 | 4,35E-53 | 4,13E-51 | NO |
| 1485202 | 1074 | 4,06 | 2,72E-11 | 4,25E-08 | 3,23 | 1,97E-07 | 3,69E-04 | NO |
| 194179 | 4986 | 4,08 | 2,86E-31 | 8,41E-29 | 4,68 | 5,63E-41 | 1,05E-38 | NO |
| 1376567 | 195 | 4,09 | 7,40E-17 | 6,57E-14 | 4,68 | 6,87E-22 | 3,68E-19 | NO |
| 423978 | 1305 | 4,11 | 1,55E-37 | 3,07E-35 | 4,45 | 5,68E-44 | 9,14E-42 | NO |
| 1425748 | 614 | 4,12 | 7,69E-40 | 1,28E-37 | 5,74 | 1,50E-77 | 4,95E-76 | NO |
| 1449122 | 692 | 4,15 | 6,37E-27 | 2,65E-24 | 1,98 | 8,00E-07 | 1,66E-03 | NO |
| 974066 | 4568 | 4,17 | 2,12E-26 | 9,08E-24 | 5,81 | 2,40E-50 | 2,67E-48 | NO |
| 1427917 | 374 | 4,17 | 5,82E-20 | 3,95E-17 | 4,41 | 3,24E-22 | 1,69E-19 | NO |
| 1367964 | 26852 | 4,18 | 8,31E-30 | 2,85E-27 | 3,58 | 5,04E-22 | 2,68E-19 | NO |
| 1026821 | 1317 | 4,19 | 1,86E-30 | 6,02E-28 | 3,92 | 9,73E-27 | 3,86E-24 | NO |
| 1431190 | 1508 | 4,2 | 3,56E-41 | 5,51E-39 | 5 | 2,40E-58 | 1,77E-56 | NO |
| 1430865 | 193 | 4,27 | 1,86E-18 | 1,44E-15 | 0 | NA | NA | NO |
| 1479460 | 2829 | 4,27 | 7,44E-34 | 1,86E-31 | 4,06 | 1,20E-30 | 3,86E-28 | NO |
| 1431373 | 463 | 4,29 | 3,54E-25 | 1,68E-22 | 4,29 | 3,94E-25 | 1,70E-22 | NO |
| 1431440 | 1934 | 4,3 | 1,34E-30 | 4,28E-28 | 3,1 | 2,51E-16 | 2,03E-13 | NO |
| 1439379 | 1158 | 4,3 | 3,51E-51 | 3,27E-49 | 4,35 | 2,75E-52 | 2,78E-50 | NO |
| 1469252 | 368 | 4,31 | 7,24E-38 | 1,40E-35 | 3,91 | 2,59E-31 | 8,01E-29 | NO |
| 675342 | 1647 | 4,35 | 4,75E-92 | 5,23E-91 | 4,23 | 2,11E-87 | 5,06E-86 | NO |
| 1358514 | 947 | 4,35 | 1,35E-28 | 4,99E-26 | 4,48 | 2,02E-30 | 6,53E-28 | NO |
| 1367344 | 833 | 4,35 | 6,57E-20 | 4,47E-17 | 0 | NA | NA | NO |
| 1467785 | 644 | 4,4 | 2,22E-46 | 2,68E-44 | 4,45 | 1,24E-47 | 1,58E-45 | NO |
| 1481812 | 218 | 4,42 | 1,01E-47 | 1,12E-45 | 4,13 | 7,22E-42 | 1,31E-39 | NO |
| 1437246 | 297 | 4,44 | 5,92E-50 | 5,74E-48 | 4,19 | 1,03E-44 | 1,60E-42 | NO |
| 1430513 | 7683 | 4,46 | 1,23E-27 | 4,94E-25 | 2,2 | 2,27E-07 | 4,32E-04 | NO |
| 1439530 | 226 | 4,46 | 3,82E-31 | 1,14E-28 | 4,5 | 9,10E-32 | 2,74E-29 | NO |
| 1122612 | 2875 | 4,47 | 1,46E-53 | 1,15E-51 | 5,14 | 9,20E-71 | 3,95E-69 | NO |
| 1470054 | 542 | 4,49 | 7,39E-32 | 2,09E-29 | 4,79 | 3,69E-36 | 8,72E-34 | NO |
| 1437494 | 312 | 4,52 | 3,18E-21 | 1,96E-18 | 3,44 | 1,48E-12 | 1,57E-09 | NO |
| 1472180 | 104 | 4,55 | 5,67E-31 | 1,72E-28 | 3,71 | 1,16E-20 | 6,85E-18 | NO |
| 1333125 | 115 | 4,56 | 2,96E-22 | 1,69E-19 | 4,61 | 9,01E-23 | 4,59E-20 | NO |
| 1465720 | 11836 | 4,6 | 5,28E-15 | 5,59E-12 | 0 | NA | NA | NO |
| 1299593 | 637 | 4,61 | 1,32E-47 | 1,49E-45 | 4,16 | 7,37E-39 | 1,52E-36 | NO |
| 1377613 | 1333 | 4,63 | 7,24E-65 | 3,19E-63 | 3,07 | 7,74E-29 | 2,75E-26 | NO |
| 1424822 | 1501 | 4,65 | 2,32E-67 | 9,50E-66 | 4,75 | 2,15E-70 | 9,47E-69 | NO |
| 1440175 | 1155 | 4,65 | 5,49E-73 | 1,87E-71 | 4,34 | 8,70E-64 | 5,31E-62 | NO |
| 1365342 | 44 | 4,72 | 1,12E-14 | 1,22E-11 | 4,04 | 6,64E-11 | 8,36E-08 | NO |
| 1378300 | 515 | 4,77 | 3,18E-39 | 5,60E-37 | 4,14 | 8,59E-30 | 2,89E-27 | NO |
| 1435985 | 570 | 4,89 | 9,84E-55 | 7,48E-53 | 5,27 | 5,09E-64 | 3,00E-62 | NO |
| 1446065 | 2934 | 4,92 | 5,58E-39 | 1,01E-36 | 7,6 | 1,79E-92 | 3,59E-91 | NO |
| 1434242 | 3811 | 4,95 | 1,32E-51 | 1,19E-49 | 5,07 | 2,84E-54 | 2,45E-52 | NO |
| 1437664 | 174 | 5,04 | 3,10E-31 | 9,16E-29 | 4,4 | 5,72E-24 | 2,66E-21 | NO |
| 1425384 | 2962 | 5,09 | 1,06E-59 | 5,81E-58 | 4,97 | 4,26E-57 | 3,32E-55 | NO |
| 1358770 | 999 | 5,12 | 6,74E-28 | 2,63E-25 | 3,11 | 7,71E-11 | 9,77E-08 | NO |
| 1461828 | 101 | 5,28 | 1,05E-27 | 4,16E-25 | 4,67 | 8,39E-22 | 4,52E-19 | NO |
| 1376304 | 836 | 5,33 | 3,67E-59 | 2,17E-57 | 4,92 | 1,53E-50 | 1,66E-48 | NO |
| 1437632 | 195 | 5,36 | 8,86E-25 | 4,32E-22 | 7,26 | 3,47E-45 | 5,21E-43 | NO |
| 679895 | 23453 | 5,37 | 8,68E-36 | 1,88E-33 | 0 | NA | NA | NO |
| 1389582 | 11797 | 5,37 | 2,95E-53 | 2,42E-51 | 6,32 | 1,34E-73 | 5,23E-72 | NO |
| 378107 | 3502 | 5,41 | 1,09E-51 | 9,56E-50 | 5,28 | 2,22E-49 | 2,58E-47 | NO |
| 1430324 | 605 | 5,41 | 8,02E-81 | 1,52E-79 | 4,77 | 3,82E-63 | 2,37E-61 | NO |
| 1476541 | 732 | 5,41 | 5,69E-39 | 1,04E-36 | 3,95 | 5,41E-21 | 3,13E-18 | NO |
| 1448251 | 931 | 5,46 | 1,18E-95 | 9,42E-95 | 4,73 | 5,54E-72 | 2,33E-70 | NO |
| 1434156 | 3310 | 5,49 | 1,37E-71 | 5,07E-70 | 6,35 | 1,30E-95 | 1,96E-94 | NO |
| 1029442 | 772 | 5,55 | 3,52E-45 | 4,50E-43 | 3,94 | 4,64E-23 | 2,31E-20 | NO |
| 1433407 | 3052 | 5,68 | 1,67E-86 | 2,67E-85 | 5,09 | 9,59E-70 | 4,41E-68 | NO |
| 1161828 | 68 | 5,74 | 6,13E-23 | 3,38E-20 | 5,79 | 2,26E-23 | 1,11E-20 | NO |
| 1460350 | 345 | 5,83 | 3,25E-33 | 8,51E-31 | 5,47 | 2,62E-29 | 9,04E-27 | NO |
| 1462030 | 397 | 5,86 | 6,61E-72 | 2,38E-70 | 5,71 | 1,98E-68 | 1,01E-66 | NO |
| 1437846 | 832 | 5,89 | 1,50E-53 | 1,20E-51 | 6,7 | 1,96E-69 | 9,23E-68 | NO |
| 1481388 | 81 | 5,99 | 1,03E-23 | 5,33E-21 | 5,56 | 1,81E-20 | 1,09E-17 | NO |
| 1466783 | 759 | 6,55 | 6,56E-42 | 9,77E-40 | 2,25 | 1,10E-05 | 2,75E-02 | NO |
| 1430411 | 2048 | 6,93 | 1,10E-95 | 7,67E-95 | 6,95 | 1,08E-96 | 1,51E-95 | NO |
| 1447350 | 2840 | 7,77 | 6,76E-112 | 1,35E-111 | 8,6 | 6,20E-138 | 2,48E-137 | NO |
| 448186 | 1672 | 8,84 | 1,22E-55 | 8,93E-54 | 10,07 | 4,83E-72 | 1,98E-70 | NO |
| 825568 | 154 | -5,61 | 4,55E-44 | 6,28E-42 | -6,58 | 2,55E-51 | 2,75E-49 | YES |
| 1430780 | 228 | -5,12 | 3,08E-40 | 4,95E-38 | -5,64 | 3,84E-47 | 5,07E-45 | YES |
| 1372485 | 2274 | -4,62 | 1,63E-56 | 1,09E-54 | -4,16 | 8,64E-47 | 1,18E-44 | YES |
| 1477168 | 8 | -4,45 | 1,13E-07 | 2,68E-04 | 0 | NA | NA | YES |
| 1378713 | 38262 | -4,41 | 5,62E-29 | 2,05E-26 | -4,37 | 1,93E-28 | 7,00E-26 | YES |
| 1463826 | 599 | -4,02 | 8,75E-24 | 4,48E-21 | -6,37 | 2,47E-53 | 2,32E-51 | YES |
| 1432235 | 16584 | -3,86 | 1,11E-30 | 3,51E-28 | -3,76 | 4,06E-29 | 1,41E-26 | YES |
| 1429447 | 860 | -3,69 | 4,14E-24 | 2,08E-21 | -4,23 | 3,53E-31 | 1,09E-28 | YES |
| 439739 | 575 | -3,69 | 6,78E-37 | 1,38E-34 | -3,63 | 3,81E-36 | 9,04E-34 | YES |
| 1438106 | 305 | -3,63 | 1,42E-40 | 2,26E-38 | -3,27 | 9,51E-35 | 2,50E-32 | YES |
| 1383560 | 730 | -3,56 | 2,87E-49 | 2,93E-47 | -3,9 | 7,63E-59 | 5,34E-57 | YES |
| 1473616 | 53 | -3,21 | 2,52E-09 | 4,88E-06 | -3,25 | 1,25E-09 | 1,79E-06 | YES |
| 1433312 | 2759 | -3,02 | 1,23E-18 | 9,29E-16 | -2,07 | 2,97E-09 | 4,46E-06 | YES |
| 1465293 | 35 | -2,85 | 5,32E-09 | 1,07E-05 | -3,16 | 1,24E-10 | 1,60E-07 | YES |
| 1422211 | 162 | -2,57 | 2,25E-25 | 1,06E-22 | -2,7 | 1,64E-28 | 5,91E-26 | YES |
| 82221 | 162 | -2,55 | 2,67E-27 | 1,08E-24 | -2,27 | 2,07E-23 | 1,01E-20 | YES |
| 1362523 | 188 | -2,54 | 7,59E-11 | 1,23E-07 | 0 | NA | NA | YES |
| 1458250 | 326 | -2,46 | 1,01E-11 | 1,51E-08 | 0 | NA | NA | YES |
| 1430232 | 21795 | -2,45 | 1,72E-09 | 3,25E-06 | -3,73 | 1,60E-20 | 9,61E-18 | YES |
| 551752 | 729 | -2,3 | 4,96E-20 | 3,34E-17 | -2,07 | 7,80E-17 | 6,04E-14 | YES |
| 1370680 | 8848 | -2,26 | 6,00E-12 | 8,75E-09 | -3,31 | 2,67E-24 | 1,23E-21 | YES |
| 1369435 | 159 | -2 | 6,48E-07 | 1,72E-03 | 0 | NA | NA | YES |
| 320808 | 6789 | -1,88 | 4,92E-07 | 1,28E-03 | -2,01 | 9,08E-08 | 1,63E-04 | YES |
| 1371481 | 28513 | -1,85 | 6,14E-06 | 1,87E-02 | -1,87 | 6,12E-06 | 1,46E-02 | YES |
| 414257 | 1545 | -1,85 | 1,21E-05 | 3,88E-02 | 0 | NA | NA | YES |
| 749314 | 114 | -1,8 | 4,50E-10 | 7,96E-07 | -1,42 | 6,10E-07 | 1,25E-03 | YES |
| 551360 | 407 | -1,79 | 1,38E-11 | 2,09E-08 | -1,63 | 8,45E-10 | 1,19E-06 | YES |
| 622110 | 69 | -1,77 | 1,61E-08 | 3,44E-05 | -1,34 | 9,68E-06 | 2,40E-02 | YES |
| 346141 | 10768 | -1,72 | 4,74E-09 | 9,46E-06 | -2,39 | 1,88E-16 | 1,50E-13 | YES |
| 1471120 | 72 | -1,69 | 1,44E-06 | 4,00E-03 | 0 | NA | NA | YES |
| 1439764 | 6942 | -1,67 | 2,36E-07 | 5,87E-04 | 0 | NA | NA | YES |
| 549630 | 112 | -1,56 | 5,25E-06 | 1,58E-02 | 0 | NA | NA | YES |
| 1083007 | 503 | -1,5 | 4,76E-07 | 1,24E-03 | 0 | NA | NA | YES |
| 1469190 | 520 | -1,49 | 7,31E-10 | 1,33E-06 | -1,24 | 3,47E-07 | 6,81E-04 | YES |
| 1146624 | 493 | -1,48 | 2,88E-08 | 6,33E-05 | -1,64 | 7,20E-10 | 1,00E-06 | YES |
| 1378508 | 3594 | -1,37 | 1,05E-09 | 1,95E-06 | -1,25 | 4,11E-08 | 7,09E-05 | YES |
| 1436723 | 231 | -1,37 | 6,16E-06 | 1,88E-02 | 0 | NA | NA | YES |
| 1439266 | 124573 | -1,35 | 1,52E-05 | 4,97E-02 | 0 | NA | NA | YES |
| 1433781 | 790 | -1,32 | 4,40E-08 | 9,91E-05 | 0 | NA | NA | YES |
| 1429301 | 1149 | -1,31 | 2,77E-09 | 5,39E-06 | 0 | NA | NA | YES |
| 1430159 | 459 | -1,28 | 2,03E-09 | 3,87E-06 | -2,41 | 1,14E-29 | 3,87E-27 | YES |
| 1471841 | 559 | -1,21 | 8,87E-07 | 2,41E-03 | 0 | NA | NA | YES |
| 1456299 | 658 | -1,2 | 4,11E-06 | 1,22E-02 | 0 | NA | NA | YES |
| 1463558 | 572 | -1,16 | 3,76E-06 | 1,12E-02 | 0 | NA | NA | YES |
| 1476087 | 3316 | -1,07 | 7,35E-06 | 2,27E-02 | 0 | NA | NA | YES |
| 1476659 | 22 | 0 | NA | NA | -3,37 | 2,50E-07 | 4,81E-04 | YES |
| 1460061 | 52 | 0 | NA | NA | -2,5 | 8,24E-08 | 1,47E-04 | YES |
| 1429790 | 2795 | 0 | NA | NA | -2,49 | 3,19E-08 | 5,43E-05 | YES |
| 1477746 | 73 | 0 | NA | NA | -2,01 | 6,25E-09 | 9,70E-06 | YES |
| 1435482 | 532 | 0 | NA | NA | -2 | 1,08E-09 | 1,53E-06 | YES |
| 1438616 | 228 | 0 | NA | NA | -1,9 | 5,97E-12 | 6,80E-09 | YES |
| 1450304 | 3398 | 0 | NA | NA | -1,88 | 1,08E-06 | 2,31E-03 | YES |
| 1370491 | 17314 | 0 | NA | NA | -1,67 | 2,12E-07 | 4,00E-04 | YES |
| 903408 | 2424 | 0 | NA | NA | -1,64 | 8,67E-06 | 2,13E-02 | YES |
| 1436784 | 121 | 0 | NA | NA | -1,5 | 1,48E-05 | 3,76E-02 | YES |
| 1438167 | 17045 | 0 | NA | NA | -1,47 | 3,72E-11 | 4,58E-08 | YES |
| 1466642 | 203 | 0 | NA | NA | -1,43 | 2,21E-06 | 4,93E-03 | YES |
| 1365786 | 1446 | 0 | NA | NA | -1,4 | 6,18E-06 | 1,48E-02 | YES |
| 1417357 | 1789 | 0 | NA | NA | -1,14 | 1,91E-05 | 4,95E-02 | YES |
| 100763 | 431 | 0 | NA | NA | 0 | NA | NA | YES |
| 141031 | 2305 | 0 | NA | NA | 0 | NA | NA | YES |
| 200988 | 89 | 0 | NA | NA | 0 | NA | NA | YES |
| 283597 | 46 | 0 | NA | NA | 0 | NA | NA | YES |
| 289748 | 106 | 0 | NA | NA | 0 | NA | NA | YES |
| 439297 | 131 | 0 | NA | NA | 0 | NA | NA | YES |
| 439566 | 60 | 0 | NA | NA | 0 | NA | NA | YES |
| 545636 | 593 | 0 | NA | NA | 0 | NA | NA | YES |
| 552246 | 823 | 0 | NA | NA | 0 | NA | NA | YES |
| 623803 | 174 | 0 | NA | NA | 0 | NA | NA | YES |
| 623888 | 64 | 0 | NA | NA | 0 | NA | NA | YES |
| 635387 | 12 | 0 | NA | NA | 0 | NA | NA | YES |
| 635629 | 65 | 0 | NA | NA | 0 | NA | NA | YES |
| 676339 | 53 | 0 | NA | NA | 0 | NA | NA | YES |
| 695093 | 25151 | 0 | NA | NA | 0 | NA | NA | YES |
| 903830 | 486 | 0 | NA | NA | 0 | NA | NA | YES |
| 922875 | 572 | 0 | NA | NA | 0 | NA | NA | YES |
| 924573 | 856 | 0 | NA | NA | 0 | NA | NA | YES |
| 946139 | 730 | 0 | NA | NA | 0 | NA | NA | YES |
| 1071275 | 210 | 0 | NA | NA | 0 | NA | NA | YES |
| 1105342 | 111 | 0 | NA | NA | 0 | NA | NA | YES |
| 1222790 | 170 | 0 | NA | NA | 0 | NA | NA | YES |
| 1360228 | 133 | 0 | NA | NA | 0 | NA | NA | YES |
| 1360626 | 1775 | 0 | NA | NA | 0 | NA | NA | YES |
| 1361382 | 1609 | 0 | NA | NA | 0 | NA | NA | YES |
| 1363939 | 1036 | 0 | NA | NA | 0 | NA | NA | YES |
| 1365397 | 4028 | 0 | NA | NA | 0 | NA | NA | YES |
| 1367557 | 2366 | 0 | NA | NA | 0 | NA | NA | YES |
| 1371540 | 88 | 0 | NA | NA | 0 | NA | NA | YES |
| 1372816 | 866 | 0 | NA | NA | 0 | NA | NA | YES |
| 1374005 | 34168 | 0 | NA | NA | 0 | NA | NA | YES |
| 1378433 | 938 | 0 | NA | NA | 0 | NA | NA | YES |
| 1379075 | 345 | 0 | NA | NA | 0 | NA | NA | YES |
| 1408307 | 665 | 0 | NA | NA | 0 | NA | NA | YES |
| 1408988 | 2986 | 0 | NA | NA | 0 | NA | NA | YES |
| 1414805 | 21 | 0 | NA | NA | 0 | NA | NA | YES |
| 1415355 | 203 | 0 | NA | NA | 0 | NA | NA | YES |
| 1422261 | 28 | 0 | NA | NA | 0 | NA | NA | YES |
| 1424636 | 16202 | 0 | NA | NA | 0 | NA | NA | YES |
| 1425407 | 279 | 0 | NA | NA | 0 | NA | NA | YES |
| 1427221 | 45 | 0 | NA | NA | 0 | NA | NA | YES |
| 1427569 | 150 | 0 | NA | NA | 0 | NA | NA | YES |
| 1427639 | 283 | 0 | NA | NA | 0 | NA | NA | YES |
| 1429324 | 98399 | 0 | NA | NA | 0 | NA | NA | YES |
| 1431031 | 5373 | 0 | NA | NA | 0 | NA | NA | YES |
| 1431278 | 1251 | 0 | NA | NA | 0 | NA | NA | YES |
| 1432154 | 29138 | 0 | NA | NA | 0 | NA | NA | YES |
| 1432578 | 50 | 0 | NA | NA | 0 | NA | NA | YES |
| 1433270 | 178 | 0 | NA | NA | 0 | NA | NA | YES |
| 1433612 | 2332 | 0 | NA | NA | 0 | NA | NA | YES |
| 1433856 | 16588 | 0 | NA | NA | 0 | NA | NA | YES |
| 1435663 | 5289 | 0 | NA | NA | 0 | NA | NA | YES |
| 1435742 | 19981 | 0 | NA | NA | 0 | NA | NA | YES |
| 1435819 | 63 | 0 | NA | NA | 0 | NA | NA | YES |
| 1435863 | 118 | 0 | NA | NA | 0 | NA | NA | YES |
| 1436014 | 71229 | 0 | NA | NA | 0 | NA | NA | YES |
| 1436016 | 471 | 0 | NA | NA | 0 | NA | NA | YES |
| 1436839 | 737 | 0 | NA | NA | 0 | NA | NA | YES |
| 1437024 | 9763 | 0 | NA | NA | 0 | NA | NA | YES |
| 1438311 | 208 | 0 | NA | NA | 0 | NA | NA | YES |
| 1438450 | 113 | 0 | NA | NA | 0 | NA | NA | YES |
| 1438835 | 6449 | 0 | NA | NA | 0 | NA | NA | YES |
| 1439269 | 29 | 0 | NA | NA | 0 | NA | NA | YES |
| 1440027 | 5331 | 0 | NA | NA | 0 | NA | NA | YES |
| 1443198 | 86 | 0 | NA | NA | 0 | NA | NA | YES |
| 1443697 | 824 | 0 | NA | NA | 0 | NA | NA | YES |
| 1453681 | 2418 | 0 | NA | NA | 0 | NA | NA | YES |
| 1455990 | 147570 | 0 | NA | NA | 0 | NA | NA | YES |
| 1456177 | 49 | 0 | NA | NA | 0 | NA | NA | YES |
| 1460063 | 53 | 0 | NA | NA | 0 | NA | NA | YES |
| 1462150 | 112924 | 0 | NA | NA | 0 | NA | NA | YES |
| 1465319 | 799 | 0 | NA | NA | 0 | NA | NA | YES |
| 1466016 | 971 | 0 | NA | NA | 0 | NA | NA | YES |
| 1467686 | 267 | 0 | NA | NA | 0 | NA | NA | YES |
| 1467721 | 1470 | 0 | NA | NA | 0 | NA | NA | YES |
| 1469390 | 838 | 0 | NA | NA | 0 | NA | NA | YES |
| 1470097 | 13335 | 0 | NA | NA | 0 | NA | NA | YES |
| 1471557 | 187 | 0 | NA | NA | 0 | NA | NA | YES |
| 1471647 | 8953 | 0 | NA | NA | 0 | NA | NA | YES |
| 1472453 | 5154 | 0 | NA | NA | 0 | NA | NA | YES |
| 1473281 | 365 | 0 | NA | NA | 0 | NA | NA | YES |
| 1474622 | 772 | 0 | NA | NA | 0 | NA | NA | YES |
| 1475073 | 147556 | 0 | NA | NA | 0 | NA | NA | YES |
| 1476709 | 87 | 0 | NA | NA | 0 | NA | NA | YES |
| 1476845 | 1192 | 0 | NA | NA | 0 | NA | NA | YES |
| 1476926 | 1421 | 0 | NA | NA | 0 | NA | NA | YES |
| 1479693 | 524 | 0 | NA | NA | 0 | NA | NA | YES |
| 1481102 | 1099 | 0 | NA | NA | 0 | NA | NA | YES |
| 1481758 | 49 | 0 | NA | NA | 0 | NA | NA | YES |
| 851098 | 515 | 0 | NA | NA | 1,1 | 1,59E-05 | 4,08E-02 | YES |
| 1357265 | 485 | 0 | NA | NA | 1,38 | 5,12E-07 | 1,03E-03 | YES |
| 1396733 | 7502 | 0 | NA | NA | 1,41 | 1,46E-08 | 2,37E-05 | YES |
| 1446896 | 1704 | 0 | NA | NA | 1,42 | 2,25E-09 | 3,33E-06 | YES |
| 1452204 | 1299 | 0 | NA | NA | 1,45 | 1,17E-06 | 2,50E-03 | YES |
| 1438510 | 9931 | 0 | NA | NA | 1,46 | 5,79E-07 | 1,18E-03 | YES |
| 1358178 | 173 | 0 | NA | NA | 1,51 | 1,17E-06 | 2,50E-03 | YES |
| 851061 | 1089 | 0 | NA | NA | 1,52 | 1,22E-19 | 7,75E-17 | YES |
| 1440416 | 199 | 0 | NA | NA | 1,81 | 9,06E-11 | 1,16E-07 | YES |
| 1433414 | 2102 | 0 | NA | NA | 1,9 | 7,57E-09 | 1,18E-05 | YES |
| 1432889 | 347 | 0 | NA | NA | 2,03 | 2,37E-10 | 3,16E-07 | YES |
| 1432779 | 148 | 0 | NA | NA | 2,13 | 8,34E-11 | 1,06E-07 | YES |
| 1395309 | 26 | 0 | NA | NA | 2,16 | 2,49E-06 | 5,60E-03 | YES |
| 1211525 | 48 | 0 | NA | NA | 2,33 | 6,27E-06 | 1,50E-02 | YES |
| 1364698 | 1918 | 0 | NA | NA | 2,34 | 8,21E-09 | 1,29E-05 | YES |
| 1301709 | 34 | 0 | NA | NA | 2,35 | 1,55E-05 | 3,96E-02 | YES |
| 1439851 | 436 | 0 | NA | NA | 2,42 | 5,39E-08 | 9,43E-05 | YES |
| 1464422 | 1816 | 0 | NA | NA | 3,05 | 8,45E-23 | 4,29E-20 | YES |
| 1496601 | 15 | 0 | NA | NA | 3,27 | 5,84E-06 | 1,39E-02 | YES |
| 1468081 | 2495 | 0 | NA | NA | 3,39 | 2,06E-08 | 3,41E-05 | YES |
| 1431530 | 8217 | 0 | NA | NA | 3,84 | 6,90E-17 | 5,30E-14 | YES |
| 1440356 | 326 | 0 | NA | NA | 3,84 | 1,68E-24 | 7,53E-22 | YES |
| 1435353 | 495 | 0 | NA | NA | 4,35 | 8,26E-43 | 1,41E-40 | YES |
| 1419800 | 6394 | 0 | NA | NA | 7,01 | 6,10E-34 | 1,65E-31 | YES |
| 245006 | 1559 | 0,88 | 1,17E-05 | 3,73E-02 | 0 | NA | NA | YES |
| 1432507 | 4744 | 0,94 | 3,36E-06 | 9,90E-03 | 0 | NA | NA | YES |
| 1445940 | 1171 | 0,95 | 1,83E-06 | 5,17E-03 | 0 | NA | NA | YES |
| 1463624 | 4756 | 1,06 | 1,34E-09 | 2,50E-06 | 1,19 | 9,79E-12 | 1,14E-08 | YES |
| 1438381 | 9718 | 1,1 | 1,42E-06 | 3,94E-03 | 0 | NA | NA | YES |
| 1431975 | 264 | 1,13 | 7,99E-06 | 2,48E-02 | 0 | NA | NA | YES |
| 1471729 | 227 | 1,25 | 1,93E-06 | 5,48E-03 | 1,49 | 1,11E-08 | 1,78E-05 | YES |
| 1374607 | 2517 | 1,36 | 4,15E-07 | 1,07E-03 | 1,37 | 4,39E-07 | 8,77E-04 | YES |
| 1466102 | 1063 | 1,42 | 1,94E-07 | 4,73E-04 | 1,35 | 9,05E-07 | 1,91E-03 | YES |
| 1357883 | 1424 | 1,46 | 2,37E-06 | 6,82E-03 | 0 | NA | NA | YES |
| 1430221 | 21049 | 1,47 | 1,15E-07 | 2,72E-04 | 0 | NA | NA | YES |
| 1433150 | 17773 | 1,49 | 1,70E-08 | 3,63E-05 | 0 | NA | NA | YES |
| 1433585 | 2135 | 1,52 | 1,80E-07 | 4,36E-04 | 0 | NA | NA | YES |
| 1448357 | 1654 | 1,59 | 5,22E-07 | 1,37E-03 | 0 | NA | NA | YES |
| 1459831 | 2808 | 1,59 | 6,97E-10 | 1,26E-06 | 1,51 | 6,14E-09 | 9,52E-06 | YES |
| 360196 | 861 | 1,68 | 1,04E-05 | 3,27E-02 | 2,99 | 1,24E-15 | 1,05E-12 | YES |
| 1357153 | 1427 | 1,69 | 3,05E-06 | 8,89E-03 | 0 | NA | NA | YES |
| 1430362 | 29798 | 1,69 | 1,10E-10 | 1,84E-07 | 0 | NA | NA | YES |
| 362390 | 1307 | 1,7 | 1,65E-11 | 2,52E-08 | 0 | NA | NA | YES |
| 1212120 | 4036 | 1,71 | 7,22E-10 | 1,31E-06 | 0 | NA | NA | YES |
| 1371908 | 3347 | 1,71 | 5,93E-11 | 9,54E-08 | 2,01 | 1,18E-14 | 1,06E-11 | YES |
| 1472054 | 794 | 1,73 | 3,01E-11 | 4,72E-08 | 1,18 | 1,04E-05 | 2,59E-02 | YES |
| 1370196 | 303 | 1,75 | 1,41E-07 | 3,38E-04 | 0 | NA | NA | YES |
| 1371041 | 668 | 1,75 | 6,37E-07 | 1,69E-03 | 2,34 | 1,53E-11 | 1,81E-08 | YES |
| 1123967 | 1218 | 1,78 | 1,37E-13 | 1,67E-10 | 0 | NA | NA | YES |
| 1410891 | 966 | 1,83 | 1,16E-08 | 2,45E-05 | 0 | NA | NA | YES |
| 1438017 | 53 | 1,87 | 6,42E-08 | 1,47E-04 | 0 | NA | NA | YES |
| 1392983 | 909 | 1,89 | 3,15E-11 | 4,96E-08 | 1,91 | 2,40E-11 | 2,91E-08 | YES |
| 1434140 | 1015 | 1,91 | 6,65E-12 | 9,77E-09 | 0 | NA | NA | YES |
| 1464936 | 1190 | 1,96 | 3,76E-13 | 4,77E-10 | 0 | NA | NA | YES |
| 1513135 | 189 | 1,96 | 1,94E-08 | 4,16E-05 | 0 | NA | NA | YES |
| 379384 | 9557 | 1,97 | 5,00E-07 | 1,31E-03 | 0 | NA | NA | YES |
| 1361583 | 975 | 1,99 | 4,47E-09 | 8,87E-06 | 0 | NA | NA | YES |
| 1424623 | 466 | 2 | 1,11E-08 | 2,32E-05 | 0 | NA | NA | YES |
| 1481209 | 389 | 2,01 | 4,11E-15 | 4,30E-12 | 2,3 | 1,06E-19 | 6,71E-17 | YES |
| 1358666 | 7419 | 2,09 | 3,48E-12 | 4,93E-09 | 0 | NA | NA | YES |
| 1437943 | 58 | 2,11 | 1,85E-06 | 5,24E-03 | 0 | NA | NA | YES |
| 1437014 | 387 | 2,11 | 1,95E-10 | 3,34E-07 | 2,73 | 8,34E-17 | 6,48E-14 | YES |
| 1401603 | 2846 | 2,12 | 3,22E-12 | 4,55E-09 | 2,04 | 2,37E-11 | 2,87E-08 | YES |
| 837609 | 228 | 2,15 | 8,70E-08 | 2,04E-04 | 2,8 | 1,14E-12 | 1,20E-09 | YES |
| 1393004 | 1451 | 2,16 | 7,75E-12 | 1,15E-08 | 0 | NA | NA | YES |
| 142587 | 989 | 2,18 | 6,91E-24 | 3,51E-21 | 1,59 | 4,28E-13 | 4,32E-10 | YES |
| 1468852 | 53 | 2,19 | 6,40E-10 | 1,15E-06 | 2,03 | 1,36E-08 | 2,20E-05 | YES |
| 1419907 | 112853 | 2,2 | 9,12E-13 | 1,21E-09 | 2,54 | 1,75E-16 | 1,40E-13 | YES |
| 1463286 | 1236 | 2,2 | 2,04E-07 | 4,99E-04 | 4,42 | 8,06E-27 | 3,18E-24 | YES |
| 957841 | 688 | 2,23 | 3,72E-06 | 1,10E-02 | 0 | NA | NA | YES |
| 1436512 | 12228 | 2,23 | 1,08E-12 | 1,44E-09 | 1,55 | 1,45E-06 | 3,17E-03 | YES |
| 1426787 | 9081 | 2,24 | 1,02E-13 | 1,21E-10 | 0 | NA | NA | YES |
| 1439817 | 11978 | 2,28 | 4,51E-14 | 5,20E-11 | 0 | NA | NA | YES |
| 1462884 | 1486 | 2,3 | 4,29E-14 | 4,94E-11 | 1,56 | 5,54E-07 | 1,13E-03 | YES |
| 1437868 | 1250 | 2,32 | 2,76E-18 | 2,17E-15 | 2,31 | 4,44E-18 | 3,12E-15 | YES |
| 1427877 | 42 | 2,37 | 2,02E-06 | 5,75E-03 | 0 | NA | NA | YES |
| 1476545 | 10249 | 2,37 | 4,98E-22 | 2,89E-19 | 1,7 | 1,02E-11 | 1,20E-08 | YES |
| 963141 | 213 | 2,4 | 8,42E-08 | 1,96E-04 | 0 | NA | NA | YES |
| 1385227 | 366 | 2,42 | 4,39E-25 | 2,09E-22 | 1,67 | 2,72E-12 | 2,99E-09 | YES |
| 1433130 | 1082 | 2,45 | 2,36E-12 | 3,29E-09 | 4,75 | 1,21E-44 | 1,89E-42 | YES |
| 1469854 | 3787 | 2,47 | 3,14E-16 | 2,94E-13 | 1,52 | 9,23E-07 | 1,95E-03 | YES |
| 1430225 | 453 | 2,47 | 1,12E-10 | 1,87E-07 | 1,89 | 1,42E-06 | 3,08E-03 | YES |
| 1496796 | 106 | 2,53 | 8,78E-09 | 1,82E-05 | 0 | NA | NA | YES |
| 1426324 | 14067 | 2,53 | 1,33E-24 | 6,59E-22 | 1,76 | 2,24E-12 | 2,44E-09 | YES |
| 1431105 | 97 | 2,54 | 1,16E-07 | 2,76E-04 | 0 | NA | NA | YES |
| 1439990 | 4016 | 2,55 | 3,97E-47 | 4,64E-45 | 1,87 | 9,51E-26 | 3,97E-23 | YES |
| 1471918 | 2599 | 2,57 | 1,61E-15 | 1,62E-12 | 2,3 | 1,54E-12 | 1,65E-09 | YES |
| 1394179 | 435 | 2,6 | 1,33E-19 | 9,26E-17 | 1,51 | 4,10E-07 | 8,15E-04 | YES |
| 1437714 | 1213 | 2,61 | 2,15E-13 | 2,67E-10 | 0 | NA | NA | YES |
| 1438096 | 1584 | 2,61 | 5,33E-09 | 1,07E-05 | 0 | NA | NA | YES |
| 1373458 | 453 | 2,61 | 8,24E-19 | 6,07E-16 | 2,54 | 8,61E-18 | 6,16E-15 | YES |
| 1438101 | 4493 | 2,63 | 2,78E-15 | 2,85E-12 | 0 | NA | NA | YES |
| 1372210 | 1170 | 2,64 | 8,19E-14 | 9,67E-11 | 2,46 | 5,10E-12 | 5,78E-09 | YES |
| 1429393 | 6540 | 2,65 | 3,88E-19 | 2,81E-16 | 2,17 | 4,21E-13 | 4,24E-10 | YES |
| 1438012 | 2296 | 2,68 | 1,94E-17 | 1,64E-14 | 2,41 | 2,63E-14 | 2,42E-11 | YES |
| 532900 | 924 | 2,69 | 8,73E-22 | 5,17E-19 | 0 | NA | NA | YES |
| 1441479 | 2747 | 2,75 | 9,08E-14 | 1,08E-10 | 3,93 | 4,03E-27 | 1,55E-24 | YES |
| 459887 | 1354 | 2,77 | 2,11E-13 | 2,61E-10 | 2,85 | 4,42E-14 | 4,14E-11 | YES |
| 1367894 | 655 | 2,82 | 3,90E-20 | 2,59E-17 | 3,71 | 2,31E-34 | 6,13E-32 | YES |
| 1365031 | 17097 | 2,87 | 2,39E-23 | 1,27E-20 | 2,74 | 2,09E-21 | 1,16E-18 | YES |
| 1381070 | 363 | 2,89 | 2,33E-14 | 2,63E-11 | 0 | NA | NA | YES |
| 1474491 | 270 | 2,91 | 8,21E-15 | 8,82E-12 | 0 | NA | NA | YES |
| 1512352 | 11231 | 2,97 | 1,32E-34 | 3,16E-32 | 2,18 | 4,53E-19 | 2,99E-16 | YES |
| 1438060 | 8730 | 2,99 | 4,12E-16 | 3,92E-13 | 4,34 | 7,45E-33 | 2,11E-30 | YES |
| 1464576 | 2135 | 3,01 | 3,24E-14 | 3,67E-11 | 0 | NA | NA | YES |
| 1430664 | 34355 | 3,01 | 5,39E-24 | 2,72E-21 | 2,01 | 3,01E-11 | 3,68E-08 | YES |
| 1466289 | 1570 | 3,02 | 1,23E-14 | 1,35E-11 | 2,8 | 1,21E-12 | 1,28E-09 | YES |
| 1418815 | 199 | 3,08 | 1,86E-15 | 1,89E-12 | 0 | NA | NA | YES |
| 1473624 | 15332 | 3,1 | 1,40E-10 | 2,35E-07 | 0 | NA | NA | YES |
| 1415976 | 254 | 3,12 | 7,75E-11 | 1,26E-07 | 0 | NA | NA | YES |
| 1468953 | 1020 | 3,15 | 1,40E-34 | 3,37E-32 | 2,75 | 1,07E-26 | 4,27E-24 | YES |
| 1481424 | 1508 | 3,17 | 2,04E-17 | 1,74E-14 | 2,62 | 3,38E-12 | 3,75E-09 | YES |
| 1458725 | 110 | 3,17 | 1,57E-19 | 1,10E-16 | 2,93 | 1,01E-16 | 7,89E-14 | YES |
| 349389 | 163 | 3,23 | 4,36E-09 | 8,64E-06 | 0 | NA | NA | YES |
| 380332 | 66260 | 3,24 | 3,17E-23 | 1,70E-20 | 3,48 | 1,18E-26 | 4,73E-24 | YES |
| 1361878 | 1856 | 3,3 | 2,23E-82 | 4,02E-81 | 3,34 | 7,50E-85 | 1,88E-83 | YES |
| 1440549 | 1451 | 3,34 | 1,81E-30 | 5,82E-28 | 3,74 | 3,94E-38 | 8,28E-36 | YES |
| 1369226 | 1749 | 3,5 | 2,25E-08 | 4,88E-05 | 0 | NA | NA | YES |
| 1435229 | 2466 | 3,51 | 2,13E-18 | 1,66E-15 | 2,71 | 2,89E-11 | 3,53E-08 | YES |
| 1430637 | 1662 | 3,55 | 2,87E-18 | 2,27E-15 | 1,99 | 2,37E-06 | 5,30E-03 | YES |
| 1446467 | 700 | 3,58 | 4,56E-24 | 2,29E-21 | 2,91 | 3,54E-16 | 2,88E-13 | YES |
| 1463290 | 2386 | 3,61 | 3,48E-28 | 1,34E-25 | 3,29 | 1,75E-23 | 8,45E-21 | YES |
| 1464761 | 35266 | 3,72 | 4,50E-33 | 1,21E-30 | 2,02 | 2,37E-10 | 3,15E-07 | YES |
| 1439628 | 1285 | 3,9 | 1,00E-32 | 2,75E-30 | 2,38 | 1,00E-12 | 1,05E-09 | YES |
| 1433043 | 507 | 3,93 | 3,19E-47 | 3,70E-45 | 3,51 | 6,89E-38 | 1,47E-35 | YES |
| 430950 | 797 | 3,98 | 1,93E-35 | 4,33E-33 | 2,91 | 2,20E-19 | 1,43E-16 | YES |
| 1437467 | 114850 | 4,07 | 5,80E-36 | 1,24E-33 | 3,63 | 8,55E-29 | 3,05E-26 | YES |
| 1469536 | 1106 | 4,33 | 3,56E-30 | 1,17E-27 | 4,61 | 3,39E-34 | 9,05E-32 | YES |
| 1445412 | 1193 | 4,36 | 3,63E-33 | 9,55E-31 | 2,47 | 2,83E-11 | 3,44E-08 | YES |
| 1474390 | 4507 | 4,54 | 1,19E-11 | 1,80E-08 | 5,67 | 1,84E-17 | 1,34E-14 | YES |
| 1445998 | 4116 | 4,55 | 6,91E-110 | 2,07E-109 | 3,23 | 4,04E-56 | 3,23E-54 | YES |
| 1439153 | 2574 | 4,55 | 1,11E-37 | 2,17E-35 | 5,06 | 1,39E-46 | 1,90E-44 | YES |
| 1445359 | 186 | 4,58 | 3,43E-12 | 4,87E-09 | 2,99 | 1,03E-05 | 2,57E-02 | YES |
| 1420128 | 33 | 4,65 | 6,33E-12 | 9,28E-09 | 3,93 | 1,25E-08 | 2,01E-05 | YES |
| 1462033 | 1242 | 5,05 | 5,03E-87 | 7,05E-86 | 5,21 | 4,23E-93 | 8,05E-92 | YES |
| 1467913 | 13325 | 5,24 | 3,65E-33 | 9,65E-31 | 6,56 | 1,49E-51 | 1,58E-49 | YES |
| 1358820 | 7550 | 5,25 | 4,53E-75 | 1,27E-73 | 3,85 | 5,74E-41 | 1,08E-38 | YES |
| 1363452 | 4138 | 5,35 | 7,86E-37 | 1,62E-34 | 4 | 5,89E-21 | 3,41E-18 | YES |
| 1477226 | 135422 | 5,51 | 2,42E-59 | 1,40E-57 | 4,88 | 7,00E-47 | 9,38E-45 | YES |
| 1413494 | 507 | 5,66 | 2,96E-29 | 1,07E-26 | 4,94 | 1,71E-22 | 8,82E-20 | YES |
| 1443603 | 1283 | 5,85 | 1,39E-60 | 7,38E-59 | 5,71 | 9,94E-58 | 7,46E-56 | YES |
| 1480292 | 1628 | 5,89 | 8,03E-22 | 4,72E-19 | 4,5 | 4,67E-13 | 4,73E-10 | YES |
| 1425076 | 20518 | 5,92 | 2,21E-93 | 1,99E-92 | 8,05 | 3,15E-173 | 6,29E-173 | YES |
| 270010 | 138040 | 5,97 | 5,15E-20 | 3,48E-17 | 5,02 | 2,19E-14 | 2,01E-11 | YES |
| 701883 | 2612 | 6,18 | 3,25E-40 | 5,30E-38 | 6,91 | 3,08E-50 | 3,45E-48 | YES |
| 1364528 | 4193 | 6,19 | 3,00E-48 | 3,21E-46 | 6,57 | 2,94E-54 | 2,56E-52 | YES |
| 271638 | 1928 | 8,33 | 1,42E-69 | 5,38E-68 | 8,35 | 5,12E-70 | 2,30E-68 | YES |
| 1477222 | 3938 | 9,04 | 1,45E-42 | 2,10E-40 | 8 | 1,25E-33 | 3,42E-31 | YES |
| 1468135 | 5586 | 9,2 | 4,02E-78 | 1,00E-76 | 10,54 | 1,84E-102 | 2,02E-101 | YES |
| 1437297 | 8732 | 9,27 | 4,76E-103 | 2,85E-102 | 9,51 | 1,27E-108 | 8,91E-108 | YES |

The table shows the mean number of reads for each gene in the maltose control condition and the fold change of transcript levels expressed as log2 on each substrate as compared to maltose.

NA: not applicable

padj: p value adjusted with the Benjamini-Hochberg method

padj_Bonf: p value adjusted with the Bonferroni method

**Supplementary Table S6**: Proteins of unknown function identified in proteome analyses

|  | Number of identified peptides | | | Blast NCBI |
| --- | --- | --- | --- | --- |
| Protein ID | PcoMaltose | PcoPine | PcoAspen | Putative conserved domains |
| 695093 | Nd | 3 | 2 | No putative conserved domain |
| 1369226 | Nd | Nd | 2 | DUF3455 |
| 1372210 | Nd | 2 | 4 | No putative conserved domain |
| 1408249 | Nd | 2 | 2 | Polysaccharide lyase 14-like |
| 1415976 | Nd | 5 | 6 | Ferritin-like |
| 1419907 | Nd | 3 | 3 | GPI-anchored |
| 1429324 | Nd | 5 | 2 | No putative conserved domain |
| 1430664 | Nd | 8 | 5 | No putative conserved domain |
| 1435353 | Nd | Nd | 2 | Putative endoglucanase-like |
| 1436014 | Nd | 11 | 6 | Cerato-platanin-like |
| 1438060 | Nd | 3 | Nd | Peptidase-like |
| 1441479 | Nd | 7 | 7 | PNGaseA-like |
| 1445926 | Nd | 9 | 8 | Ferritin-like |
| 1464761 | Nd | 3 | Nd | Cupredoxin-like |
| 1466289 | Nd | 2 | 3 | GH88-like |
| 1474634 | Nd | 2 | 2 | Feruloyl esterase-like |
| 1370491 | 2 | 4 | 4 | Chitin deactetylase-like |
| 1378713 | 2 | Nd | Nd | No putative conserved domain |
| 1436839 | 2 | Nd | Nd | Phosphoesterase-like |
| 1356397 | 3 | 11 | 10 | GPI-anchored |
| 1365786 | 3 | 4 | 2 | FAD-binding |
| 1439266 | 3 | 14 | 9 | No putative conserved domain |
| 320808 | 3 | Nd | Nd | No putative conserved domain |
| 378107 | 3 | 2 | 4 | Lactonase-like |
| 1435742 | 3 | Nd | 2 | Cerato-platanin-like |
| 1430281 | 4 | 2 | 3 | Sugar lactone lactonase-like |
| 1438510 | 4 | Nd | Nd | Endonuclease/exonuclease/phosphatase-like |
| 1477152 | 5 | 7 | 6 | No putative conserved domain |
| 1370680 | 6 | Nd | Nd | Cupin-like |
| 1371414 | 6 | 7 | 5 | No putative conserved domain |
| 1372485 | 8 | 2 | Nd | Cupin-like |
| 1409615 | 8 | 17 | 14 | No putative conserved domain |
| 1437297 | 8 | 18 | 23 | Thaumatin-like |
| 1424636 | 10 | 6 | 5 | Peptidase M35-like |
| 1480292 | 10 | 23 | 19 | No putative conserved domain |
| 1437289 | 12 | 6 | 6 | Esterase/lipase-like |
| 1369309 | 13 | Nd | Nd | No putative conserved domain |
| 1432154 | 13 | Nd | Nd | No putative conserved domain |
| 1471512 | 15 | 3 | Nd | No putative conserved domain |
| 1430637 | 19 | 22 | 17 | Nuclease-like |
| 1471500 | 19 | 3 | Nd | No putative conserved domain |
| 1372762 | 22 | Nd | 7 | Peptidase G1-like |
| 1364528 | 31 | 65 | 84 | DUF1793 |
| 1371481 | 31 | 9 | 13 | Thaumatin-like |
| 1435663 | 32 | 17 | 12 | Nuclease-like |
| 1437412 | 35 | 47 | 25 | Phosphatase-like |
| 1435229 | 39 | 12 | 3 | Peptidase-like |
| 1469417 | 39 | 27 | 20 | Phosphatase-like |

Nd : not detected

**Supplementary Table S7**: Carbohydrate composition of pine and aspen substrates used in the study

|  | % dry weight | |
| --- | --- | --- |
|  | Pine | Aspen |
| Rhamnose | 0.14 +/- 0.08 | 0.21 +/-0.12 |
| Arabinose | 2.09 +/- 0.09 | 0.40 +/-0.03 |
| Xylose | 7 .62 +/-0.19 | 16.85 +/-0.16 |
| Mannose | 9.04 +/-0.37 | 1.77 +/-0.05 |
| Galactose | 2.58 +/-0,02 | Nd |
| Glucose | 38.72 +/-0.48 | 45.89 +/-0.20 |
| Other | 4.89 +/-0.04 | 4.76 +/-0.02 |
| Total Carbohydrate | 65.06 +/-0.82 | 69.86 +/-0.31 |

Nd : not detected

**Supplementary Table S8**: Expert annotation and transcription profile of AA1s from *Pycnoporus coccineus* CIRM-BRFM 310

| ProteinID | CAZy  family | Predicted signal peptide | Base  Mean  Maltose | log2Fold  Change  PIN | Padj  PIN | padjBonf  PIN | log2Fold  Change  ASP | padj_ASP | padjBonf  ASP | Detected in secretomes |  |
| --- | --- | --- | --- | --- | --- | --- | --- | --- | --- | --- | --- |
| 1370215 | AA1 | NO | 0 | 0 | NA | NA | 0 | NA | NA | nd |  |
| 1363242 | AA1_1 | YES | 0 | 0 | NA | NA | 0 | NA | NA | nd |  |
| 1366139 | AA1_1 | YES | 38309 | -1.92 | 5.75E-07 | 1,51E-03 | -2.55 | 2.19E-11 | 2.64E-08 | maltose, pine, aspen | |
| 1434777 | AA1_1 | YES | 0 | 0 | NA | NA | 0 | NA | NA | maltose |  |
| 1452465 | AA1_1 | YES | 303 | 0 | NA | NA | 0 | NA | NA | pine, aspen |  |
| 1477425 | AA1_1 | YES | 2419 | -2.14 | 4.45E-17 | 3,88E-14 | -2.70 | 1.59E-26 | 6.43E-24 | maltose |  |
| 1425237 | AA1_2 | YES | 1644 | 0 | NA | NA | 0 | NA | NA | nd |  |

|  |
| --- |

JGI references for the 7 protein models of laccases (AA1_1) and ferroxidases (AA1_2). No laccase-like multicopper oxidases (AA1_3) was identified from the genome. The calls for signal peptides was done with signalp 3.0 from the Center for Biological Sequence Analysis (CBS) (1). *Pycnoporus coccineus* CIRM-BRFM 310 was grown in liquid cultures on 20 g/l maltose (control), 15 g/l pine or 15 g/l aspen as described in Materials and Methods. Total RNAs were extracted at day 3 from triplicates and sequenced using the Illumina HighSeq-2500 JGI platform. Gene transcripts were analyzed using DESeq2 as described in Materials and Methods. The table shows the mean number of reads for each gene in the maltose control condition and the fold change of transcript levels expressed as log2 on each substrate as compared to maltose. For comparison, the presence/absence of the proteins in the culture medium is indicated.

nd: not detected

NA: not applicable

padj: p value adjusted with the Benjamini-Hochberg method

padj_Bonf: p value adjusted with the Bonferroni method

**Reference**

1. Emanuelsson O, Runak S, von Heijne G, Nielson H. Locating proteins in the cell using TargetP, SignalP, and related tools. Nature Protocols. 2007 ; 2:953-71.

**Supplementary Table S9**: Expert annotation and transcription profile of AA2s from *Pycnoporus coccineus* CIRM-BRFM 310

| ProteinID | POD | Predicted signal peptide | Base  Mean  Maltose | log2Fold  Change  PIN | padj_PIN | padj_Bonf  PIN | log2Fold  Change  ASP | padj_ASP | padj_Bonf  ASP |
| --- | --- | --- | --- | --- | --- | --- | --- | --- | --- |
| 1369658 | MnP-short | YES | 0 | 0 | NA | NA | 0 | NA | NA |
| 1436321 | MnP-short | YES | 8113 | 6.52 | 1.38E-31 | 3.94E-29 | 6.25 | 3.50E-29 | 1.22E-26 |
| 1464049 | MnP-short | YES | 2996 | 0 | NA | NA | 6.91 | 7.16E-74 | 2.65E-72 |
| 1468611 | MnP-short | YES | 24345 | 9.08 | 3.82E-106 | 1.53E-105 | 9.71 | 1.67E-121 | 8.33E-121 |
| 779035 | LiP | YES | 0 | 0 | NA | NA | 0 | NA | NA |
| 859168 | LiP | YES | 0 | 0 | NA | NA | 0 | NA | NA |
| 1403742 | LiP | YES | 0 | 0 | NA | NA | 0 | NA | NA |
| 1431101 | LiP | YES | 0 | 0 | NA | NA | 0 | NA | NA |
| 1468768 | VP | YES | 0 | 0 | NA | NA | 0 | NA | NA |
| 1469331 | VP | YES | 0 | 0 | NA | NA | 0 | NA | NA |
| 1438352 | VP-atypical | YES | 1162 | -3.46 | 2.12E-26 | 9.13E-24 | -4.10 | 1.98E-36 | 4.64E-34 |

JGI references for the 11 protein models of class II peroxidase (short MnP, LiP, and typical and atypical VP). The calls for signal peptides was done with signalp 3.0 from the Center for Biological Sequence Analysis (CBS) (1). *Pycnoporus coccineus* CIRM-BRFM 310 was grown in liquid cultures on 20 g/l maltose (control), 15 g/l pine or 15 g/l aspen as described in Materials and Methods. Total RNAs were extracted at day 3 from triplicates and sequenced using the Illumina HighSeq-2500 JGI platform. Gene transcripts were analyzed using DESeq2 as described in Materials and Methods. The table shows the mean number of reads for each gene in the maltose control condition and the fold change of transcript levels expressed as log2 on each substrate as compared to maltose.

NA: not applicable

padj: p value adjusted with the Benjamini-Hochberg method

padj_Bonf: p value adjusted with the Bonferroni method

**Reference**

1. Emanuelsson O, Runak S, von Heijne G, Nielson H. Locating proteins in the cell using TargetP, SignalP, and related tools. Nature Protocols. 2007; 2 :953-71.

**Supplementary Table S10**: Characteristics of RNA libraries used in this study

| Sample | Library | Number of reads | Total number of reads which mapped as single end reads | Total number of reads which mapped as paired end reads | Number of reads which map to the same gene model | Number of reads which map to different gene models | Length to trim reads to before alignment |
| --- | --- | --- | --- | --- | --- | --- | --- |
| M1 | PAWY | 41183400 | 0 | 32853022 | 32667084 | 185938 | 70 |
| M2 | PAWO | 44697506 | 0 | 35011152 | 34689956 | 321196 | 70 |
| M3 | PAUX | 37877826 | 0 | 29889534 | 29739150 | 150384 | 70 |
| Pin1 | PAWN | 49277240 | 0 | 38654866 | 38432056 | 222810 | 70 |
| Pin2 | PAWA | 48035880 | 0 | 35009522 | 34831518 | 178004 | 78 |
| Pin3 | PAWC | 39786326 | 0 | 28399534 | 28249610 | 149924 | 70 |
| Asp1 | PAUW | 43175354 | 0 | 33765684 | 33502322 | 263362 | 78 |
| Asp2 | PAUY | 43535636 | 0 | 33947846 | 33521444 | 426402 | 70 |
| Asp3 | PAWW | 41087678 | 0 | 32699904 | 32532786 | 167118 | 78 |

M: Maltose, Pin: Pine, Asp : Aspen

**Supplementary Figure 11:** Heat map of the Pearson correlations between RNASeq read counts obtained from biological triplicates of the fungus grown on maltose (M), pine (Pin) and aspen (Asp)

M1 M2 M3 Pin1 Pin2 Pin3 Asp1 Asp2 Asp3

M1 1.00 0.99 0.95 0.51 0.46 0.54 0.54 0.55 0.56

M2 0.99 1.00 0.96 0.52 0.47 0.55 0.55 0.56 0.58

M3 0.95 0.96 1.00 0.53 0.49 0.57 0.56 0.57 0.59

Pin1 0.51 0.52 0.53 1.00 0.94 0.93 0.87 0.87 0.89

Pin2 0.46 0.47 0.49 0.94 1.00 0.91 0.79 0.80 0.83

Pin3 0.54 0.55 0.57 0.93 0.91 1.00 0.86 0.91 0.92

Asp1 0.54 0.55 0.56 0.87 0.79 0.86 1.00 0.97 0.97

Asp2 0.55 0.56 0.57 0.87 0.80 0.91 0.97 1.00 0.99

Asp3 0.56 0.58 0.59 0.89 0.83 0.92 0.97 0.99 1.00

**Supplementary Table S12**: Primers used for qPCR in this study

| Corresponding protein ID | Primer | Sequence (5’-3’) |
| --- | --- | --- |
| 1468611 | PcoAA2_F | GGC ATC TCC CCG AAG ATC |
|  | PcoAA2_R | CTC GTC GAT GAT CTC GTC A |
| 1395316 | PcoGH10_F | CCG GCT CTA CTG CCA ATG |
|  | PcoGH10_R | CCT CCA GAA TTG CAG TGT ACT GA |
| 688728 | PcoGH28_F | AGC GCC CAT GAT GAA GAT |
|  | PcoGH28_R | TCG TCA ATG ACA AGG TTG G |
| 1362558 | PcoP450_558_F | GCG TTT ACG CGA ACC TTA |
|  | PcoP450_558_R | TTC CAG TAG ATG GGC TTG CT |
| 1297918 | PcoP450_918_F | GGA GGC GCT CAA GAA CAT AG |
|  | PcoP450_918_R | AGG CCA CGG AAC CAG TC |
| 1362447 | PcoP450_447_F | GTC CTT CGT CAG TGG TTC G |
|  | PcoP450_447_R | GAC GAG GTG TTC GTG GAA TT |
| 1437297 | PcoUNK_F | CGG ATT CTA CCC GAC ATC TGT |
|  | PcoUNK_R | CAA CAA TCC GCA TCA CAT TC |
| 1368318 | PcoAA3-318F | GCTCCTCAACAAGCTCAACA |
|  | PcoAA3-318R | CTGAACATCCTTGACGCCAC |
| 1466495 | PcoAA9-495F | AGCGGCAGACTCCATCTAT |
|  | PcoAA9-495R | ACCAGGCTGGAGGCATC |
