# Supplementary material for: Enhanced degradation of softwood versus hardwood by the white-rot fungus Pycnoporus coccineus
Source: Biotechnol Biofuels. 2015 Dec 18;8:216. doi: 10.1186/s13068-015-0407-8 (PMC4683735; doi:10.1186/s13068-015-0407-8)
Supplement: Supplementary file 3 — 10.1186/s13068-015-0407-8 Transcripts abundancies determination of a set of genes by qPCR and RNAseq. Full bars indicate qPCR results, striped bars indicate RNASeq results. Expression levels on pine are shown in blue, expression levels on aspen are shown in green. Results are expressed as log2 of enrichment as compared to maltose control. UNK: 1437297, 447: 1362447, 558:1362558, 918:1297918, AA9: 1466495, AA2: 1468611, GH10: 1395316, GH28: 688728. Figure S2. Electrophoresis profile of P. coccineus secretomes. MW: Molecular weight in kDa (lane 1), secreted proteins after 3 day cultivations on maltose (lane 2), pine (lane 3), and aspen (lane 4). Figure S3. HPAEC profiles of soluble fractions after saccharification of aspen (A) and pine (B) using P. coccineus secretomes highlighting glucose release (star). Figure S4. Homology models for the molecular structures of class I and II heme peroxidases from the P. coccineus CIRM-BRFM 310 genome. Ligninolytic peroxidases, including models for 1431101 (A), 1403742 (B), 779035 (C) and 859168 (D) - harboring an exposed tryptophan potentially involved in oxidation of high redox-potential substrates, MnP-short models 1468611(E), 1436321 (F), 1464049 (G) and 1369658 (H) - harboring a putative Mn2+ oxidation site (formed by two glutamates and one aspartate), two VP models - 1468768 (I) and 1469331 (J) - harboring the two catalytic sites described for LiPs and MnPs, one atypical VP - 1438352 (K) - containing an atypical Mn2+ oxidation site formed by two aspartates and one glutamate; and CCP 1449695 (L). [file 13068_2015_407_MOESM3_ESM.docx]

**Additional File 3 : Supplementary Figures 1 to 4**


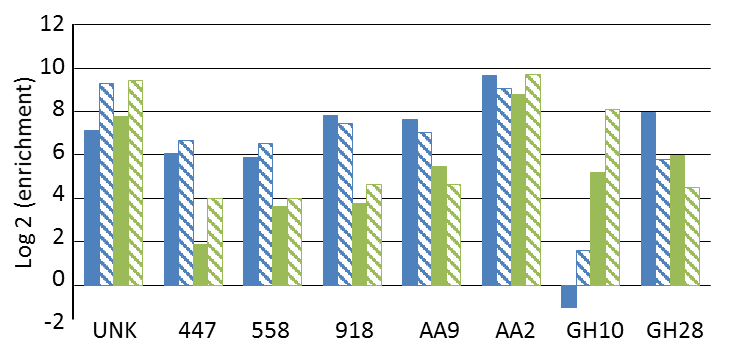


**Supplementary Figure s1: Transcripts abundancies determination of a set of genes by qPCR and RNAseq.** Full bars indicate qPCR results, striped bars indicate RNASeq results. Expression levels on pine are shown in blue, expression levels on aspen are shown in green. Results are expressed as log2 of enrichment as compared to maltose control. UNK:1437297, 447: 1362447, 558:1362558, 918:1297918, AA9: 1466495, AA2: 1468611, GH10: 1395316, GH28: 688728.


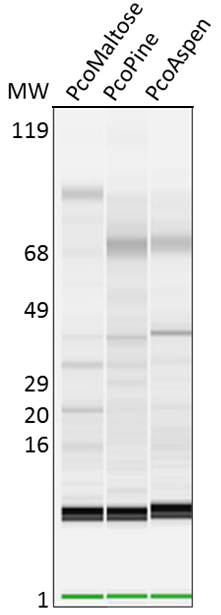


**Supplementary Figure S2: Electrophoresis profile of *P. coccineus* secretomes.** MW: Molecular weight in kDa (lane 1), secreted proteins after 3 day cultivations on maltose (lane 2), pine (lane 3), and aspen (lane 4).


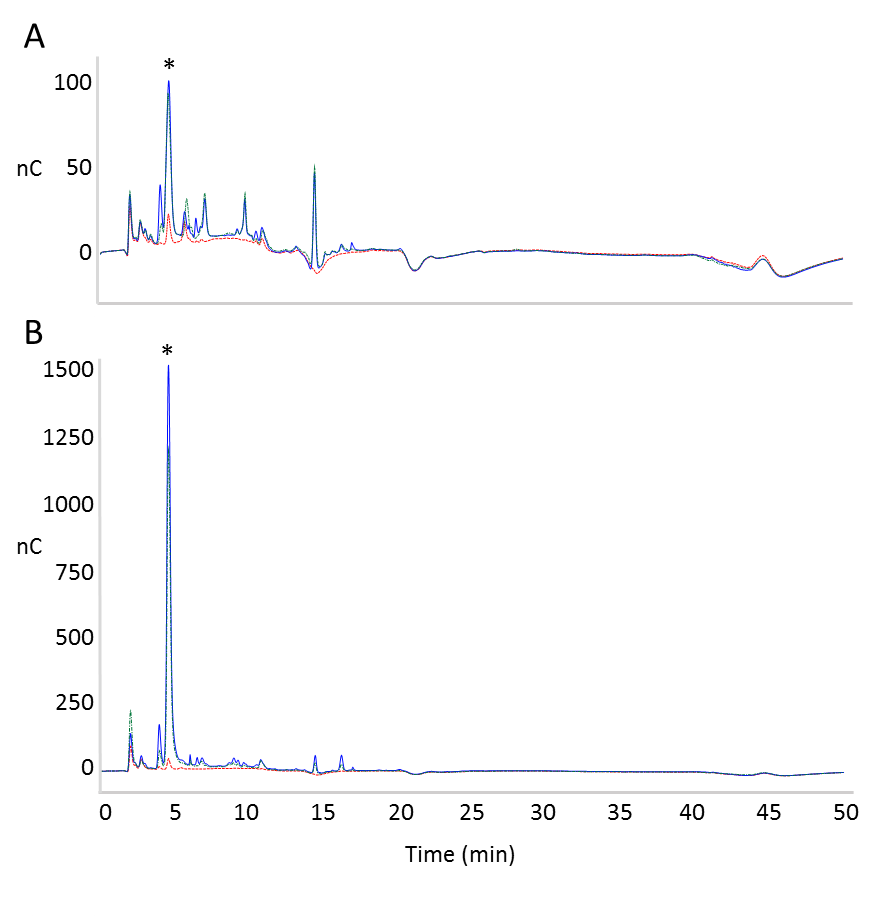


**Supplementary Figure S3:** **HPAEC profiles of soluble fractions after saccharification of aspen (A) and pine (B) using *P. coccineus* secretomes.** Glucose release is highlighted with a star.

**
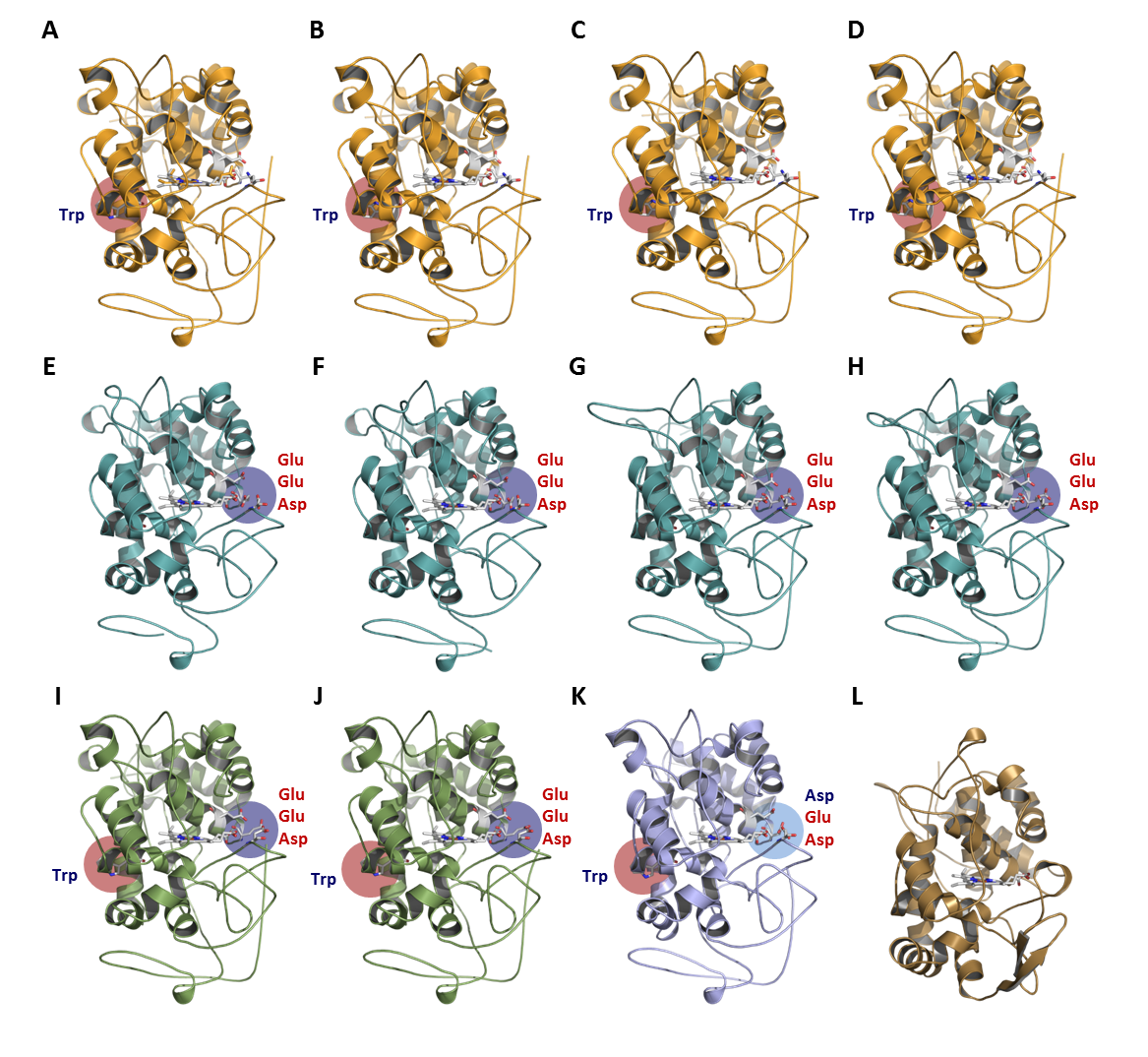
**

**Supplementary Figure S4: Homology models for the molecular structures of class I and II heme peroxidases from the *P. coccineus* CIRM-BRFM 310 genome.** Ligninolytic peroxidases, including models for 1431101 (A), 1403742 (B), 779035 (C) and 859168 (D) - harboring an exposed tryptophan potentially involved in oxidation of high redox-potential substrates, MnP-short models 1468611 (E), 1436321 (F), 1464049 (G) and 1369658 (H) - harboring a putative Mn^2+^ oxidation site (formed by two glutamates and one aspartate), two VP models - 1468768 (I) and 1469331 (J) - harboring the two catalytic sites described for LiPs and MnPs, one atypical VP - 1438352 (K) - containing an atypical Mn^2+^ oxidation site formed by two aspartates and one glutamate; and CCP 1449695 (L).
